# Reactive Oxygen Species Production Is Responsible for Antineoplastic Activity of Osmium, Ruthenium, Iridium and Rhodium Half-Sandwich Type Complexes with Bidentate Glycosyl Heterocyclic Ligands in Various Cancer Cell Models

**DOI:** 10.3390/ijms23020813

**Published:** 2022-01-12

**Authors:** István Kacsir, Adrienn Sipos, Attila Bényei, Eszter Janka, Péter Buglyó, László Somsák, Péter Bai, Éva Bokor

**Affiliations:** 1 Department of Organic Chemistry, University of Debrecen, P.O. Box 400, H-4002 Debrecen, Hungary; kacsir.istvan@science.unideb.hu (I.K.); somsak.laszlo@science.unideb.hu (L.S.); 2 Doctoral School of Chemistry, University of Debrecen, P.O. Box 400, H-4002 Debrecen, Hungary; 3 Department of Medical Chemistry, Faculty of Medicine, University of Debrecen, Egyetem tér 1., H-4032 Debrecen, Hungary; siposadri@med.unideb.hu; 4 Department of Physical Chemistry, Faculty of Sciences and Technology, University of Debrecen, Egyetem tér 1., H-4032 Debrecen, Hungary; benyei.attila@science.unideb.hu; 5 Department of Dermatology, Faculty of Medicine, University of Debrecen, Egyetem tér 1., H-4032 Debrecen, Hungary; janka.eszter@med.unideb.hu; 6 Department of Inorganic & Analytical Chemistry, Faculty of Sciences and Technology, University of Debrecen, Egyetem tér 1., H-4032 Debrecen, Hungary; buglyo@science.unideb.hu; 7 NKFIH-DE Lendület Laboratory of Cellular Metabolism, H-4032 Debrecen, Hungary; 8 Research Center for Molecular Medicine, Faculty of Medicine, University of Debrecen, Egyetem tér 1., H-4032 Debrecen, Hungary

**Keywords:** osmium complex, iridium complex, ruthenium complex, rhodium complex, half-sandwich, cooperative binding, reactive oxygen species production, glycosyl heterocycle, oxadiazole, triazole, ovarian cancer, Hodgkin’s lymphoma, osteosarcoma

## Abstract

Platinum complexes are used in chemotherapy, primarily as antineoplastic agents. In this study, we assessed the cytotoxic and cytostatic properties of a set of osmium(II), ruthenium(II), iridium(III) and rhodium(III) half-sandwich-type complexes with bidentate monosaccharide ligands. We identified 5 compounds with moderate to negligible acute cytotoxicity but with potent long-term cytostatic activity. These structure-activity relationship studies revealed that: (1) osmium(II) *p*-cymene complexes were active in all models, while rhodium(III) and iridium(III) Cp* complexes proved largely inactive; (2) the biological effect was influenced by the nature of the central azole ring of the ligands—1,2,3-triazole was the most effective, followed by 1,3,4-oxadiazole, while the isomeric 1,2,4-oxadiazole abolished the cytostatic activity; (3) we found a correlation between the hydrophobic character of the complexes and their cytostatic activity: compounds with *O*-benzoyl protective groups on the carbohydrate moiety were active, compared to *O*-deprotected ones. The best compound, an osmium(II) complex, had an IC_50_ value of 0.70 µM. Furthermore, the steepness of the inhibitory curve of the active complexes suggested cooperative binding; cooperative molecules were better inhibitors than non-cooperative ones. The cytostatic activity of the active complexes was abolished by a lipid-soluble antioxidant, vitamin E, suggesting that oxidative stress plays a major role in the biological activity of the complexes. The complexes were active on ovarian cancer, pancreatic adenocarcinoma, osteosarcoma and Hodgkin’s lymphoma cells, but were inactive on primary, non-transformed human fibroblasts, indicating their applicability as potential anticancer agents.

## 1. Introduction

Metal complex-based drugs are frequently used in antineoplastic therapy [1]. Currently, platinum complexes (cisplatin, oxaliplatin, carboplatin) are EMA/FDA registered; however, other platinum-group metal ions can form compounds that have oncopharmacological relevance [1,2]. Platinum complexes play a very important role in modern chemotherapy; nevertheless, platinum complexes also have ototoxicity [3,4] and, in most neoplasias, platinum resistance occurs over time [5,6,7]. These unresolved issues call for novel therapeutic options and the use of alternative solutions, such as organometallic complexes of other platinum-group metals. There are numerous complexes of ruthenium [1,8,9,10,11,12,13,14], osmium [2,9,12,13,14,15,16,17], rhodium [13,18,19], or iridium [2,12,13,18,20] that are used in oncological settings. In fact, one ruthenium complex, IT-139, has passed clinical phase-I studies and is intended for use in colorectal cancer [8]. Some of the complexes developed using other platinum-group metals apparently have advantageous properties compared to Pt-based drugs, e.g., better delivery properties [1], improved cellular entry under hypoxia [21,22], and a better toxicity profile [23,24,25,26]. These complexes can easily be directed to their biological targets by coupling to bait molecules, such as biotin, transferrin, hormones, or carbohydrates [1,27,28,29].

**Figure 1:**
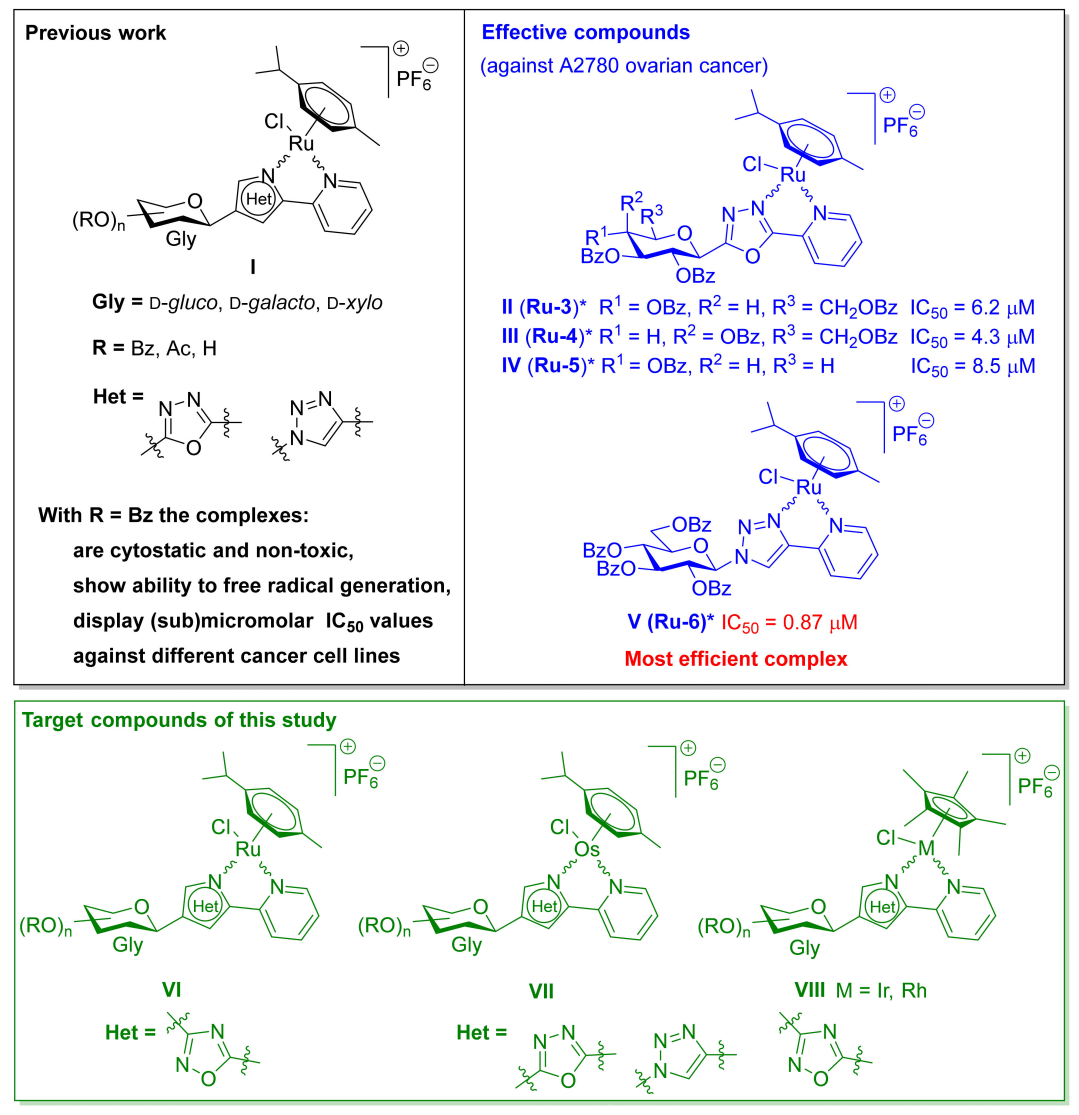
Highlights of our recent study and target compounds of this work. (* To maintain a uniform numbering scheme, these compounds will appear as **Ru-3–Ru-6** in this paper, referring to the structural similarities of the complexes with other metals.)

In our previous study [30], a series of half-sandwich-type Ru(II) complexes (Figure 1, **I**) were shown to be cytostatic and non-toxic, be able to generate free radicals, and to display cooperative binding in (sub)micromolar concentrations against different cancer cell lines (ovarian and breast cancer, pancreatic adenocarcinoma and glioblastoma). More specifically, *O*-perbenzoylated (as opposed to its *O*-peracetylated and *O*-unprotected counterparts) *C*-glycosyl 1,3,4-oxadiazole ligands **II–IV** exhibited low micromolar efficiency against, e.g., the A2780 ovarian cancer cell line. Exchange of the oxadiazole part to an *N*-glycosidically linked 1,2,3-triazole led to the most efficient compound, **V**, with a submicromolar IC_50_ value.

It was also evidenced that these ruthenium complexes exerted biological activity through reactive species production [30], which is similar to other ruthenium complexes [23,27,31,32]. To a lesser extent, PARP activation may also contribute to cytostasis [30], a feature that was observed for a subset of ruthenium complexes [31,32,33,34].

In this study, we assessed a novel set of molecules that have a similar gross buildup to those in our previous report (Figure 1, **I–V**) [30]; however, the compounds have been changed in crucial structural elements: complexes of additional platinum-group metals with different coordinating hexahapto arene and pentahapto arenyl groups, and modified *C*-glycosyl heterocyclic chelators were prepared and studied as to their effects on the biological activity of these molecules. These particular modifications are summarized in target compounds **VI–VIII** in Figure 1. Replacement of the central metal ion and the hexahapto-coordinating arene by an arenyl ligand may result in significant changes to the lipophilicity, membrane permeability and kinetic properties of the complexes; therefore, in the present study, we aimed to explore the effect of the use of other platinum-group metals (Os, Rh, Ir), having also proven their anticancer potential in the form of half-sandwich-type organometallic compounds [2,9,10,12,13,14,15,16,17,18,19,20]. In addition, to investigate the effect of the important central heterocyclic moiety in the carbohydrate ligand, 1,2,4-oxadiazole derivatives were also included.

## 2. Results

### 2.1. Chemistry

In our previous study [30], the central heterocyclic ring of the carbohydrate ligand appeared most important (*c.f.* oxadiazole **II** = **Ru-3** and **V** = **Ru-6** in Figure 1), therefore, to further investigate the effect of the five-membered heterocycle to the biological activity of such types of complexes, the replacement of the 1,3,4-oxadiazole ring with an isomeric 1,2,4-oxadiazole was envisaged.

**Scheme 1:**
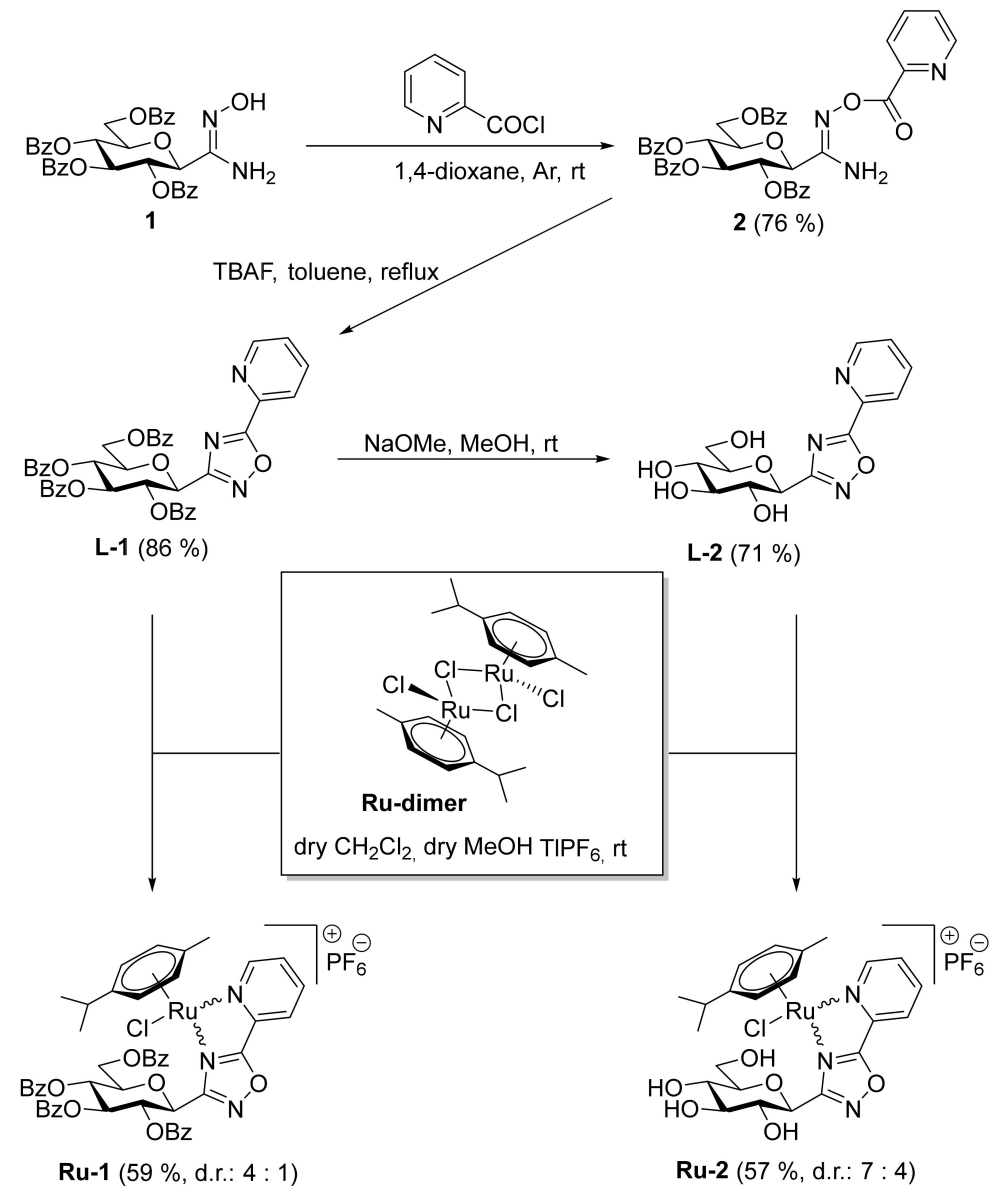
Preparation of 3-(β-D-glucopyranosyl)-5-(2-pyridyl)-1,2,4-oxadiazoles (Compounds **2** and **L-1** were first synthesized by É.B. during a stay in the laboratory of Dr. Jean-Pierre Praly and Dr. Sebastien Vidal in the Institut de Chimie et Biochimie Moléculaires et Supramoléculaires (ICBMS-UMR 5246), Université Claude-Bernard Lyon 1 (France)) and their [(η^6^-*p*-cym)Ru^II^(N-N)Cl]PF_6_ complexes.

For the preparation of the corresponding *C*-glucosyl 1,2,4-oxadiazole, a literature method was adapted [35,36]. Thus, the *O*-perbenzoylated *C*-glucosyl formamidrazone [36] (Scheme 1, **1**) was converted with picolinoyl chloride to the corresponding *O*-picolinoyl amidoxime **2**, whose TBAF-mediated intramolecular ring-closure furnished the desired 1,2,4-oxadiazole-based ligand, **L-1**. For comparative studies, the *O*-deprotected derivative **L-2** was also prepared by the Zemplén-type debenzoylation of compound **L-1**. The ligands **L-1** and **L-2** were then subjected to complexation, carried out with a dichloro(η^6^-*p*-cymene)ruthenium(II) dimer ([(η^6^-*p*-cym)RuCl_2_]_2_, **Ru-dimer**) in the presence of TlPF_6_, to yield the test compounds **Ru-1** and **Ru-2**, respectively, as diastereomeric mixtures (Scheme 1).

Since the most effective [(η^6^-*p*-cym)Ru^II^(N-N)Cl]PF_6_ complexes have 2-pyridyl-substituted glycosyl azole ligands (Figure 1, **II** = **Ru-3**, **III** = **Ru-4**, **IV** = **Ru-5**, **V** = **Ru-6**), as published earlier [30], replacement of the Ru(II) to Os(II) ion was also carried out. The previously prepared *O*-perbenzoylated 2-(β-D-glycopyranosyl)-5-(2-pyridyl)-1,3,4-oxadiazoles [30] (**L-3‒L5**) and 1-(β-D-glucopyranosyl)-4-(2-pyridyl)-1,2,3-triazole [30] (**L-6**) were treated with dichloro(η^6^-*p*-cymene)osmium(II) dimer [37] ([(η^6^-*p*-cym)OsCl_2_]_2_, **Os-dimer**) and TlPF_6_ was used for chloride abstraction. The target complexes, **Os-3‒Os-6**, were obtained as mixtures of diastereoisomers in good yields (Table 1).

In order to extend our SAR study, cationic half-sandwich-type Ir(III) and Rh(III) complexes with pentamethylcyclopentadienyl moiety (Cp*) and glycosyl azole-type bidentate ligands were also synthesized and tested.

For the formation of the Ir(III) complexes, a wider range of glycosyl azoles, including *O*-perbenzoylated, *O*-peracetylated and *O*-unprotected derivatives, was used (Table 2) to assess a potential synergism or antagonism of the sugar residues with the not previously applied Cp* arenyl moiety. Thus, the ligands **L-1‒L-11** [30] were reacted with dichloro(pentamethylcyclopentadienyl)iridium(III) dimer ([(η^5^-Cp*)IrCl_2_]_2_, **Ir-dimer**) under the same conditions described above, resulting in the appropriate diastereomeric complexes **Ir-1‒Ir-11** in moderate to high yields.

The synthesis of certain selected representatives of the Rh(III) analogs (Table 3) was accomplished via the treatment of the corresponding dichloro(pentamethylcyclopentadienyl)rhodium(III) dimer ([(η^5^-Cp*)RhCl_2_]_2_, **Rh-dimer**) with *O*-benzoyl protected *C*-glycosyl-1,2,4-oxadiazoles **L-3‒L-5 [30]** and *N*-glucosyl-1,2,3-triazole **L-6** [30] and TlPF_6_. The incorporation of these monosaccharide-containing N,N-chelators to the coordination sphere of the Rh(III) afforded the desired PF_6_ salts of the [(η^5^-Cp*)Rh^III^(N-N))Cl]^+^ complexes in excellent yields.

The structure elucidation of the prepared compounds was primarily based on ^1^H and ^13^C NMR spectroscopic measurements. In general, the transformation of the starting dimeric platinum metal complexes (**Ru-dimer**, **Os-dimer**, **Ir-dimer** or **Rh-dimer**) into the desired [(η^6^-*p*-cym)M^II^(N-N)Cl]PF_6_ (**Ru-1**, **Ru-2**, **Os-3‒Os-6**) and [(η^5^-Cp*)M^III^(N-N)Cl]PF_6_ (**Ir-1‒Ir-12**, **Rh-1‒Rh-6**) caused noticeable changes to the chemical shifts of the signals of the η^6-^arene and η^5^-arenyl moieties. For instance, 4–6 ppm downfield shifts were observed in the case of the aromatic quaternary carbon peaks for compounds with *p*-cymene moiety, and 3–4 ppm shifts for the Cp*-containing derivatives. Similar to the earlier obtained half-sandwich Ru(II) complexes [30], significant downfield shifts of the pyridine C-6 resonance of the ligands (Δ = δ_complex_–δ_ligand_ = +1.5 − +8.0 ppm) could be seen as a consequence of the complex formation. In addition, the pyridine H-6 peak of the new Ru(II) and Os(II) complexes (**Ru-1**, **Ru-2**, **Os-3‒Os-6**), as well as the same signal of the Ir(III) derivatives with *O*-deprotected ligands (**Ir-2**, **Ir-8‒Ir-10**, **Ir-12**), showed a downfield shift relative to that of the corresponding free ligands, **L-1**, **L-2**, **L-3‒L-6**, **L-8‒L10**, **L-12** (Δ = δ_complex_–δ_ligand_ = +0.35 − +0.85 ppm). A similar trend could not be identified in the case of the Ir(III) and Rh(III) complexes of the *O*-peracylated ligands (**Ir-1**, **Ir-3‒Ir-7**, **Ir-11**, **Rh-3‒Rh-6**). The incorporation of monosaccharide-based ligands into the coordination spheres could also be seen among others from the downfield shift of the H-1′ protons of the sugar skeletons (~0.1–0.2 ppm).

**Figure 2:**
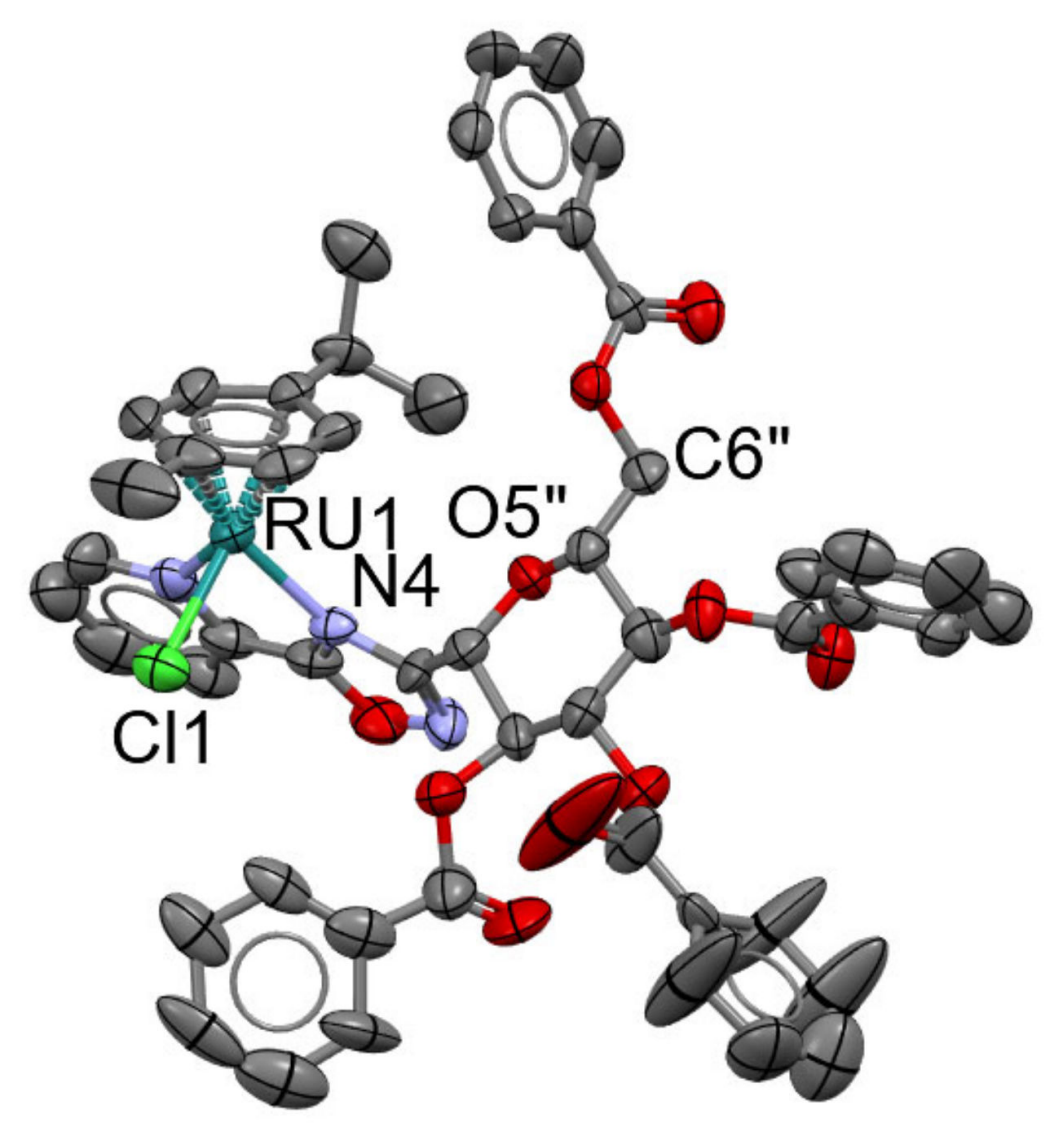
ORTEP view of **Ru-1** at a 50% probability level, with a partial numbering scheme. Only one of the complexes in the asymmetric unit is shown. Hydrogen atoms and the PF_6_^−^ counter ion, as well as solvent chloroform molecules, are omitted for clarity.

**Figure 3:**
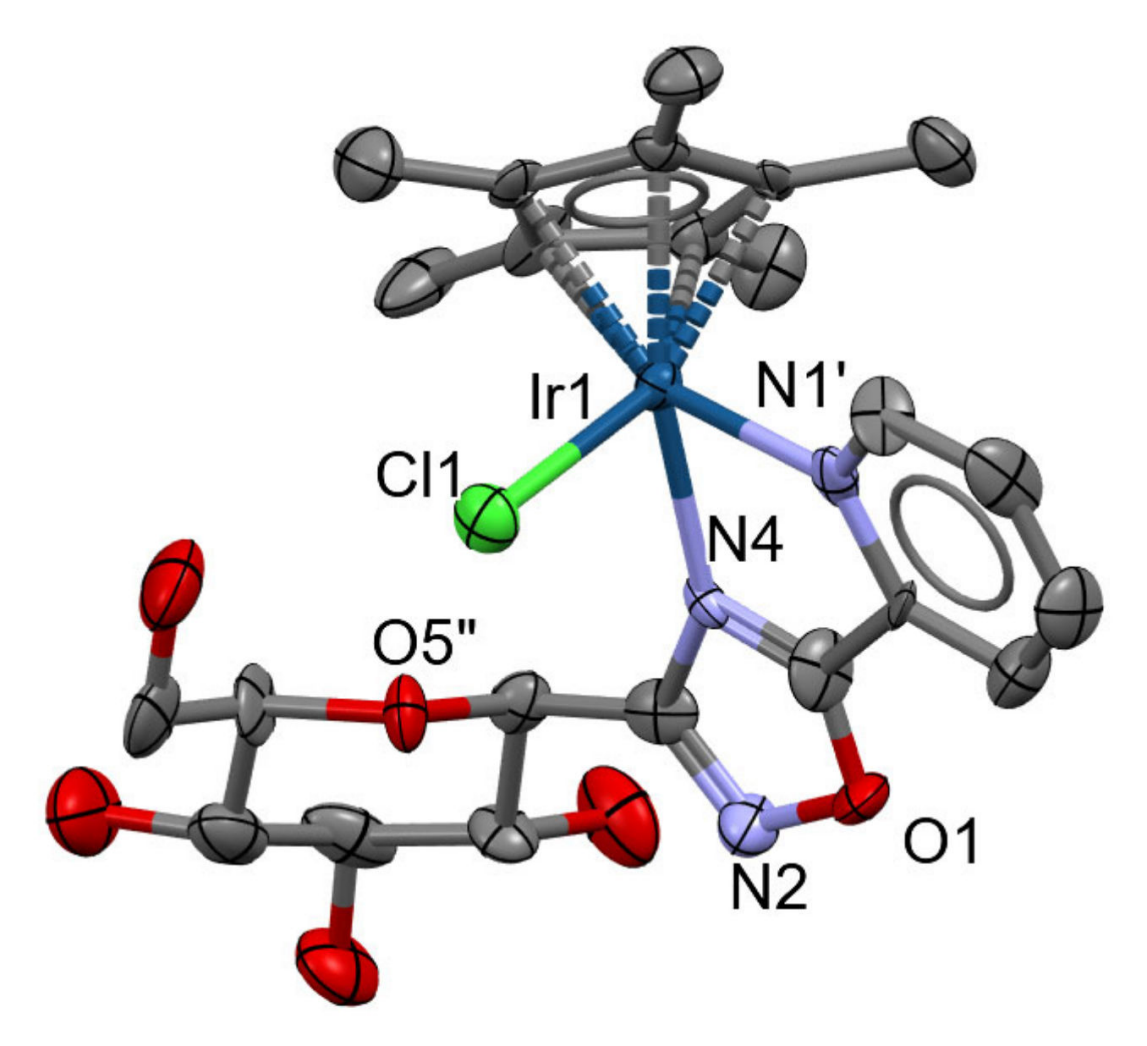
ORTEP view of **Ir-2** at a 50% probability level, with a partial numbering scheme. Hydrogen atoms and the PF_6_^-^ counter ion are omitted for clarity.

The mode of the coordination and the existence of the 5-membered chelates were also proven by X-ray crystallography of the complexes **Ru-1** and **Ir-2**. One isomer of both of these derivatives could be obtained as single crystals by the slow evaporation of the appropriate mother liquors (**Ru-1** from CHCl_3_, **Ir-2** from MeOH). The crystal and molecular structures of compounds **Ru-1** (Figure 2) and **Ir-2** (Figure 3) were established by single-crystal X-ray diffraction analysis. The results for the X-ray diffraction structure determinations were very good, according to the Checkcif functionality of the PLATON software (Utrecht University, Utrecht, The Netherlands) [38]. The reasons for A- and B-level errors are the presence of a very heavy element (ruthenium or iridium), the undefined orientation of hydroxyl groups in **Ir-2**, and the slight disorder of solvent chloroform molecules in **Ru-1**. All these errors were answered in the CIF file. In the case of **Ru-1**, there are two complexes with counter ions and five chloroform molecules in the asymmetric unit. The complexes had slightly different conformation, as is shown in the supporting material in Figure S2. Further experimental details of the crystal parameters, data collection, and the results of structure refinement are given in the Materials and Methods section and in Table 6. The absolute configuration of the stereogenic centers was also verified using the anomalous dispersion method, as is proven by the Flack parameters being close to 0 (Table 6). This is in full agreement with the synthetic route.

The coordination of the ruthenium or iridium center is similar to that found for half-sandwich complexes, considering the orientation of the chloride, aryl and other ligands around the transition metal. However, these are the first ruthenium and iridium complexes of *N*-coordinated oxadiazole. A search of the Cambridge Structural Database (Ver. 5.42 Updates, September 2021) [39] revealed 62 hits of oxadiazole complexes of Pt, Pd, Cu, Zn, Ag, Ni, Co and Au. However, it is only in 16 complexes that one can find double bonds for both N2-C3 and N4-C5, and, in all cases, the oxadiazole ring is parallel or perpendicular with respect to the other ligand(s). Moreover, in the **Ir-2** structure, the angle of the plane of the oxadiazole and aryl rings is 52 degrees, while in the case of **Ru-1**, the angles for the two moieties are 57 and 62 degrees because of the steric requirement of the bulky aryl ligand. The supplementary crystallographic data for each compound can be obtained free of charge from the Cambridge Crystallographic Data Center via http://www.ccdc.cam.ac.uk/data_request/cif (accessed on 6 January 2022), using the reference deposition numbers of 2129695 and 2129694 for **Ru-1** and **Ir-2**, respectively.

### 2.2. Biological Characterization of the Complexes

#### 2.2.1. Selection of Biologically Active Complexes

The newly synthesized compounds were assessed using A2780 ovarian cancer cells that proved suitable and were sensitive to molecules with a similar structure to those investigated in our previous study [30]. The compounds were tested between 0.001694 and 33.3333 µM, using a 4-hour-long MTT test to assess rapid toxicity and a 48-hour-long SRB assay to assess the long-term effects of the inhibition of proliferation (cytostasis). The MTT assay assesses the activity of mitochondrial complex I and detects rapid, direct toxicity, as well as indicating apoptosis and necrosis [40,41]. The SRB assay measures the total protein content that is proportional to cell numbers; therefore, the assay can detect changes in cell numbers and can thus be used as a readout for cell proliferation [42]. The SRB assay was performed after 48 h of treatment (with the exception of L428 cells, which were tested for 96 h due to their slower proliferation rate). In this setting, the SRB assay informs us of long-term changes to cell proliferation. We assessed the maximal inhibition within that concentration range, and descriptive statistics were performed. We performed a one-way ANOVA, followed by the Kruskal–Wallis test, regardless of the normal or non-normal distribution of the data. Due to the large number of significance values, the results of the significance calculations are provided in an Excel spreadsheet at https://figshare.com/s/28d14597d5041f08eb08 (accessed on 6 January 2022), among other primary data of the study. Where possible, nonlinear regression was performed on the curves to determine the IC_50_ values ((inhibitor) vs. response—variable slope (four parameters) method with Graphpad v8.0.1). These data are compiled in Table 4 and Table 5. In Table 5, we added the IC_50_ values that we determined for a set of 4 active ruthenium half-sandwich complexes we identified previously [30], to be used in subsequent structure-activity relationship (SAR) analyses.

In total, we assessed 27 complexes and free ligands. The free ligands, **L-3‒L-12**, were tested previously and were found to have no biological activity [30]. The [(η^6^-*p*-cym)Ru^II^(N-N)Cl]PF_6_-type complexes of these ligands were also assessed previously [30] in the same system and under the same conditions; hence, we included the data on the most active compounds, **Ru-3‒Ru-6**, in the SAR analysis.

**Figure 4:**
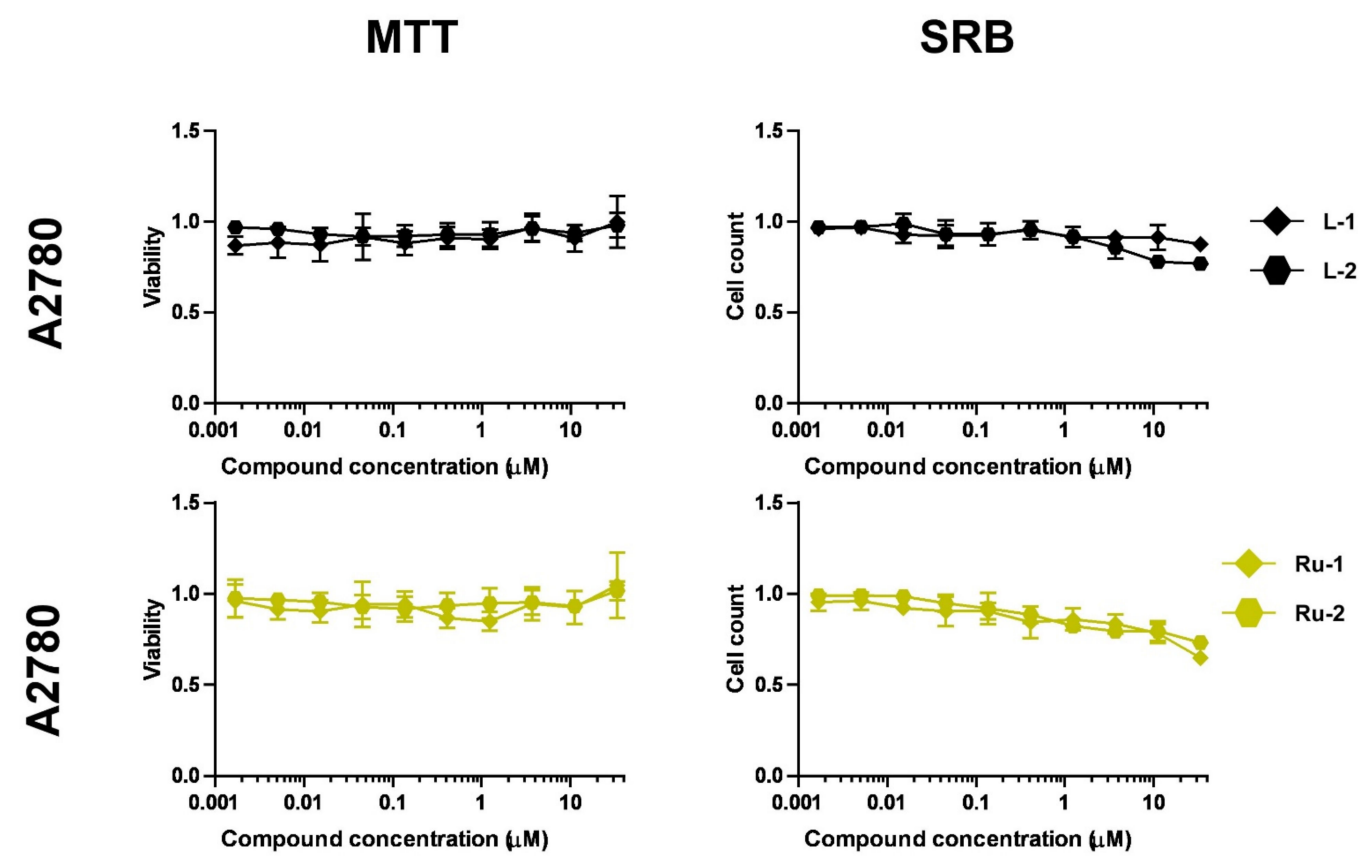
Assessment of free ligands **L-1** and **L-2** and ruthenium complexes **Ru-1** and **Ru-2** in terms of cytotoxic and cytostatic activity. Briefly, 3 × 10^3^ A2780 cells were plated onto 96-well plates. Cells were treated with the compounds in the concentrations indicated, for either 4 h for an MTT assay or 48 h for an SRB assay. Data are represented as average ± SD, from three biological replicates; individual assays were performed in duplicate. Values were normalized for vehicle-treated cells; absorbance for vehicle-treated cells is equal to 1. A one-way ANOVA, followed by a Kruskal–Wallis test, was performed individually for each compound. The results of the statistical analysis are presented in an Excel spreadsheet at https://figshare.com/s/28d14597d5041f08eb08 (accessed on 6 January 2022). Nonlinear regression was performed on the data.

The two new 1,2,4-oxadiazole-containing ligands (**L-1** and **L-2**) were assessed in detail in our model system and were found to be practically inactive in both the MTT and SRB assays, which is similar to the other free ligands that were tested earlier [30]. Their Ru(II) complexes (**Ru-1** and **Ru-2**, respectively) were assessed; however, neither of them showed considerable activity in either the MTT or SRB assays (Figure 4).

**Figure 5:**
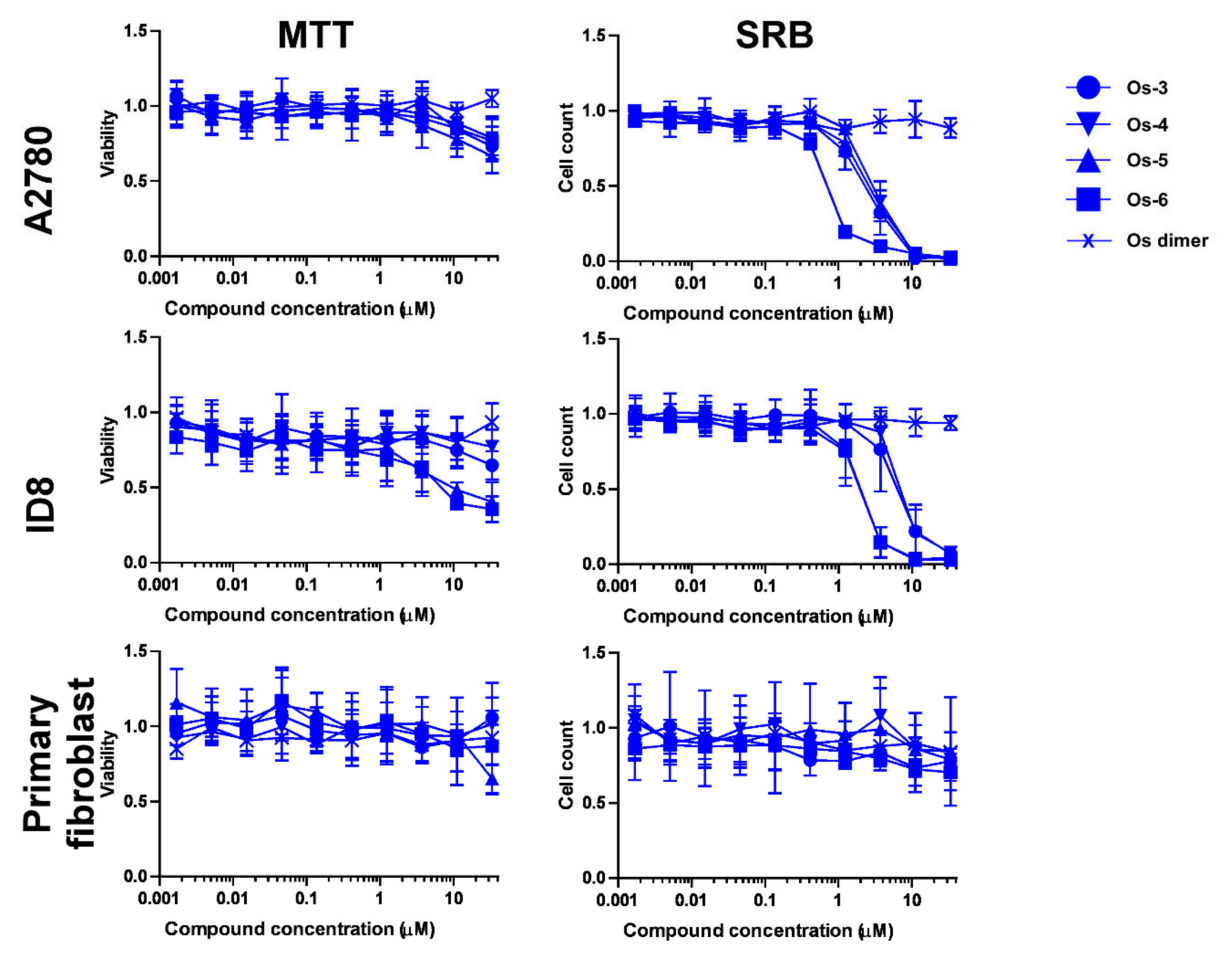
Assessment of the osmium complexes **Os-3‒Os-6** for cytotoxic and cytostatic activity. Briefly, 3 × 10^3^ A2780 cells, 2 × 10^3^ ID8 cells, and 4 × 10^3^ primary fibroblasts were plated onto 96-well plates. Cells were treated with the compounds in the concentrations indicated for either 4 h for an MTT assay or for 48 h for an SRB assay. Data are represented as average ± SD, from three biological replicates (in SRB assays for ID8, five biological replicates were used); individual assays were performed in duplicate. Values were normalized for vehicle-treated cells; absorbance for vehicle-treated cells is equal to 1. A one-way ANOVA, followed by a Kruskal-Wallis test, was performed individually for each compound. The results of the statistical analysis are presented in an Excel spreadsheet at https://figshare.com/s/28d14597d5041f08eb08 (accessed on 6 January 2022). Nonlinear regression was performed on the data.

Next, we assessed the osmium(II) complexes (**Os-3‒Os-6**), together with the chloro-bridged osmium dimer (**Os-dimer**) that was used to synthesize the title compounds. The complexes exerted only mild toxicity on A2780 cells, as judged from the MTT assays after 4 h of treatment (Figure 5). However, in the SRB assays, **Os-3**, **Os-4**, **Os-5** and **Os-6** exerted cytostatic activity, **Os-6** being the strongest with a submicromolar IC_50_ value (0.7 µM) (Figure 5, Table 5). The osmium dimer was inactive (Figure 5). We assessed whether the cytostatic ability of the complexes was specific for the A2780 cell lines by testing them on another ovarian cancer cell line, ID8. The **Os-3**, **Os-5** and **Os-6** complexes showed a cytotoxic capacity to a greater extent than on A2780 cells. All complexes showed cytostatic activity on ID8 cells (Figure 5, Table 5). The **Os-dimer** was inactive (Figure 5). Finally, we wanted to test whether the mild cytotoxic and strong cytostatic activity of the complexes was specific for neoplastic cells or if it could be elicited on non-transformed cells. For that purpose, we tested the compounds on human primary fibroblasts. None of the complexes, **Os-3**‒**Os-6**, or the **Os-dimer** exerted cytotoxic or cytostatic activity on fibroblasts (Figure 5).

**Figure 6:**
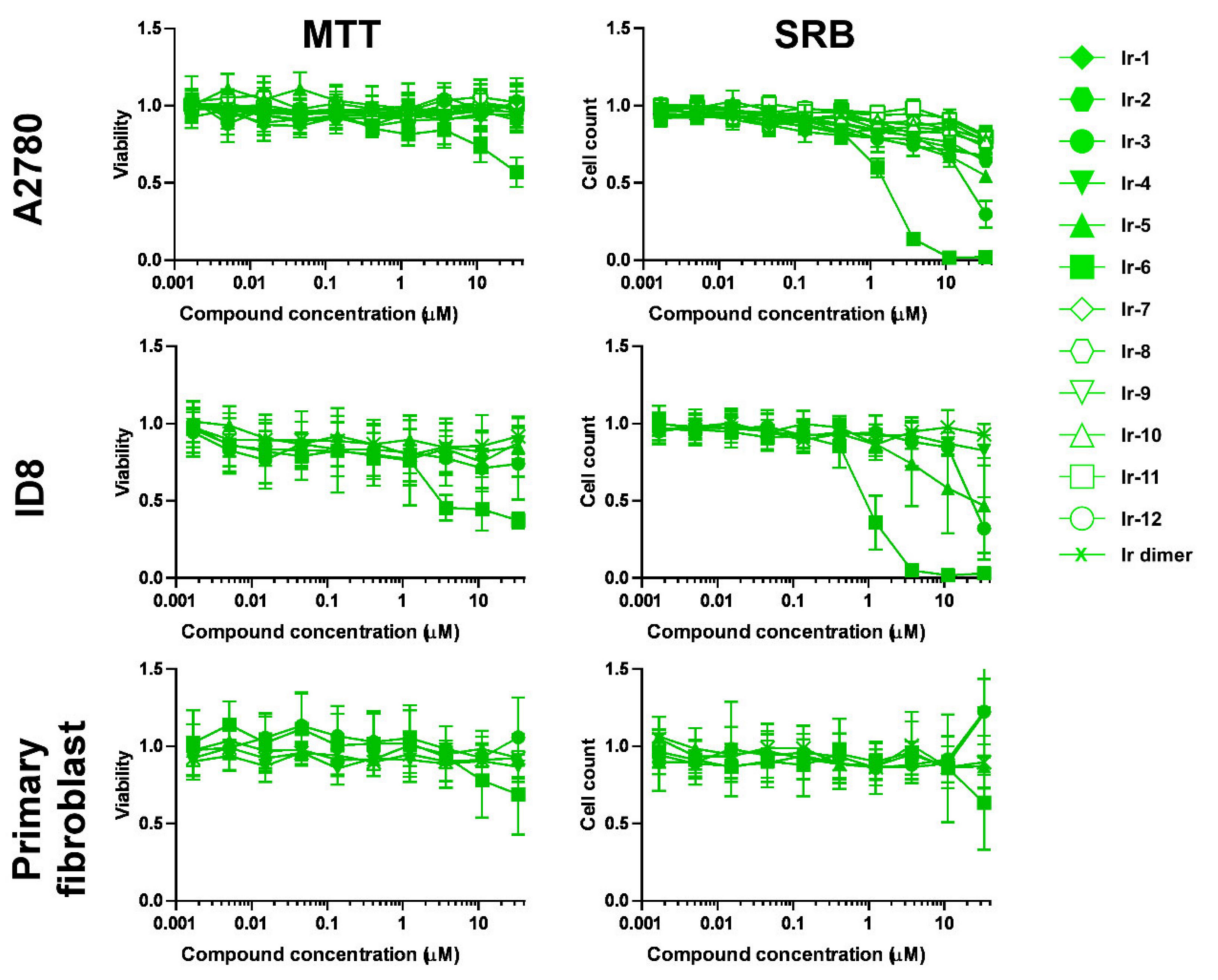
Assessment of iridium complexes **Ir-1‒Ir-12** for cytotoxic and cytostatic activity. Briefly, 3 × 10^3^ A2780 cells, 2 × 10^3^ ID8 cells, and 4 × 10^3^ primary fibroblasts were plated onto 96-well plates. Cells were treated with the compounds in the concentrations indicated for either 4 h for an MTT assay or for 48 h for an SRB assay. Data are represented as average ± SD, from three biological replicates, individual assays were performed in duplicates. Values were normalized for vehicle-treated cells; absorbance for vehicle-treated cells is equal to 1. A one-way ANOVA, followed by a Kruskal-Wallis test, was performed individually for each compound. The results of the statistical analysis are presented in an Excel spreadsheet at https://figshare.com/s/28d14597d5041f08eb08 (accessed on 6 January 2022). Nonlinear regression was performed on the data.

Subsequently, we assessed the iridium(III) complexes (**Ir-1‒Ir-12**), together with the chloro-bridged iridium dimer precursor (**Ir-dimer**) that was used to synthesize the appropriate compounds. The complexes proved non-toxic to A2780 cells in short-term MTT assays, with the exception of **Ir-6**, which exerted toxicity in higher concentrations (Figure 6). In the SRB assays, **Ir-6** showed a pronounced, and **Ir-3** a mild, cytostatic activity, while none of the other complexes showed any considerable cytostatic activity (Figure 6, Table 5). **Ir-dimer** was inactive (Figure 6). We assessed whether the cytostatic ability of the complexes was specific for A2780 cell lines by testing them on another ovarian cancer cell line, ID8. On ID8 cells, only **Ir-6** was active, exerting mild cytotoxicity and pronounced cytostatic activity (Figure 6, Table 5). The **Ir-dimer** was inactive (Figure 6). Finally, we wanted to test whether the mild cytotoxic and strong cytostatic activity of the complexes was specific for neoplastic cells or can be elicited on non-transformed cells. For that purpose, we tested the compounds on human primary fibroblasts. None of the complexes, **Ir-1‒Ir-12**, or the **Ir-dimer** exerted cytotoxic or cytostatic activity on fibroblasts (Figure 6).

**Figure 7:**
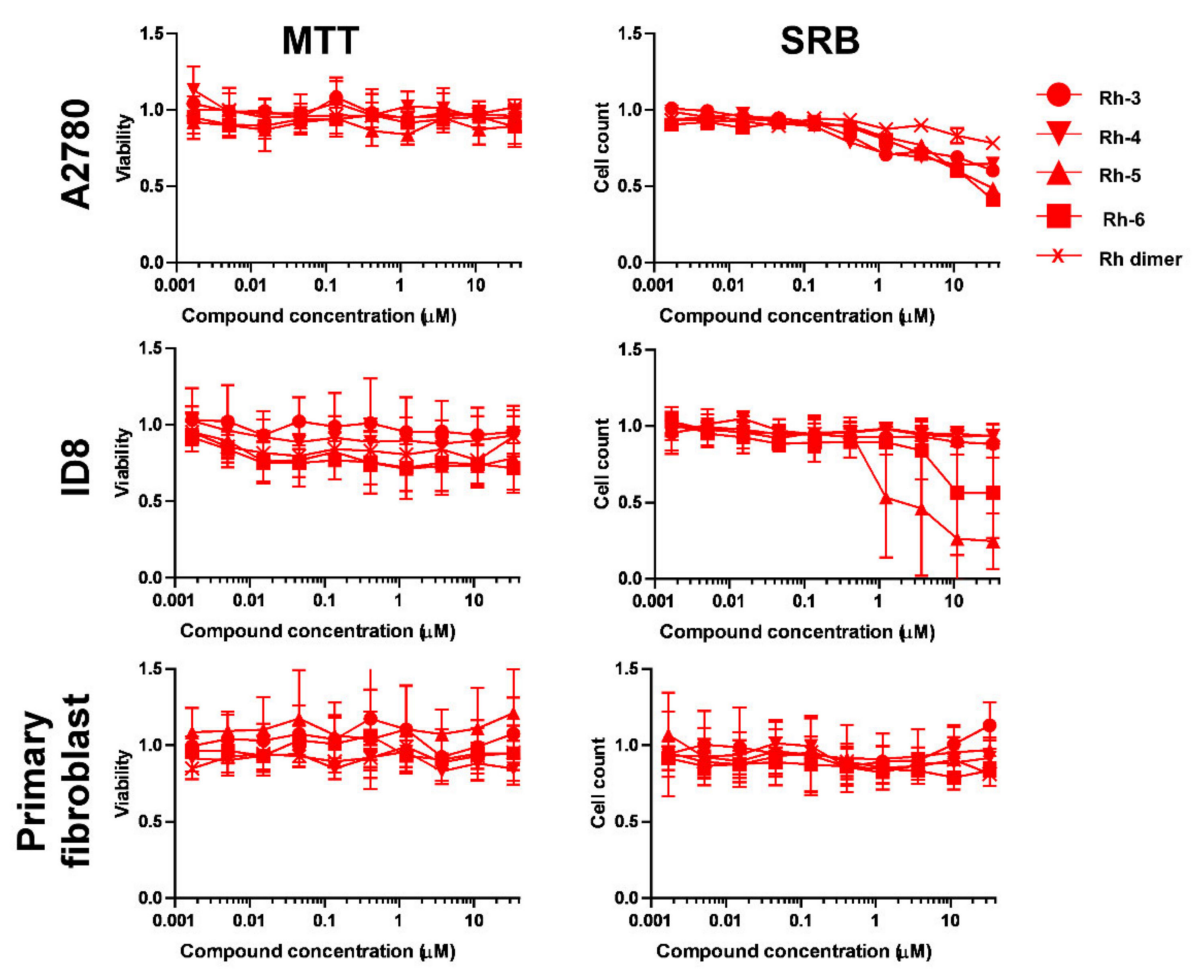
Assessment of rhodium complexes **Rh-3‒Rh-6** for cytotoxic and cytostatic activity. Briefly, 3 × 10^3^ A2780 cells, 2 × 10^3^ ID8 cells, and 4 × 10^3^ primary fibroblasts were plated onto 96-well plates. Cells were treated with the compounds in the concentrations indicated for either 4 h for an MTT assay or for 48 h for an SRB assay. Data are represented as average ± SD, from three biological replicates; individual assays were performed in duplicate. Values were normalized for vehicle-treated cells; absorbance for vehicle-treated cells is equal to 1. A one-way ANOVA, followed by a Kruskal–Wallis test, was performed individually for each compound. The results of the statistical analysis are presented in an Excel spreadsheet at https://figshare.com/s/28d14597d5041f08eb08 (accessed on 6 January 2022). Nonlinear regression was performed on the data.

Finally, we assessed the rhodium-containing compounds (**Rh-3‒Rh-6**), together with the appropriate rhodium dimer precursor (**Rh-dimer**) that was used for the synthesis of the complexes. The complexes proved non-toxic to A2780 cells in short-term MTT assays (Figure 7). In the SRB assays, all complexes had mild cytostatic activity (Figure 7, Table 5). **Rh-dimer** was inactive (Figure 7). We assessed whether the complexes behaved differently on other cell lines by testing them on another ovarian cancer cell line, ID8. On ID8 cells, **Rh-5** and **Rh-6** had mild cytostatic activity (Figure 7, Table 5). **Rh-dimer** was inactive (Figure 7). Finally, we wanted to test whether the mild cytotoxic and cytostatic activity of the complexes was specific for neoplastic cells or could be elicited on non-transformed cells. For that purpose, we tested the compounds on human primary fibroblasts. None of the complexes **Rh-3‒Rh-6** or the **Rh-dimer** exerted cytotoxic or cytostatic activity on fibroblasts (Figure 7).

**Figure 8:**
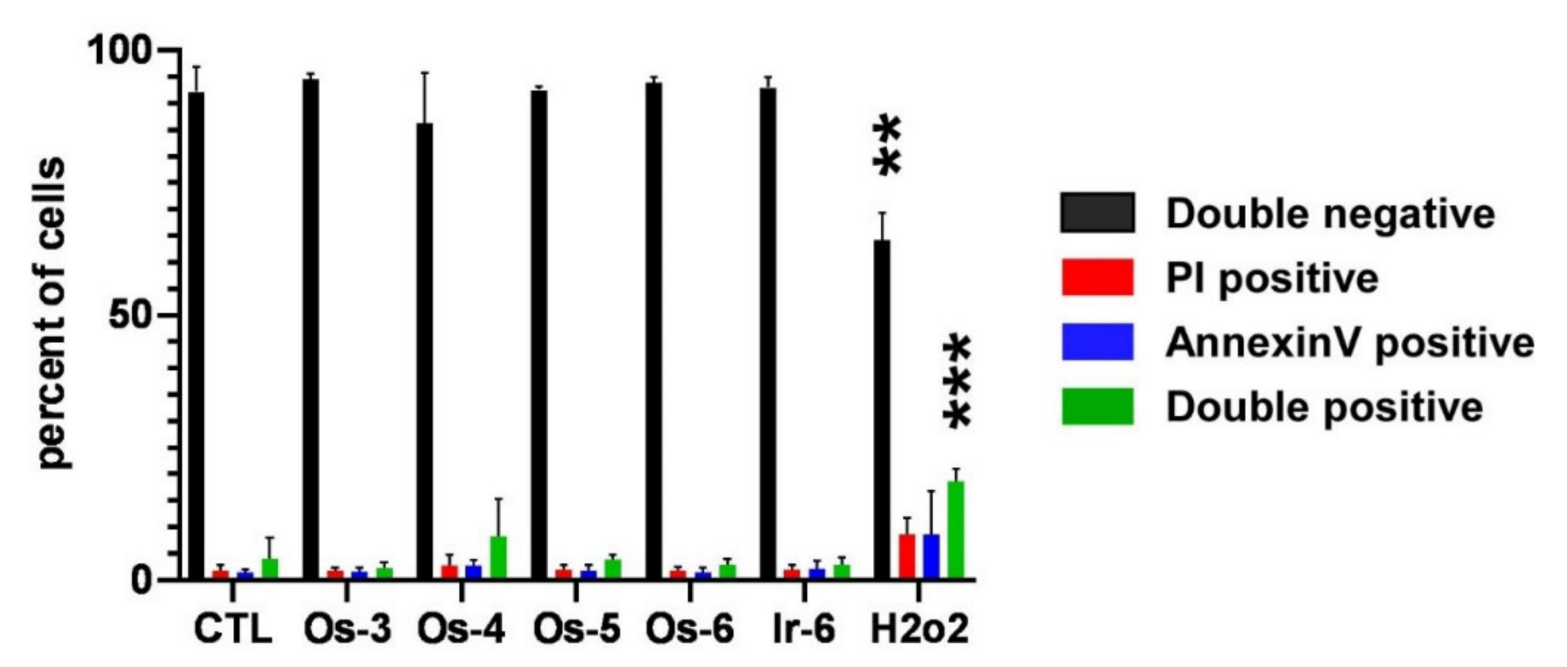
Complexes **Os-3‒Os-6** and **Ir-6** did not induce cell death. Here, 1 × 10^6^ A2780 cells were plated in 6-well plates and were treated with the corresponding complexes (**Os-3** at 2.68 µM, **Os-4** at 3.25 µM, **Os-5** at 2.84 µM, **Os-6** at 0.70 µM, **Ir-6** at 1.63 µM,) and 300 µM hydrogen peroxide for 2 h. Cells were then stained with Annexin V and propidium iodide (PI), then, cells were subjected to flow cytometry as described in the Materials and Methods section. The percentage of cells in the quadrants was plotted. Data are represented as average ± SD, from three biological replicates; individual assays were performed in duplicate. Normality was tested, on all datasets. A two-step transformation was used to achieve normal distribution. Statistical significance was determined using a two-way ANOVA test; all measurement points were compared with each other. ** and *** indicate statistically significant differences between vehicle-treated (control) and treated cells (ruthenium complexes or 300 µM H_2_O_2_) corresponding to the same quadrant (e.g., vehicle-treated double-negative cells vs. **Os-3**-treated double-negative cells) at *p* < 0.01 and *p* < 0.001, respectively.

We selected the **Os-3, Os-4, Os-5, Os-6** and **Ir-6** complexes for further study, based on their strong cytostatic activity on the ovarian cancer cell lines, but there was a lack of activity on primary fibroblasts. As the complexes showed mild activity in short-term toxicity assays (MTT assays), we thus assessed cell death using an Annexin V—propidium iodide double-staining to identify necrotic and apoptotic cells upon treatment with **Os-3**, **Os-4**, **Os-5**, **Os-6** and **Ir-6**. Similar to previous approaches, as propidium iodide or Annexin V positivity is an early feature of cell death [30,43], we treated cells for 2 h and then performed double-staining with Annexin V—propidium iodide. None of the complexes induced cell death, in contrast to 300 µM hydrogen peroxide, which was used as a positive control (Figure 8).

#### 2.2.2. The Bioactive Complexes Are Active on Cell Lines Other than Ovarian Cancer

**Figure 9:**
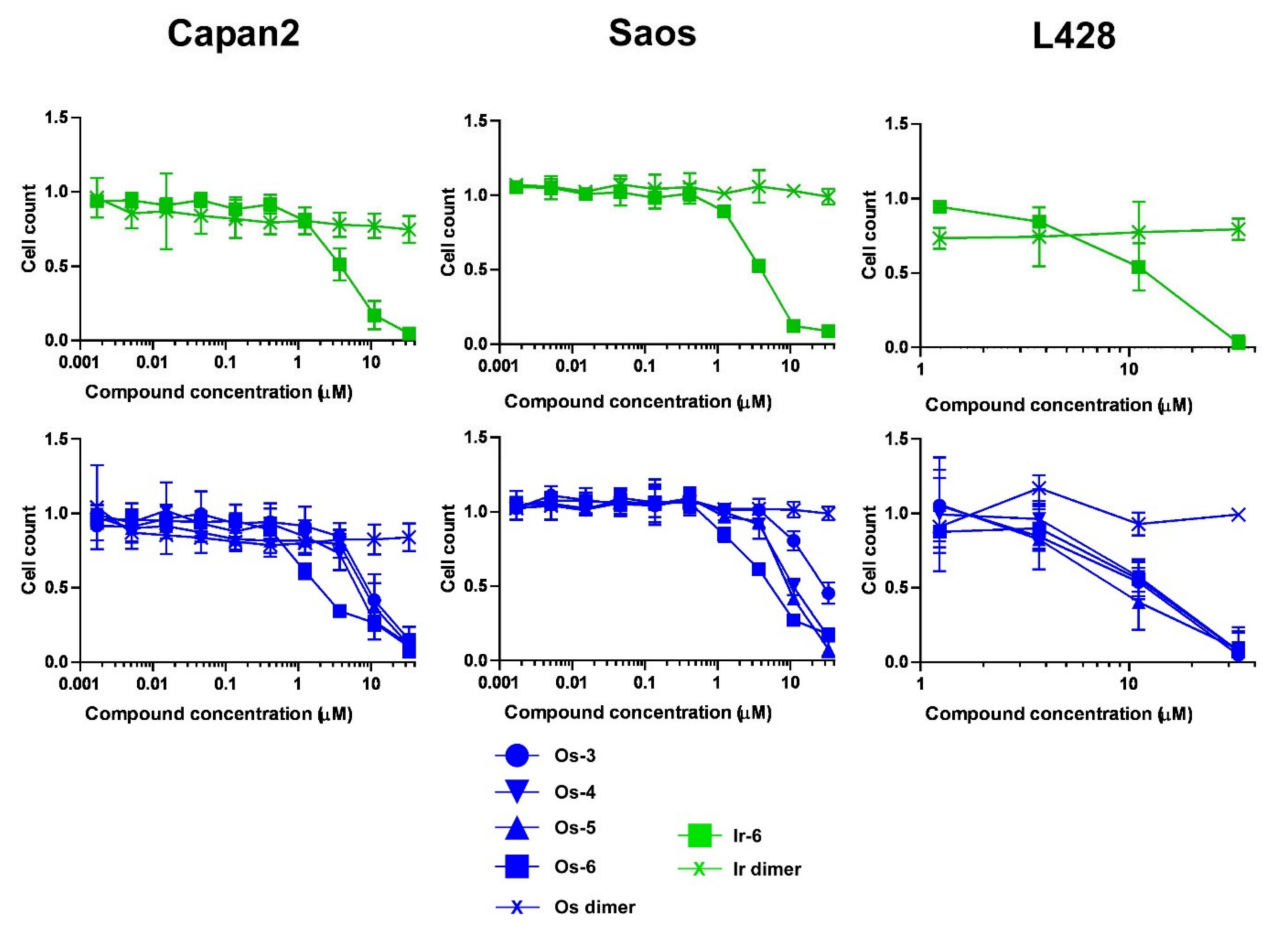
Assessment of selected Os(II) and Ir(III) complexes (**Os-3‒Os-6**, **Os-dimer**, **Ir-6** and **Ir-dimer**) for cytostatic activity. Here, 1.5 × 10^3^ Capan2, 3 × 10^3^ Saos cells and 10^4^ L428 cells were plated onto 96-well plates. Capan2 and Saos cells were treated with the compounds in the concentrations indicated for 48 h for an SRB assay. L428 cells were treated with the compounds in the concentrations indicated for 96 h, then cells were counted using conventional microscopy and a Burker chamber. Data are represented as average ± SD, from three biological replicates; individual assays were performed in duplicate. Values were normalized for vehicle-treated cells; the absorbance for vehicle-treated cells is equal to 1. A one-way ANOVA, followed by a Kruskal–Wallis test, was performed individually for each compound. The results of the statistical analysis are presented in an Excel spreadsheet at https://figshare.com/s/28d14597d5041f08eb08 (accessed on 6 January 2022). Nonlinear regression was performed on the data.

We turned next to assessing whether the cytostatic activity of **Os-3, Os-4, Os-5, Os-6** and **Ir-6** complexes is specific for ovarian cancer cell lines or if they may be active on other cell lines. To that end, we tested the complexes on another carcinoma cell line (Capan2, a pancreatic adenocarcinoma cell line), on a sarcoma cell line (Saos, an osteosarcoma cell line) and on a lymphoma cell line (L428, a Hodgkin’s lymphoma cell line). All five compounds were active on all cell lines in somewhat higher concentrations than on A2780 or ID8 ovarian cancer cells (Figure 9, Table 5).

#### 2.2.3. The Active Complexes Induce Oxidative Stress

**Figure 10:**
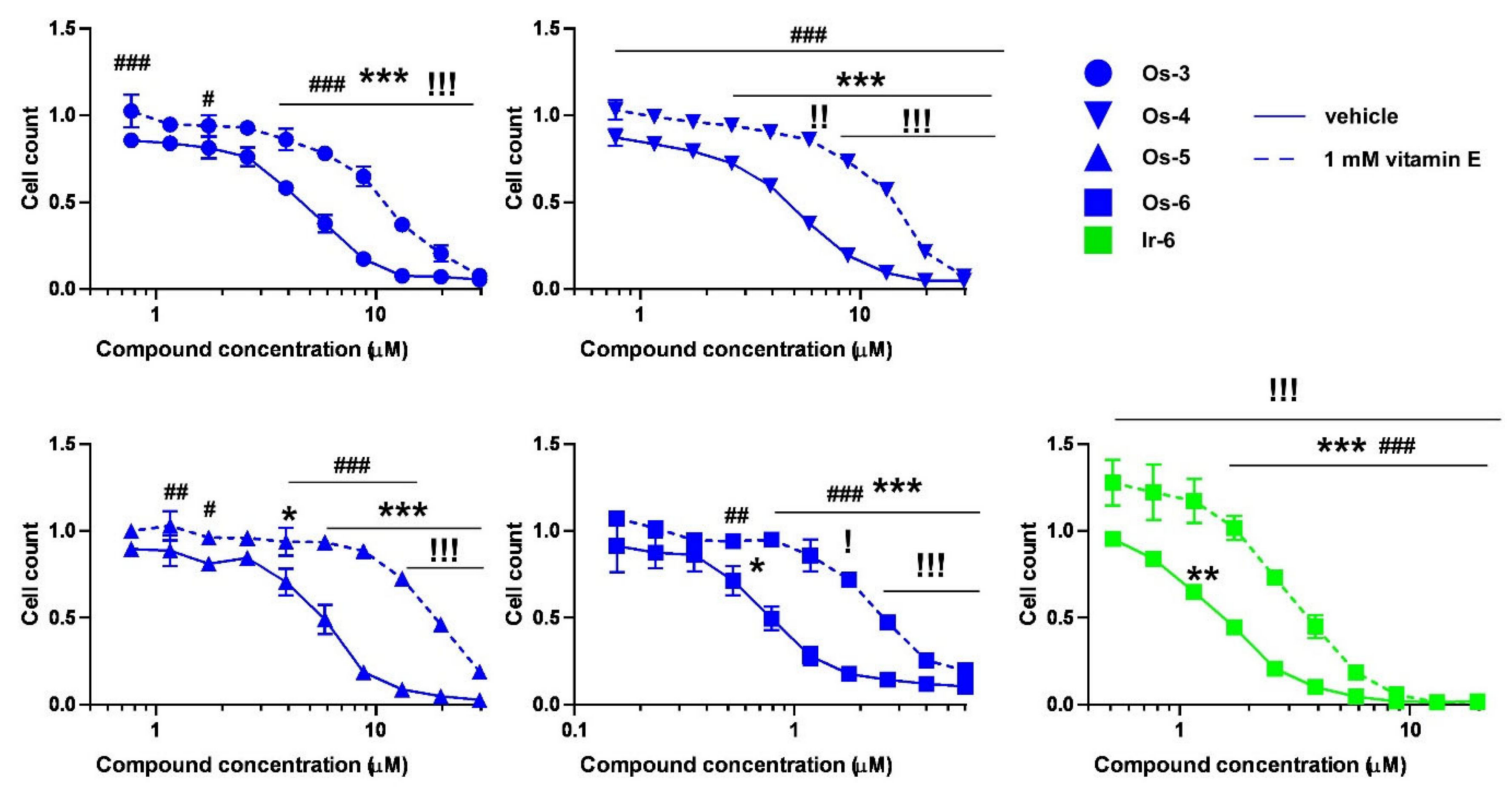
Assessment of selected Os(II) and Ir(III) complexes (**Os-3‒Os-6**, **Ir-6**) for cytostatic activity. Here, 1.5 × 10^3^ A2780 cells were plated onto 96-well plates. Cells were treated with the compounds in the concentrations indicated for 48 h for an SRB assay with or without 1 mM vitamin E. Data are represented as average ± SD, from three biological replicates; individual assays were performed in duplicate. Values were normalized for vehicle-treated cells. Absorbance for vehicle-treated cells is equal to 1. Normality was assessed using the d’Agostino and Pearson test. **Os-3, Os-4, Os-6, Ir-6** data had normal distribution, while in **Os-5**, normality was achieved using the Box-Cox normalization method. Statistical difference was assessed using a two-way ANOVA test; all measurement points were compared with each other. *, ** and *** indicate statistically significant differences between vehicle-treated (control) cells and those treated with a compound but not with vitamin E, at *p* < 0.05 and *p* < 0.001, respectively. #, ##, and ### indicate statistically significant differences between vehicle-treated (no Vitamin E) and the cells treated with a Vitamin E at *p* < 0.05, *p* < 0.01 and *p* < 0.001, respectively. !, !!, and !!! indicate statistically significant differences between vitamin E and vehicle-treated cells (control) and the cells treated with vitamin E and a compound at *p* < 0.05, *p* < 0.01 and *p* < 0.001, respectively.

Several ruthenium complexes were shown to induce oxidative stress in target cell models [23,27,30,31,32] and, according to the literature reports, organoosmium [44,45,46] and organoiridium complexes [47,48] can also induce oxidative stress. To assess the causative role of oxidative stress in cytostasis induced by the compounds we identified in this study, we co-treated cells with the compounds and with vitamin E, a lipophilic antioxidant. The cytostatic properties of **Os-3, Os-4, Os-5, Os-6** and **Ir-6** complexes were potently inhibited by vitamin E; the sigmoid inhibitory curve shifted to the right (Figure 10), suggesting a major role for reactive species production in **Os-3, Os-4, Os-5, Os-6** and **Ir-6**-induced cytostasis.

## 3. Discussion

In this study, we assessed a set of carbohydrate-based half-sandwich-type complexes with various platinum-group metal ions, such as ruthenium(II), osmium(II), iridium(III) and rhodium(III). We identified five compounds with strong long-term cytostatic ability but with little short-term direct toxicity on cancer cells. Most importantly, the complexes were inactive on primary human fibroblasts, suggesting a selectivity toward transformed cancer cells, this being similar to the previously identified set of ruthenium complexes [30]. These data go well with the observation that ruthenium complexes have low toxicity [23,24,25,26] and project similar properties to osmium and iridium complexes.

We showed that the complexes were effective on ovarian cancer cell lines, similar to previously reported instances [30,49,50,51]. Other Ru-based compounds were shown to be active on other carcinomas, such as MDA-MD-231 breast cancer cells [49], colon cancer [50,51,52,53], non-small-cell lung cancer [51] or cervical carcinoma (HeLa) cells [54], suggesting the applicability of the compounds on a wide array of carcinomas. Nevertheless, the question still stood whether the compounds would be active on sarcomas or hematological malignancies that have a different origin than carcinomas. To test that hypothesis, we opted for Saos cells modeling osteosarcoma, and L428 cells modeling Hodgkin’s lymphoma. Most importantly, the active compounds identified in this study were efficient on both cell lines in somewhat higher concentrations than on carcinomas, suggesting an even wider possible applicability of the compounds.

**Figure 11:**
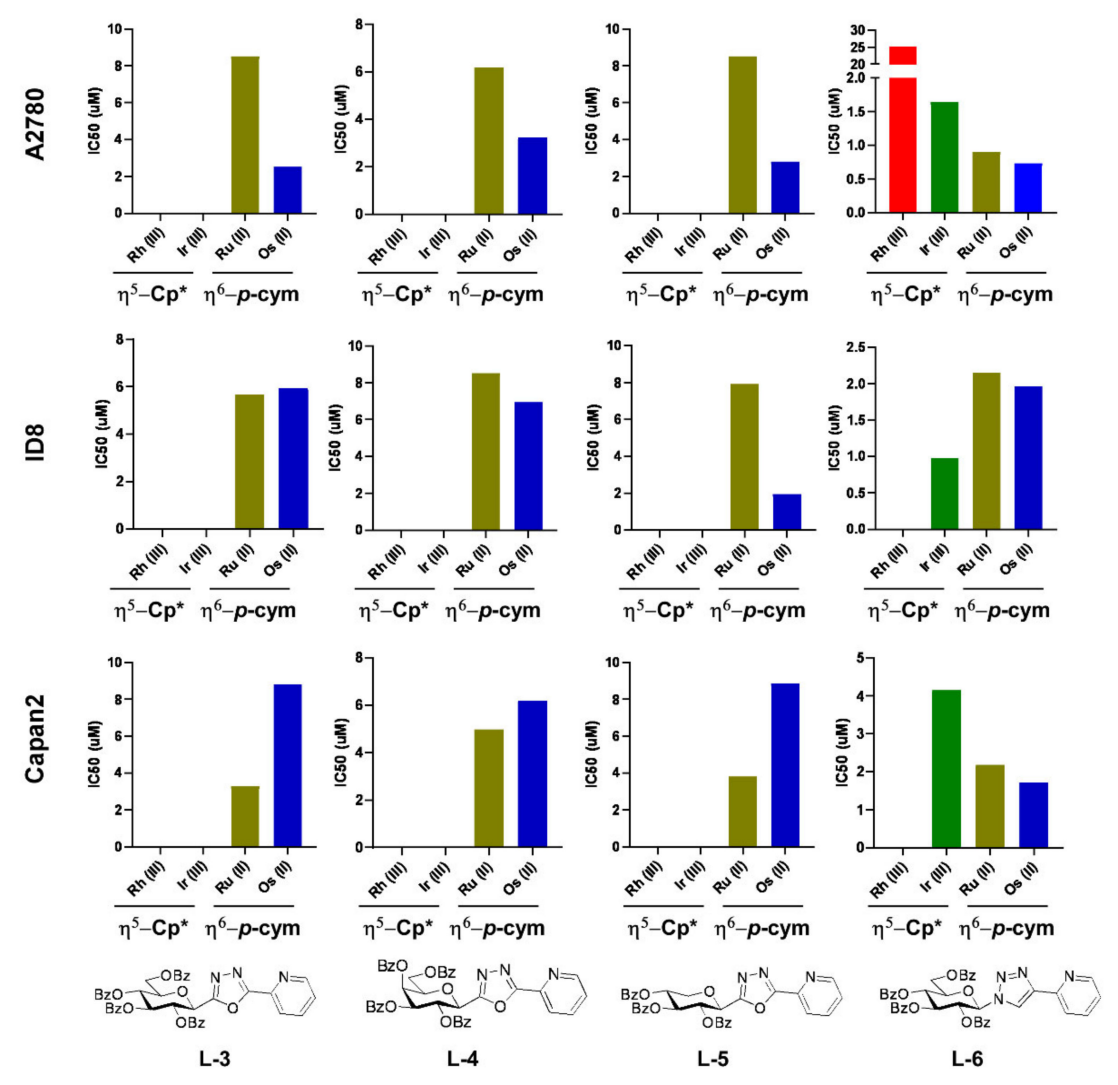
Structure–function relationship between complexes of **L-3‒L-6** ligands. Empty bars represent experiments where the calculation of the IC_50_ values was not possible.

In this study, we applied osmium(II) ions with a *p*-cymene ligand (**Os-3-Os-6**) and rhodium(III) and iridium(III) ions with a pentamethylcyclopentadienyl ligand (**Ir-1-Ir-12, Rh-3-Rh-6**). In a previous study [30], we assessed ruthenium(II) complexes with a *p*-cymene ligand (**Ru-3-Ru-6**). Complexes were tested under the same conditions as the current ones, hence providing comparable data (Table 5). The free sugar-based ligands had no biological activity [30]. Those complexes exerting biological activity contained the ligands **L-3**, **L-4**, **L-5** or **L-6**. Furthermore, the ligands **L-3**, **L-4**, **L-5** and **L-6** in *p*-cymene containing Os(II) and Ru(II) complexes had significantly better cytostatic properties than the same ligands in Cp* containing Rh(III) or Ir(III) complexes (Figure 11). These observations are further underlined by the observations of Böge et al. [55], who assessed carbohydrate-based ruthenium(II), rhodium(III), and iridium(III) complexes and provided evidence that ruthenium complexes were active in anticancer model systems.

Based on these IC_50_ values, it may be hypothesized that the presence of the hexahapto-bound *p*-cymene ligand (Ru, Os), compared to the pentahapto arenyl (Rh, Ir), provides a higher level of activity. This might be due to lower electron density and, thus, less stability of the metal-carbon bonds for the former pair of metal ions [56]. When the appropriate 4*d* and 5*d* metal pairs are compared, our results indicate superior activity of the complexes of the 5*d* metals over the 4*d* counter-pairs. This might be explained by kinetic differences, as it is widely accepted that the half-sandwich-type Os and Ir complexes, in general, exhibit much lower ligand exchange rates than the Ru and Rh analogs [57]. Furthermore, since high(er) oxidation states get stabilized down in a given column for the *d* block metals, identical oxidation states (+2 for Ru and Os; +3 for Rh and Ir) would mean lower stability and, thus, higher/appropriate reactivity for the complexes with the heavier *5d* metal ions over those of their lighter congener pairs.

Based on our results, it can also be concluded that the azole ring of the N,N-chelators applied has an influence on the biological activity of the complexes. First, in the case of **L-6**-based complexes containing a triazole heterocycle, we identified biologically active Ir(III) and Rh(III) complexes (**Rh-6** and **Ir-6**), but the same ions with oxadiazole-containing ligands **L-3**, **L-4** and **L-5** were inactive (Figure 11). Furthermore, for the comparable pairs of the different platinum metal complexes (**Ru-3** vs. **Ru-6**, **Os-3** vs. **Os-6**, **Ir-3** vs. **Ir-6** and **Rh-3** vs. **Rh-6**), superior potencies of those complexes with *N*-glucosidically linked 1,2,3-triazoles (**Ru-6**, **Os-6**, **Ir-6**, **Rh-6**) to their *C*-coupled 1,3,4-oxadiazole counterparts (**Ru-3**, **Os-3**, **Ir-3**, **Rh-3**) were observed in A2780 cells (Figure 11, Table 5). However, changing the constitution of the oxadiazole ring (namely, the replacement of the 1,3,4-oxadiazole moiety by a 1,2,4-oxadiazole ring) surprisingly abolished the inhibitory properties of the molecules (e.g., **Ru-3** vs. **Ru-1**, **Ir-3** vs. **Ir-1**, Table 5).

We already pointed out that the sufficiently apolar nature of the compounds (LogD > +2) is a necessary feature for their effectivity [30]. In good agreement with this finding, **Ir-6** with the *O*-perbenzoylated glucosyl-1,2,3-triazole ligand displayed cytostatic activity, while **Ir-11** and **Ir-12** with *O*-peracetylated and *O*-deprotected forms of the same ligand (both having negative logD values) were biologically inactive. These are backed by an observation from Hamala and colleagues [49], demonstrating that in 1,4-bis(β-D-glycopyranosyl)tetrazene containing half-sandwich Ru(II) complexes, the protection of the hydroxyl groups of the sugar moiety by longer chain esters enhanced the biological activity. More precisely, the increasing length of the acyl chain improved the inhibitory efficacy (acetyl < propionyl < butyryl). Furthermore, Hanif and colleagues [51] showed with RAPTA-analogs that increasing the apolar character of the arene moiety by replacing phenyl with biphenyl enhanced the inhibitory activity of the molecules.

As we pointed out in an earlier work [30], the inhibitory curve of ruthenium complexes displays cooperative binding, as highlighted by a high Hill coefficient or Hill slope. A higher Hill coefficient suggests that the binding of a molecule to its target facilitates the binding of the next molecule [58]. There is an example for that in the literature, in the study of Hanif et al. [51] in which RAPTA-analogs were assessed; one compound had a steep inhibitory curve suggestive of cooperative binding. We extended these observations by assessing the compounds identified in this study and in a previous study [30] by comparing the apolar character (logD value), the cooperativity of the compounds (Hill coefficient) and the inhibitory properties (IC_50_ value) (Table 5). We found that good inhibitors with IC_50_ values in the low micromolar range have logD values above 2 and a Hill coefficient of close to 2 or above. In other words, cooperativity and an apolar nature largely improve the inhibitory character of the molecules. The polar character of the molecules (for example, deprotection of the carbohydrate moiety) completely abolished the inhibitory activity.

Platinum-group metal complexes can generate reactive oxygen species (ROS [23,27,30,31,32,59,60]. Given the fact that ROS production confers cytostatic properties in numerous carcinomas [23,27,31,32,61,62,63,64], we assessed whether ROS production could be a cause for cytostasis upon applying the active compounds we identified. In good agreement with that hypothesis, vitamin E protected the A2780 cells against cytostasis induced by osmium or iridium complexes. This suggests that these complexes bring about ROS production, leading to cytostasis, which is similar to another set of ruthenium complexes [30]. It is also important to note that vitamin E has a long, apolar phytyl chain. When the phytyl chain is removed, the protective capacity is lost, further underscoring the importance of the apolar nature of the complexes and making it likely that the complexes target the apolar compartments in a cell.

## 4. Conclusions

The syntheses of novel Ru, Os, Ir and Rh half-sandwich complexes with *p*-cymene vs. cyclopentadienyl ligands and bidentate *C*- and *N*-glycopyranosyl azole-type N,N-chelators was accomplished. The antiproliferative assay of these complexes and their glycosyl heterocyclic ligands showed no activity for the uncomplexed ligands, but in the case of several complexes, low micromolar efficiencies were observed for tumorous cell lines, such as ovarian cancer, pancreatic adenocarcinoma, osteosarcoma, and Hodgkin’s lymphoma cells. The most important structural features for significant biological activity were identified as the presence of: a) an *N*-glycosidically linked 1,2,3-triazole (vs. *C*-glycosyl oxadiazoles); b) strongly hydrophobic *O*-protection of the monosaccharide unit (OBz vs. OAc or OH); c) Ru(II) or Os(II) central ions with a hexahapto arene ligand (namely, *p*-cymene) rather than Ir(III) or Rh(III) with pentahapto arenyl moiety (namely, Cp*).

We propose the use of these newly identified compounds in neoplasias where other metal-based cytostatic agents are in use but therapeutic options are limited, as in ovarian cancer [65] or pancreatic adenocarcinoma [66].

## 5. Materials and Methods

### 5.1. Syntheses

#### 5.1.1. General Methods

Optical rotations were determined by a Jasco P-2000 polarimeter (Jasco, Easton, MD, USA) at r.t., and the data correspond to the average of three parallel measurements. NMR spectra were recorded with DRX360 (360/90 MHz for ^1^H/^13^C) or DRX400 (400/100 MHz for ^1^H/^13^C) spectrometers (Bruker, Karlsruhe, Germany). Chemical shifts were referenced to Me_4_Si (^1^H-NMR) or to the residual solvent signals (^13^C-NMR). MS data were measured by a Bruker maXis II (ESI-HRMS) instrument. For TLC analysis, DC Kieselgel 60 F254 plates (Sigma-Aldrich, St. Louis, MO, USA) was used, checking of the spots was performed under UV light and the spots were developed by gentle heating. Column chromatographic purifications were carried out by applying Kieselgel 60 silica gel (Molar Chemicals, Halásztelek, Hungary, particle size 0.063–0.2 mm). Anhydrous solvents were freshly prepared following standard distillation procedures: anhydrous CHCl_3_ and toluene were produced by distillation from P_4_O_10_, then stored over 4 Å molecular sieves and sodium wires, respectively; MeOH was dried by distillation over Mg turnings and iodine; 1,4-dioxane was distilled from sodium benzophenone ketyl and the anhydrous solvent was then stored over sodium wires. Picolinic acid (Fluka), dichloro(η^6^-*p*-cymene)ruthenium(II) dimer (**Ru-dimer**, Strem Chemicals, Newburyport, MA, USA), dichloro(pentamethylcyclopentadienyl)iridium(III) dimer (**Ir-dimer**, Acros Organics), the dichloro(pentamethylcyclopentadienyl)rhodium(III) dimer (**Rh-dimer**, Alfa Aesar) and TlPF_6_ (Strem Chemicals) were purchased from the indicated suppliers. Dichloro(η^6^-*p*-cymene)osmium(II) dimer (**Os-dimer**) [37] and *C*-(2,3,4,6-tetra-*O*-benzoyl-β-D-glucopyranosyl)formamidoxime (**1**) [36] were synthesized based on the cited literature methods. 2-(2′,3′,4′,6′-Tetra-*O*-benzoyl-β-D-glucopyranosyl)-5-(pyridin-2-yl)-1,3,4-oxadiazole (**L-3**), 2-(2′,3′,4′,6′-tetra-*O*-benzoyl-β-D-galactopyranosyl)-5-(pyridin-2-yl)-1,3,4-oxadiazole (**L-4**), 2-(2′,3′,4′-tri-*O*-benzoyl-β-D-xylopyranosyl)-5-(pyridin-2-yl)-1,3,4-oxadiazole (**L-5**), 1-(2′,3′,4′,6′-tetra-*O*-benzoyl-β-D-glucopyranosyl)-4-(pyridin-2-yl)-1,2,3-triazole (**L-6**), 2-(2′,3′,4′,6′-tetra-*O*-acetyl-β-D-galactopyranosyl)-5-(pyridin-2-yl)-1,3,4-oxadiazole (**L-7**), 2-(β-D-glucopyranosyl)-5-(pyridin-2-yl)-1,3,4-oxadiazole (**L-8**), 2-(β-D-galactopyranosyl)-5-(pyridin-2-yl)-1,3,4-oxadiazole (**L-9**), 2-(β-D-xylopyranosyl)-5-(pyridin-2-yl)-1,3,4-oxadiazole (**L-10**), 1-(2′,3′,4′,6′-tetra-*O*-acetyl-β-D-glucopyranosyl)-4-(pyridin-2-yl)-1,2,3-triazole (**L-11**), and 1-(β-D-glucopyranosyl)-4-(pyridin-2-yl)-1,2,3-triazole (**L-12**) were prepared according to our earlier reported procedures [30].

#### 5.1.2. General Method I for the Synthesis of the [(η^6^-*p*-cym)M^II^(N-N))Cl]PF_6_ (M = Ru, Os) and [(η^5^-Cp*)M^III^(N-N))Cl]PF_6_ (M = Ir, Rh)-Type Complexes Containing *O*-Peracylated Glycosyl Azole Ligands

The corresponding dimer ([(η^6^-*p*-cym)M^II^Cl_2_]_2_ (M = Ru, Os), **Ru-dimer**, **Os-dimer** or [(η^5^-Cp*)M^III^Cl_2_]_2_ (M = Ir, Rh), **Ir-dimer**, **Rh-dimer**) was dissolved in CH_2_Cl_2_ (10 mg/1 mL) and the appropriate *O*-peracylated glycosyl azole (2 eq.) and TlPF_6_ (2 eq.) were added. Under stirring, the same volume of methanol was added to the mixture in order to precipitate the TlCl. The heterogenous mixture was then stirred at rt for an hour, and the disappearance of the starting dimer was monitored by TLC (9:1 CHCl_3_-MeOH). After completion of the reaction, the TlCl was filtered off and the residual solution was evaporated under diminished pressure. The crude complex was purified by column chromatography or precipitation from its solution in CHCl_3_ by the addition of Et_2_O.

#### 5.1.3. General Method II for the Formation of the [(η^6^-*p*-cym)Ru^II^(N-N))Cl]PF_6_ and [(η^5^-Cp*)Ir^III^(N-N))Cl]PF_6_ Type Complexes Containing Unprotected Glycosyl Azole Ligands

The corresponding dimer ([(η^6^-*p*-cym)Ru^II^Cl_2_]_2_, **Ru-dimer** or [(η^5^-Cp*)Ir^III^Cl_2_]_2_, **Ir-dimer**) was dissolved in CH_2_Cl_2_ (10 mg/1 mL) and the appropriate unprotected glycosyl azole (2 eq.) and TlPF_6_ (2 eq.) were added to make a suspension. Under stirring the same volume of methanol was added to this mixture in order to help dissolution of the glycosyl azole and precipitation of the TlCl. The reaction mixture was then stirred at rt for an hour and the transformation of the starting dimer was monitored by TLC (9:1 CHCl_3_-MeOH). After completion of the reaction, the TlCl was filtered off and the residual solution was evaporated under reduced pressure. The crude complex was purified by recrystallization from *i-*PrOH or from a solvent mixture of *i*-PrOH and MeOH.

#### 5.1.4. *O*-Picolinoyl-*C*-(2,3,4,6-tetra-*O*-benzoyl-β-D-glucopyranosyl)formamidoxime (**2**)

2-Picolinic acid (116 mg, 0.94 mmol, 3 eq.) was boiled in thionyl chloride (2 mL) for 4 h. The thionyl chloride was then removed under reduced pressure and dry toluene (2 × 5 mL) was then evaporated from the residual syrup. The obtained picolinoyl chloride was dissolved in dry 1,4-dioxane (5 mL) and *C*-(2,3,4,6-tetra-*O*-benzoyl-β-D-glucopyranosyl)formamidoxime (**1**, 200 mg, 0.31 mmol) was added. The reaction mixture was stirred under argon until the TLC (4:1 EtOAc-hexane) showed the total consumption of **1** (1 h). The insoluble materials were filtered off, washed with CH_2_Cl_2_ and the filtrate was evaporated under reduced pressure. The residue was redissolved in CH_2_Cl_2_ (25 mL), washed with saturated NaHCO_3_ solution (25 mL) and the organic phase was dried over MgSO_4_, then filtered, and the solvent was removed. The crude product was purified by column chromatography (4:1 EtOAc-hexane), yielding 156 mg white amorphous solid (76%). R_f_ = 0.27 (4:1 EtOAc-hexane); [α]_D_ = −40 (c 0.20, CHCl_3_). ^1^H NMR (400 MHz, CDCl_3_) δ (ppm): 8.62 (1H, ddd, *J* = 4.8, 1.8, 0.9 Hz, Py-H-6), 8.05–7.75, 7.56–7.23 (23H, m, Ar, Py-H-3–Py-H-5), 5.97 (1H, pt, *J* = 9.6, 9.6 Hz, H-2′ or H-3′ or H-4′), 5.77 (1H, pt, *J* = 9.8, 9.2 Hz, H-2′ or H-3′ or H-4′), 5.76 (1H, pt, *J* = 9.8, 9.7 Hz, H-2′ or H-3′ or H-4′), 5.80–5.74 (1H, m, NH), 4.65 (1H, dd, *J* = 12.4, 2.7 Hz, H-6′a), 4.60 (1H, d, *J* = 9.9 Hz, H-1′), 4.52 (1H, dd, *J* = 12.4, 5.4 Hz, H-6′b), 4.27 (1H, ddd, *J* = 10.0, 5.4, 2.7 Hz, H-5′), 2.47 (1H, m, NH); ^13^C NMR (100 MHz, CDCl_3_) δ (ppm): 166.3, 165.8, 165.7, 165.3 (4 × C=O), 162.1, 154.9 (N=C, O=C), 149.3 (Py-C-6), 147.4 (Py-C-2), 137.3 (Py-C-4), 133.7, 133.4, 133.3 (2), 130.1, 130.0, 129.9, 129.8, 129.5, 128.9, 128.8, 128.7, 128.6, 128.5, 128.4 (2) (Ar), 127.1 (Py-C-5), 125.6 (Py-C-3), 76.8, 76.0, 73.9, 70.0, 69.3 (C-1′–C-5′), 63.1 (C-6′). ESI-HRMS positive mode (*m/z*): calcd for C_41_H_34_N_3_O_11_^+^ [M+H]^+^ 744.2188; C_41_H_33_N_3_O_11_Na^+^ [M+Na]^+^ 766.2007. Found: [M+H]^+^ 744.2188; [M+Na]^+^ 766.2003.

#### 5.1.5. 3-(2′,3′,4′,6′-Tetra-*O*-benzoyl-β-D-glucopyranosyl)-5-(pyridin-2-yl)-1,2,4-oxadiazole (**L-1**)

The *O*-acylated amidoxime **2** (450 mg, 0.51 mmol) was suspended in dry toluene (10 mL) and 1M solution of Bu_4_NF in THF (0.06 mL, 0.06 mmol, 0.12 eq.) was added. The reaction mixture was heated under reflux until the TLC showed total consumption of the starting material **2** (1 h). The solvent was removed under reduced pressure and the residue was purified by column chromatography (1:1 EtOAc-hexane) yielding 376 mg white solid (86%). R_f_ = 0.31 (1:1 EtOAc-hexane). [α]_D_ = −48 (c 0.20 CHCl_3_). ^1^H NMR (400 MHz, CDCl_3_) δ (ppm): 8.78 (1H, d, *J* = 4.8 Hz, Py-H-6), 8.19–7.82, 7.54–7.28 (23H, m, Ar, Py-H-3–Py-H-5), 6.13 (1H, pt, *J* = 9.2, 9.2 Hz, H-2′ or H-3′ or H-4′), 6.08 (1H, pt, *J* = 9.2, 8.9 Hz, H-2′ or H-3′ or H-4′), 5.88 (1H, pt, *J* = 9.2, 8.9 Hz, H-2′ or H-3′ or H-4′), 5.23 (1H, d, *J* = 9.2 Hz, H-1′), 4.68 (1H, m, H-6′a), 4.57 (1H, dd, *J* = 12.5, 5.4 Hz, H-6′b), 4.40 (1H, m, H-5′); ^13^C NMR (100 MHz, CDCl_3_) δ (ppm): 167.2 (2), 166.3, 165.9, 165.3, 164.8 (4 × C=O, OD-C-3, OD-C-5), 150.7 (Py-C-6), 143.3 (Py-C-2), 137.4 (Py-C-4), 133.6, 133.5, 133.4, 133.2, 130.0, 129.9 (3), 129.6, 128.8, 128.7 (2), 128.5, 128.4 (3) (Ar), 127.0 (Py-C-5), 124.6 (Py-C-3), 77.1, 74.3, 72.6, 70.7, 69.4 (C-1′–C-5′), 63.4 (C-6′). ESI-HRMS positive mode (*m/z*): calcd for C_41_H_31_N_3_O_10_Na^+^ [M+Na]^+^ 748.1902. Found: [M+Na]^+^ 748.1901.

#### 5.1.6. 3-(β-D-Glucopyranosyl)-5-(pyridin-2-yl)-1,2,4-oxadiazole (**L-2**)

Oxadiazole **L-1** (365 mg, 0.503 mmol) was dissolved in a 1:1 mixture of dry MeOH and dry CHCl_3_ (10 mL) and a few drops of ~1 M solution of NaOMe in MeOH was added to reach pH = 8–9. The reaction mixture was then kept at rt until the TLC (9:1 CHCl_3_-MeOH) indicated complete transformation of **L-1** (4 h). The neutralization of the solution was then carried out by the addition of a cation exchange resin (Amberlyst 15, H^+^ form). The resin was filtered off and the solvents were removed under diminished pressure. The residual syrup was purified by column chromatography (9:1 CHCl_3_-MeOH), resulting in 110 mg of white amorphous solid (71%). R_f_ = 0.23 (9:1 CHCl_3_-MeOH); [α]_D_ = +8 (c 0.20, MeOH). ^1^H NMR (400 MHz, CD_3_OD) δ (ppm): 8.78 (1H, ddd, *J* = 4.8, 1.7, 0.8 Hz, Py-H-6), 8.32 (1H, ddd, *J* = 7.8, 1.1, 0.8 Hz, Py-H-3), 8.10 (1H, dt, *J* = 7.8, 1.7 Hz, Py-H-4), 7.69 (1H, ddd, *J* = 7.8, 4.8, 1.1 Hz, Py-H-5), 4.55 (1H, d, *J* = 9.8 Hz, H-1′), 3.90 (1H, dd, *J* = 12.2, 1.8 Hz, H-6′a), 3.83 (1H, pt, *J* = 9.0, 9.0 Hz, H-2′ or H-3′ or H-4′), 3.70 (1H, dd, *J* = 12.2, 5.1 Hz, H-6′b), 3.56–3.44 (3H, m, H-2′ and/or H-3′ and/or H-4′, H-5′); ^13^C NMR (100 MHz, CD_3_OD) δ (ppm): 176.0, 170.3 (OD-C-3, OD-C-5), 151.6 (Py-C-6), 144.3 (Py-C_-_2), 139.5 (Py-C-4), 128.7 (Py-C-5), 125.6 (Py-C-3), 82.8, 79.2, 75.0, 73.5, 71.4 (C-1′–C-5′), 62.9 (C-6′). ESI-HRMS positive mode (*m/z*): calcd for C_13_H_15_N_3_O_6_Na^+^ [M+Na]^+^ 332.0853; C_26_H_30_N_6_O_12_Na^+^ [2M+Na]^+^ 641.1814. Found: [M+Na]^+^ 332.0852; [2M+Na]^+^ 641.1813.

#### 5.1.7. Complex **Ru-1**

Complex **Ru-1** was prepared from complex **Ru-dimer** (10.0 mg, 0.0163 mmol), compound **L-1** (23.7 mg, 0.0327 mmol, 2.0 eq.) and TlPF_6_ (11.4 mg, 0.0326 mmol) according to general method I. After filtration and removal of the solvent the residue was dissolved in CHCl_3_ (3 mL) and Et_2_O (6 mL) was added. The precipitated product was filtered off, then washed with CHCl_3_ (1 mL) to give 22.1 mg (59%) orange powder. R_f_: 0.21 (9:1 CHCl_3_-MeOH). Diastereomeric ratio: 4:1. ^1^H NMR (360 MHz, acetone-d_6_) δ (ppm): 9.80 (d, *J* = 5.6 Hz, major Py-H-6), 9.72 (d, *J* = 5.6 Hz, minor Py-H-6), 8.61–7.31 (m, minor and major Ar, Py-H-3–Py-H-5), 6.49–6.14 (m, minor and major H-2′, H-3′, H-4′, 4 × *p*-cym-CH_Ar_, minor H-1′), 5.95 (d, *J* = 10.3 Hz, major H-1′), 5.34 (ddd, *J* = 9.6, 4.5, 2.4 Hz, major H-5′), 5.11–5.04 (m, minor H-5), 5.06 (dd, *J* = 13.0, 2.4 Hz major H-6′a), 4.89 (dd, *J* = 12.8, 2.9 Hz minor H-6′a), 4.84–4.77 (m, minor H-6′b), 4.79 (dd, *J* = 13.0, 4.5 Hz, major H-6′b), 2.98–2.81 (m, minor and major *i*-Pr-CH), 2.37 (s, major C_6_H_4_-C*H_3_*), 2.35 (s, minor C_6_H_4_-C*H_3_*), 1.21, 1.16 (2 d, *J* = 6.9 Hz in each, minor and major 2 × *i*-Pr-C*H*_3_); ^13^C NMR (90 MHz, acetone-d_6_) δ (ppm) (only major isomer): 176.6, 166.7, 166.4 (2), 165.8, 165.0 (C=O, OD-C-3, OD-C-5), 158.3 (Py-C-6), 142.0 (Py-C-4), 141.0 (Py-C-2), 134.7, 134.6, 134.5, 132.5, 130.6–129.4 (Ar, Py-C-5), 128.6 (Py-C-3), 108.6, 105.5 (*p*-cym-C_qAr_), 86.0, 83.5, 83.0, 82.3 (*p*-cym-CH_Ar_), 77.8, 74.9, 71.8, 70.3, 69.7 (C-1′–C-5′), 64.0 (C-6′), 32.1 (*i*-Pr-*C*H), 22.7, 22.1 (*i*-Pr-*C*H_3_), 19.3 (C_6_H_4_-*C*H_3_). ESI-HRMS positive mode (*m/z*): calcd for C_51_H_45_ClN_3_O_10_Ru^+^ [M-PF_6_]^+^ 996.1843. Found: [M-PF_6_]^+^ 996.1841.

#### 5.1.8. Complex **Ru-2**

Complex **Ru-2** was prepared from complex **Ru-dimer** (25 mg, 0.0408 mmol), compound **L-2** (25.3 mg, 0.0818 mmol, 2 eq.) and TlPF_6_ (28.5 mg, 0.0816 mmol) according to general method II. The crude product was triturated with *i*-PrOH to give 33.8 mg (57%) orange powder. Diastereomeric ratio: 7:4. ^1^H NMR (400 MHz, CD_3_OD) δ (ppm): 9.59 (d, *J* = 5.5 Hz, minor Py-H-6), 9.57 (d, *J* = 5.5 Hz, major Py-H-6), 8.54 (d, *J* = 7.8 Hz minor and major Py-H-3), 8.42 (t, *J* = 7.9 Hz, minor and major Py-H-4), 8.04 (m, minor and major Py-H-5), 6.51–6.48 (m, minor and major *p*-cym-CH_Ar_), 6.23 (d, *J* = 6.2 Hz, minor *p*-cym-CH_Ar_), 6.19–6.14 (m, 2 × major *p*-cym-CH_Ar_), 6.02, 6.00 (2 d, *J* = 6.6 Hz in each, 2 × minor *p*-cym-CH_Ar_), 5.93 (d, *J* = 6.2 Hz, major *p*-cym-CH_Ar_), 4.91 (d, *J* = 10.3 Hz, major H-1′), 4.67 (d, *J* = 10.0 Hz, minor H-1′), 4.19–3.37 (m, minor and major H-2′–H-6′), 2.82–2.71 (m, minor and major *i*-Pr-CH), 2.28 (s, major C_6_H_4_-C*H_3_*), 2.27 (s, minor C_6_H_4_-C*H_3_*), 1.18–1.11 (m, minor and major 2 × *i*-Pr-C*H_3_*); ^13^C NMR (90 MHz, CD_3_OD) δ (ppm): 176.4, 168.2 (major OD-C-3, OD-C-5), 176.3, 167.8 (minor OD-C-3, OD-C-5), 158.5 (minor Py-C-6), 158.3 (major Py-C-6), 142.2 (major Py-C-4), 142.1 (minor Py-C-4), 141.6 (minor Py-C-2), 141.4 (major Py-C-2), 132.6 (major Py-C-5), 132.5 (minor Py-C-5), 128.4 (major Py-C-3), 128.3 (minor Py-C-3), 107.9, 103.0 (minor and major *p*-cym-C_qAr_), 86.6, 85.4, 82.8, 82.1 (minor and major *p*-cym-CH_Ar_), 83.7, 78.8, 74.5, 71.9, 71.5 (minor C-1′–C-5′), 83.6, 79.5, 74.9, 72.4, 70.7 (major C-1′–C-5′), 63.3 (minor C-6′), 61.7 (major C-6′), 32.5 (minor *i*-Pr-*C*H), 32.4 (major *i*-Pr-*C*H), 22.7, 22.3 (major *i*-Pr-*C*H_3_), 22.6, 22.5 (minor *i*-Pr-*C*H_3_), 19.1 (minor C_6_H_4_-*C*H_3_), 19.0 (major C_6_H_4_-*C*H_3_). ESI-HRMS positive mode (*m/z*): calcd for C_23_H_29_ClN_3_O_6_Ru^+^ [M-PF_6_]^+^ 580.0786. Found: [M-PF_6_]^+^ 580.0781.

#### 5.1.9. Complex **Os-3**

Complex **Os-3** was prepared from complex **Os-dimer** (10.0 mg, 0.0126 mmol), compound **L-3** (18.4 mg, 0.0254 mmol, 2.0 eq.) and TlPF_6_ (8.8 mg, 0.0252 mmol) according to general method I. After filtration and removal of the solvent, the residue was dissolved in CHCl_3_ (3 mL) and Et_2_O (12 mL) was added. The precipitated product was filtered off, then washed with a solvent mixture of CHCl_3_-Et_2_O = 1:2 (1 mL) to give 22.1 mg (71%) orange powder. R_f_: 0.44 (95:5 CHCl_3_-MeOH). Diastereomeric ratio: 7:6. ^1^H NMR (400 MHz, CDCl_3_) δ (ppm): 9.45 (d, *J* = 5.6 Hz, major Py-H-6), 9.18 (d, *J* = 5.6 Hz, minor Py-H-6), 8.18–7.28 (m, minor and major Ar, Py-H-3–Py-H-5), 6.21–6.05, 5.90–5.84, 5.77–5.72 (m, minor and major H-2′, H-3′, H-4′, 4 × major *p*-cym-CH_Ar_, 2 × minor *p*-cym-CH_Ar_), 5.57, 5.51 (2 d, *J* = 5.7 Hz in each, 2 × minor *p*-cym-CH_Ar_), 5.46 (d, *J* = 9.8 Hz, minor H-1′), 5.35 (d, *J* = 9.9 Hz, major H-1′), 4.73, 4.72 (2 dd, *J* = 12.6, 2.6 Hz in each, minor and major H-6′a), 4.56, 4.55 (2 dd, *J* = 12.6, 5.6 Hz in each, minor and major H-6′b), 4.48, 4.45 (2 ddd, *J* = 9.9, 5.6, 2.6 Hz in each, minor and major H-5′), 2.63 (hept, *J* = 6.9 Hz, minor *i*-Pr-CH), 2.57 (hept, *J* = 6.9 Hz, major *i*-Pr-CH), 2.09 (s, major C_6_H_4_-C*H_3_*), 2.01 (s, minor C_6_H_4_-C*H_3_*), 1.14, 1.10, 1.05, 1.04 (4 d, *J* = 6.9 Hz in each, minor and major 2 × *i*-Pr-C*H*_3_); ^13^C NMR (100 MHz, CDCl_3_) δ (ppm): 169.1, 168.8, 166.3, 165.8, 165.7, 165.3, 165.2 (2), 164.9, 164.8 (minor and major C=O, OD-C-2, OD-C-5), 158.3 (minor Py-C-6), 156.5 (major Py-C-6), 140.3 (major Py-C-4), 140.1 (minor Py-C-2), 140.1 (minor Py-C-4), 139.0 (major Py-C-2), 134.6, 134.0 (2), 133.8, 133.7, 133.5 (2), 133.4, 131.8–127.7 (minor and major Ar, Py-C-5), 125.3 (2) (minor and major Py-C-3), 98.2, 94.9 (major *p*-cym-C_qAr_), 95.5, 93.7 (minor *p*-cym-C_qAr_), 81.0, 77.8, 77.7, 77.5, 77.4, 76.7, 75.4, 74.9, 74.8, 74.4, 73.8, 72.8, 71.8, 71.4 (2), 69.7, 68.8, 68.7 (minor and major *p*-cym-CH_Ar_, C-1′–C-5′), 63.0 (major C-6′), 62.6 (minor C-6′), 31.4 (major *i*-Pr-*C*H), 31.1 (minor *i*-Pr-*C*H), 23.5, 21.3 (minor *i*-Pr-*C*H_3_), 22.5, 22.2 (major *i*-Pr-*C*H_3_), 18.7 (major C_6_H_4_-*C*H_3_), 18.0 (minor C_6_H_4_-*C*H_3_). ESI-HRMS positive mode (*m/z*): calcd for C_51_H_45_ClN_3_O_10_Os^+^ [M-PF_6_]^+^ 1086.2406. Found: [M-PF_6_]^+^ 1086.2391.

#### 5.1.10. Complex **Os-4**

Complex **Os-4** was prepared from complex **Os-dimer** (20.0 mg, 0.0253 mmol), compound **L-4** (36.7 mg, 0.0506 mmol, 2.0 eq.) and TlPF_6_ (17.6 mg, 0.0504 mmol) according to general method I. After filtration and removal of the solvent the residue was dissolved in CHCl_3_ (5 mL) and Et_2_O (10 mL) was added. The precipitated product was filtered off, then washed with a solvent mixture of CHCl_3_-Et_2_O = 1:2 (2 mL) to give 48.4 mg (78%) orange powder. R_f_: 0.69 (9:1 CHCl_3_-MeOH). Diastereomeric ratio: 3:2. ^1^H NMR (400 MHz, CDCl_3_) δ (ppm): 9.43 (d, *J* = 5.6 Hz, major Py-H-6), 9.22 (d, *J* = 5.6 Hz, minor Py-H-6), 8.21–7.24 (m, minor and major Ar, Py-H-3–Py-H-5), 6.42 (pt, *J* = 10.1, 10.0 Hz, minor H-2′), 6.20 (d, *J* = 3.4 Hz, minor H-4′), 6.17 (d, *J* = 2.6 Hz, major H-4′), 6.06, 6.04 (2 d, *J* = 5.8 Hz in each, 2 × major *p*-cym-CH_Ar_), 6.03–5.95 (m, major H-2′ and H-3′), 5.88–5.84 (m, minor and 2 × major *p*-cym-CH_Ar_), 5.80 (dd, *J* = 10.1, 3.4 Hz, minor H-3′), 5.76, 5.56, 5.53 (3 d, *J* = 5.8 Hz in each, 3 × minor *p*-cym-CH_Ar_), 5.48 (d, *J* = 10.2 Hz, minor H-1′), 5.39 (d, *J* = 9.7 Hz, major H-1′), 4.73–4.63 (m, minor and major H-5′, H-6′a), 4.54 (dd, *J* = 10.3, 4.1 Hz, major H-6′b), 4.48 (m, minor H-6′b), 2.63 (hept, *J* = 6.9 Hz, minor *i*-Pr-CH), 2.57 (hept, *J* = 6.9 Hz, major *i*-Pr-CH), 2.10 (s, major C_6_H_4_-C*H_3_*), 2.00 (s, minor C_6_H_4_-C*H_3_*), 1.13, 1.06 (2 d, *J* = 6.9 Hz in each, 2 × minor *i*-Pr-C*H*_3_), 1.10, 1.04 (2 d, *J* = 6.9 Hz in each, 2 × major *i*-Pr-C*H*_3_); ^13^C NMR (100 MHz, CDCl_3_) δ (ppm): 169.0, 168.8, 166.2, 166.1, 165.9, 165.7, 165.6, 165.5, 165.4, 165.3, 165.0, 164.9 (minor and major C=O, OD-C-2, OD-C-5), 158.1 (major Py-C-6), 156.6 (minor Py-C-6), 140.4 (major Py-C-4), 140.2 (minor Py-C-4), 140.1 (minor Py-C-2), 139.1 (major Py-C-2), 134.5, 134.2, 133.9, 133.8 (2), 133.6, 133.5 (2), 131.6–128.0 (minor and major Ar, Py-C-5), 125.4 (major Py-C-3), 125.3 (minor Py-C-3), 97.9, 94.9 (major *p*-cym-C_qAr_), 95.6, 93.7 (minor *p*-cym-C_qAr_), 80.9, 78.0, 77.3, 76.7, 76.6, 76.3, 75.3, 74.9 (2), 74.5, 72.4, 72.1, 71.7, 71.3, 68.7, 68.4, 68.3, 66.8 (minor and major *p*-cym-CH_Ar_, C-1′–C-5′), 62.4 (major C-6′), 62.2 (minor C-6′), 31.3 (major *i*-Pr-*C*H), 31.0 (minor *i*-Pr-*C*H), 23.5, 21.3 (minor *i*-Pr-*C*H_3_), 22.6, 22.1 (major *i*-Pr-*C*H_3_), 18.6 (major C_6_H_4_-*C*H_3_), 17.9 (minor C_6_H_4_-*C*H_3_). ESI-HRMS positive mode (*m/z*): calcd for C_51_H_45_ClN_3_O_10_Os^+^ [M-PF_6_]^+^ 1086.2406. Found: [M-PF_6_]^+^ 1086.2390.

#### 5.1.11. Complex **Os-5**

Complex **Os-5** was prepared from complex **Os-dimer** (10.0 mg, 0.0126 mmol), compound **L-5** (15.0 mg, 0.0254 mmol, 2.0 eq.) and TlPF_6_ (8.8 mg, 0.0252 mmol), according to general method I. After filtration and removal of the solvent, the residue was dissolved in CHCl_3_ (3 mL) and Et_2_O (12 mL) was added. The precipitated product was filtered off, then washed with a solvent mixture of CHCl_3_-Et_2_O = 1:2 (2 mL) to give 23.3 mg (84%) orange powder. R_f_: 0.34 (95:5 CHCl_3_-MeOH). Diastereomeric ratio: 7:5. ^1^H NMR (400 MHz, CDCl_3_) δ (ppm): 9.40 (d, *J* = 5.6 Hz, major Py-H-6), 9.22 (d, *J* = 5.6 Hz, minor Py-H-6), 8.17–7.32 (m, minor and major Ar, Py-H-3–Py-H-5), 6.11 (pt, *J* = 9.0, 9.0 Hz, major H-2′ or H-3′), 6.08–6.03 (m, major and minor H-2′ or H-3′and *p*-cym-CH_Ar_), 5.91 (d, *J* = 5.8 Hz, minor *p*-cym-CH_Ar_), 5.86, 5.84 (2 d, *J* = 6.2 Hz in each, 2 × major *p*-cym-CH_Ar_), 5.76 (pt, *J* = 9.1, 9.0 Hz, minor H-2′ or H-3′), 5.75 (m, major *p*-cym-CH_Ar_), 5.64 (d, *J* = 5.8 Hz, minor *p*-cym-CH_Ar_), 5.62–5.51 (m, major and minor H-4′, minor *p*-cym-CH_Ar_), 5.30 (d, *J* = 9.7 Hz, minor H-1′), 5.25 (d, *J* = 9.0 Hz, major H-1′), 4.66 (dd, *J* = 11.7, 5.2 Hz, major H-5′eq), 4.60 (dd, *J* = 11.4, 5.5 Hz, minor H-5′eq), 3.88 (pt, *J* = 11.4, 9.7 Hz, major H-5′ax), 3.87 (pt, *J* = 11.4, 10.4 Hz, minor H-5′ax), 2.63 (hept, *J* = 6.9 Hz, minor *i*-Pr-CH), 2.60 (hept, *J* = 6.9 Hz, major *i*-Pr-CH), 2.11 (s, major C_6_H_4_-C*H_3_*), 2.04 (s, minor C_6_H_4_-C*H_3_*), 1.14, 1.12, 1.06, 1.05 (4 d, *J* = 6.9 Hz in each, minor and major 2 × *i*-Pr-C*H*_3_); ^13^C NMR (100 MHz, CDCl_3_) δ (ppm): 167.0, 168.8, 165.8, 165.7 (2), 165.6, 165.5, 165.4, 165.3, 165.1 (minor and major C=O, OD-C-2, OD-C-5), 158.0 (major Py-C-6), 156.7 (minor Py-C-6), 140.3 (major Py-C-4), 140.2 (minor Py-C-4), 140.0 (minor Py-C-2), 139.2 (major Py-C-2), 134.5, 134.0, 133.9 (2), 133.8, 133.6, 131.5–128.0 (minor and major Ar, Py-C-5), 125.3 (2) (minor and major Py-C-3), 97.7, 94.9 (major *p*-cym-C_qAr_), 95.7, 93.9 (minor *p*-cym-C_qAr_), 80.7, 78.3, 77.3, 76.7, 75.2, 74.9, 74.8, 74.4, 73.0, 72.1, 71.9, 71.8, 70.7, 69.7, 69.6, 69.0 (minor and major *p*-cym-CH_Ar_, C-1′–C-4′), 67.9 (minor C-5′), 67.5 (major C-5′), 31.3 (major *i*-Pr-*C*H), 31.1 (minor *i*-Pr-*C*H), 23.4, 21.4 (minor *i*-Pr-*C*H_3_), 22.7, 22.1 (major *i*-Pr-*C*H_3_), 18.6 (major C_6_H_4_-*C*H_3_), 18.0 (minor C_6_H_4_-*C*H_3_). ESI-HRMS positive mode (*m/z*): calcd for C_43_H_39_ClN_3_O_8_Os^+^ [M-PF_6_]^+^ 952.2037. Found: [M-PF_6_]^+^ 952.2025.

#### 5.1.12. Complex **Os-6**

Complex **Os-6** was prepared from complex **Os-dimer** (20.0 mg, 0.0253 mmol), compound **L-6** (36.7 mg, 0.0506 mmol, 2.0 eq.) and TlPF_6_ (17.6 mg, 0.0504 mmol) according to general method I. After filtration and removal of the solvent, the residue was dissolved in CHCl_3_ (5 mL) and Et_2_O (10 mL) was added. The precipitated product was filtered off, then washed with a solvent mixture of CHCl_3_-Et_2_O = 1:1 (2 mL) to give 52.9 mg (85%) yellow powder. R_f_: 0.57 (9:1 CHCl_3_-MeOH). Diastereomeric ratio: 1:1. ^1^H NMR (400 MHz, CDCl_3_) δ (ppm): 9.19 (2H, d, *J* = 5.3 Hz, 2 × Py-H-6), 9.07, 9.05 (2 × 1H, 2 s, 2 × Tria-H-5), 8.05–7.75, 7.53–7.23 (46H, m, 2 × 20 × Ar, 2 × Py-H-3–Py-H-5), 6.58, 6.24–5.60 (14H, m, 2 × H-2′, 2 × H-3′, 2 × H-4′, 2 × 4 × *p*-cym-CH_Ar_), 6.49, 6.43 (2 × 1H, 2 d, *J* = 9.2 and 9.0 Hz, respectively, 2 × H-1′), 4.79–4.53 (6H, m, 2 × H-5′, 2 × H-6′a, 2 × H-6′b), 2.35, 2.16 (2 × 1H, 2 hept, *J* = 6.9 Hz in each, 2 × *i*-Pr-CH), 2.14, 2.07 (2 × 3H, 2 s, 2 × C_6_H_4_-C*H_3_*), 0.89, 0.86, 0.75, 0.63 (2 × 2 × 3H, 2 × 2 d, *J* = 6.9 Hz in each, 2 × 2 × *i*-Pr-C*H*_3_); ^13^C NMR (100 MHz, CDCl_3_) δ (ppm): 166.3, 166.2, 165.6, 165.5, 165.2 (2), 164.9, 164.8 (2 × 4 × C=O), 155.8, 155.7 (2 × Py-C-6), 148.4, 148.1, 148.0, 147.9 (2 × Tria-C-4, 2 × Py-C-2), 140.3, 140.2 (2 × Py-C-4), 134.3, 134.2, 133.9, 133.8, 133.7, 133.6, 133.5, 133.3, 130.2–128.4, 128.0, 127.9, 127.7, 127.6 (Ar, 2 × Py-C-5, 2 × Tria-C-5), 122.5, 122.4 (2 × Py-C-3), 97.0, 96.4, 96.1, 95.2 (2 × 2 × *p*-cym-C_qAr_), 87.2, 86.7 (2 × C-1′), 78.2, 77.7, 76.9, 76.8, 76.1, 75.7, 74.9, 74.5, 73.7, 73.6, 72.8, 72.7, 71.7, 70.4, 68.7, 68.6 (2 × 4 × *p*-cym-CH_Ar_, 2 × C-1′ − C-5′), 62.8, 62.7 (2 × C-6′), 31.1 (2) (2 × *i*-Pr-*C*H), 22.8, 22.6, 21.9, 21.5 (2 × 2 × *i*-Pr-*C*H_3_), 18.6, 18.5 (2 × C_6_H_4_-*C*H_3_). ESI-HRMS positive mode (*m/z*): calcd for C_51_H_46_ClN_4_O_9_Os^+^ [M-PF_6_]^+^ 1085.2566. Found: 1085.2555.

#### 5.1.13. Complex **Ir-1**

Complex **Ir-1** was prepared from complex **Ir-dimer** (10.0 mg, 0.0126 mmol), compound **L-1** (18.9 mg, 0.0251 mmol, 2.0 eq.) and TlPF_6_ (8.8 mg, 0.0249 mmol) according to general method I. After filtration and removal of the solvent, the residue was dissolved in CHCl_3_ (3 mL) and Et_2_O (6 mL) was added. The precipitated product was filtered off, then washed with a solvent mixture of CHCl_3_-Et_2_O = 1:1 (3 mL) to give 21.7 mg (70%) yellow powder. R_f_: 0.26 (95:5 CHCl_3_-MeOH). Diastereomeric ratio: 6:1. ^1^H NMR (400 MHz, CDCl_3_) δ (ppm): 8.91 (m, minor Py-H-6), 8.90 (d, *J* = 5.4 Hz, major Py-H-6), 8.31–7.76, 7.54–7.18 (m, minor and major Ar, Py-H-3–Py-H-5), 6.41 (pt, *J* = 9.9, 9.7 Hz, minor H-2′ or H-3′ or H-4′), 6.33 (pt, *J* = 9.9, 9.9 Hz, major H-2′ or H-3′ or H-4′), 6.07 (pt, *J* = 9.5, 9.5 Hz, minor H-2′ or H-3′ or H-4′), 6.00 (pt, *J* = 9.7, 9.6 Hz, major H-2′ or H-3′ or H-4′), 5.86 (pt, *J* = 9.8, 9.7 Hz, major H-2′ or H-3′ or H-4′), 5.80 (pt, *J* = 9.7, 9.6 Hz, minor H-2′ or H-3′ or H-4′), 5.36 (d, *J* = 10.2 Hz, minor H-1′), 5.20 (d, *J* = 10.2 Hz, major H-1′), 4.73 (dd, *J* = 12.5, 2.9 Hz, major H-6′a), 4.64–4.57 (m, minor H-6′a), 4.59 (dd, *J* = 12.5, 5.8 Hz, major H-6′b), 4.54–4.41 (m, minor H-6′b and H-5′), 4.48 (ddd, *J* = 9.1, 5.8, 2.9 Hz, major H-5′), 1.75 (s, major Cp*-C*H*_3_), 1.73 (s, minor Cp*-C*H*_3_); ^13^C NMR (100 MHz, CDCl_3_) δ (ppm) (only major isomer): 179.0, 166.3, 165.9, 165.3, 164.9, 164.6 (4 × C=O, OD-C-3, OD-C-5), 152.8 (Py-C-6), 140.9 (Py-C-4), 139.5 (Py-C-2), 133.9, 133.8, 133.7, 133.6, 133.0, 130.4–128.0 (Ar, Py-C-3, Py-C-5), 90.2 (Cp*), 77.2, 73.8, 70.6, 70.4, 69.0 (C-1′–C-5′), 63.6 (C-6′), 9.1 (Cp*-*C*H_3_). ESI-HRMS positive mode (*m/z*): calcd for C_51_H_46_ClN_3_O_10_Ir^+^ [M-PF_6_]^+^ 1088.2491. Found: [M-PF_6_]^+^ 1088.2493.

#### 5.1.14. Complex **Ir-2**

Complex **Ir-2** was prepared from complex **Ir-dimer** (20.0 mg, 0.0251 mmol), compound **L-2** (15.5 mg, 0.501 mmol, 2 eq.) and TlPF_6_ (17.5 mg, 0.0501 mmol) according to general method II. The crude product was purified by recrystallization from a solvent mixture of MeOH-*i-*PrOH (2 mL and 4 mL, respectively), then filtered and washed with cold *i-*PrOH (1 mL) to give 35.2 mg (86%) yellow powder. Diastereomeric ratio: 1:1. ^1^H NMR (400 MHz, CD_3_OD) δ (ppm): 9.11 (2H, m, 2 × Py-H-6), 8.63 (2H, d, *J* = 7.8 Hz, 2 × Py-H-3), 8.46 (2H, t, *J* = 7.8 Hz, 2 × Py-H-4), 8.12, 8.10 (2 × 1H, ddd, *J* = 7.4, 5.5, 1.5 Hz in each, 2 × Py-H-5), 4.72, 4.56 (2 × 1H, 2 d, *J* = 9.9 Hz in each, 2 × H-1′), 4.10, 4.04 (2 × 1H, 2 pt, *J* = 9.4, 9.4 Hz in each, 2 × H-2′ or H-3′ or H-4′), 4.00, 3.87 (2 × 1H, 2 dd, *J* = 12.0, 1.8 Hz in each, 2 × H-6′a), 3.76–3.38 (8H, m, 2 × H-2′ and/or H-3′ and/or H-4′, 2 × H-5′, 2 × H-6′b), 1.79, 1.78 (2 × 15H, 2 s, 2 × 5 × Cp*-C*H*_3_); ^13^C NMR (90 MHz, CD_3_OD) δ (ppm): 180.2, 180.1, 168.2, 168.0 (2 × OD-C-3, 2 × OD-C-5), 154.3, 154.2 (2 × Py-C-6), 142.6, 142.5 (2 × Py-C-4), 141.7, 141.5 (2 × Py-C-2), 133.7, 133.6 (2 × Py-C-5), 128.5, 128.4 (2 × Py-C-3), 91.4, 91.3 (Cp*), 83.1, 83.1, 79.3, 78.7, 74.1, 74.0, 73.9, 73.0, 71.3, 71.1 (2 × C-1′ − C-5′), 63.2, 62.9 (2 × C-6′), 9.5, 9.3 (2 × 5 × Cp*-*C*H_3_). ESI-HRMS positive mode (*m/z*): C_23_H_30_ClN_3_O_6_Ir^+^ [M-PF_6_]^+^ 672.1439. Found: [M-PF_6_]^+^ 672.1446.

#### 5.1.15. Complex **Ir-3**

Complex **Ir-3** was prepared from complex **Ir-dimer** (20.0 mg, 0.0251 mmol), compound **L-3** (36.4 mg, 0.0502 mmol, 2.0 eq.) and TlPF_6_ (17.5 mg, 0.0501 mmol) according to general method I. It was then purified by column chromatography (95:5 CHCl_3_-MeOH) to give 49.3 mg (80%) yellow powder. R_f_: 0.32 (95:5 CHCl_3_-MeOH). Diastereomeric ratio: 5:2. ^1^H NMR (400 MHz, CDCl_3_) δ (ppm): 8.95 (d, *J* = 5.5 Hz, minor Py-H-6), 8.78 (d, *J* = 5.5 Hz, major Py-H-6), 8.22–7.78, 7.57–7.26 (m, minor and major Ar, Py-H-3–Py-H-5), 6.32 (pt, *J* = 9.7, 9.6 Hz, major H-2′ or H-3′ or H-4′), 6.19 (pt, *J* = 9.7, 9.5 Hz, minor H-2′ or H-3′ or H-4′), 6.04 (pt, *J* = 9.5, 9.5 Hz, major H-2′ or H-3′ or H-4′), 5.87 (pt, *J* = 9.8, 9.7 Hz, minor H-2′ or H-3′ or H-4′), 5.86 (pt, *J* = 9.8, 9.7 Hz, major H-2′ or H-3′ or H-4′), 5.78 (pt, *J* = 9.7, 9.7 Hz, minor H-2′ or H-3′ or H-4′), 5.51 (d, *J* = 10.2 Hz, major H-1′), 5.42 (d, *J* = 9.9 Hz, minor H-1′), 4.72 (dd, *J* = 12.8, 2.4 Hz, minor H-6′a), 4.67 (dd, *J* = 12.6, 2.4 Hz, major H-6′a), 4.57 (dd, *J* = 12.8, 5.6 Hz, minor H-6′b), 4.53 (dd, *J* = 12.6, 4.3 Hz, major H-6′b), 4.47 (ddd, *J* = 9.8, 4.3, 2.4 Hz, major H-5′), 4.46 (m, minor H-5′), 1.61 (s, minor Cp*-C*H*_3_), 1.50 (s, major Cp*-C*H*_3_); ^13^C NMR (90 MHz, CDCl_3_) δ (ppm): 169.4, 166.2, 165.8, 165.3, 165.2, 165.0 (major C=O, OD-C-2, OD-C-5), 169.2, 166.2, 165.8, 165.7, 165.6, 165.4 (minor C=O, OD-C-2, OD-C-5), 153.8 (minor Py-C-6), 152.5 (major Py-C-6), 140.8 (minor Py-C-4), 140.4 (major Py-C-4), 140.0 (major Py-C-2), 138.9 (minor Py-C-2), 134.4, 133.9, 133.8, 133.7, 133.6, 133.5, 133.4, 133.3, 132.0, 130.9, 130.5, 130.1–127.8 (minor and major Ar, Py-C-5), 125.8 (major Py-C-3), 125.7 (minor Py-C-3), 90.3 (minor Cp*), 90.0 (major Cp*), 77.7, 72.9, 71.8, 71.3, 68.8 (minor C-1′–C-5′), 77.3, 74.2, 71.2, 69.1, 68.7 (major C-1′–C-5′), 63.0 (minor C-6′), 62.6 (major C-6′), 8.8 (minor Cp*-*C*H_3_), 8.5 (major Cp*-*C*H_3_). ESI-HRMS positive mode (*m/z*): calcd for C_53_H_53_N_3_O_12_Ir^+^ [M-PF_6_-Cl+OMe+MeOH]^+^ 1116.3253. Found: [M-PF_6_-Cl+OMe+MeOH]^+^ 1116.3248.

#### 5.1.16. Complex **Ir-4**

Complex **Ir-4** was repared from complex **Ir-dimer** (20.0 mg, 0.0251 mmol), compound **L-4** (36.7 mg, 0.0506 mmol, 2.0 eq.) and TlPF_6_ (17.5 mg, 0.0501 mmol) according to general method I. After filtration and removal of the solvent, the residue was dissolved in CHCl_3_ (5 mL) and Et_2_O (10 mL) was added. The precipitated product was filtered off, then washed with a solvent mixture of CHCl_3_-Et_2_O = 1:1 (2 mL) to give 47.5 mg (77%) yellow powder. R_f_: 0.60 (9:1 CHCl_3_-MeOH). Diastereomeric ratio: 2:1. ^1^H NMR (400 MHz, CDCl_3_) δ (ppm): 8.94 (d, *J* = 5.5 Hz, minor Py-H-6), 8.79 (d, *J* = 5.5 Hz, major Py-H-6), 8.22–7.79, 7.69–7.24 (m, minor and major Ar, Py-H-3–Py-H-5), 6.54 (pt, *J* = 10.2, 10.1 Hz, major H-2′), 6.19 (dd, *J* = 3.3, 1.0 Hz, major H-4′), 6.17 (dd, *J* = 3.3, 1.0 Hz, minor H-4′), 6.05 (pt, *J* = 10.1, 9.8 Hz, minor H-2′), 5.94 (dd, *J* = 10.1, 3.3 Hz, minor H-3′), 5.75 (dd, *J* = 10.1, 3.3 Hz, major H-3′), 5.50 (d, *J* = 10.2 Hz, major H-1′), 5.44 (d, *J* = 9.8 Hz, minor H-1′), 4.74–4.42 (m, minor and major H-5′, H-6′a, H-6′b), 1.62 (s, minor Cp*-C*H*_3_), 1.51 (s, major Cp*-C*H*_3_); ^13^C NMR (100 MHz, CDCl_3_) δ (ppm): 169.4, 166.1, 165.7, 165.5, 165.3, 165.1 (major C=O, OD-C-2, OD-C-5), 169.2, 166.2, 165.7, 165.6, 165.5, 165.4 (minor C=O, OD-C-2, OD-C-5), 153.5 (minor Py-C-6), 152.3 (major Py-C-6), 140.8 (minor Py-C-4), 140.5 (major Py-C-4), 140.2 (major Py-C-2), 139.2 (minor Py-C-2), 134.3, 134.2, 133.8 (2), 133.7, 133.6, 133.5, 133.4, 131.7, 130.7–128.2 (minor and major Ar, Py-C-5), 125.9 (major Py-C-3), 125.7 (minor Py-C-3), 90.3 (minor Cp*), 90.0 (major Cp*), 76.6, 72.1, 71.5, 68.5, 68.2 (minor C-1′–C-5′), 76.2, 72.8, 71.4, 68.3, 66.1 (major C-1′–C-5′), 62.4 (minor C-6′), 62.2 (major C-6′), 8.8 (minor Cp*-*C*H_3_), 8.5 (major Cp*-*C*H_3_). ESI-HRMS positive mode (*m/z*): calcd for C_51_H_46_ClN_3_O_10_Ir^+^ [M-PF_6_]^+^ 1088.2491. Found: [M-PF_6_]^+^ 1088.2492.

#### 5.1.17. Complex **Ir-5**

Complex **Ir-5** was prepared from complex **Ir-dimer** (20.0 mg, 0.0251 mmol), compound **L-5** (29.7 mg, 0.0502 mmol, 2.0 eq.) and TlPF_6_ (17.5 mg, 0.0501 mmol) according to general method I. After filtration and removal of the solvent, the residue was dissolved in CHCl_3_ (5 mL) and Et_2_O (10 mL) was added. The precipitated product was filtered off, then washed with a solvent mixture of CHCl_3_-Et_2_O = 1:2 (1 mL) to give 34.6 mg (72%) yellow powder. R_f_: 0.44 (95:5 CHCl_3_-MeOH). Diastereomeric ratio: 3:1. ^1^H NMR (400 MHz, CDCl_3_) δ (ppm): 8.93 (d, *J* = 5.5 Hz, minor Py-H-6), 8.80 (d, *J* = 5.5 Hz, major Py-H-6), 8.23–7.80, 7.57–7.30 (m, minor and major Ar, Py-H-3–Py-H-5), 6.21 (pt, *J* = 9.7, 9.5 Hz, major H-2′ or H-3′), 6.11 (pt, *J* = 9.2, 9.2 Hz, minor H-2′ or H-3′), 6.00 (pt, *J* = 9.6, 9.5 Hz, major H-2′ or H-3′), 5.78 (pt, *J* = 9.2, 9.2 Hz, minor H-2′ or H-3′), 5.59 (ddd, *J* = 10.1, 9.6, 5.4 Hz, minor H-4′), 5.51 (ddd, *J* = 10.4, 9.9, 5.4 Hz, major H-4′), 5.33 (d, *J* = 10.0 Hz, major H-1′), 5.29 (d, *J* = 9.3 Hz, minor H-1′), 4.65 (dd, *J* = 11.6, 5.4 Hz, minor H-5′eq), 4.57 (dd, *J* = 11.3, 5.4 Hz, major H-5′eq), 3.87 (pt, *J* = 11.3, 10.4 Hz, major H-5′ax), 3.86 (pt, *J* = 11.6, 10.1 Hz, minor H-5′ax), 1.63 (s, minor Cp*-C*H*_3_), 1.52 (s, major Cp*-C*H*_3_); ^13^C NMR (90 MHz, CDCl_3_) δ (ppm): 169.4, 169.1, 165.8 (2), 165.7, 165.6 (3), 165.5, 165.1 (minor and major C=O, OD-C-2, OD-C-5), 153.5 (minor Py-C-6), 152.4 (major Py-C-6), 140.8 (minor Py-C-4), 140.5 (major Py-C-4), 140.1 (major Py-C-2), 139.2 (minor Py-C-2), 134.3, 133.8, 133.7, 133.5, 131.7, 130.8–128.1 (minor and major Ar, Py-C-5), 125.9 (major Py-C-3), 125.6 (minor Py-C-3), 90.3 (minor Cp*), 90.0 (major Cp*), 73.5, 71.7, 69.5, 69.0 (major C-1′–C-4′), 72.3, 72.2, 70.8, 69.2 (minor C-1′–C-4′), 67.8 (major C-5′), 67.7 (minor C-5′), 8.8 (minor Cp*-*C*H_3_), 8.6 (major Cp*-*C*H_3_). ESI-HRMS positive mode (*m/z*): calcd for C_43_H_40_ClN_3_O_8_Ir^+^ [M-PF_6_]^+^ 954.2128. Found: [M-PF_6_]^+^ 954.2121.

#### 5.1.18. Complex **Ir-6**

Complex **Ir-6** was prepared from complex **Ir-dimer** (20.0 mg, 0.0251 mmol), compound **L-6** (36.4 mg, 0.0502 mmol, 2.0 eq.) and TlPF_6_ (17.5 mg, 0.0501 mmol) according to general method I. After filtration and removal of the solvent, the residue was dissolved in CHCl_3_ (5 mL) and Et_2_O (10 mL) was added. The precipitated product was filtered off, washed with a solvent mixture of CHCl_3_-Et_2_O = 1:1 mixture (2 mL) to give 51.9 mg (84%) yellow powder. R_f_: 0.58 (9:1 CHCl_3_-MeOH). Diastereomeric ratio: 2:1. ^1^H NMR (400 MHz, CDCl_3_) δ (ppm): 9.20 (s, minor Tria-H-5), 9.13 (s, major Tria-H-5), 8.68 (1 signal, minor and major Py-H-6), 8.05–7.82, 7.58–7.23 (m, minor and major Ar, Py-H-3–Py-H-5), 6.53 (d, *J* = 9.3 Hz, major H-1′), 6.48 (d, *J* = 8.5 Hz, minor H-1′), 6.46 (pt, *J* = 9.4, 9.2 Hz, major H-2′ or H-3′ or H-4′), 6.22 (pt, *J* = 9.2, 9.2 Hz, minor H-2′ or H-3′ or H-4′), 6.19 (pt, *J* = 9.2, 8.7 Hz, minor H-2′ or H-3′ or H-4′), 6.13 (pt, *J* = 9.5, 9.5 Hz, major H-2′ or H-3′ or H-4′), 5.98 (pt, *J* = 9.5, 9.5 Hz, minor H-2′ or H-3′ or H-4′), 5.92 (pt, *J* = 9.9, 9.7 Hz, major H-2′ or H-3′ or H-4′), 4.78–4.54 (m, minor and major H-5′, H-6′a, H-6′b), 1.62 (s, major Cp*-C*H*_3_), 1.57 (s, minor Cp*-C*H*_3_); ^13^C NMR (100 MHz, CDCl_3_) δ (ppm): 166.3, 165.6, 165.2, 164.6 (minor C=O), 166.2, 165.5, 165.3, 164.7 (major C=O), 151.5 (2) (minor and major Py-C-6), 148.5, 147.8 (minor Tria-C-4, Py-C-2), 148.1, 147.9 (major Tria-C-4, Py-C-2), 140.6 (minor Py-C-4), 140.5 (major Py-C-4), 134.1, 134.0, 133.8, 133.7, 133.6, 133.5, 133.4, 133.3, 130.2–127.8 (minor and major Ar, Py-C-5, Tria-C-5), 123.0 (minor Py-C-3), 122.9 (major Py-C-3), 89.6 (major Cp*), 89.5 (minor Cp*), 87.3 (minor C-1′), 87.0 (major C-1′), 76.1, 72.9, 71.4, 68.7 (minor C-2′–C-5′), 75.8, 73.6, 70.3, 68.6 (major C-2′–C-5′), 62.8 (minor C-6′), 62.7 (major C-6′), 8.7 (minor and major Cp*-*C*H_3_). ESI-HRMS positive mode (*m/z*): calcd for C_51_H_47_ClN_4_O_9_Ir^+^ [M-PF_6_]^+^ 1087.2655. Found: [M-PF_6_]^+^ 1087.2647.

#### 5.1.19. Complex **Ir-7**

Complex **Ir-7** was prepared from complex **Ir-dimer** (20.0 mg, 0.0251 mmol), compound **L-7** (24.0 mg, 0.0503 mmol, 2.0 eq.) and TlPF_6_ (17.5 mg, 0.0501 mmol) according to general method I. After filtration and removal of the solvent, the residue was dissolved in CHCl_3_ (5 mL) and Et_2_O (10 mL) was added. The precipitated product was filtered off, then washed with a solvent mixture of CHCl_3_-Et_2_O = 1:2 (2 mL) to give 40.0 mg (81%) orange powder. R_f_: 0.37 (95:5 CHCl_3_-MeOH). Diastereomeric ratio: 5:2. ^1^H NMR (400 MHz, CDCl_3_) δ (ppm): 8.93 (d, *J* = 5.5 Hz, minor Py-H-6), 8.85 (d, *J* = 5.5 Hz, major Py-H-6), 8.30–8.17 (m, minor and major Py-H-3, Py-H-4), 7.95 (ddd, *J* = 7.5, 5.5, 1.8 Hz, minor Py-H-5), 7.85 (ddd, *J* = 7.1, 5.5, 1.6 Hz, major Py-H-5), 5.99 (pt, *J* = 10.1, 10.0 Hz, major H-2′), 5.58–5.53 (m, minor H-2′, minor and major H-4′), 5.28 (dd, *J* = 10.1, 3.4 Hz, minor H-3′), 5.22 (dd, *J* = 10.0, 3.4 Hz, major H-3′), 5.03 (d, *J* = 10.2 Hz, major H-1′), 5.02 (d, *J* = 10.0 Hz, minor H-1′), 4.23–4.07 (m, minor and major H-5′, H-6′a, H-6′b), 2.25, 2.24, 2.17, 2.08, 2.07, 2.04, 2.03 (2) (singlets, minor and major COC*H_3_*), 1.79 (2) (2 s, minor and major Cp*-C*H*_3_); ^13^C NMR (90 MHz, CDCl_3_) δ (ppm): 170.6, 170.5, 170.3, 170.0, 169.6, 169.5, 165.0, 165.3 (minor and major C=O, OD-C-2, OD-C-5), 153.4 (minor Py-C-6), 152.4 (major Py-C-6), 140.9 (minor Py-C-4), 140.5 (major Py-C-4), 140.1 (major Py-C-2), 139.2 (minor, Py-C-2), 131.7 (minor, Py-C-5), 130.8 (major, Py-C-5), 126.0 (2) (minor and major Py-C-3), 90.3 (minor Cp*), 90.2 (major Cp*), 75.8, 71.6, 71.1, 67.1, 67.0 (minor C-1′–C-5′), 75.6, 71.8, 71.0, 67.2, 65.4 (major C-1′–C-5′), 61.6 (major C-6′), 61.5 (minor C-6′), 20.9, 20.8, 20.6, 20.5 (minor and major CO*C*H_3_), 8.9 (minor Cp*-*C*H_3_), 8.8 (major Cp*-*C*H_3_). ESI-HRMS positive mode (*m/z*): calcd for C_31_H_38_ClN_3_O_10_Ir^+^ [M-PF_6_]^+^ 840.1863. Found: [M-PF_6_]^+^ 840.1862.

#### 5.1.20. Complex **Ir-8**

Complex **Ir-8** was prepared from complex **Ir-dimer** (20.0 mg, 0.0251 mmol), compound **L-8** (15.5 mg, 0.501 mmol, 2 eq.) and TlPF_6_ (17.5 mg, 0.0501 mmol) according to general method II. The crude product was purified by recrystallization from *i-*PrOH, then filtered and washed with cold *i-*PrOH (1 mL) to give 32.3 mg (79%) yellow powder. Diastereomeric ratio: 1:1. ^1^H NMR (400 MHz, CD_3_OD) δ (ppm): 9.08 (2H, d, *J* = 5.4 Hz, 2 × Py-H-6), 8.47 (2H, d, *J* = 7.9 Hz, 2 × Py-H-3), 8.40 (2H, t, *J* = 7.8 Hz, 2 × Py-H-4), 8.00 (2H, t, *J* = 6.6 Hz, 2 × Py-H-5), 4.87, 4.86 (2 × 1H, 2 d, *J* = 10.0 Hz in each, 2 × H-1′), 3.93, 3.91 (2 × 1H, 2 dd, *J* = 12.1, 2.1 Hz in each, 2 × H-6′a), 3.86, 3.84 (2 × 1H, 2 pt, *J* = 9.6, 9.2 Hz in each, 2 × H-2′ or H-3′ or H-4′), 3.72, 3.71 (2 × 1H, 2 dd, *J* = 12.1, 5.6 Hz in each, 2 × H-6′b), 3.60–3.54 (4H, m, 2 × H-2′ or H-3′ or H-4′, 2 × H-5′), 3.48, 3.46 (2 × 1H, 2 pt, *J* = 9.3, 9.2 Hz in each, 2 × H-2′ or H-3′ or H-4′), 1.82, 1.81 (30H, 2 s, 2 × 5 × Cp*-C*H*_3_); ^13^C NMR (100 MHz, CD_3_OD) δ (ppm): 170.5, 170.4, 169.0, 168,9 (2 × OD-C-2, 2 × OD-C-5), 154.8, 154.7 (2 × Py-C-6), 142.3 (2) (2 × Py-C-4), 141.2, 141.1 (2 × Py-C-2), 132.2 (2) (2 × Py-C-5), 126.6, 126.5 (2 × Py-C-3), 91.4 (2) (Cp*), 83.3, 83.2, 79.0, 78.9, 74.7, 74.6, 73.3, 73.2, 71.2, 71.1 (2 × C-1′ − C-5′), 62.7, 62.6 (2 × C-6′), 8.9 (2 × 5 × Cp*-*C*H_3_). ESI-HRMS positive mode (*m/z*): calcd for C_23_H_30_ClN_3_O_6_Ir^+^ [M-PF_6_]^+^ 672.1439. Found: [M-PF_6_]^+^ 672.1437.

#### 5.1.21. Complex **Ir-9**

Complex **Ir-9** was prepared from complex **Ir-dimer** (20.0 mg, 0.0251 mmol), compound **L-9** (15.5 mg, 0.501 mmol, 2 eq.) and TlPF_6_ (17.5 mg, 0.0501 mmol) according to general method II. The crude product was purified by recrystallization from *i-*PrOH (5 mL), then filtered and washed with cold *i-*PrOH (1 mL) to give 25.0 mg (61%) yellow powder. Diastereomeric ratio: 1:1. ^1^H NMR (360 MHz, CD_3_OD) δ (ppm): 9.08 (2H, d, *J* = 5.5 Hz, 2 × Py-H-6), 8.47 (2H, m, 2 × Py-H-3), 8.40 (2H, m, 2 × Py-H-4), 8.00 (2H, m, 2 × Py-H-5), 4.80, 4.79 (2 × 1H, 2 d, *J* = 9.9 Hz in each, 2 × H-1′), 4.20, 4.18 (2 × 1H, 2 pt, *J* = 9.7, 9.7 in each, 2 × H-2′), 4.01 (2H, d, *J* = 3.3 Hz, 2 × H-4′), 3.85–3.73 (6H, m, 2 × H-5′, 2 × H-6′a, 2 × H-6′b), 3.70, 3.69 (2 × 1H, 2 dd, J = 9.4, 3.3 in each, 2 × H-3′), 1.82 (2) (30H, s, 2 × 5 × Cp*-C*H*_3_); ^13^C NMR (90 MHz, CD_3_OD) δ (ppm): 170.5, 170.4, 169.1, 169.0 (2 × OD-C-2, 2 × OD-C-5), 154.7, 154.6 (2 × Py-C-6), 142.3 (2) (2 × Py-C-4), 141.2 (2) (2 × Py-C-2), 132.2 (2) (2 × Py-C-5), 126.6, 126.5 (2 × Py-C-3), 91.4 (2) (Cp*), 82.0, 81.9, 75.6, 75.5, 75.1, 75.0, 70.6, 70.5, 70.1, 70.0 (2 × C-1′ − C-5′), 62.7 (2) (2 × C-6′), 8.9 (2 × 5 × Cp*-*C*H_3_). ESI-HRMS positive mode (*m/z*): C_25_H_37_N_3_O_8_Ir^+^ [M-PF_6_-Cl+OMe+MeOH]^+^ 700.2206. Found: [M-PF_6_-Cl+OMe+MeOH]^+^ 700.2203.

#### 5.1.22. Complex **Ir-10**

Complex **Ir-10** was prepared from complex **Ir-dimer** (20.0 mg, 0.0251 mmol), compound **L-10** (14.0 mg, 0.501 mmol, 2 eq.) and TlPF_6_ (17.5 mg, 0.0501 mmol) according to general method II. The crude product was purified by recrystallization from *i-*PrOH, then filtered and washed with cold *i-*PrOH (1 mL) to give 24.0 mg (74%) yellow powder. Diastereomeric ratio: 1:1. ^1^H NMR (360 MHz, CD_3_OD) δ (ppm): 9.10 (2H, d, *J* = 5.5 Hz, 2 × Py-H-6), 8.47 (2H, m, 2 × Py-H-3), 8.41 (2H, m, 2 × Py-H-4), 8.01 (2H, m, 2 × Py-H-5), 4.81 (2H, d, *J* = 9.8 Hz, 2 × H-1′), 4.09 (2H, dd, *J* = 11.2, 5.3 Hz, 2 × H-5′eq), 3.83, 3.82 (2 × 1H, 2 pt, *J* = 9.5, 9.3 Hz in each, 2 × H-2′), 3.73–3.66 (2H, m, 2 × H-4′), 3.55–3.46 (4H, m, 2 × H-3′, 2 × H-5′ax), 1.83 (30H, s, 2 × 5 × Cp*-C*H*_3_); ^13^C NMR (90 MHz, CD_3_OD) δ (ppm): 170.5, 170.4, 169.2, 169.0 (2 × OD-C-2, 2 × OD-C-5), 154.7, 154.6 (2 × Py-C-6), 142.3 (2) (2 × Py-C-4), 141.2 (2) (2 × Py-C-2), 132.2 (2) (2 × Py-C-5), 126.6, 126.5 (2 × Py-C-3), 91.4 (2) (Cp*), 79.0, 78.9, 75.4, 75.3, 73.5, 73.2, 70.8 (2) (2 × C-1′ − C-4′), 71.8, 71.7 (2 × C-5′), 8.9 (2 × 5 × Cp*-*C*H_3_). ESI-HRMS positive mode (*m/z*): calcd for C_24_H_35_N_3_O_7_Ir^+^ [M-PF_6_-Cl+OMe+MeOH]^+^ 670.2100. Found: [M-PF_6_-Cl+OMe+MeOH]^+^ 670.2105.

#### 5.1.23. Complex **Ir-11**

Complex **Ir-11** was prepared from complex **Ir-dimer** (20.0 mg, 0.0251 mmol), compound **L-11** (23.9 mg, 0.0502 mmol, 2.0 eq.) and TlPF_6_ (17.5 mg, 0.0501 mmol) according to general method I. This was then purified by column chromatography (95:5 CHCl_3_-MeOH) to give 39.9 mg (81%) yellow powder. R_f_: 0.36 (95:5 CHCl_3_-MeOH). Diastereomeric ratio: 6:5. ^1^H NMR (400 MHz, CDCl_3_) δ (ppm): 9.11, 8.93 (2 × 1H, 2 s, 2 × Tria-H-5), 8.76, 8.72 (2 × 1H, 2 d, *J* = 5.6 Hz in each, 2 × Py-H-6), 8.13–8.04 (4H, m, 2 × Py-H-3, 2 × Py-H-4), 7.66–7.59 (2H, m, 2 × Py-H-5), 6.07 (2H, d, *J* = 9.4, 2 × H-1′), 5.95, 5.91 (2 × 1H, 2 pt, *J* = 9.4, 9.4 in each, 2 × H-2′ and/or 2 × H-3′ and/or H-4′), 5.45 (2) (2 × 1H, 2 pt, *J* = 9.5, 9.4 in each, 2 × H-2′ and/or 2 × H-3′ and/or H-4′), 5.35, 5.32 (2 × 1H, 2 pt, *J* = 10.0, 9.8 in each, 2 × H-2′ and/or 2 × H-3′ and/or H-4′), 4.37–4.16 (6H, m, 2 × H-5′, 2 × H-6′a, 2 × H-6′b), 2.10, 2.09 (2), 2.08, 2.04 (2), 1.94, 1.91 (24H, singlets, 2 × 4 × COC*H_3_*), 1.77, 1.76 (2 × 15H, 2 s, 2 × 5 × Cp*-C*H*_3_); ^13^C NMR (90 MHz, CDCl_3_) δ (ppm): 170.8 (2), 170.0, 169.9, 169.6 (2), 169.5, 169.2 (2 × 4 × C=O), 151.4, 151.3 (2 × Py-C-6), 148.6, 148.3, 148.2, 148.0 (2 × Py-C-2, 2 × Tria-C-4), 140.8, 140.6 (2 × Py-C-4), 127.8, 127.6 (2 × Py-C-5), 126.6 (2) (2 × Tria-C-5), 123.2 (2) (2 × Py-C-3), 89.7 (2) (2 × Cp*), 86.7, 86.4 (2 × C-1′), 75.6, 75.3, 73.2 (2), 69.8, 69.3, 67.5 (2) (2 × C-2′– C-5′), 61.6, 61.5 (2 × C-6′), 20.8, 20.7 (3), 20.6 (2), 20.5, 20.3 (2 × 4 × CO*C*H_3_), 8.9, 8.8 (2 × 5 × Cp*-*C*H_3_). ESI-HRMS positive mode (*m/z*): calcd for C_31_H_39_ClN_4_O_9_Ir^+^ [M-PF_6_]^+^ 839.2023. Found: [M-PF_6_]^+^ 839.2020.

#### 5.1.24. Complex **Ir-12**

Complex **Ir-12** was prepared from complex **Ir-dimer** (20.0 mg, 0.0251 mmol), compound **L-12** (15.5 mg, 0.503 mmol, 2 eq.) and TlPF_6_ (17.5 mg, 0.0501 mmol) according to general method II. The crude product was purified by recrystallization from *i-*PrOH (3 mL), filtered and washed with cold *i-*PrOH (1 mL) to give 23.4 mg (56%) yellow powder. Diastereomeric ratio: 1:1. ^1^H NMR (360 MHz, CD_3_OD) δ (ppm): 9.30, 9.29 (2 × 1H, 2 s, 2 × Tria-H-5), 8.94 (2H, d, *J* = 5.7 Hz, 2 × Py-H-6), 8.26–8.20 (4H, m, 2 × Py-H-3, 2 × Py-H-4), 7.75–7.71 (2H, m, 2 × Py-H-5), 5.91, 5.89 (2 × 1H, 2 d, *J* = 9.1 in each, 2 × H-1′), 3.97–3.53 (12H, m, 2 × H-2′ − H-5′, 2 × H-6′a, 2 × H-6′b), 1.78 (2) (2 × 15H, 2 s, 2 × 5 × Cp*-C*H*_3_); ^13^C NMR (90 MHz, CD_3_OD) δ (ppm): 153.5, 153.4 (2 × Py-C-6), 149.7 (2), 149.6, 149.5 (2 × Py-C-2, 2 × Tria-C-4), 141.9 (2) (2 × Py-C-4), 128.7 (2) (2 × Py-C-5), 126.1, 125.5 (2 × Tria-C-5), 123.5 (2) (2 × Py-C-3), 91.4, 91.3 (2 × C-1′), 91.0, 90.9 (2 × Cp*), 81.7, 81.6, 78.2, 78.1, 74.5, 74.4, 70.8, 70.7 (2 × C-2′– C-5′), 62.3, 62.2 (2 × C-6′), 8.8, 8.7 (2 × 5 × Cp*-*C*H_3_). ESI-HRMS positive mode (*m/z*): calcd for C_23_H_31_ClN_4_O_5_Ir^+^ [M-PF_6_]^+^ 671.1599. Found: [M-PF_6_]^+^ 671.1599.

#### 5.1.25. Complex **Rh-3**

Complex **Rh-3** was prepared from complex **Rh-dimer** (10.0 mg, 0.0162 mmol), compound **L-3** (23.5 mg, 0.0324 mmol, 2.0 eq.) and TlPF_6_ (11.3 mg, 0.0323 mmol), according to general method I. After filtration and removal of the solvent, the residue was dissolved in CHCl_3_ (3 mL) and Et_2_O (12 mL) was added. The precipitated product was filtered off, then washed with a solvent mixture of CHCl_3_-Et_2_O = 1:2 (4 mL) to give 32.6 mg (88%) orange powder. R_f_: 0.62 (95:5 CHCl_3_-MeOH). Diastereomeric ratio: 3:1. ^1^H NMR (400 MHz, CDCl_3_) δ (ppm): 8.96 (d, *J* = 5.4 Hz, minor Py-H-6), 8.76 (d, *J* = 5.4 Hz, major Py-H-6), 8.22–7.79, 7.59–7.28 (m, minor and major Ar, Py-H-3–Py-H-5), 6.33 (pt, *J* = 10.0, 9.7 Hz, major H-2′ or H-3′ or H-4′), 6.18 (pt, *J* = 9.6, 9.6 Hz, minor H-2′ or H-3′ or H-4′), 6.04 (pt, *J* = 9.5, 9.4 Hz, major H-2′ or H-3′ or H-4′), 5.85 (2 pt, *J* = 9.7, 9.7 Hz in each, minor and major H-2′ or H-3′ or H-4′), 5.74 (pt, *J* = 9.9, 9.7 Hz, minor H-2′ or H-3′ or H-4′), 5.48 (d, *J* = 10.1 Hz, major H-1′), 5.37 (d, *J* = 10.0 Hz, minor H-1′), 4.73–4.66 (m, minor and major H-6′a), 4.57–4.51 (m, minor and major H-6′b), 4.48–4.40 (m, minor and major H-5′), 1.61 (s, minor Cp*-C*H*_3_), 1.52 (s, major Cp*-C*H*_3_); ^13^C NMR (100 MHz, CDCl_3_) δ (ppm): 166.3, 166.2, 165.8, 165.7, 165.3, 165.2, 165.1, 165.0, 164.9 (minor and major C=O, OD-C-2, OD-C-5), 153.6 (minor Py-C-6), 152.1 (major Py-C-6), 140.6 (minor Py-C-4), 140.3 (major Py-C-4), 140.1 (major Py-C-2), 139.0 (minor Py-C-2), 134.6, 133.9, 133.8 (2), 133.7, 133.5, 133.4, 133.3, 131.4–127.8 (minor and major Ar, Py-C-5), 125.5 (major Py-C-3), 125.2 (minor Py-C-3), 98.1, 98.0 (minor Cp*), 97.8, 97.7 (major Cp*), 77.7, 72.8, 71.9, 71.3, 68.8 (minor C-1′–C-5′), 77.2, 74.2, 71.2, 69.1, 68.7 (major C-1′–C-5′), 63.0 (minor C-6′), 62.5 (major C-6′), 9.1 (minor Cp*-*C*H_3_), 8.8 (major Cp*-*C*H_3_). ESI-HRMS positive mode (*m/z*): calcd for C_51_H_46_ClN_3_O_10_Rh^+^ [M-PF_6_]^+^ 998.1921. Found: [M-PF_6_]^+^ 998.1905.

#### 5.1.26. Complex **Rh-4**

Complex **Rh-4** was prepared from complex **Rh-dimer** (20.0 mg, 0.0324 mmol), compound **L-4** (47.0 mg, 0.0648 mmol, 2.0 eq.) and TlPF_6_ (22.6 mg, 0.0647 mmol) according to general method I. After filtration and removal of the solvent, the residue was dissolved in CHCl_3_ (6 mL) and Et_2_O (12 mL) was added. The precipitated product was filtered off, then washed with a solvent mixture of CHCl_3_-Et_2_O = 1:2 (2 mL) to give 63.0 mg (85%) orange powder. R_f_: 0.29 (95:5 CHCl_3_-MeOH). Diastereomeric ratio: 9:2. ^1^H NMR (400 MHz, CDCl_3_) δ (ppm): 8.95 (d, *J* = 5.4 Hz, minor Py-H-6), 8.81 (d, *J* = 5.3 Hz, major Py-H-6), 8.22–7.24 (m, minor and major Ar, Py-H-3–Py-H-5), 6.55 (pt, *J* = 10.2, 10.1 Hz, major H-2′), 6.19 (d, *J* = 3.4 Hz, major H-4′), 6.16 (d, *J* = 3.3 Hz, minor H-4′), 6.02 (pt, *J* = 10.1, 9.7 Hz, minor H-2′), 5.94 (dd, *J* = 10.1, 3.3 Hz, minor H-3′), 5.76 (dd, *J* = 10.1, 3.4 Hz, major H-3′), 5.47 (d, *J* = 10.2 Hz, major H-1′), 5.40 (d, *J* = 9.7 Hz, minor H-1′), 4.71–4.40 (m, minor and major H-5′, H-6′a, H-6′b), 1.61 (s, minor Cp*-C*H*_3_), 1.52 (s, major Cp*-C*H*_3_); ^13^C NMR (100 MHz, CDCl_3_) δ (ppm): 166.2, 165.8, 165.6, 165.4 (2), 164.9 (minor C=O, OD-C-2, OD-C-5), 166.1, 165.7, 165.5, 165.3, 165.2, 165.0 (major C=O, OD-C-2, OD-C-5), 153.5 (minor Py-C-6), 152.2 (major Py-C-6), 140.6 (minor Py-C-4), 140.4 (major Py-C-4), 140.1 (major Py-C-2), 139.2 (minor Py-C-2), 134.5, 134.2, 133.8 (2), 133.6, 133.5 (2), 131.2–128.1 (minor and major Ar, Py-C-5), 125.5 (major Py-C-3), 125.2 (minor Py-C-3), 98.1, 98.0 (minor Cp*), 97.8, 97.7 (major Cp*), 76.6, 72.2, 71.5, 68.5, 68.3 (minor C-1′–C-5′), 76.1, 72.8, 71.4, 68.3, 66.2 (major C-1′–C-5′), 62.4 (minor C-6′), 62.1 (major C-6′), 9.0 (minor Cp*-*C*H_3_), 8.8 (major Cp*-*C*H_3_). ESI-HRMS positive mode (*m/z*): calcd for C_51_H_46_ClN_3_O_10_Rh^+^ [M-PF_6_]^+^ 998.1921. Found: [M-PF_6_]^+^ 998.1920.

#### 5.1.27. Complex **Rh-5**

Complex **Rh-5** was prepared from complex **Rh-dimer** (10.0 mg, 0.0162 mmol), compound **L-5** (19.1 mg, 0.0323 mmol, 2.0 eq.) and TlPF_6_ (11.3 mg, 0.0323 mmol) according to general method I. After filtration and removal of the solvent, the residue was dissolved in CHCl_3_ (3 mL) and Et_2_O (12 mL) was added. The precipitated product was filtered off, then washed with a solvent mixture of CHCl_3_-Et_2_O = 1:1 (2 mL) to give 28.3 mg (87%) orange powder. R_f_: 0.34 (95:5 CHCl_3_-MeOH). Diastereomeric ratio: 5:2. ^1^H NMR (400 MHz, CDCl_3_) δ (ppm): 8.95 (d, *J* = 5.3 Hz, minor Py-H-6), 8.80 (d, *J* = 5.3 Hz, major Py-H-6), 8.21–7.82, 7.59–7.31 (m, minor and major Ar, Py-H-3–Py-H-5), 6.22 (pt, *J* = 9.6, 9.7 Hz, major H-2′ or H-3′), 6.12 (pt, *J* = 9.3, 9.3 Hz, minor H-2′ or H-3′), 6.00 (pt, *J* = 9.4, 9.3 Hz, major H-2′ or H-3′), 5.75 (pt, *J* = 9.4, 9.3 Hz, minor H-2′ or H-3′), 5.59 (ddd, *J* = 10.7, 9.7, 5.4 Hz, minor H-4′), 5.51 (ddd, *J* = 10.7, 9.7, 5.5 Hz, major H-4′), 5.31 (d, *J* = 10.0 Hz, major H-1′), 5.25 (d, *J* = 9.4 Hz, minor H-1′), 4.64 (dd, *J* = 11.6, 5.4 Hz, minor H-5′eq), 4.56 (dd, *J* = 11.3, 5.5 Hz, major H-5′eq), 3.87 (pt, *J* = 11.3, 10.7 Hz, major H-5′ax), 3.84 (pt, *J* = 11.6, 10.7 Hz, minor H-5′ax), 1.63 (s, minor Cp*-C*H*_3_), 1.53 (s, major Cp*-C*H*_3_); ^13^C NMR (100 MHz, CDCl_3_) δ (ppm): 165.8, 165.7, 165.6 (2), 165.5 (2), 165.2, 165.1, 164.9 (2) (minor and major C=O, OD-C-2, OD-C-5), 153.5 (minor Py-C-6), 152.2 (major Py-C-6), 140.6 (minor Py-C-4), 140.4 (major Py-C-4), 140.1 (major Py-C-2), 139.2 (minor Py-C-2), 134.5, 133.9, 133.8, 133.7, 133.5, 131.2–128.0 (minor and major Ar, Py-C-5), 125.5 (major Py-C-3), 125.1 (minor Py-C-3), 98.1, 98.0 (minor Cp*), 97.8, 97.7 (major Cp*), 73.5, 71.7, 69.6, 69.0 (major C-1′–C-4′), 72.4, 72.2, 70.8, 69.2 (minor C-1′–C-4′), 67.7 (2) (minor and major C-5′), 9.1 (minor Cp*-*C*H_3_), 8.8 (major Cp*-*C*H_3_). ESI-HRMS positive mode (*m/z*): calcd for C_43_H_40_ClN_3_O_8_Rh^+^ [M-PF_6_]^+^ 864.1553. Found: [M-PF_6_]^+^ 864.1552.

#### 5.1.28. Complex **Rh-6**

Complex **Rh-6** was prepared from complex **Rh-dimer** (20.0 mg, 0.0324 mmol), compound **L-6** (46.9 mg, 0.0647 mmol, 2.0 eq.) and TlPF_6_ (22.6 mg, 0.0647 mmol) according to general method I. After filtration and removal of the solvent, the residue was dissolved in CHCl_3_ (6 mL) and Et_2_O (12 mL) was added. The precipitated product was filtered off, then washed with a solvent mixture of CHCl_3_-Et_2_O = 1:1 (2 mL) to give 67.5 mg (91%) orange powder. R_f_: 0.33 (9:1 CHCl_3_-MeOH). Diastereomeric ratio: 2:1. ^1^H NMR (400 MHz, CDCl_3_) δ (ppm): 9.06 (s, minor Tria-H-5), 9.03 (s, major Tria-H-5), 8.67 (1 signal, minor and major Py-H-6), 8.04–7.82, 7.60–7.22 (m, minor and major Ar, Py-H-3–Py-H-5), 6.53–6.46, 6.25–6.11, 5.99–5.90 (m, minor and major H-1′–H-4′), 4.77–4.52 (m, minor and major H-5′, H-6′a, H-6′b), 1.62 (s, major Cp*-C*H*_3_), 1.55 (s, minor Cp*-C*H*_3_); ^13^C NMR (100 MHz, CDCl_3_) δ (ppm): 166.3, 165.6, 165.2, 164.6 (minor C=O), 166.2, 165.5, 165.2, 164.7 (major C=O), 151.5 (2) (minor and major Py-C-6), 146.9, 146.8, 146.2 (2) (minor and major Tria-C-4, Py-C-2), 140.4 (minor Py-C-4), 140.2 (major Py-C-4), 134.3, 134.1, 133.9, 133.8, 133.7, 133.5, 133.4, 133.3, 130.2–128.5, 127.9, 127.8, 127.5, 127.4 (minor and major Ar, Py-C-5, Tria-C-5), 122.9 (minor Py-C-3), 122.8 (major Py-C-3), 97.5 (minor Cp*), 97.4 (major Cp*), 87.3 (minor C-1′), 86.8 (major C-1′), 76.1, 72.8, 71.6, 68.7 (minor C-2′–C-5′), 75.7, 73.7, 70.3, 68.6 (major C-2′–C-5′), 62.9 (minor C-6′), 62.7 (major C-6′), 9.0 (minor and major Cp*-*C*H_3_). ESI-HRMS positive mode (*m/z*): calcd for C_51_H_47_ClN_4_O_9_Rh^+^ [M-PF_6_]^+^ 997.2081. Found: [M-PF_6_]^+^ 997.2068.

### 5.2. X-ray Crystallography

X-ray-quality crystals were grown by slow evaporation of the mother liquors (**Ru-1** from CHCl_3_, **Ir-2** from MeOH). A crystal of good quality in polarized light was fixed under a microscope onto a Mitegen loop, using high-density oil. Diffraction intensity data were collected at room temperature (295–300 K) using a Bruker-D8 Venture diffractometer (Bruker AXS GmbH, Karlsruhe, Germany), equipped with INCOATEC IμS 3.0 (Incoatec GmbH, Geesthacht, Germany) dual (Cu and Mo) sealed tube micro sources and a Photon II Charge-Integrating Pixel Array detector (Bruker AXS GmbH, Karlsruhe, Germany) under Mo-Kα (λ = 0.71073 Å) radiation. High-multiplicity data collection and integration were performed using APEX3 (version 2017.3–0, Bruker AXS Inc., 2017, Madison, WI, USA) software. Data reduction and multi-scan absorption correction were performed using SAINT (version 8.38A, Bruker AXS Inc., 2017, Madison, WI, USA). The structure was solved using direct methods and refined on F^2^, using the SHELXL program [67] incorporated into the APEX3 suite. Refinement was performed anisotropically for all nonhydrogen atoms. Hydrogen atoms were placed into geometric positions, except for the protons of alcoholic OHs in Ir-2 that could be found at the difference electron density map, where O-H distance should be constrained. The CIF file was manually edited using Publcif software [68], while graphics were prepared using the Mercury program [69] (Table 6).

### 5.3. Determination of the Distribution Coefficients (logD)

Before starting the experiments, *n*-octanol was saturated with an aqueous PBS solution (pH = 7.40) and vice versa. A solution of the appropriate complex (approximately 0.2–0.3 mg) in a mixture of 2.50 mL pre-saturated *n*-octanol and 2.50 mL pre-saturated PBS buffer was made, and the resulting mixture was then vigorously stirred for 3 days at rt. Based on our previous NMR stability experiments [30], this time is long enough to reach equilibrium among the various ionic complex species. Due to the lipophilic/hydrophilic features of the complexes, those with benzoyl protection could primarily be found in the *n*-octanol, while the complexes with *O*-peracetylated and *O*-deprotected ligands were in the aqueous PBS phase. The corresponding separated solution was centrifuged (ScanSpeed 406G instrument, 4000 RPM for 5 min) and the absorption of the ”stock solution” was determined (VWR UV-1600PC Spectrophotometer, 270–420 nm). Then, 2.00 mL of the stock solution was vigorously stirred with 16.00 mL of pre-saturated *n*-octanol or PBS solution. After one day had passed, the phases were separated, centrifuged, and the absorption of the solution was measured again. From the absorption difference of the stock solutions, the distribution coefficient (D) was determined using previously described formulae [71].

### 5.4. Materials

In the cell biology and biochemistry assays, all chemicals were from Sigma-Aldrich unless otherwise stated.

### 5.5. Cell culture

Cells were cultured under standard cell culture conditions, 37 °C, 5% CO_2_, humidified atmosphere.

*A2780* cells were cultured in RMPI 1640 medium, supplemented with 10% fetal calf serum, 2 mM glutamine, and 1% penicillin-streptomycin.

*ID8* cells were cultured in a high-glucose DMEM (4.5 g/L glucose) medium, supplemented with 4% fetal calf serum, 2 mM glutamine, 1% penicillin-streptomycin, and 1% ITS supplement (I3146).

*Capan2* cells were maintained in MEM, 10% fetal bovine serum, 1% penicillin/streptomycin, and 2 mM glutamine.

*Human primary dermal fibroblasts* were cultured in low glucose DMEM (1 g/L glucose) medium supplemented with 20% fetal calf serum, 2 mM glutamine, and 1% penicillin-streptomycin.

*L428* cells were maintained in RPMI 1640 medium, supplemented with 10% fetal calf serum, 2 mM glutamine, and 1% penicillin-streptomycin.

*Saos* cells were maintained in DMEM (4.5 g/L glucose) medium, supplemented with 10% fetal calf serum, 2 mM glutamine, and 1% penicillin-streptomycin.

### 5.6. Methylthiazolyldiphenyl-Tetrazolium Bromide (MTT) Reduction Assay

The MTT reduction assay measures the activity of mitochondrial complex I and can be used to detect apoptosis and necrosis [40,41]. The assay was performed similarly to the method used in [72]. Briefly, cells were plated in 96-well plates the day before the assay. Cells were treated with the compounds for 4 h, then MTT was added at 0.5 mg/mL final concentration and the cells were incubated at 37 °C in a cell incubator. The culture media were removed and the reduced MTT dye was dissolved in dimethyl-sulfoxide (DMSO) and plates were measured in a plate photometer (Thermo Scientific Multiscan GO spectrophotometer, Waltham, MA, USA) at 540 nm. On each plate, wells were designed to contain untreated/vehicle-treated cells. In the calculations, the readings for these wells were considered as 1 and all readings were expressed relative to these values.

### 5.7. Sulforhodamine B (SRB) Binding Assay

The SRB assay measures total protein content that is proportional to cell number, hence can be used to assess cell proliferation or long-term cytostasis [42]. The assay was performed similarly to the method used in [73]. Cells were seeded in 96-well plates the day before the assay. Cells were treated with the compounds for 48 h, with the exception of L428, where 96 h of treatment was applied. Cells were fixed with 10% trichloroacetic acid (TCA). Fixed cells were washed in distilled water 3 times, followed by staining with SRB (0.4 m/V% dissolved in 1% acetic acid) for 10 min. The stained cells were washed in 1% acetic acid 5 times; the acetic acid was removed, and the cells were left to dry. Protein-bound SRB was released by adding 100 µL 10 mM Tris base. Plates were measured in a plate photometer (Thermo Scientific Multiscan GO spectrophotometer, Waltham, MA, USA) at 540 nm. On each plate, wells were designed to contain untreated cells. In calculations, the readings for these wells were considered as 1 and all readings were expressed relative to these values.

### 5.8. Cell Death

The proportion of dead cells was assessed using the Annexin V–propidium iodide assay and was measured using flow cytometry with a BD FacsCalibur (BD Biosciences, Franklin Lakes, NJ, USA) instrument and the FITC Annexin V/Dead Cell Apoptosis kit (Life Technologies, Eugene, OR, USA), according to the manufacturer’s instructions, in a process similar to that in [43]. Quadrants were set, based on the FITC and PI values observed for the vehicle-treated cells.

### 5.9. Statistical Analysis

Statistical analysis was performed using the 8.0.1 version of Graphpad Prism. Values were tested for normal distribution, using the d’Agostino and Pearson normality test. When necessary, values were log-normalized or were normalized using the Box-Cox normalization method [74] or using a two-step transformation, as indicated in the figure captions. The following statistical test, post hoc test and the level of significance are indicated in the figure captions. Nonlinear regression was performed using the built-in “[Inhibitor] vs. Response—Variable slope (four parameters), least-square fit” utility of Graphpad, yielding the IC_50_ and Hill slope values.

## Figures and Tables

**Table 1:** Synthesis of [(η^6^-*p*-cym)Os^II^(N-N)Cl]PF_6_ complexes containing *O*-perbenzoylated *C*- and *N*-glycosyl azoles.

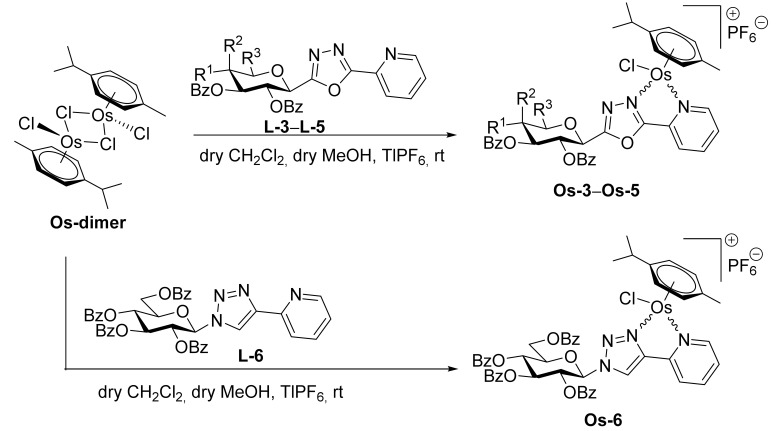						
Heterocyclic Ligand	R^1^	R^2^	R^3^	Product	Yield (%)	Diastereomeric Ratio
**L-3**	OBz	H	CH_2_OBz	**Os-3**	71	7:6
**L-4**	H	OBz	CH_2_OBz	**Os-4**	78	3:2
**L-5**	OBz	H	H	**Os-5**	84	7:5
**L-6**	-	-	-	**Os-6**	85	1:1

**Table 2:** Synthesis of [(η^5^-Cp*)Ir^III^(N-N))Cl]PF_6_ complexes containing *C*- and *N*-glycosyl azoles.

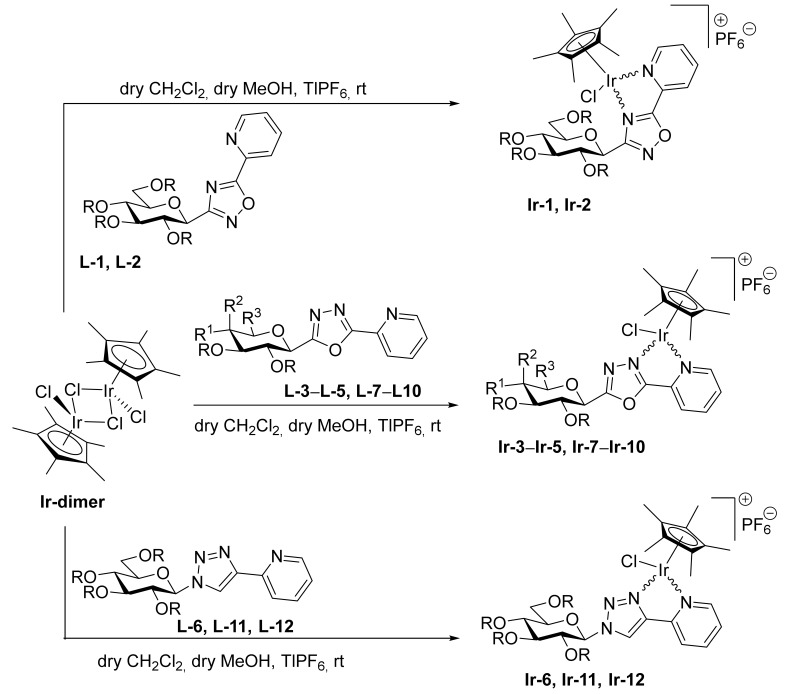							
Heterocyclic Ligand	R	R^1^	R^2^	R^3^	Product	Yield (%)	Diastereomeric Ratio
**L-1**	Bz	-	-	-	**Ir-1**	70	6:1
**L-2**	H	-	-	-	**Ir-2**	86	1:1
**L-3**	Bz	OBz	H	CH_2_OBz	**Ir-3**	80	5:2
**L-4**	Bz	H	OBz	CH_2_OBz	**Ir-4**	77	2:1
**L-5**	Bz	OBz	H	H	**Ir-5**	72	3:1
**L-6**	Bz	-	-	-	**Ir-6**	84	2:1
**L-7**	Ac	H	OAc	CH_2_OAc	**Ir-7**	81	5:2
**L-8**	H	OH	H	CH_2_OH	**Ir-8**	79	1:1
**L-9**	H	H	OH	CH_2_OH	**Ir-9**	61	1:1
**L-10**	H	OH	H	H	**Ir-10**	74	1:1
**L-11**	Ac	-	-	-	**Ir-11**	81	6:5
**L-12**	H	-	-	-	**Ir-12**	56	1:1

**Table 3:** Synthesis of [(η^5^-Cp*)Rh^III^(N-N))Cl]PF_6_ complexes containing *O*-perbenzoylated *C*- and *N*-glycosyl azoles.

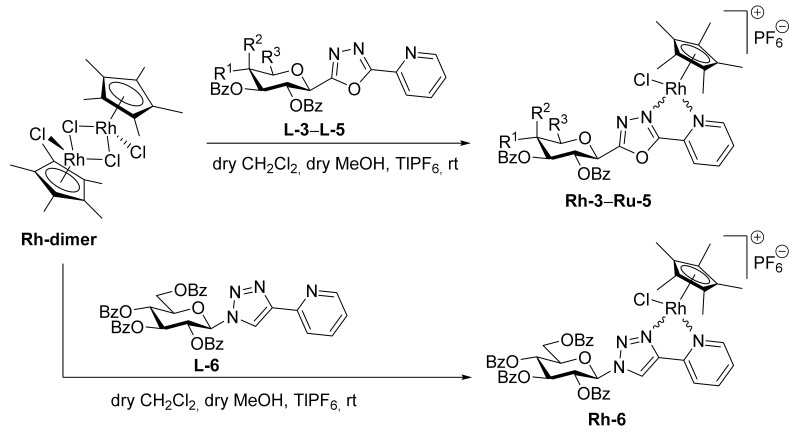						
Heterocyclic Ligand	R^1^	R^2^	R^3^	Product	Yield (%)	Diastereomeric Ratio
**L-3**	OBz	H	CH_2_OBz	**Rh-3**	88	3:1
**L-4**	H	OBz	CH_2_OBz	**Rh-4**	85	9:2
**L-5**	OBz	H	H	**Rh-5**	87	5:2
**L-6**	-	-	-	**Rh-6**	91	2:1

**Table 4:** Maximum inhibition in MTT assays.

Compound	Maximum Inhibition (%)			Compound	Maximum Inhibition (%)		
	A2780	ID8	Fibroblast		A2780	ID8	Fibroblast
**L-1**	ND			**Ir-7**	ND		
**L-2**	ND			**Ir-8**	ND		
**Ru-1**	ND			**Ir-9**	ND		
**Ru-2**	ND			**Ir-10**	ND		
**Os-3**	37.42	35.24	ND	**Ir-11**	ND		
**Os-4**	26.67	22.91	ND	**Ir-12**	ND		
**Os-5**	31.38	59.51	ND	**Rh-3**	ND	ND	ND
**Os-6**	21.38	64.44	ND	**Rh-4**	ND	ND	ND
**Ir-1**	ND			**Rh-5**	ND	21.87	ND
**Ir-2**	ND			**Rh-6**	ND	28.28	ND
**Ir-3**	ND	26.11	ND	**Ir-dimer**	ND	ND	ND
**Ir-4**	ND	ND	31.19	**Rh-dimer**	ND	ND	ND
**Ir-5**	ND	ND	ND	**Os-dimer**	ND	ND	ND
**Ir-6**	43.07	62.71	ND				

ND—not detected or cannot be calculated.

**Table 5:** Maximum inhibition, IC_50_, Hill coefficient values from SRB assays, and the logD value.

	A2780			ID8			Capan2			Saos			L428			Fibroblast			LogD
	Max. Inh. (%)	IC_50_ (µM)	Hill (µM)	Max. Inh. (%)	IC_50_ (µM)	Hill (µM)	Max. Inh. (%)	IC_50_ (µM)	Hill (µM)	Max. Inh. (%)	IC_50_ (µM)	Hill (µM)	Max. Inh. (%)	IC_50_ (µM)	Hill (µM)	Max. Inh. (%)	IC_50_ (µM)	Hill (µM)	
**L-1**	ND	ND	ND																
**L-2**	ND	ND	ND																
**Ru-1**	ND	ND	ND																2.79
**Ru-2**	ND	ND	ND																−0.96
**Os-3**	100	2.52	1.82	100	5.94	2.39	100	8.80	2.30	65	ND	ND	100	ND	ND	ND	ND	ND	3.32
**Os-4**	100	3.23	2.52	100	6.95	3.69	100	6.20	1.85	100	8.78	ND	100	ND	ND	ND	ND	ND	2.87
**Os-5**	100	2.81	1.85	100	1.96	2.90	100	8.89	2.26	100	8.52	ND	100	ND	ND	ND	ND	ND	2.36
**Os-6**	100	0.73	3.01	100	1.96	3.14	100	1.71	1.33	100	3.60	ND	100	ND	ND	ND	ND	ND	2.33
**Ir-1**	35.13	ND	ND																2.87
**Ir-2**	36.38	ND	ND																−1.15
**Ir-3**	69.25	ND	ND	67.93	ND	ND	27.62	ND	ND							ND	ND	ND	1.46
**Ir-4**	32.45	ND	ND	ND	ND	ND	ND	ND	ND							ND	ND	ND	1.46
**Ir-5**	45.45	ND	ND	53.15	ND	ND	ND	ND	ND							ND	ND	ND	1.80
**Ir-6**	100	1.64	2.08	100	0.98	2.46	100	4.15	1.58	100	2.71	ND	100	ND	ND	ND	ND	ND	2.36
**Ir-7**	26.91	ND	ND																−0.75
**Ir-8**	ND	ND	ND																−1.60
**Ir-9**	21.29	ND	ND																−1.47
**Ir-10**	23.12	ND	ND																−1.56
**Ir-11**	20.26	ND	ND																−1.31
**Ir-12**	19.11	ND	ND																−2.08
**Rh-3**	39.86	ND	ND	ND	ND	ND	ND	ND	ND							ND	ND	ND	1.60
**Rh-4**	35.30	ND	ND	ND	ND	ND	ND	ND	ND							ND	ND	ND	1.64
**Rh-5**	51.28	ND	ND	75.25	ND	ND	ND	ND	ND							ND	ND	ND	1.8
**Rh-6**	59	25.28	0.71	43.70	ND	ND	ND	ND	ND							ND	ND	ND	1.85
**Ir-dimer**	ND	ND	ND	ND	ND	ND	ND	ND	ND	ND	ND	ND	ND	ND	ND	ND	ND	ND	
**Rh-dimer**	ND	ND	ND	ND	ND	ND	ND	ND	ND	ND	ND	ND	ND	ND	ND	ND	ND	ND	
**Os-dimer**	ND	ND	ND	ND	ND	ND	ND	ND	ND	ND	ND	ND	ND	ND	ND	ND	ND	ND	
Ru-3 *	100	6.19	2.62	100	5.66	3.04	100	3.27	1.81							ND	ND	ND	2.41
Ru-4 *	100	4.27	3.5	100	7.94	3.31	100	3.82	1.66							ND	ND	ND	2.44
Ru-5 *	100	8.54	3.74	100	6.76	3.59	100	4.97	1.66							80	24.63	3.52	2.04
Ru-6 *	100	0.87	1.95	100	2.15	2.5	100	2.18	2.43							ND	ND	ND	2.85

ND—not detected, cannot be calculated; Max. inh.—Max. inhibition; *—data previously published in [30].

**Table 6:** Experimental details of X-ray structure determination.

	Ru-1	Ir-2
Crystal data		
Chemical formula	2(C_51_H_45_ClN_3_O_10_Ru)·2(F_6_P)·5(CHCl_3_)	C_23_H_30_ClIrN_3_O_6_·F_6_P
*M*_r_	2879.61	817.12
Crystal system, space group	Monoclinic, *P*2_1_	Orthorhombic, *P*2_1_2_1_2_1_
Temperature (K)	150	295
*a*, *b*, *c* (Å)	14.531 (3), 24.879 (4), 16.855 (4)	9.3075 (8), 12.1090 (8), 24.6823 (18)
α,β,γ (°)	90, 93.610 (5), 90	90, 90, 90
*V* (Å^3^)	6081 (2)	2781.8 (4)
*Z*	2	4
Radiation type	Mo *Kα*	
µ (mm^−1^)	0.73	5.04
Crystal size (mm)	0.19 × 0.18 × 0.05	0.51 × 0.10 × 0.08

Data collection		
Diffractometer	Bruker D8 VENTURE	
Absorption correction	Multi-scan *SADABS2016*/2—Bruker AXS area detector scaling and absorption correction	
*T*_min_, *T*_max_	0.89, 0.97	0.53, 0.70
No. of measured, independent and observed [*I* > 2σ(*I*)] reflections	71,476, 23,083, 12,753	20076, 5675, 4789
*R*_int_	0.115	0.111
(sin θ/λ)_max_ (Å^−1^)	0.612	0.626

Refinement		
*R*[*F*^2^ > 2σ (*F*^2^)], *wR*(*F*^2^), *S*	0.090, 0.175, 1.32	0.059, 0.104, 1.07
No. of reflections	23083	5675
No. of parameters	1424	387
No. of restraints	10	34
H-atom treatment	H-atom parameters constrained	H atoms treated by a mixture of independent and constrained refinement
	*w* = 1/[σ^2^(*F*_o_^2^) + (0.0123*P*)^2^ + 11.6544*P*] where *P* = (*F*_o_^2^ + 2*F*_c_^2^)/3	*w* = 1/[σ^2^(*F*_o_^2^) + (0.0285*P*)^2^] where *P* = (*F*_o_^2^ + 2*F*_c_^2^)/3
Δ〉_max_, Δ〉_min_ (e Å^−3^)	1.55, −0.85	1.81, −3.07
Absolute structure	Flack x determined using 3978 quotients [(I+) − (I−)]/[(I+) + (I−)] [70]	Flack x determined using 1573 quotients [(I+) − (I−)]/[(I+) + (I−)] [70]
Absolute structure parameter	−0.04 (2)	0.017 (11)

## Data Availability

Primary biological data for this manuscript is available at https://figshare.com/s/28d14597d5041f08eb08 (accessed on 6 January 2022) (doi:10.6084/m9.figshare.16929364). Deposition Numbers 2129695 (for **Ru-1**) and 2129694 (for **Ir-2**) contain the supplementary crystallographic data for this paper. These data are provided free of charge by the joint Cambridge Crystallographic Data Center and Fachinformationszentrum Karlsruhe http://www.ccdc.cam.ac.uk/structures (accessed on 6 January 2022).

## Supplementary Materials

The following are available online at https://www.mdpi.com/article/10.3390/ijms23020813/s1.
