# Supplementary material for: Reactive Oxygen Species Production Is Responsible for Antineoplastic Activity of Osmium, Ruthenium, Iridium and Rhodium Half-Sandwich Type Complexes with Bidentate Glycosyl Heterocyclic Ligands in Various Cancer Cell Models
Source: Int J Mol Sci. 2022 Jan 12;23(2):813. doi: 10.3390/ijms23020813 (PMC8776094; doi:10.3390/ijms23020813)
Supplement: Supplementary file 1 [file ijms-23-00813-s001.zip › Kacsiretal_checkcif.pdf]

checkCIF () running

Checking for embedded fcf data in CIF ...
Found embedded fcf data in CIF. Extracting fcf data from uploaded CIF, please wait.................

checkCIF/PLATON (full publication check)

You have not supplied any structure factors. As a result the full set of tests cannot be run.

THIS REPORT IS FOR GUIDANCE ONLY. IF USED AS PART OF A REVIEW PROCEDURE FOR PUBLICATION, IT SHOULD NOT REPLACE THE EXPERTISE OF AN EXPERIENCED CRYSTALLOGRAPHIC REFEREE.You have not supplied any structure factors. As a result the full set of tests cannot be run.

No syntax errors found. CIF dictionary
Please wait while processing .... Interpreting this report
Structure factor report

Datablock: Ir_2

Bond precision:	C-C = 0.0219 A		Wavelength=0.71073
Cell:	a=9.3075(8)	b=12.1090(8)	c=24.6823(18)
	alpha=90	beta=90	gamma=90
Temperature:	295 K		

	Calculated	Reported
Volume	2781.8(4)	2781.8(4)
Space group	P 21 21 21	P 21 21 21
Hall group	P 2ac 2ab	P 2ac 2ab
Moiety formula	C23 H30 Cl Ir N3 O6, F6 P	C23 H30 Cl Ir N3 O6, F6 P
Sum formula	C23 H30 Cl F6 Ir N3 O6 P	C23 H30 Cl F6 Ir N3 O6 P
Mr	817.14	817.12
Dx,g cm-3	1.951	1.951
Z	4	4
Mu (mm-1)	5.038	5.038
F000	1600.0	1600.0
F000'	1596.25	
h,k,lmax	11,15,30	11,15,30
Nref	5711[3235]	5675
Tmin,Tmax	0.542,0.678	0.530,0.700
Tmin'	0.076	

Correction method= # Reported T Limits: Tmin=0.530 Tmax=0.700 AbsCorr = MULTI-SCAN

Data completeness= 1.75/0.99 Theta(max)= 26.420

R(reflections)= 0.0588(4789) wR2(reflections)= 0.1040(5675)

S = 1.070 Npar= 387

The following ALERTS were generated. Each ALERT has the format
test-name_ALERT_alert-type_alert-level.
Click on the hyperlinks for more details of the test.

Alert level A

PLAT213_ALERT_2_A Atom C54 has ADP max/min Ratio ..... 6.9 oblate

Author Response: Presence of very heavy element (Ir) may caused the problem. Chemical evidence shows that the structure is correct.

Alert level B

PLAT213_ALERT_2_B Atom C6' has ADP max/min Ratio ..... 4.1 oblate

Author Response: Presence of very heavy element (Ir) may caused the problem. Chemical evidence shows that the structure is correct.

PLAT342_ALERT_3_B Low Bond Precision on C-C Bonds .... 0.0219 Ang.

Author Response: resence of very heavy element (Ir) may caused the problem. Chemical evidence shows that the structure is correct.

PLAT369_ALERT_2_B Long C(sp2)-C(sp2) Bond C2' - C5 . 1.57 Ang.

Author Response: resence of very heavy element (Ir) may caused the problem. Chemical evidence shows that the structure is correct.

PLAT417_ALERT_2_B Short Inter D-H..H-D H4O ..H6O . 1.80 Ang.
-1/2+x,3/2-y,1-z = 2_466 Check

Author Response: Orientation of OH is uncertain. resence of very heavy element (Ir) may caused the problem. Chemical evidence shows that the structure is correct.

PLAT420_ALERT_2_B D-H Bond Without Acceptor O2" --H2O . Please Check

Author Response: Orientation of OH is uncertain. Presence of very heavy element (Ir) may caused the problem. Chemical evidence shows that the structure is correct.

PLAT420_ALERT_2_B D-H Bond Without Acceptor O3" --H3O . Please Check

Author Response: Orientation of OH is uncertain. Presence of very heavy element (Ir) may caused the problem. Chemical evidence shows that the structure is correct.

PLAT420_ALERT_2_B D-H Bond Without Acceptor O6" --H6O . Please Check

Author Response: Orientation of OH is uncertain. Presence of very heavy element (Ir) may caused the problem. Chemical evidence shows that the structure is correct.

PLAT728_ALERT_1_B D-H..A Calc 167.00, Rep 170(20) Dev... 3.00 Degree
O4" -H4O -O6" 1_555 1_555 2_466 # 3 Check

Author Response: Orientation of OH is uncertain. Presence of very heavy element (Ir) may caused the problem. Chemical evidence shows that the structure is correct.

Alert level C

PLAT213_ALERT_2_C Atom C2' has ADP max/min Ratio ..... 3.6 prolat

Author Response: Presence of very heavy element (Ir) may caused the problem. Chemical evidence shows that the structure is correct.

PLAT213_ALERT_2_C Atom C5" has ADP max/min Ratio ..... 3.1 oblate

Author Response: Presence of very heavy element (Ir) may caused the problem. Chemical evidence shows that the structure is correct.

PLAT234_ALERT_4_C Large Hirshfeld Difference O1 --C5 . 0.18 Ang.

And 3 other PLAT234 Alerts

PLAT234_ALERT_4_C Large Hirshfeld Difference N1' --C2' . 0.16 Ang.
PLAT234_ALERT_4_C Large Hirshfeld Difference N1' --C6' . 0.19 Ang.
PLAT234_ALERT_4_C Large Hirshfeld Difference C2' --C5 . 0.18 Ang.
PLAT245_ALERT_2_C U(iso) H4O Smaller than U(eq) O4" by 0.017 Ang**2
PLAT250_ALERT_2_C Large U3/U1 Ratio for Average U(i,j) Tensor .... 2.3 Note
PLAT260_ALERT_2_C Large Average Ueq of Residue Including P1 0.180 Check
PLAT480_ALERT_4_C Long H...A H-Bond Reported H2O ..F14 . 2.58 Ang.

Alert level G

PLAT002_ALERT_2_G Number of Distance or Angle Restraints on AtSite 8 Note
PLAT003_ALERT_2_G Number of Uiso or Uij Restrained non-H Atoms ... 5 Report
PLAT172_ALERT_4_G The CIF-Embedded .res File Contains DFIX Records 1 Report
PLAT178_ALERT_4_G The CIF-Embedded .res File Contains SIMU Records 1 Report
PLAT244_ALERT_4_G Low 'Solvent' Ueq as Compared to Neighbors of P1 Check
PLAT380_ALERT_4_G Incorrectly? Oriented X(sp2)-Methyl Moiety ..... C58 Check
PLAT395_ALERT_2_G Deviating X-O-Y Angle From 120 for O1 100.6 Degree
PLAT432_ALERT_2_G Short Inter X...Y Contact Cl1 ..C5 3.21 Ang.
1-x,1/2+y,3/2-z = 3_656 Check
PLAT720_ALERT_4_G Number of Unusual/Non-Standard Labels .... 2 Note
PLAT773_ALERT_2_G Check long C-C Bond in CIF: C3 --C5 2.00 Ang.
PLAT779_ALERT_4_G Suspect or Irrelevant (Bond) Angle(s) in CIF ... 38.50 Deg.
N4 -C3 -C5 1_555 1_555 1_555 ...... # 20 Check
PLAT779_ALERT_4_G Suspect or Irrelevant (Bond) Angle(s) in CIF ... 44.10 Deg.
N4 -C5 -C3 1_555 1_555 1_555 ...... # 43 Check

PLAT791_ALERT_4_G Model has Chirality at C1" (Sohnke SpGr) S Verify

And 4 other PLAT791 Alerts

PLAT791_ALERT_4_G Model has Chirality at C2" (Sohnke SpGr) R Verify
PLAT791_ALERT_4_G Model has Chirality at C3" (Sohnke SpGr) R Verify
PLAT791_ALERT_4_G Model has Chirality at C4" (Sohnke SpGr) S Verify
PLAT791_ALERT_4_G Model has Chirality at C5" (Sohnke SpGr) R Verify
PLAT860_ALERT_3_G Number of Least-Squares Restraints .... 34 Note

1 **ALERT level A** = Most likely a serious problem - resolve or explain
8 **ALERT level B** = A potentially serious problem, consider carefully
10 **ALERT level C** = Check. Ensure it is not caused by an omission or oversight
18 **ALERT level G** = General information/check it is not something unexpected

1 ALERT type 1 CIF construction/syntax error, inconsistent or missing data
17 ALERT type 2 Indicator that the structure model may be wrong or deficient
2 ALERT type 3 Indicator that the structure quality may be low
17 ALERT type 4 Improvement, methodology, query or suggestion
0 ALERT type 5 Informative message, check

Datablock: Ru_1

Bond precision: C-C = 0.0306 A Wavelength=0.71073

Cell:	a=14.531(3)	b=24.879(4)	c=16.855(4)
	alpha=90	beta=93.610(5)	gamma=90

Temperature: 150 K

	Calculated	Reported
Volume	6081(2)	6081(2)
Space group	P 21	P 1 21 1
Hall group	P 2yb	P 2yb
Moiety formula	2(C51 H45 Cl N3 O10 Ru), 2(F6 P), 5(C H Cl3)	2(C51 H45 Cl N3 O10 Ru), 2(F6 P), 5(C H Cl3)
Sum formula	C107 H95 Cl17 F12 N6 O20 P2 Ru2	C107 H95 Cl17 F12 N6 O20 P2 Ru2
Mr	2879.62	2879.61
Dx,g cm-3	1.573	1.573
Z	2	2
Mu (mm-1)	0.733	0.733
F000	2908.0	2908.0
F000'	2909.76	
h,k,lmax	17,30,20	17,30,20
Nref	23396[11985]	23083
Tmin,Tmax	0.869,0.965	0.890,0.970
Tmin'	0.869	

Correction method= # Reported T Limits: Tmin=0.890 Tmax=0.970 AbsCorr = MULTI-SCAN

Data completeness= 1.93/0.99 Theta(max)= 25.800

R(reflections)= 0.0898(12753) wR2(reflections)= 0.1753(23083)

S = 1.323 Npar= 1424

The following ALERTS were generated. Each ALERT has the format
test-name_ALERT_alert-type_alert-level.
Click on the hyperlinks for more details of the test.

Alert level A

PLAT213_ALERT_2_A Atom C80A has ADP max/min Ratio ..... 5.2 prolat

Author Response: Presence of very heavy element (Ru) may caused the problem. Chemical evidence shows that the structure is correct.

PLAT214_ALERT_2_A Atom C93 (Anion/Solvent) ADP max/min Ratio 7.3 prolat

Author Response: Solvent chloroform is slightly disordered. Presence of very heavy element (Ru) may caused the problem. Chemical evidence shows that the structure is correct.

PLAT234_ALERT_4_A Large Hirshfeld Difference C32A --C33A . 0.37 Ang.

Author Response: Presence of very heavy element (Ru) may caused the problem. Chemical evidence shows that the structure is correct.

Alert level B

PLAT213_ALERT_2_B Atom C30 has ADP max/min Ratio ..... 4.2 prolat

Author Response: Presence of very heavy element (Ru) may caused the problem. Chemical evidence shows that the structure is correct.

PLAT213_ALERT_2_B Atom C75A has ADP max/min Ratio ..... 4.2 prolat

Author Response: Presence of very heavy element (Ru) may caused the problem. Chemical evidence shows that the structure is correct.

PLAT213_ALERT_2_B Atom C77A has ADP max/min Ratio ..... 4.4 prolat

Author Response: Presence of very heavy element (Ru) may caused the problem. Chemical evidence shows that the structure is correct.

PLAT220_ALERT_2_B NonSolvent Resd 1 O Ueq(max)/Ueq(min) Range 6.2 Ratio

Author Response: Presence of very heavy element (Ru) may caused the problem. Chemical evidence shows that the structure is correct.

PLAT220_ALERT_2_B NonSolvent Resd 2 C Ueq(max)/Ueq(min) Range 6.2 Ratio

Author Response: Presence of very heavy element (Ru) may caused the problem. Chemical evidence shows that the structure is correct.

PLAT232_ALERT_2_B Hirshfeld Test Diff (M-X) Ru1 --C74 . 10.5 s.u.
PLAT232_ALERT_2_B Hirshfeld Test Diff (M-X) Ru2 --C74A . 10.3 s.u.
PLAT234_ALERT_4_B Large Hirshfeld Difference C23 --C24 . 0.26 Ang.

Author Response: Presence of very heavy element (Ru) may caused the problem. Chemical evidence shows that the structure is correct.

PLAT234_ALERT_4_B Large Hirshfeld Difference C24 --C25 . 0.28 Ang.

Author Response: Presence of very heavy element (Ru) may caused the problem. Chemical evidence shows that the structure is correct.

PLAT234_ALERT_4_B Large Hirshfeld Difference C42 --C43 . 0.26 Ang.

Author Response: Presence of very heavy element (Ru) may caused the problem. Chemical evidence shows that the structure is correct.

PLAT234_ALERT_4_B Large Hirshfeld Difference C43 --C44 . 0.30 Ang.

Author Response: Presence of very heavy element (Ru) may caused the problem. Chemical evidence shows that the structure is correct.

PLAT234_ALERT_4_B Large Hirshfeld Difference C74A --C75A . 0.30 Ang.

Author Response: Presence of very heavy element (Ru) may caused the problem. Chemical evidence shows that the structure is correct.

PLAT234_ALERT_4_B Large Hirshfeld Difference P2 --F23 . 0.28 Ang.

Author Response: Presence of very heavy element (Ru) may caused the problem. Chemical evidence shows that the structure is correct.

PLAT250_ALERT_2_B Large U3/U1 Ratio for Average U(i,j) Tensor .... 4.2 Note
PLAT260_ALERT_2_B Large Average Ueq of Residue Including Cl61 0.400 Check

Author Response: Solvent chloroform is slightly disordered. Chemical evidence shows that the structure is correct.

PLAT342_ALERT_3_B Low Bond Precision on C-C Bonds .... 0.03061 Ang.

Author Response: Presence of very heavy element (Ru) may caused the problem. Chemical evidence shows that the structure is correct.

PLAT413_ALERT_2_B Short Inter XH3 .. XHn H32 ..H79D . 1.99 Ang.
1-x,-1/2+y,1-z = 2_646 Check

Author Response: Presence of very heavy element (Ru) may caused the problem. Chemical evidence shows that the structure is correct.

Alert level C

PLAT213_ALERT_2_C Atom O30 has ADP max/min Ratio ..... 3.9 prolat

Author Response: Presence of very heavy element (Ru) may caused the problem. Chemical evidence shows that the structure is correct.

PLAT213_ALERT_2_C Atom C24 has ADP max/min Ratio ..... 3.3 prolat

Author Response: Presence of very heavy element (Ru) may caused the problem. Chemical evidence shows that the structure is correct.

PLAT213_ALERT_2_C Atom C73 has ADP max/min Ratio ..... 3.1 prolat

Author Response: Presence of very heavy element (Ru) may caused the problem. Chemical evidence shows that the structure is correct.

PLAT213_ALERT_2_C Atom C32A has ADP max/min Ratio ..... 3.1 prolat

Author Response: Presence of very heavy element (Ru) may caused the problem. Chemical evidence shows that the structure is correct.

PLAT213_ALERT_2_C Atom C34A has ADP max/min Ratio ..... 3.5 prolat

Author Response: Presence of very heavy element (Ru) may caused the problem. Chemical evidence shows that the structure is correct.

PLAT213_ALERT_2_C Atom C45A has ADP max/min Ratio ..... 3.2 prolat

Author Response: Presence of very heavy element (Ru) may caused the problem. Chemical evidence shows that the structure is correct.

PLAT214_ALERT_2_C Atom F23 (Anion/Solvent) ADP max/min Ratio 4.5 prolat

Author Response: Solvent chloroform is slightly disordered. Presence of very heavy element (Ru) may caused the problem. Chemical evidence shows that the structure is correct.

PLAT214_ALERT_2_C Atom Cl63 (Anion/Solvent) ADP max/min Ratio 4.8 prolat

Author Response: Solvent chloroform is slightly disordered. Presence of very heavy element (Ru) may caused the problem. Chemical evidence shows that the structure is correct.

PLAT222_ALERT_3_C NonSolvent Resd 2 H Uiso(max)/Uiso(min) Range 7.6 Ratio
PLAT230_ALERT_2_C Hirshfeld Test Diff for C73 --C74 . 5.8 s.u.
PLAT230_ALERT_2_C Hirshfeld Test Diff for C71A --C76A . 5.9 s.u.
PLAT234_ALERT_4_C Large Hirshfeld Difference O1" --N2" . 0.18 Ang.

Author Response: Presence of very heavy element (Ru) may caused the problem. Chemical evidence shows that the structure is correct.

PLAT234_ALERT_4_C Large Hirshfeld Difference C40 --C41 . 0.20 Ang.

Author Response: Presence of very heavy element (Ru) may caused the problem. Chemical evidence shows that the structure is correct.

PLAT234_ALERT_4_C Large Hirshfeld Difference C45 --C46 . 0.22 Ang.

Author Response: Presence of very heavy element (Ru) may caused the problem. Chemical evidence shows that the structure is correct.

PLAT234_ALERT_4_C Large Hirshfeld Difference C74 --C75 . 0.17 Ang.

Author Response: Presence of very heavy element (Ru) may caused the problem. Chemical evidence shows that the structure is correct.

PLAT234_ALERT_4_C Large Hirshfeld Difference O30A --C30A . 0.22 Ang.

Author Response: Presence of very heavy element (Ru) may caused the problem. Chemical evidence shows that the structure is correct.

PLAT234_ALERT_4_C Large Hirshfeld Difference C2A' --C3A' . 0.19 Ang.

Author Response: Presence of very heavy element (Ru) may caused the problem. Chemical evidence shows that the structure is correct.

PLAT234_ALERT_4_C Large Hirshfeld Difference C31A --C36A . 0.24 Ang.

Author Response: Presence of very heavy element (Ru) may caused the problem. Chemical evidence shows that the structure is correct.

PLAT234_ALERT_4_C Large Hirshfeld Difference C71A --C72A . 0.25 Ang.

Author Response: Presence of very heavy element (Ru) may caused the problem. Chemical evidence shows that the structure is correct.

PLAT234_ALERT_4_C Large Hirshfeld Difference P1 --F11 . 0.16 Ang.

Author Response: Presence of very heavy element (Ru) may caused the problem. Chemical evidence shows that the structure is correct.

PLAT234_ALERT_4_C Large Hirshfeld Difference P1 --F13 . 0.22 Ang.

Author Response: Presence of very heavy element (Ru) may caused the problem. Chemical evidence shows that the structure is correct.

PLAT241_ALERT_2_C High 'MainMol' Ueq as Compared to Neighbors of C24 Check

And 9 other PLAT241 Alerts

```
PLAT241_ALERT_2_C High   'MainMol' Ueq as Compared to Neighbors of        C43 Check
PLAT241_ALERT_2_C High   'MainMol' Ueq as Compared to Neighbors of        C63 Check
PLAT241_ALERT_2_C High   'MainMol' Ueq as Compared to Neighbors of        C74 Check
PLAT241_ALERT_2_C High   'MainMol' Ueq as Compared to Neighbors of       C33A Check
PLAT241_ALERT_2_C High   'MainMol' Ueq as Compared to Neighbors of       C35A Check
PLAT241_ALERT_2_C High   'MainMol' Ueq as Compared to Neighbors of       C43A Check
PLAT241_ALERT_2_C High   'MainMol' Ueq as Compared to Neighbors of       C45A Check
PLAT241_ALERT_2_C High   'MainMol' Ueq as Compared to Neighbors of       C71A Check
PLAT241_ALERT_2_C High   'MainMol' Ueq as Compared to Neighbors of       C75A Check
PLAT242_ALERT_2_C Low    'MainMol' Ueq as Compared to Neighbors of        Ru1 Check
```

And 10 other PLAT242 Alerts

```
PLAT242_ALERT_2_C Low    'MainMol' Ueq as Compared to Neighbors of         O3 Check
PLAT242_ALERT_2_C Low    'MainMol' Ueq as Compared to Neighbors of        C22 Check
PLAT242_ALERT_2_C Low    'MainMol' Ueq as Compared to Neighbors of        C30 Check
PLAT242_ALERT_2_C Low    'MainMol' Ueq as Compared to Neighbors of        C42 Check
PLAT242_ALERT_2_C Low    'MainMol' Ueq as Compared to Neighbors of        C78 Check
PLAT242_ALERT_2_C Low    'MainMol' Ueq as Compared to Neighbors of        Ru2 Check
PLAT242_ALERT_2_C Low    'MainMol' Ueq as Compared to Neighbors of       C31A Check
PLAT242_ALERT_2_C Low    'MainMol' Ueq as Compared to Neighbors of       C36A Check
PLAT242_ALERT_2_C Low    'MainMol' Ueq as Compared to Neighbors of       C76A Check
PLAT242_ALERT_2_C Low    'MainMol' Ueq as Compared to Neighbors of       C78A Check
PLAT243_ALERT_4_C High   'Solvent' Ueq as Compared to Neighbors of        C93 Check
PLAT244_ALERT_4_C Low    'Solvent' Ueq as Compared to Neighbors of        C92 Check
```

And 3 other PLAT244 Alerts

```
PLAT244_ALERT_4_C Low    'Solvent' Ueq as Compared to Neighbors of        C94 Check
PLAT244_ALERT_4_C Low    'Solvent' Ueq as Compared to Neighbors of        C95 Check
PLAT244_ALERT_4_C Low    'Solvent' Ueq as Compared to Neighbors of        C96 Check
PLAT250_ALERT_2_C Large U3/U1 Ratio for Average U(i,j) Tensor ....        2.1 Note
PLAT260_ALERT_2_C Large Average Ueq of Residue Including        P1      0.170 Check
```

Author Response: Solvent chloroform is slightly disordered. Chemical evidence shows that the structure is correct.

```
PLAT260_ALERT_2_C Large Average Ueq of Residue Including        P2      0.169 Check
```

Author Response: Solvent chloroform is slightly disordered. Chemical evidence shows that the structure is correct.

```
PLAT260_ALERT_2_C Large Average Ueq of Residue Including      Cl21      0.166 Check
```

Author Response: Solvent chloroform is slightly disordered. Chemical evidence shows that the structure is correct.

```
PLAT260_ALERT_2_C Large Average Ueq of Residue Including      Cl31      0.220 Check
```

Author Response: Solvent chloroform is slightly disordered. Chemical evidence shows that the structure is correct.

```
PLAT260_ALERT_2_C Large Average Ueq of Residue Including      Cl41      0.209 Check
```

Author Response: Solvent chloroform is slightly disordered. Chemical evidence shows that the structure is correct.

```
PLAT260_ALERT_2_C Large Average Ueq of Residue Including      Cl51      0.152 Check
```

Author Response: Solvent chloroform is slightly disordered. Chemical evidence shows that the structure is correct.

```
PLAT332_ALERT_2_C Large Phenyl C-C Range     C31A      -C36A     .       0.17 Ang.
PLAT336_ALERT_2_C Long Bond Distance for ..... C94       -Cl41          1.910 Ang.
PLAT360_ALERT_2_C Short  C(sp3)-C(sp3) Bond  C78A     - C79A     .       1.35 Ang.
PLAT368_ALERT_2_C Short  C(sp2)-C(sp2) Bond  C31      - C32      .       1.23 Ang.
PLAT368_ALERT_2_C Short  C(sp2)-C(sp2) Bond  C34      - C35      .       1.14 Ang.
PLAT480_ALERT_4_C Long H...A H-Bond Reported H1A      ..F15      .       2.61 Ang.
```

And 5 other PLAT480 Alerts

```
PLAT480_ALERT_4_C Long H...A H-Bond Reported H3       ..F21      .       2.58 Ang.
PLAT480_ALERT_4_C Long H...A H-Bond Reported H5       ..F21      .       2.63 Ang.
PLAT480_ALERT_4_C Long H...A H-Bond Reported H5       ..F26      .       2.64 Ang.
PLAT480_ALERT_4_C Long H...A H-Bond Reported H5A      ..F14      .       2.60 Ang.
PLAT480_ALERT_4_C Long H...A H-Bond Reported H5A'     ..CL63     .       3.01 Ang.
```

Alert level G

```
PLAT002_ALERT_2_G Number of Distance or Angle Restraints on AtSite          8 Note
PLAT083_ALERT_2_G SHELXL Second Parameter in WGHT  Unusually Large      11.65 Why ?
PLAT171_ALERT_4_G The CIF-Embedded .res File Contains EADP Records          1 Report
PLAT172_ALERT_4_G The CIF-Embedded .res File Contains DFIX Records          1 Report
PLAT175_ALERT_4_G The CIF-Embedded .res File Contains SAME Records          1 Report
PLAT232_ALERT_2_G Hirshfeld Test Diff (M-X)  Ru2      --C71A     .        6.0 s.u.
PLAT244_ALERT_4_G Low    'Solvent' Ueq as Compared to Neighbors of         P1 Check
PLAT244_ALERT_4_G Low    'Solvent' Ueq as Compared to Neighbors of         P2 Check
PLAT343_ALERT_2_G Unusual       sp3Angle Range in Main Residue for       C78A Check
PLAT395_ALERT_2_G Deviating  X-O-Y    Angle From 120 for O1"            104.8 Degree
PLAT395_ALERT_2_G Deviating  X-O-Y    Angle From 120 for O1A"           106.2 Degree
PLAT432_ALERT_2_G Short Inter X...Y Contact  F16      ..C6'              2.96 Ang.
                                          x,y,1+z  =       1_556 Check
PLAT720_ALERT_4_G Number of Unusual/Non-Standard Labels .........          18 Note
PLAT722_ALERT_1_G Angle   Calc      119.00, Rep       120.10 Dev...      1.10 Degree
              C32A    -C33A    -H33A       1_555  1_555  1_555     #  162 Check
PLAT779_ALERT_4_G Suspect or Irrelevant (Bond) Angle(s) in CIF ...      40.60 Deg.
              C96  -CL62 -CL63     1_555   1_555   1_555 ......     #  489 Check
PLAT779_ALERT_4_G Suspect or Irrelevant (Bond) Angle(s) in CIF ...      41.10 Deg.
              C96  -CL63 -CL62     1_555   1_555   1_555 ......     #  490 Check
PLAT791_ALERT_4_G Model has Chirality at C1         (Sohnke SpGr)          S Verify
```

And 9 other PLAT791 Alerts

```
PLAT791_ALERT_4_G Model has Chirality at C1A        (Sohnke SpGr)          S Verify
PLAT791_ALERT_4_G Model has Chirality at C2         (Sohnke SpGr)          S Verify
PLAT791_ALERT_4_G Model has Chirality at C2A        (Sohnke SpGr)          S Verify
PLAT791_ALERT_4_G Model has Chirality at C3         (Sohnke SpGr)          S Verify
PLAT791_ALERT_4_G Model has Chirality at C3A        (Sohnke SpGr)          R Verify
PLAT791_ALERT_4_G Model has Chirality at C4         (Sohnke SpGr)          R Verify
PLAT791_ALERT_4_G Model has Chirality at C4A        (Sohnke SpGr)          R Verify
PLAT791_ALERT_4_G Model has Chirality at C5         (Sohnke SpGr)          R Verify
PLAT791_ALERT_4_G Model has Chirality at C5A        (Sohnke SpGr)          R Verify
PLAT860_ALERT_3_G Number of Least-Squares Restraints ............          10 Note
PLAT933_ALERT_2_G Number of OMIT Records in Embedded .res File ...         25 Note
```

```
  3 ALERT level A = Most likely a serious problem - resolve or explain
 17 ALERT level B = A potentially serious problem, consider carefully
 65 ALERT level C = Check. Ensure it is not caused by an omission or oversight
 28 ALERT level G = General information/check it is not something unexpected

  1 ALERT type 1 CIF construction/syntax error, inconsistent or missing data
 63 ALERT type 2 Indicator that the structure model may be wrong or deficient
  3 ALERT type 3 Indicator that the structure quality may be low
 46 ALERT type 4 Improvement, methodology, query or suggestion
  0 ALERT type 5 Informative message, check
```

checkCIF publication errors

Alert level A

```
PUBL004_ALERT_1_A  The contact author's name and address are missing,
             _publ_contact_author_name and _publ_contact_author_address.
PUBL005_ALERT_1_A  _publ_contact_author_email, _publ_contact_author_fax and
             _publ_contact_author_phone are all missing.
            At least one of these should be present.
```

```
  2 ALERT level A = Data missing that is essential or data in wrong format
  0 ALERT level G = General alerts. Data that may be required is missing
```

Publication of your CIF

You should attempt to resolve as many as possible of the alerts in all categories. Often the minor alerts point to easily fixed oversights, errors and omissions in your CIF or refinement strategy, so attention to these fine details can be worthwhile. In order to resolve some of the more serious problems it may be necessary to carry out additional measurements or structure refinements. However, the nature of your study may justify the reported deviations from journal submission requirements and the more serious of these should be commented upon in the discussion or experimental section of a paper or in the "special_details" fields of the CIF. *checkCIF* was carefully designed to identify outliers and unusual parameters, but every test has its limitations and alerts that are not important in a particular case may appear. Conversely, the absence of alerts does not guarantee there are no aspects of the results

needing attention. It is up to the individual to critically assess their own results and, if necessary, seek expert advice.
If level A alerts remain, which you believe to be justified deviations, and you intend to submit this CIF for publication in a journal, you should additionally insert an explanation in your CIF using the Validation Reply Form (VRF) below. This will allow your explanation to be considered as part of the review process.

Validation response form

Please find below a validation response form (VRF) that can be filled in and pasted into your CIF.

```
# start Validation Reply Form
_vrf_PUBL004_GLOBAL
;
PROBLEM: The contact author's name and address are missing,
RESPONSE: ...
;
_vrf_PUBL005_GLOBAL
;
PROBLEM: _publ_contact_author_email, _publ_contact_author_fax and
RESPONSE: ...
;
# end Validation Reply Form
```

If you wish to submit your CIF for publication in Acta Crystallographica Section C or E, you should upload your CIF via the web. If you wish to submit your CIF for publication in IUCrData you should upload your CIF via the web. If your CIF is to form part of a submission to another IUCr journal, you will be asked, either during electronic submission or by the Co-editor handling your paper, to upload your CIF via our web site.

PLATON version of 13/07/2021; check.def file version of 13/07/2021

Datablock Ir_2 - ellipsoid plot

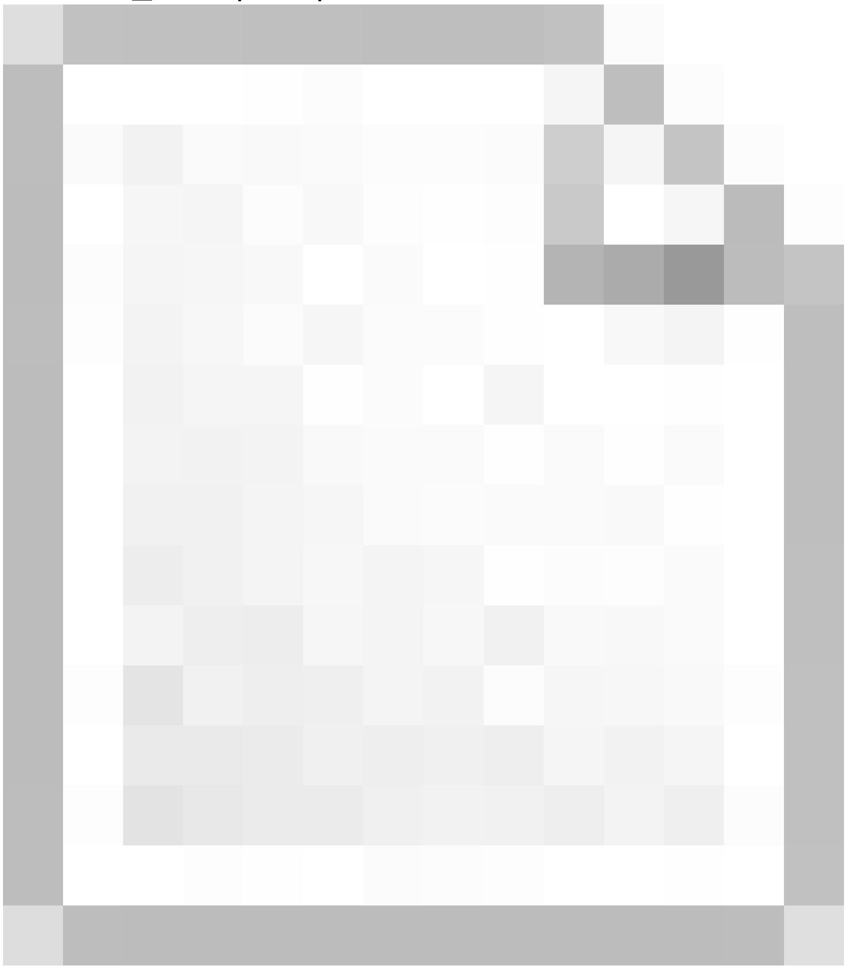

Datablock Ru_1 - ellipsoid plot

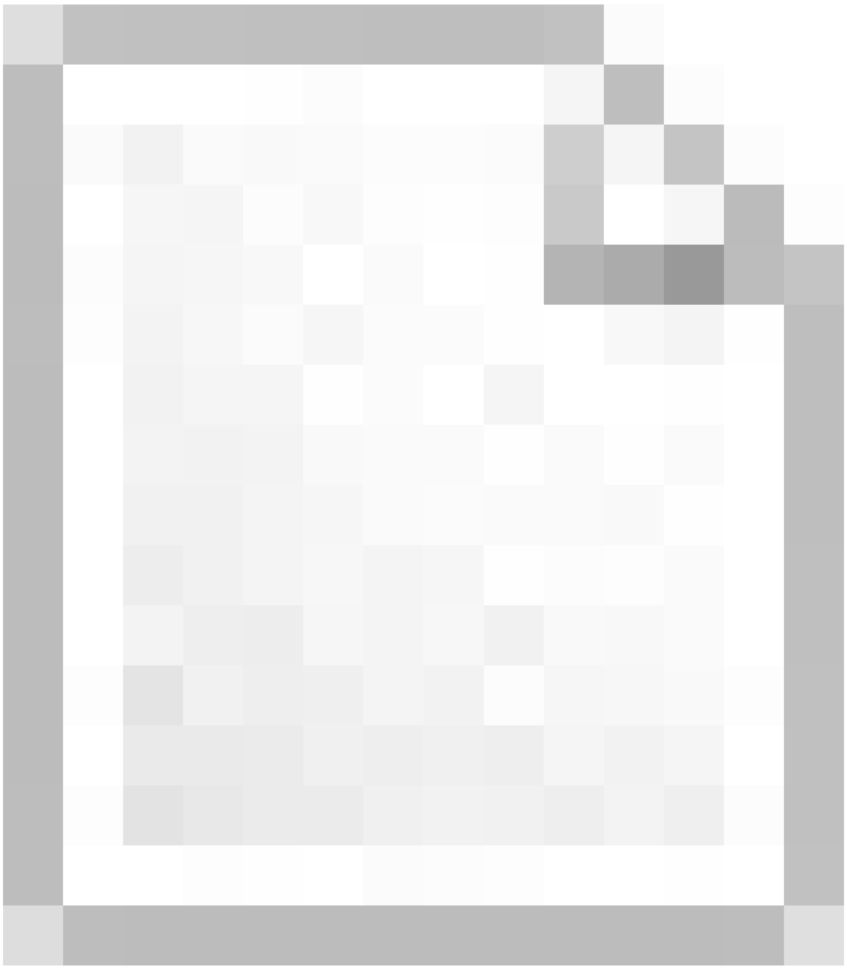

Download CIF editor (publCIF) from the IUCr
Download CIF editor (enCIFer) from the CCDC
Test a new CIF entry
