# Supplementary material for: Reactive Oxygen Species Production Is Responsible for Antineoplastic Activity of Osmium, Ruthenium, Iridium and Rhodium Half-Sandwich Type Complexes with Bidentate Glycosyl Heterocyclic Ligands in Various Cancer Cell Models
Source: Int J Mol Sci. 2022 Jan 12;23(2):813. doi: 10.3390/ijms23020813 (PMC8776094; doi:10.3390/ijms23020813)
Supplement: Supplementary file 1 [file ijms-23-00813-s001.zip › KI-cikk-2-suppinfo-final.pdf]

SUPPORTING INFORMATION

Reactive Oxygen Species Production Is Responsible for Antineoplastic Activity of Osmium, Ruthenium, Iridium and Rhodium Half-Sandwich Type Complexes with Bidentate Glycosyl Heterocyclic Ligands in Various Cancer Cell Models

István Kacsir [#1,2], Adrienn Sipos [#3], Attila Bényei [4], Eszter Janka [5], Péter Buglyó [6], László Somsák [1], Péter Bai [*3,7,8], Éva Bokor [*1]

1 Department of Organic Chemistry, University of Debrecen, PO Box 400, H-4002 Debrecen, Hungary; kacsir.istvan@science.unideb.hu (IK), somsak.laszlo@science.unideb.hu (LS), bokor.eva@science.unideb.hu (EB)
2 Doctoral School of Chemistry, University of Debrecen, PO Box 400, H-4002 Debrecen, Hungary;
3 Department of Medical Chemistry, Faculty of Medicine, University of Debrecen, H-4032 Debrecen, Hungary; siposadri@med.unideb.hu (AS), ujlaki.gyula@med.unideb.hu (GU), baip@med.unideb.hu (PB)
4 Department of Physical Chemistry, Faculty of Sciences and Technology, University of Debrecen, H-4032 Debrecen, Hungary; benyei.attila@science.unideb.hu (AB)
5 Department of Dermatology, Faculty of Medicine, University of Debrecen, Debrecen, Hungary; janka.eszter@med.unideb.hu (EJ)
6 Department of Inorganic & Analytical Chemistry, Faculty of Sciences and Technology, University of Debrecen, H-4032 Debrecen, Hungary; buglyo@science.unideb.hu (PB)
7 NKFIH-DE Lendület Laboratory of Cellular Metabolism, H-4032 Debrecen, Hungary;
8 Research Center for Molecular Medicine, Faculty of Medicine, University of Debrecen, H-4032 Debrecen, Hungary;
# Equal contribution.
* Corresponding authors.

Table of contents

1. **Copies of ^{1}H and ^{13}C NMR spectra** 2
2. **X-Ray diffraction study** 27
3. **Table S5.** Distribution coefficient of the new complexes (logD) 50
4. **References** 52

1. Copies of ^{1}H and ^{13}C NMR spectra

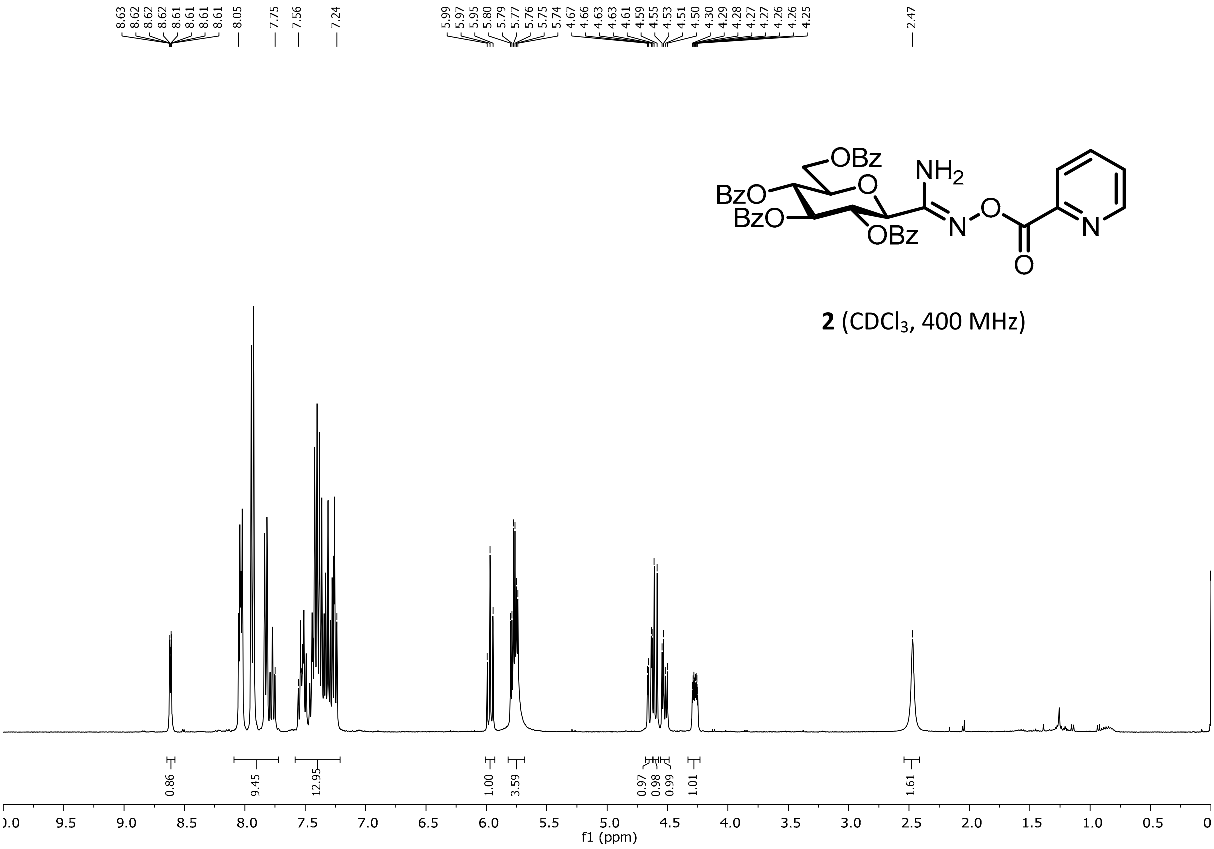

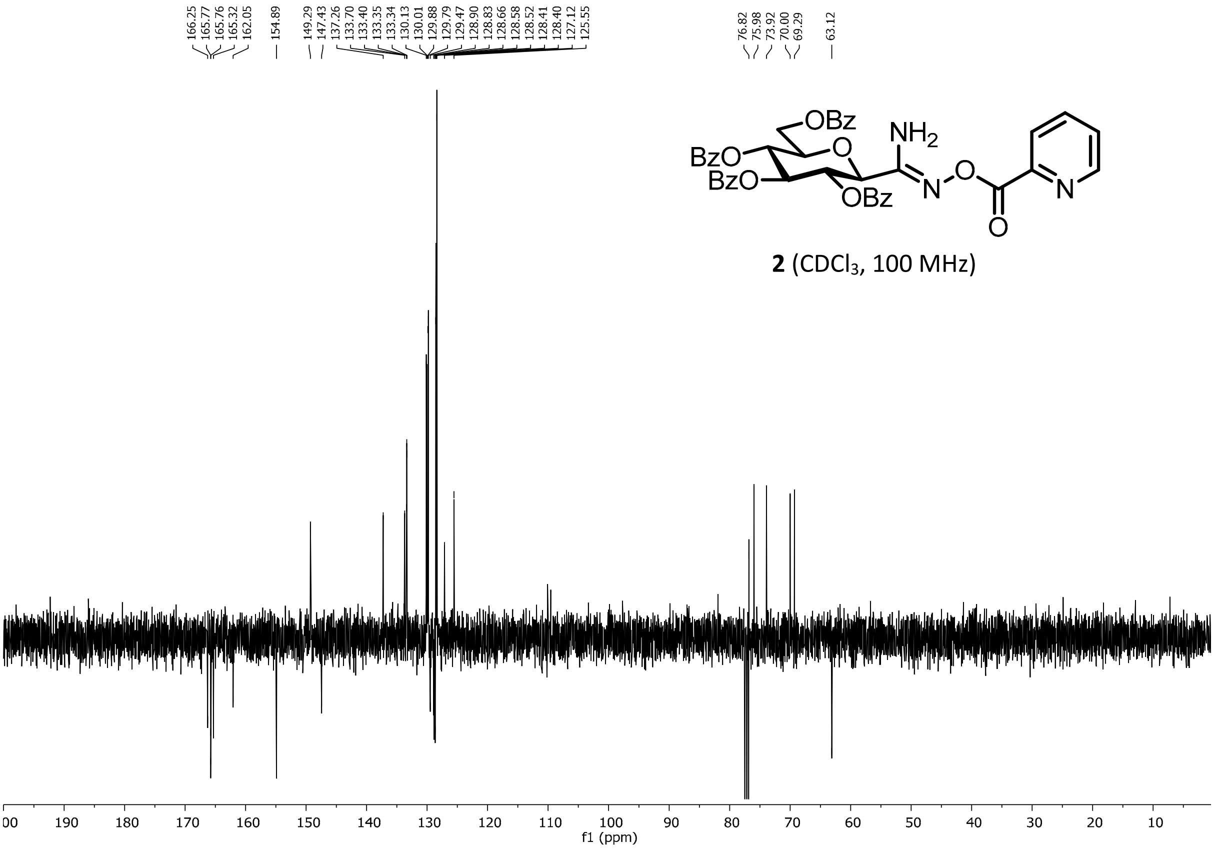

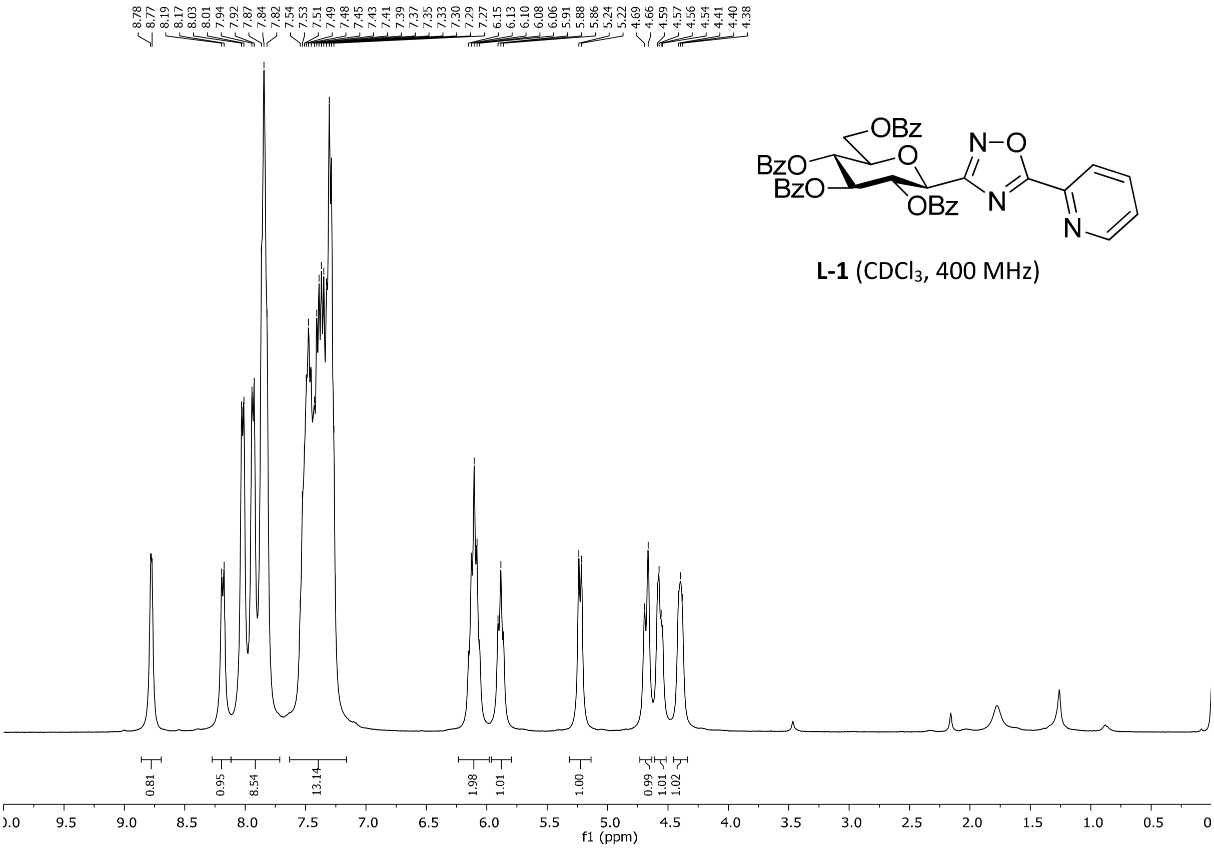
L-1 (CDCl3, 400 MHz)
f1 (ppm)

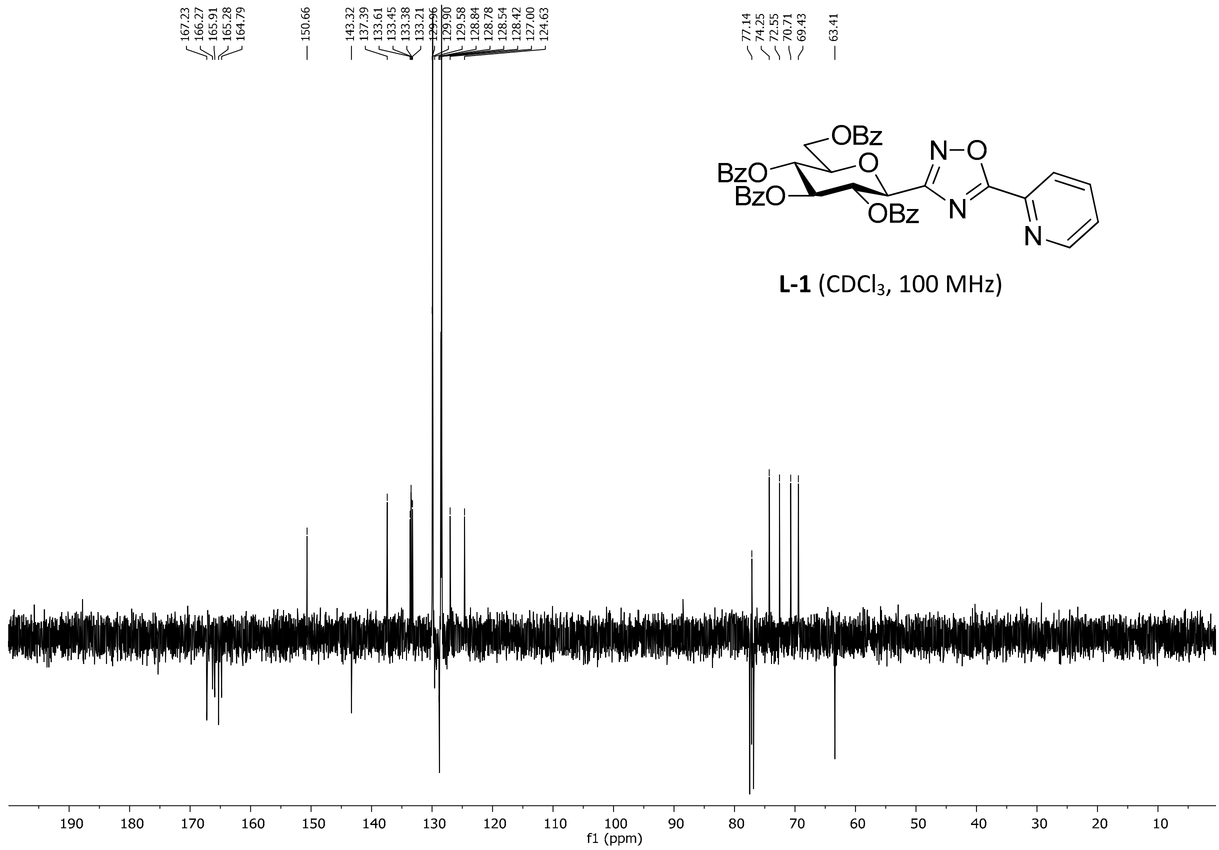
L-1 (CDCl3, 100 MHz)
f1 (ppm)

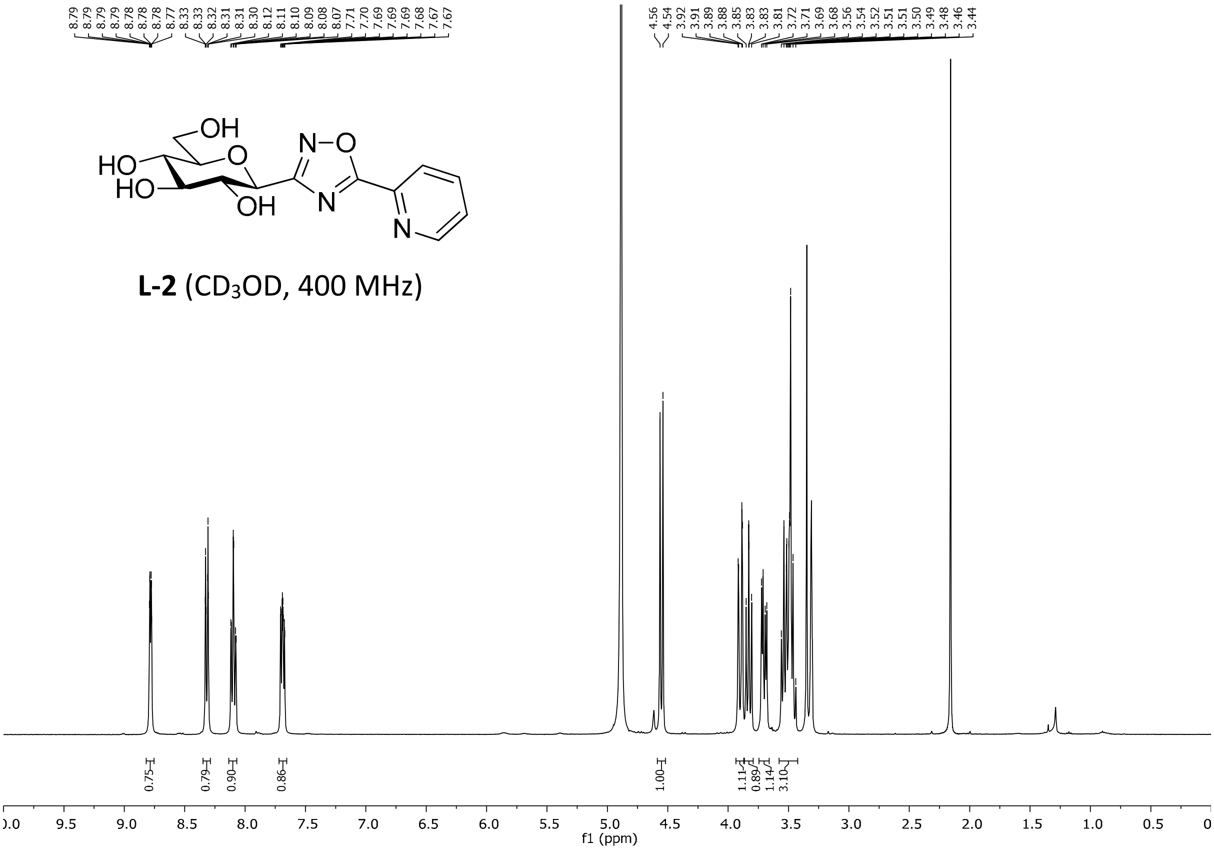

L-2 (CD_3OD, 400 MHz)

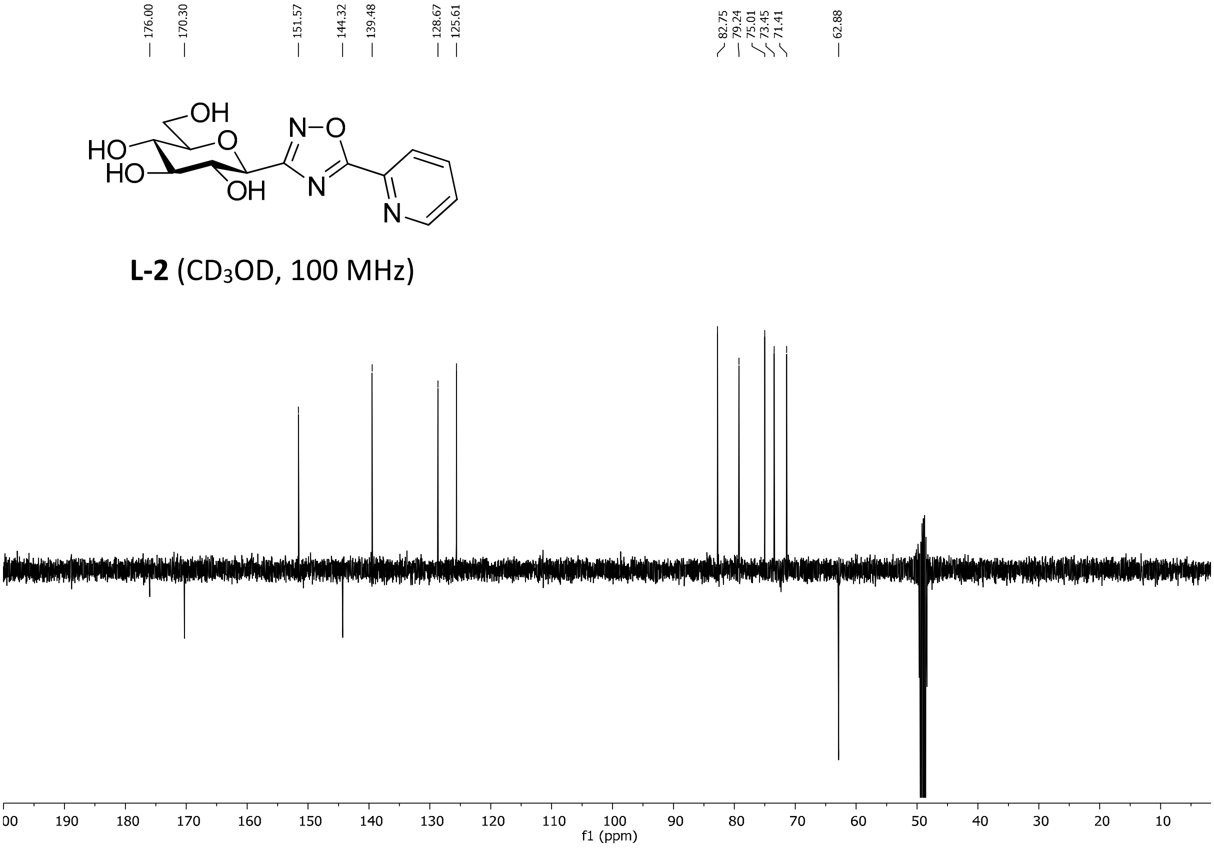

L-2 (CD_3OD, 100 MHz)

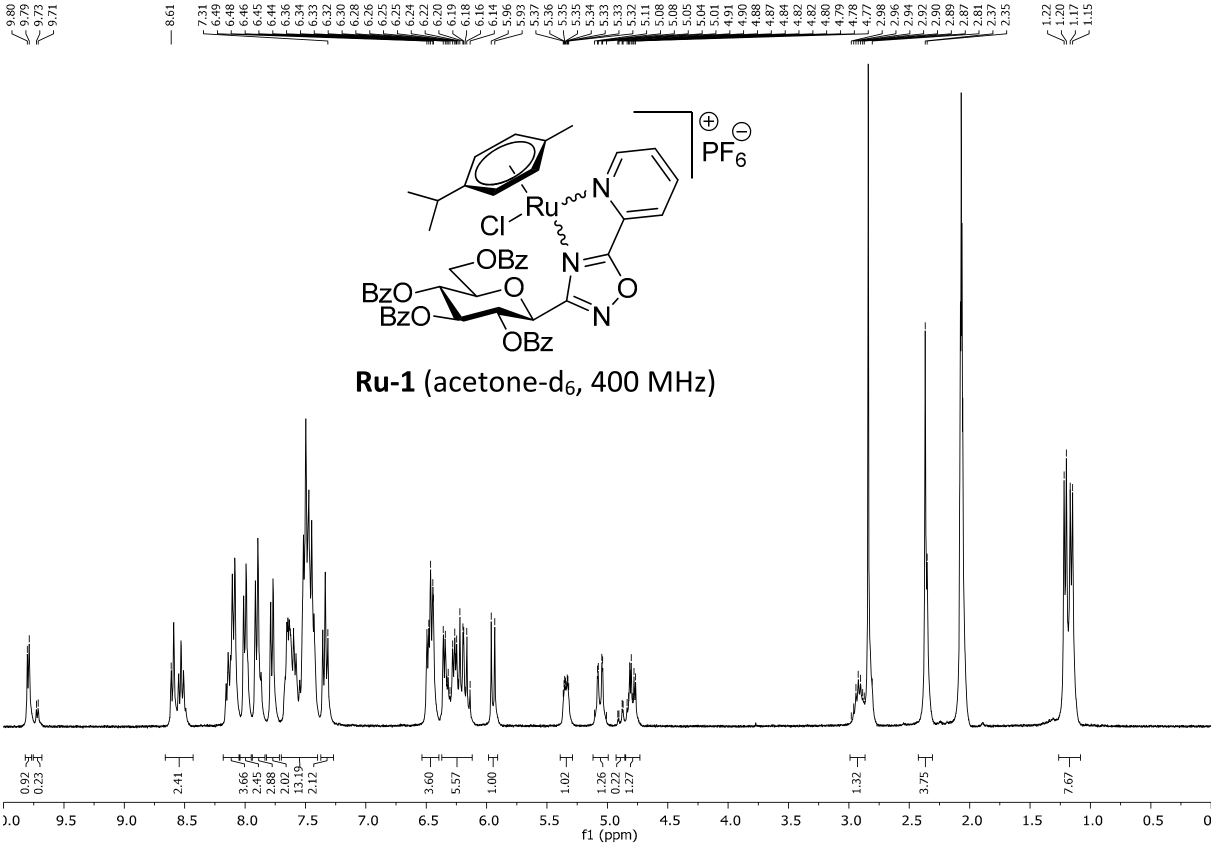
Ru-1 (acetone-d$_6$, 400 MHz)
f1 (ppm)

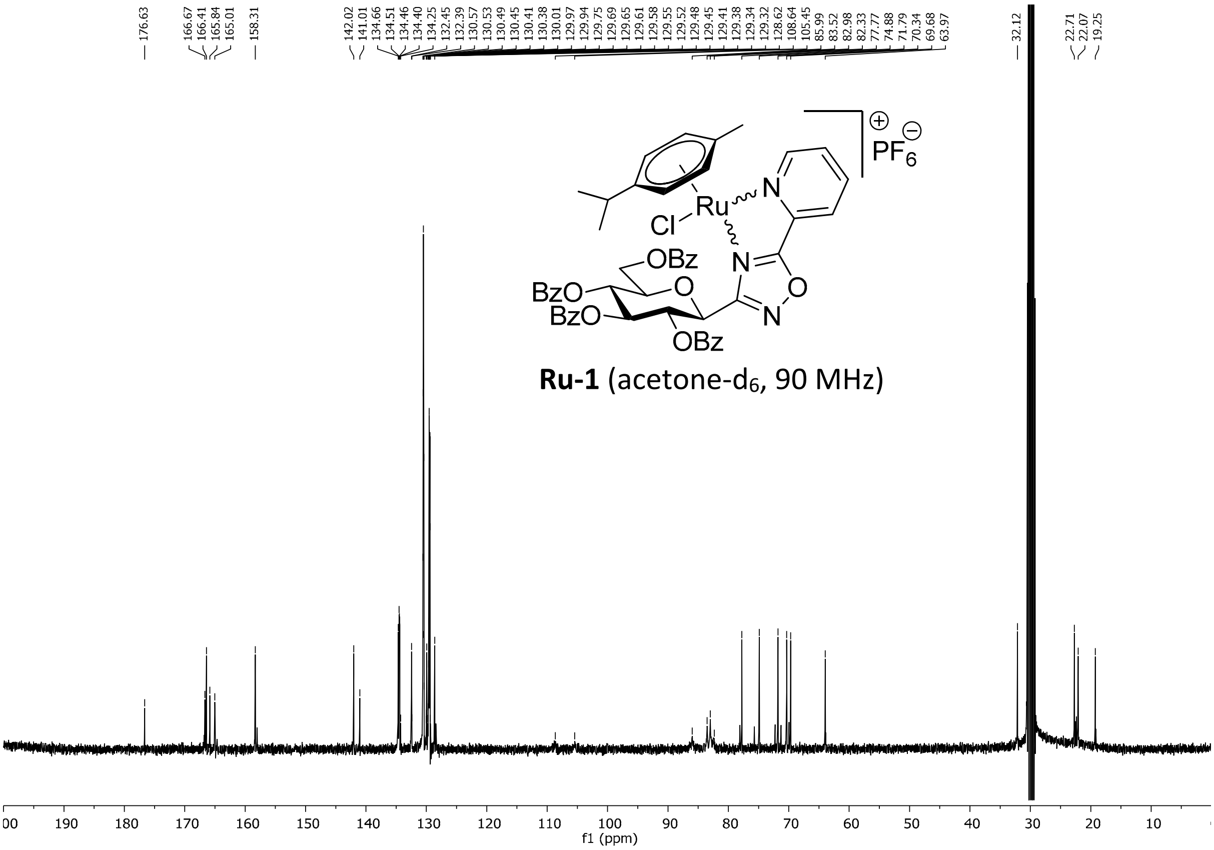
Ru-1 (acetone-d$_6$, 90 MHz)
f1 (ppm)

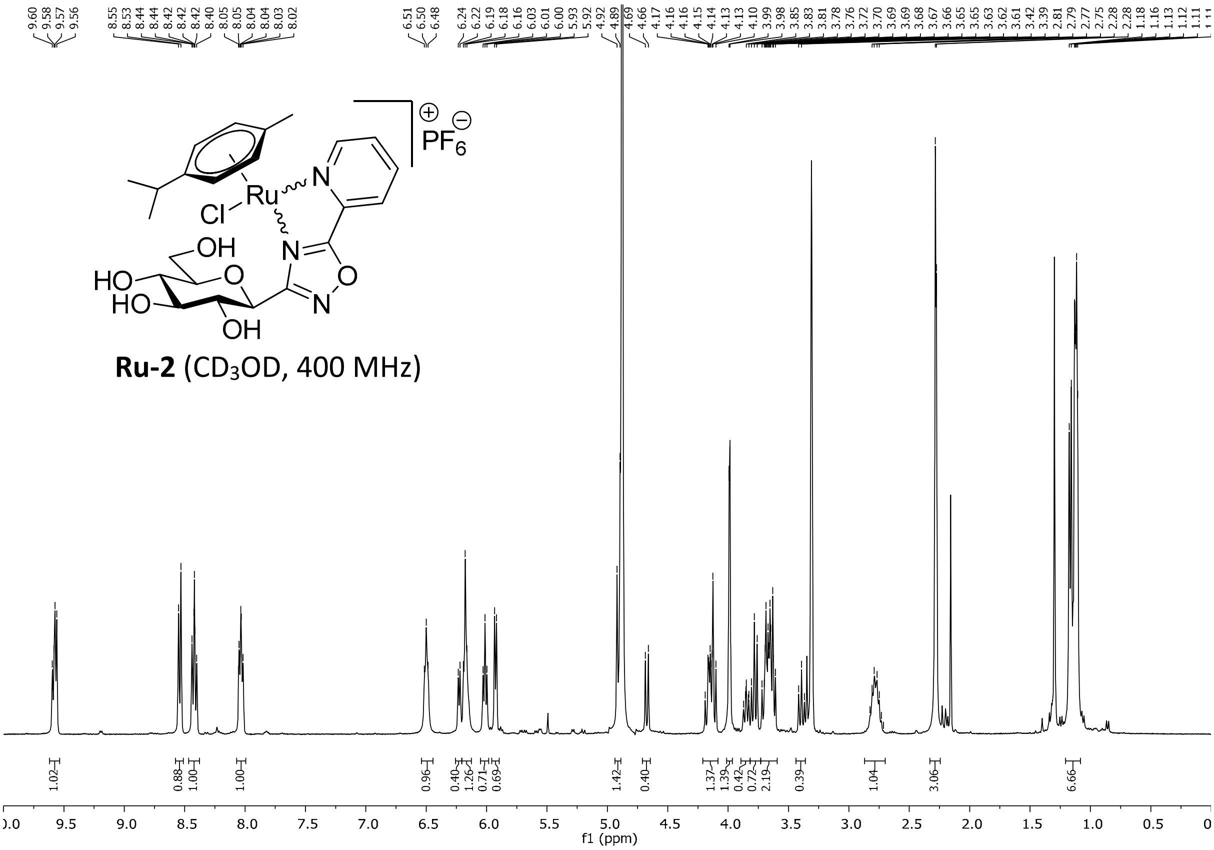
Ru-2 (CD3OD, 400 MHz)
f1 (ppm)

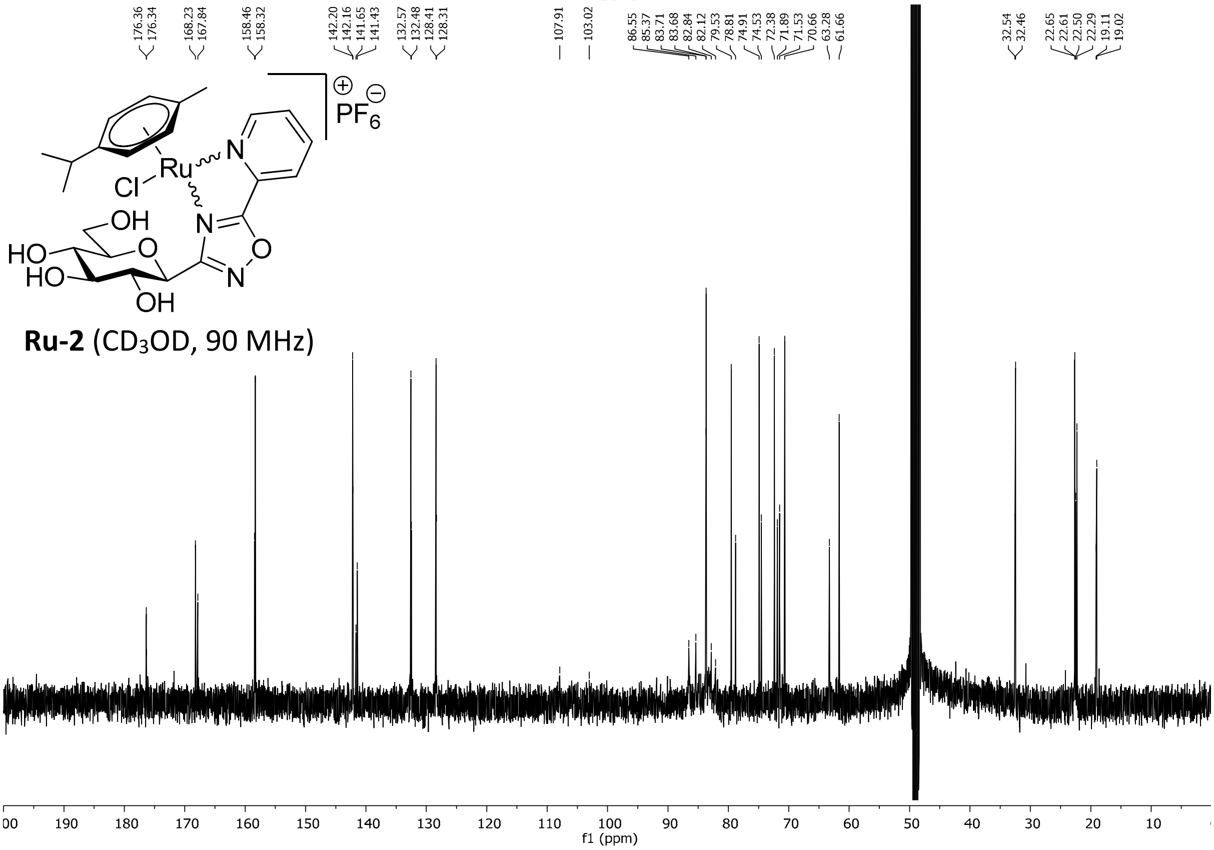
Ru-2 (CD3OD, 90 MHz)
f1 (ppm)

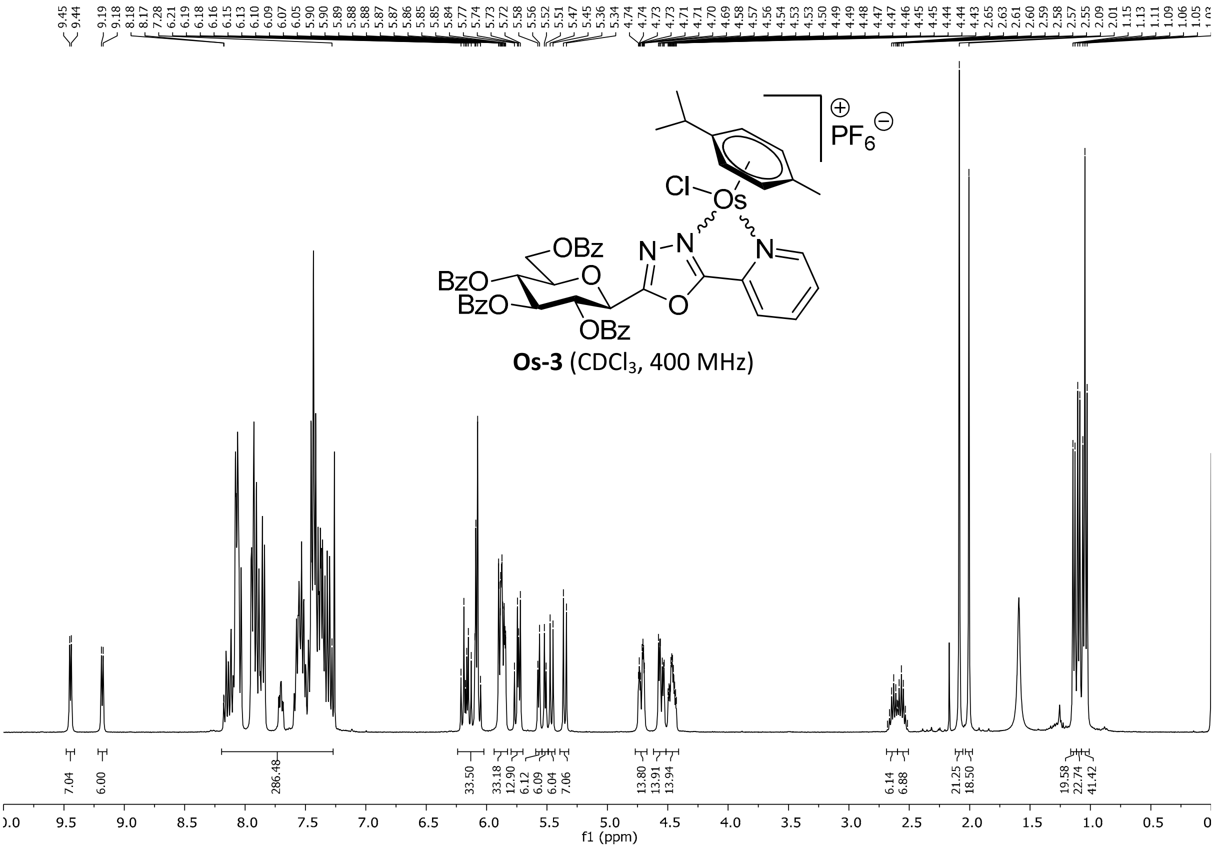
PF6
Cl
Os
OBz
BzO
BzO
OBz
N
N
N
O
O
Os-3 (CDCl3, 400 MHz)
f1 (ppm)

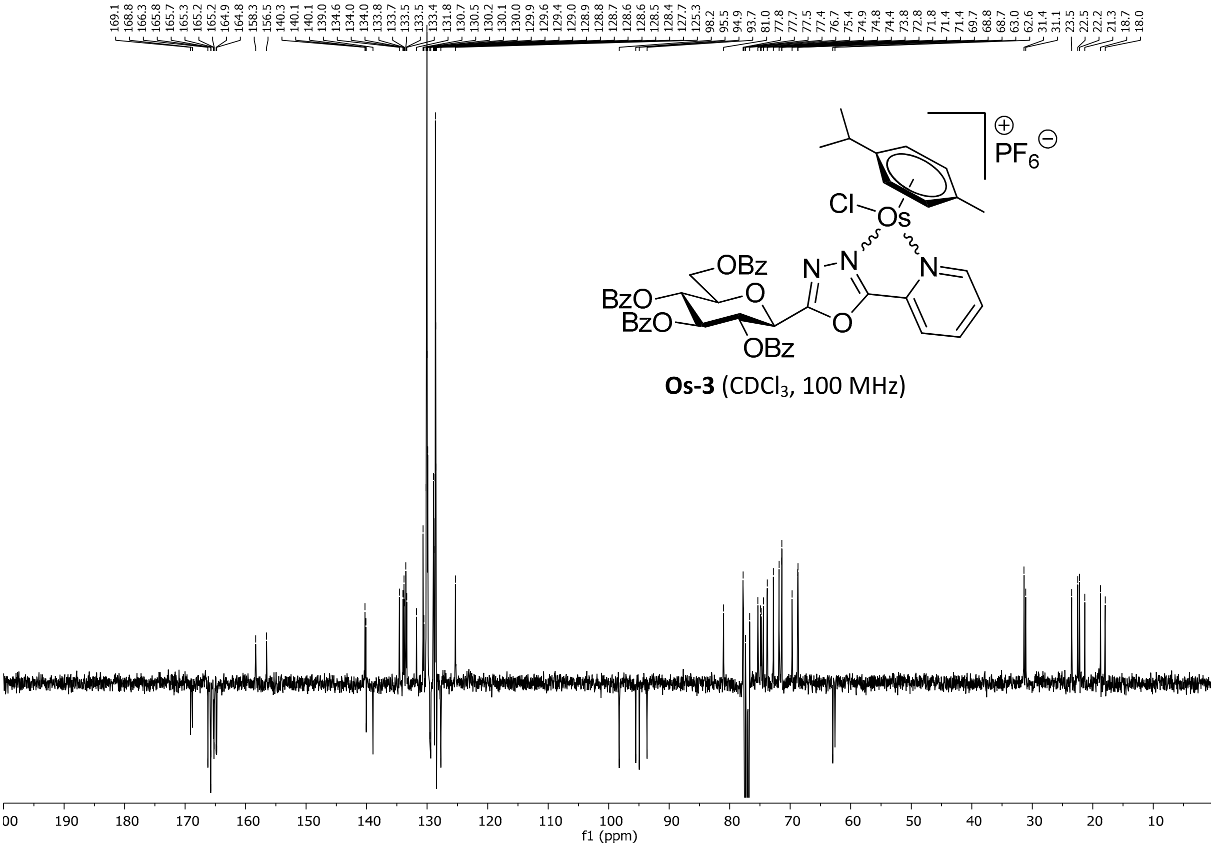
PF6
Cl
Os
OBz
BzO
BzO
OBz
N
N
N
O
O
Os-3 (CDCl3, 100 MHz)
f1 (ppm)

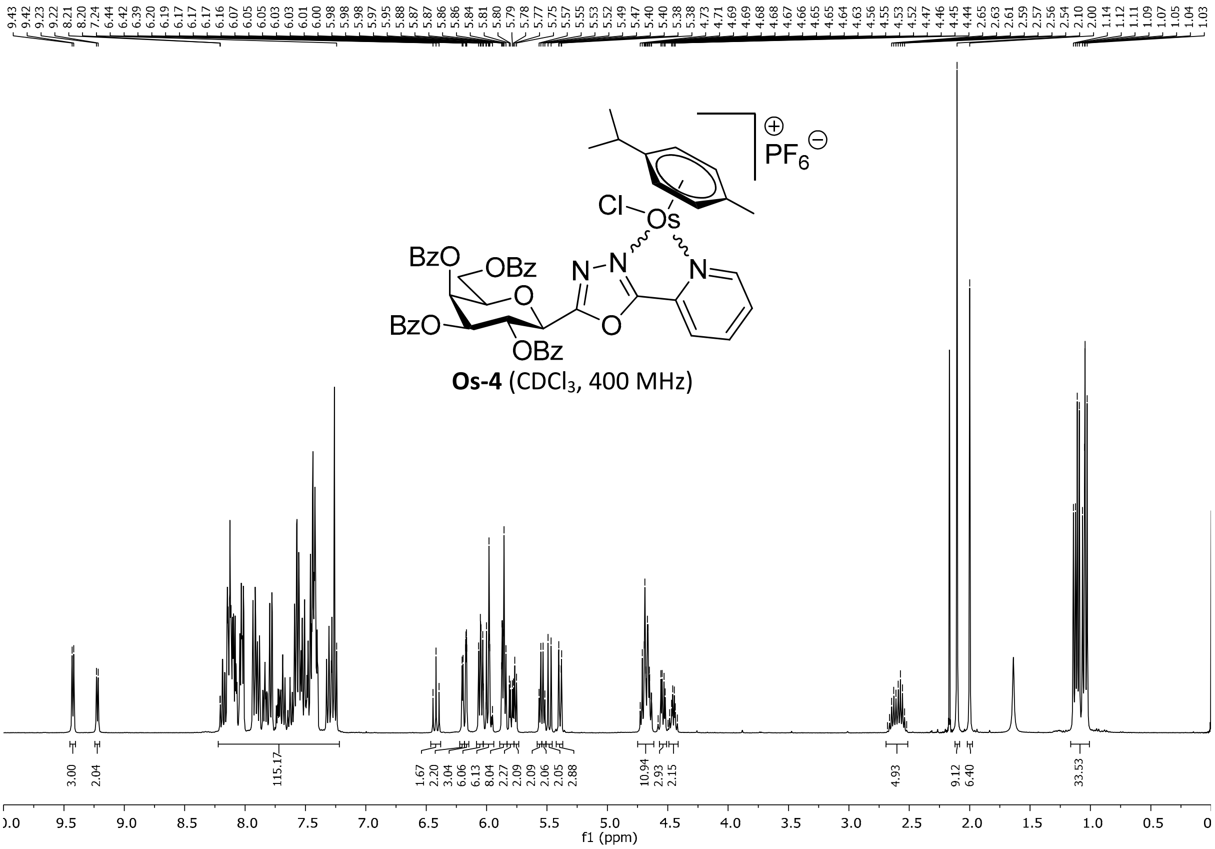
Cl
Os
PF6
BzO
OBz
N
N
N
O
BzO
O
OBz
Os-4 (CDCl3, 400 MHz)
f1 (ppm)

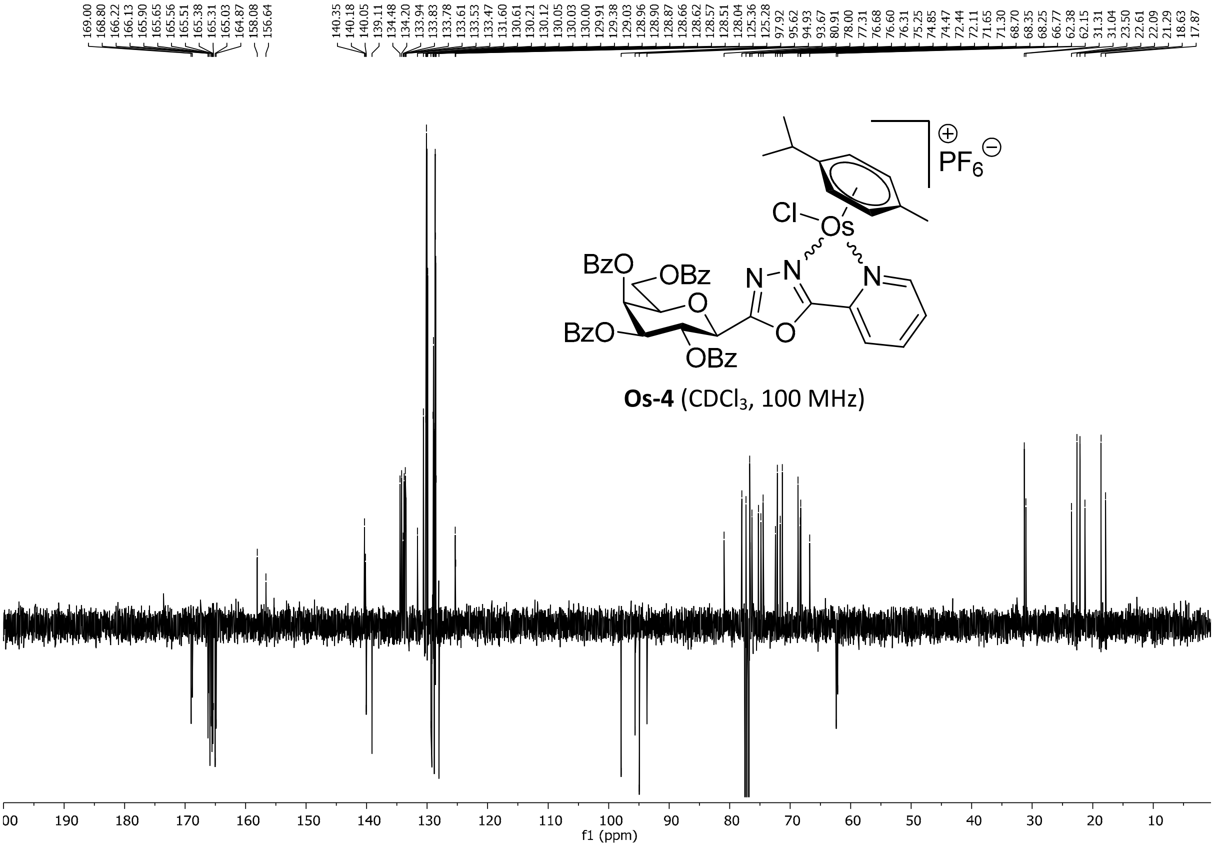
Cl
Os
PF6
BzO
OBz
N
N
N
O
BzO
O
OBz
Os-4 (CDCl3, 100 MHz)
f1 (ppm)

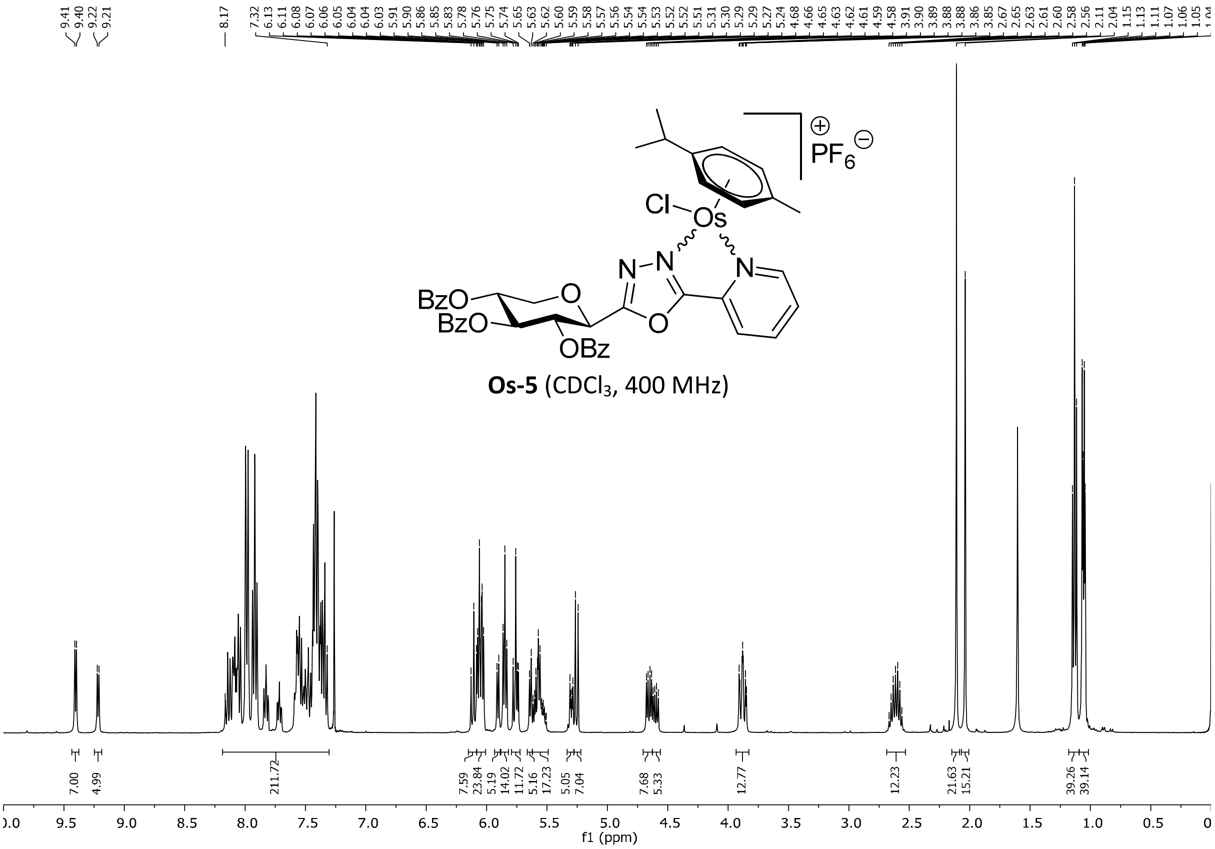
Os-5 (CDCl$_3$, 400 MHz)
f1 (ppm)

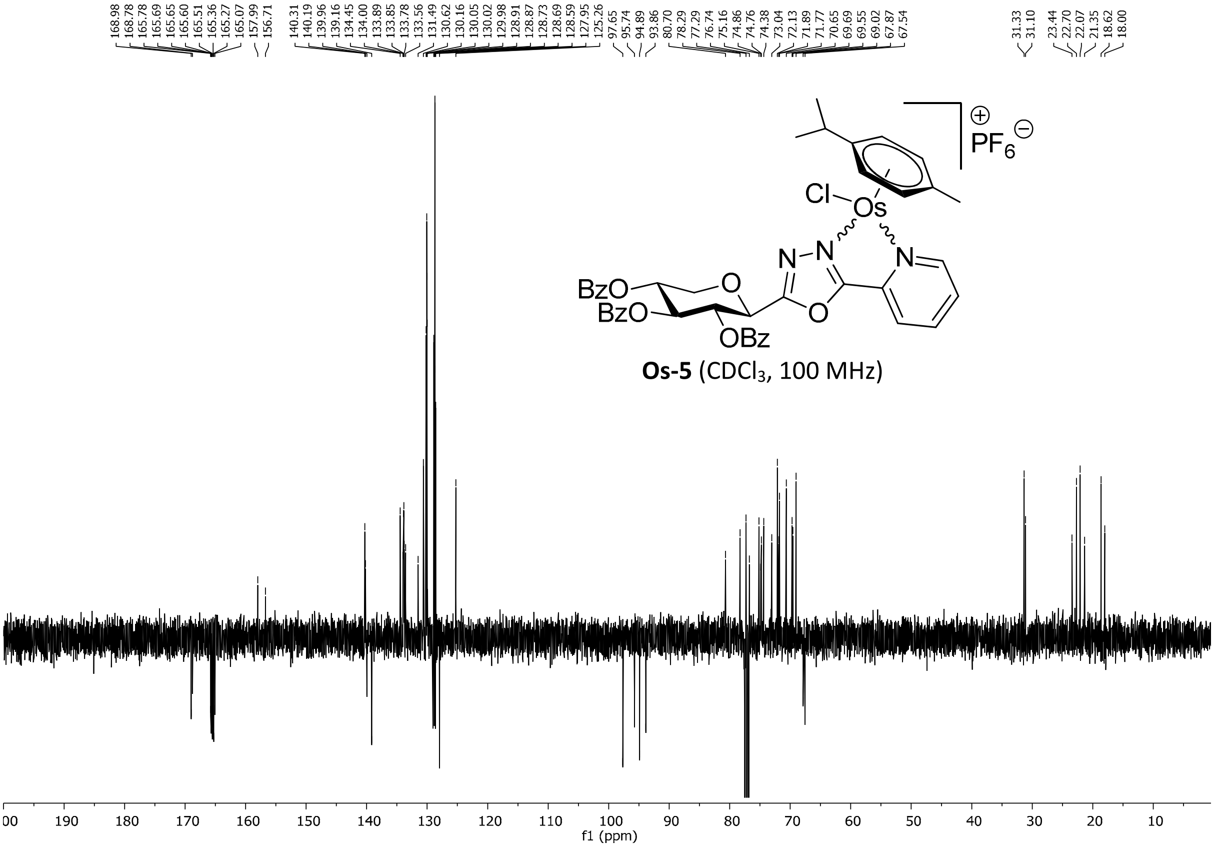
Os-5 (CDCl$_3$, 100 MHz)
f1 (ppm)

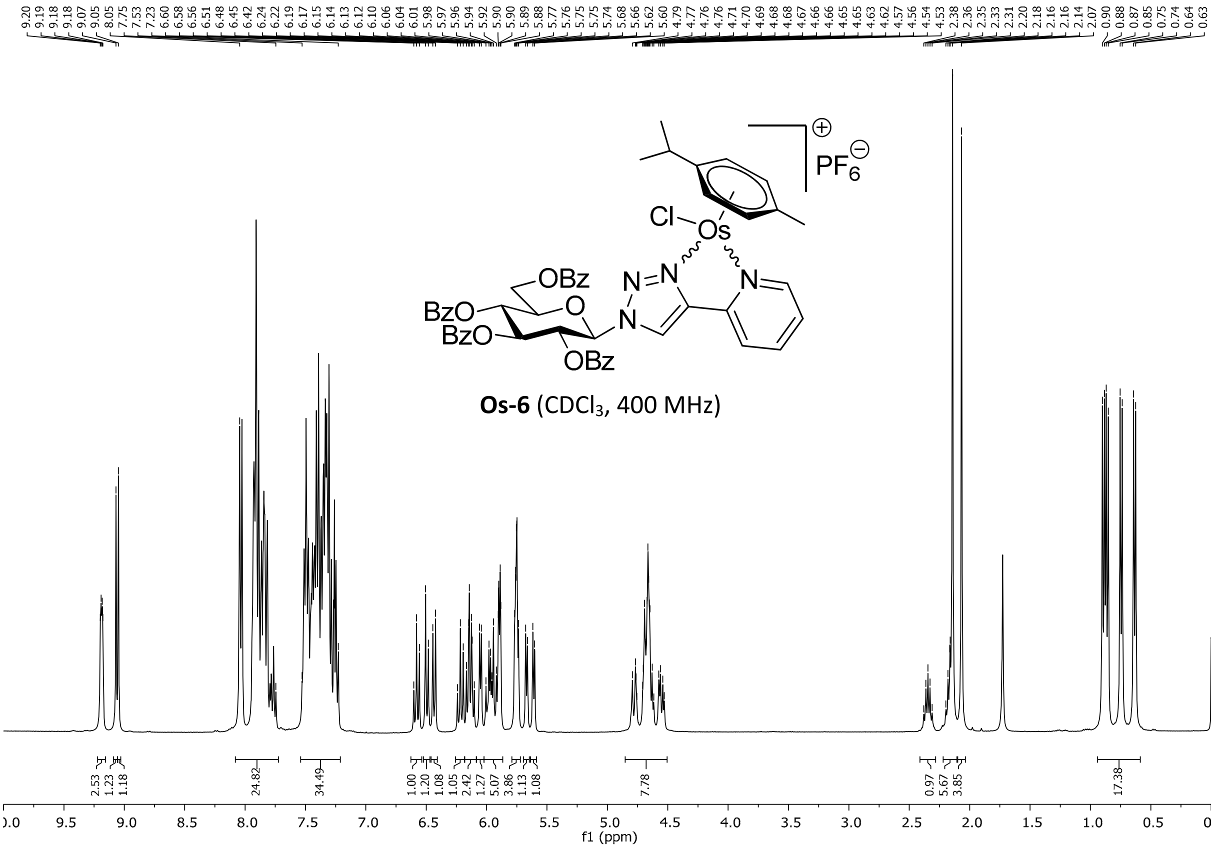
PF6
Cl
Os
N=N
N
N
OBz
BzO
BzO
O
OBz
Os-6 (CDCl3, 400 MHz)
f1 (ppm)

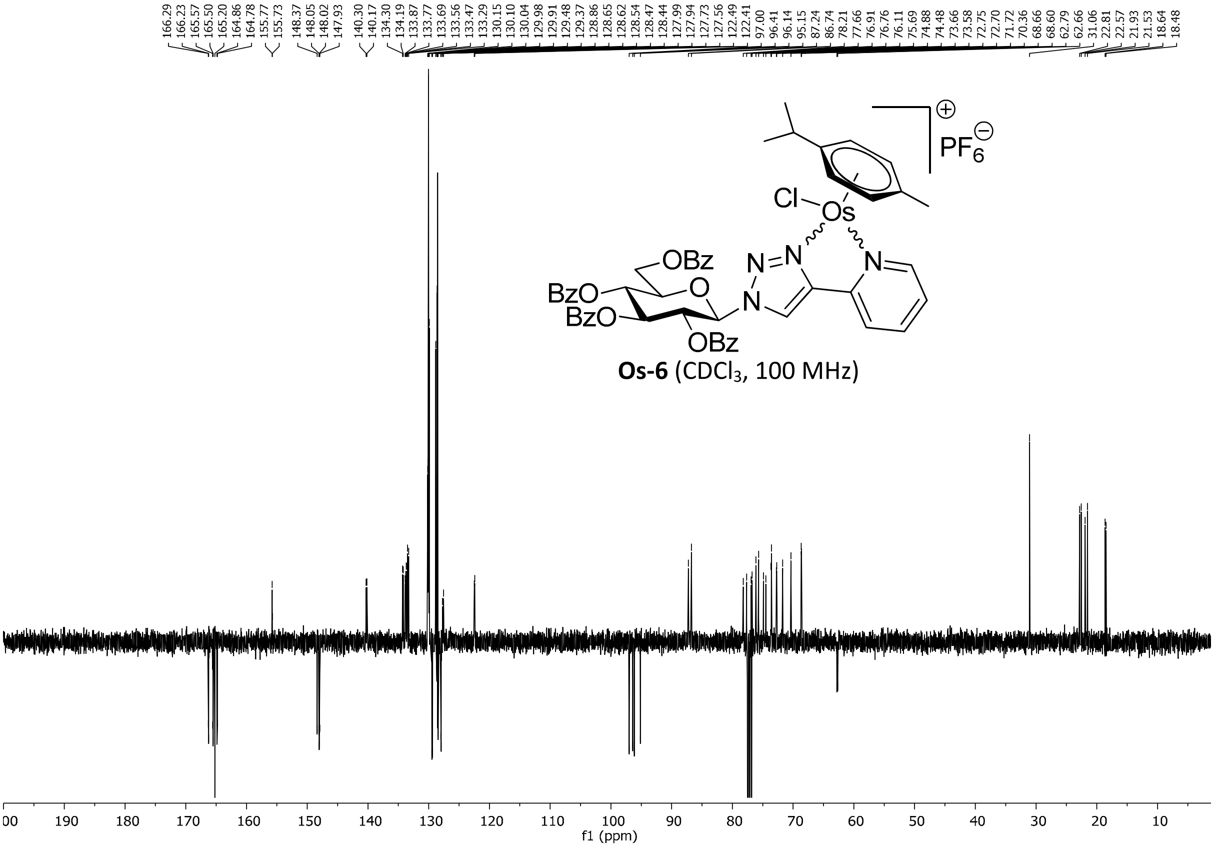
PF6
Cl
Os
N=N
N
N
OBz
BzO
BzO
O
OBz
Os-6 (CDCl3, 100 MHz)
f1 (ppm)

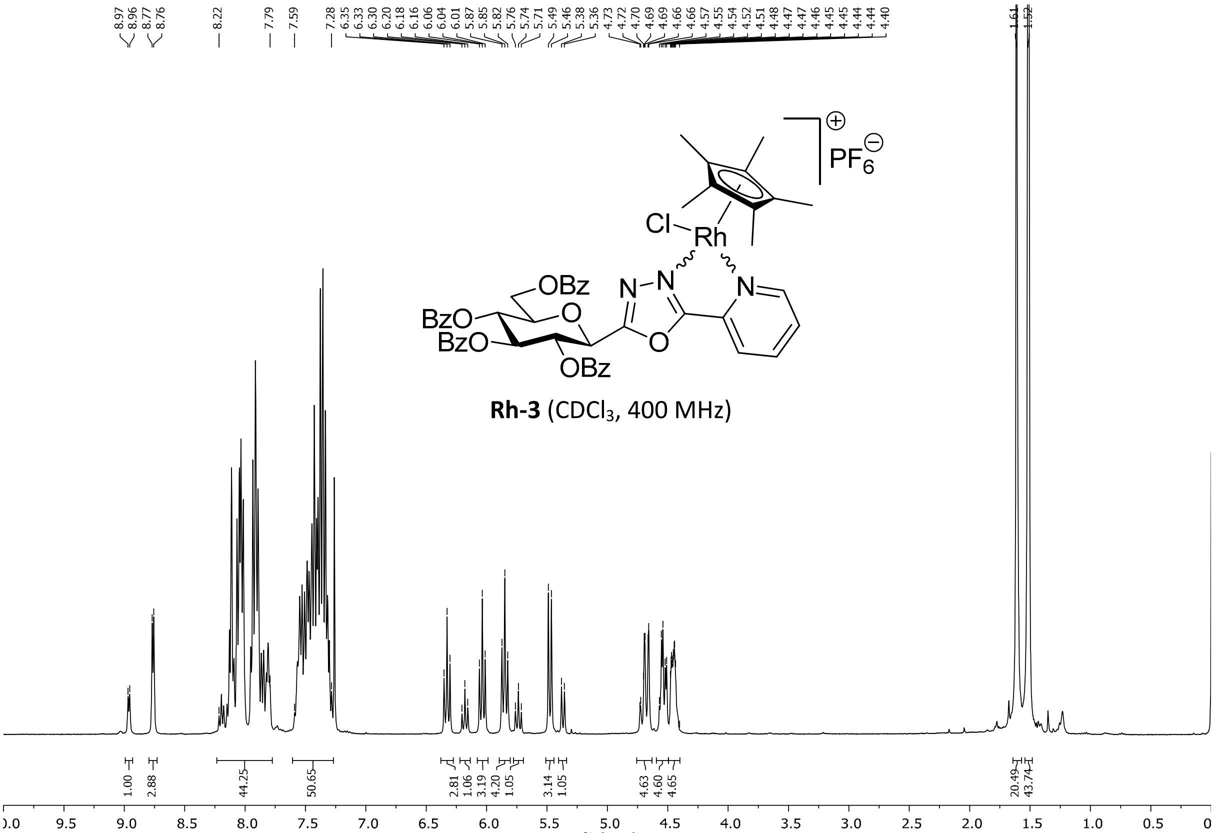
PF6
Cl
Rh
OBz
BzO
BzO
OBz
N
N
N
O
O
Rh-3 (CDCl3, 400 MHz)
f1 (ppm)

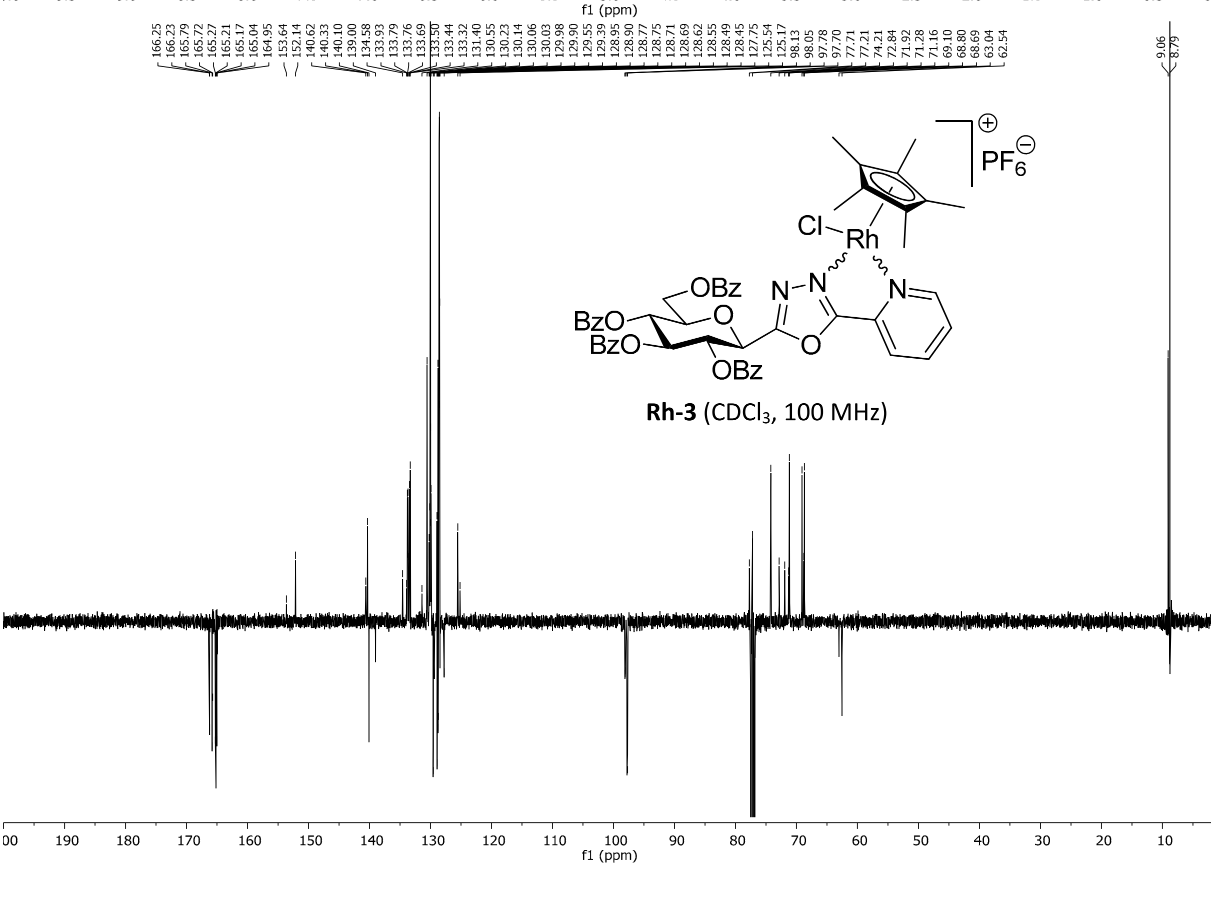
PF6
Cl
Rh
OBz
BzO
BzO
OBz
N
N
N
O
O
Rh-3 (CDCl3, 100 MHz)
f1 (ppm)

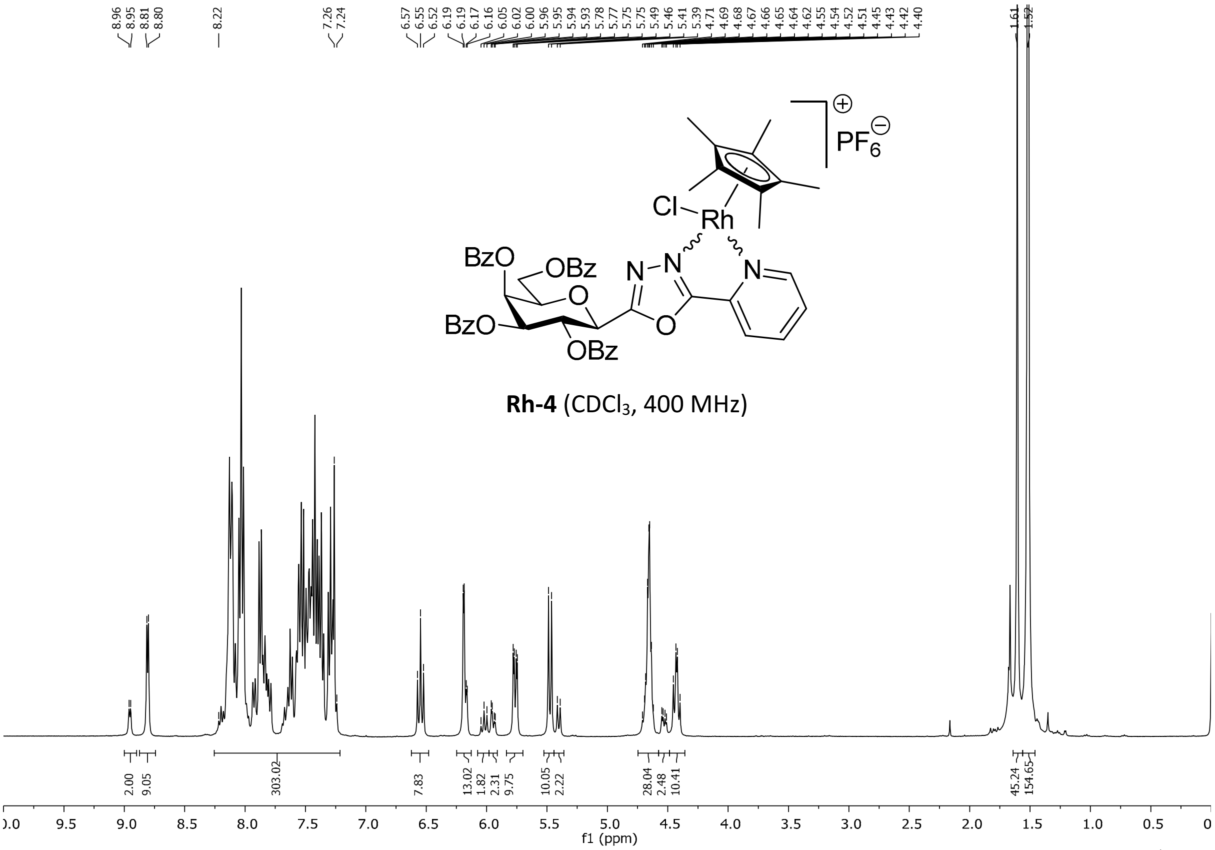

Rh-4 (CDCl$_3$, 400 MHz)

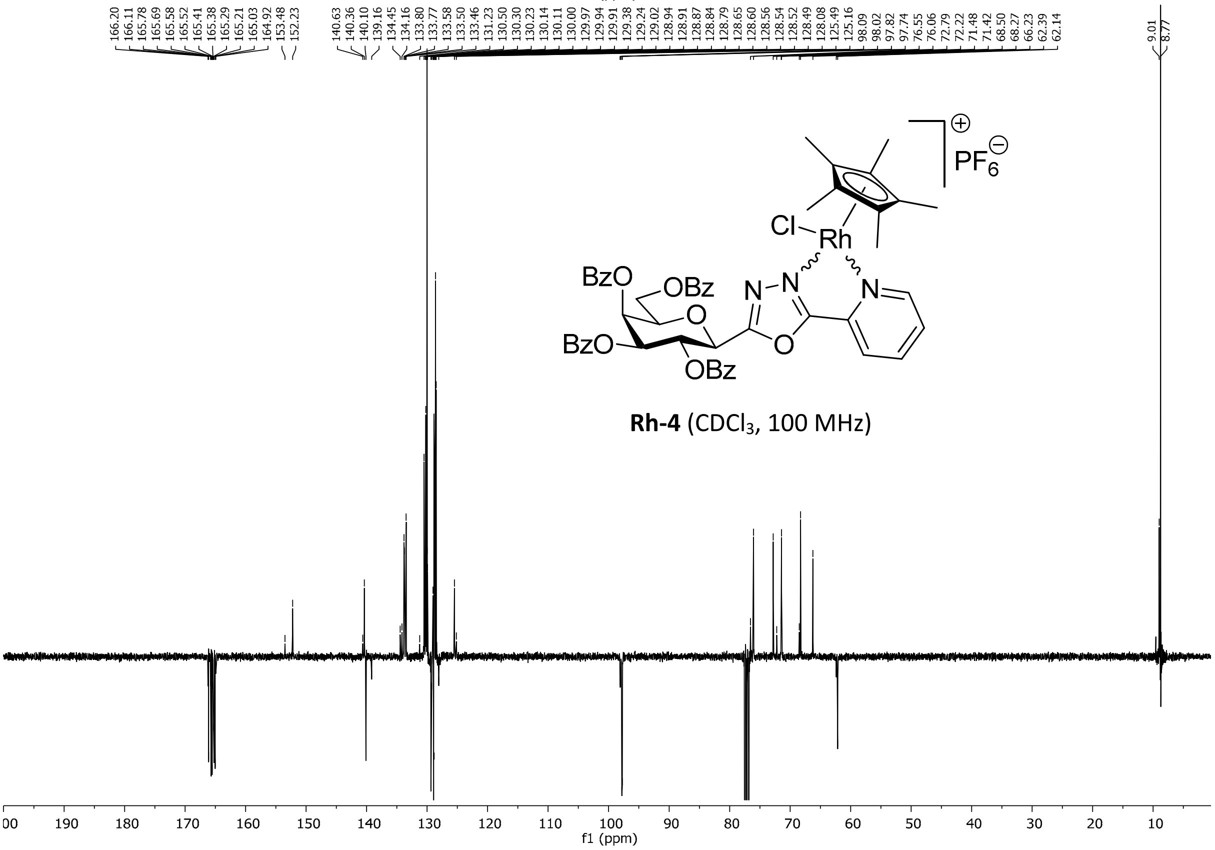

Rh-4 (CDCl$_3$, 100 MHz)

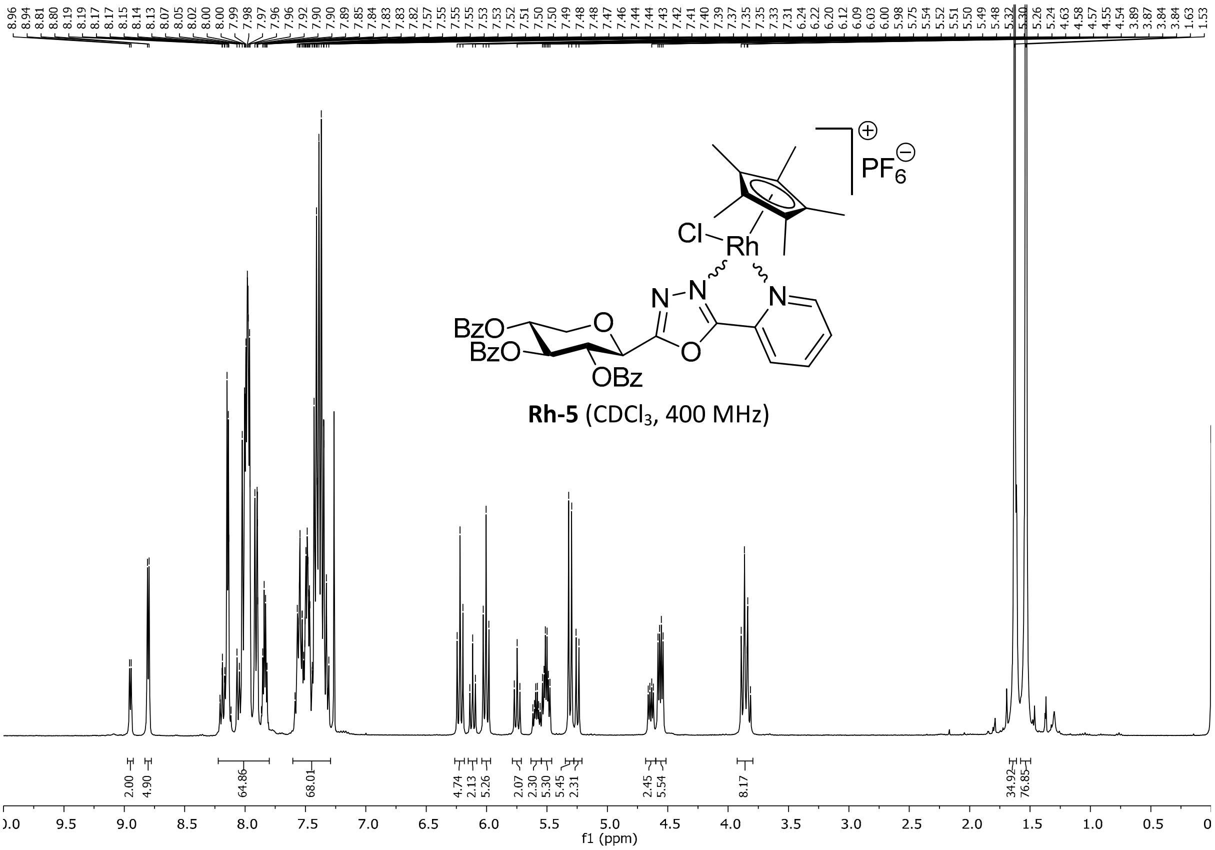
PF6
Cl
Rh
N
N
N
BzO
BzO
O
OBz
O
Rh-5 (CDCl3, 400 MHz)
f1 (ppm)

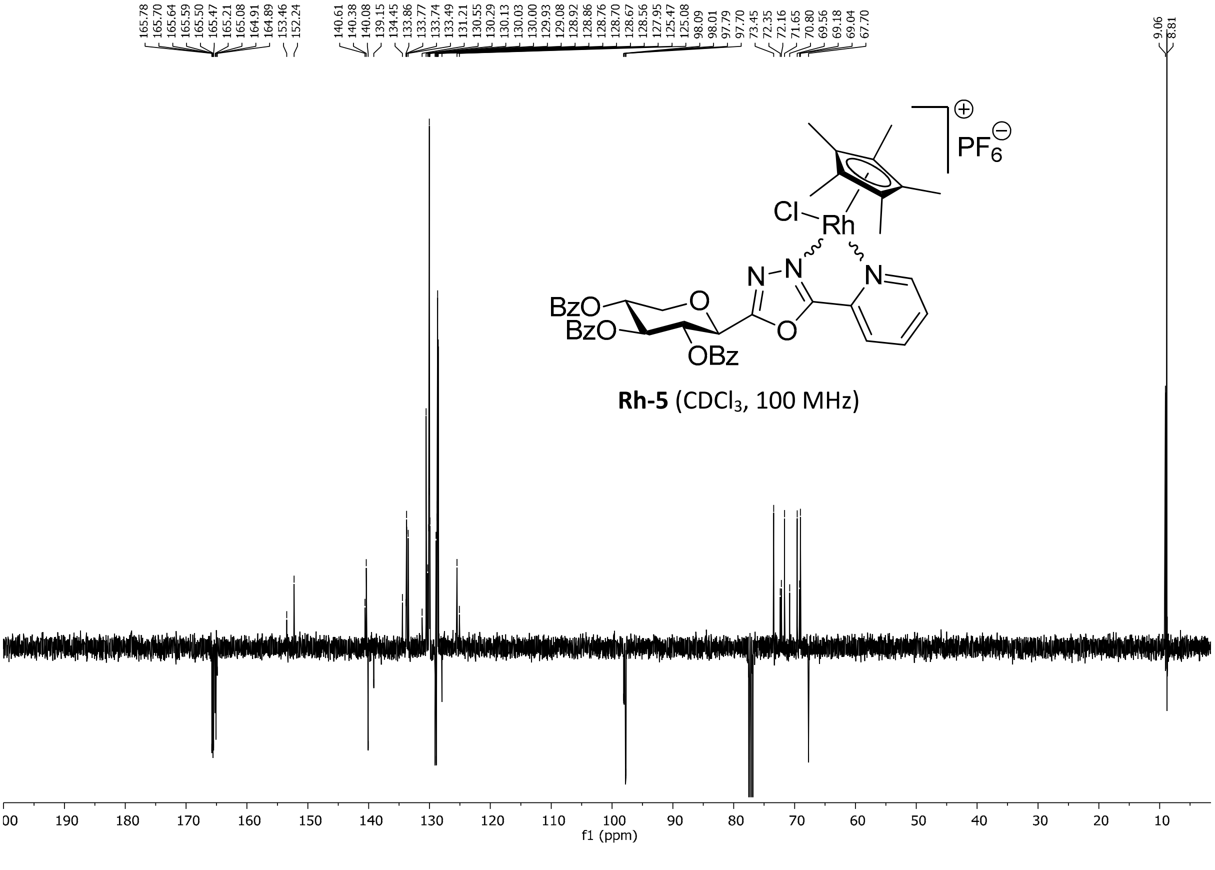
PF6
Cl
Rh
N
N
N
BzO
BzO
O
OBz
O
Rh-5 (CDCl3, 100 MHz)
f1 (ppm)

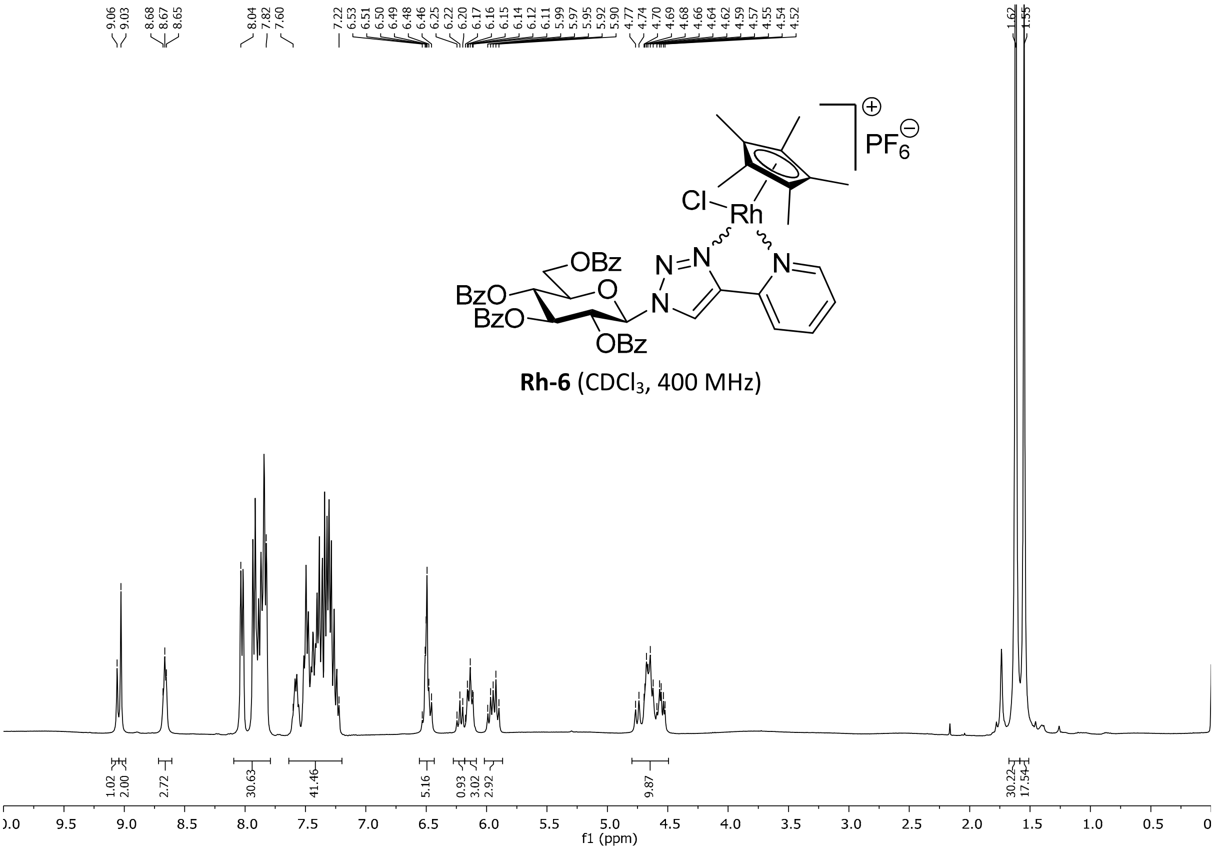

Rh-6 ($CDCl_3$, 400 MHz)

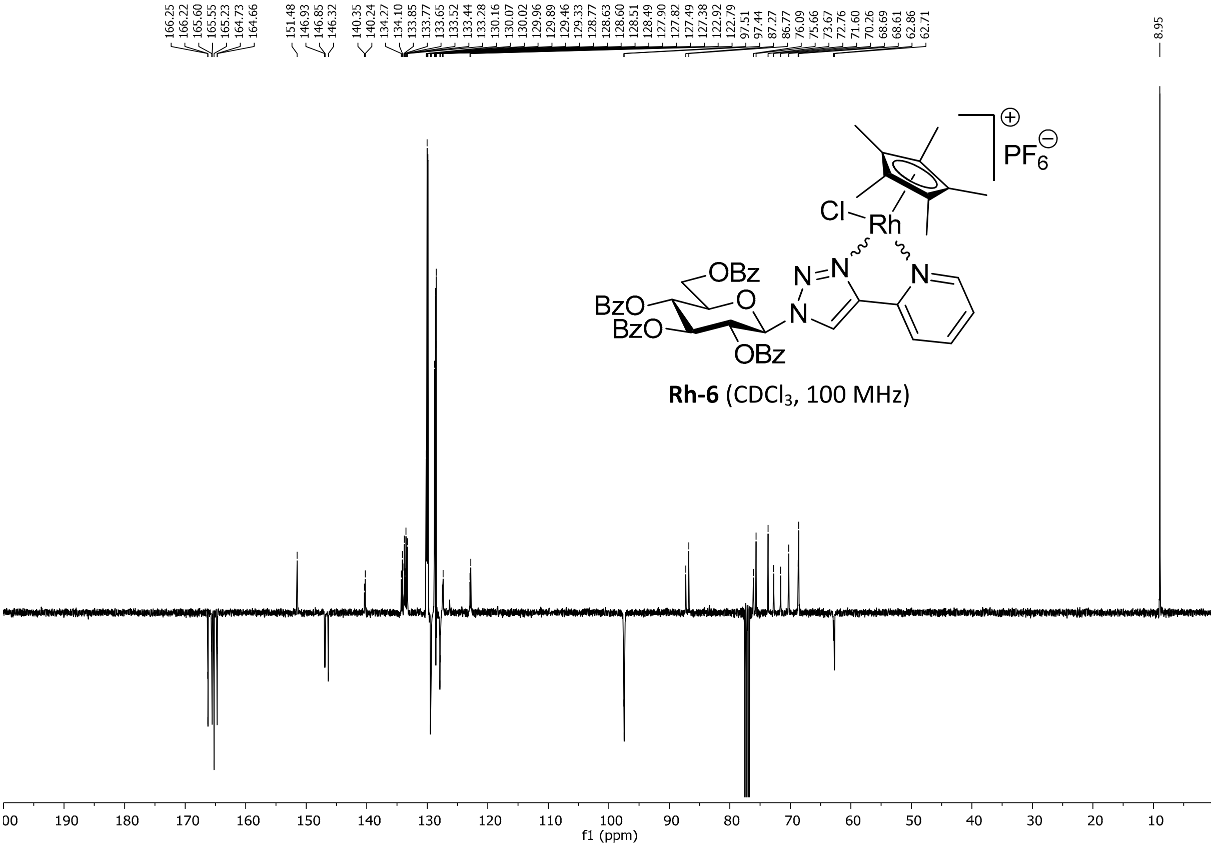

Rh-6 ($CDCl_3$, 100 MHz)

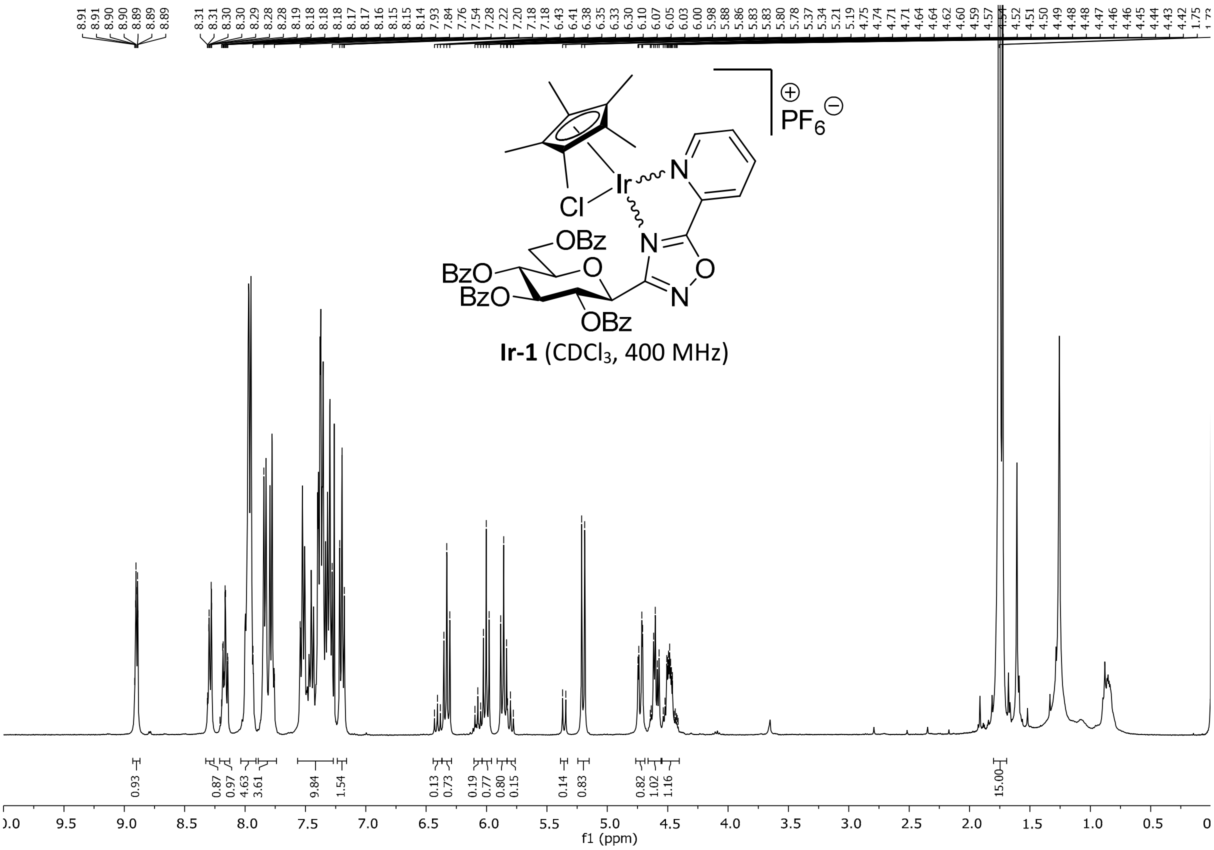
Ir-1 (CDCl3, 400 MHz)
f1 (ppm)

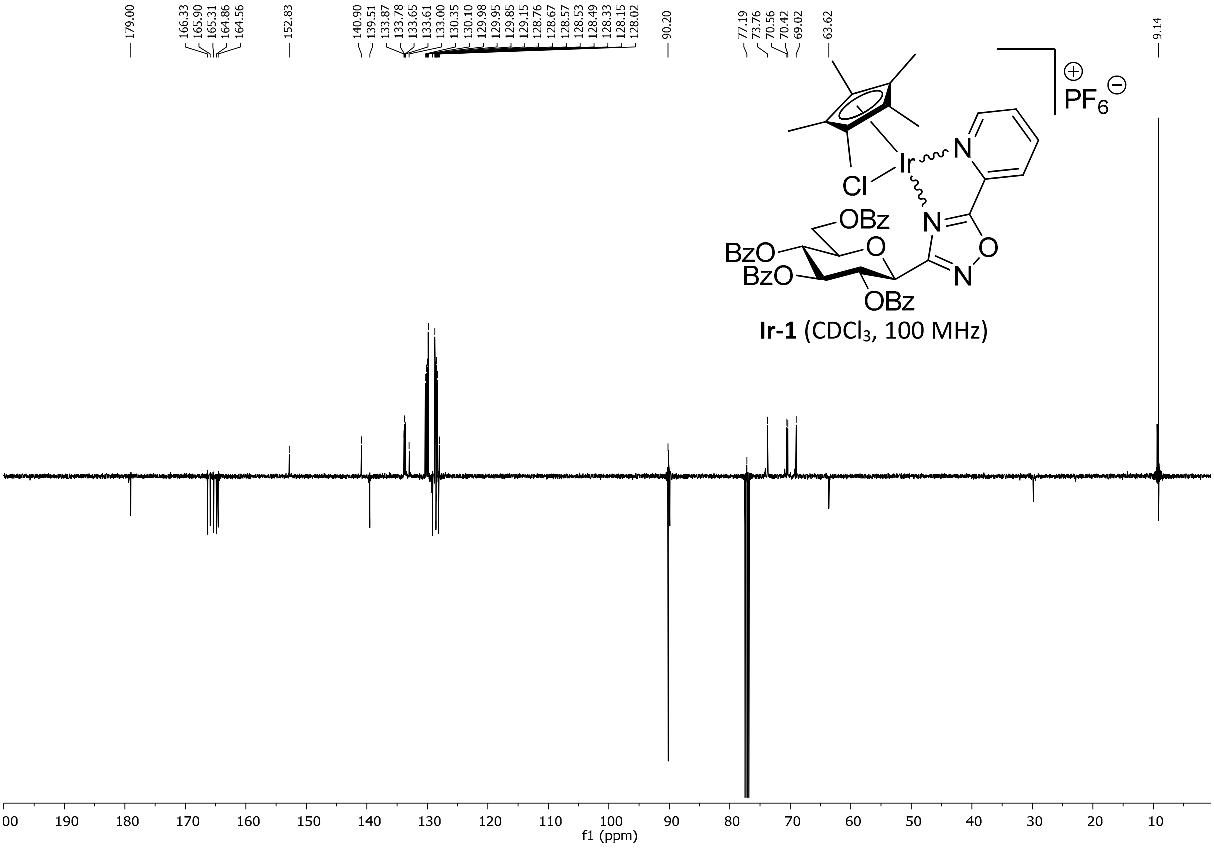
Ir-1 (CDCl3, 100 MHz)
f1 (ppm)

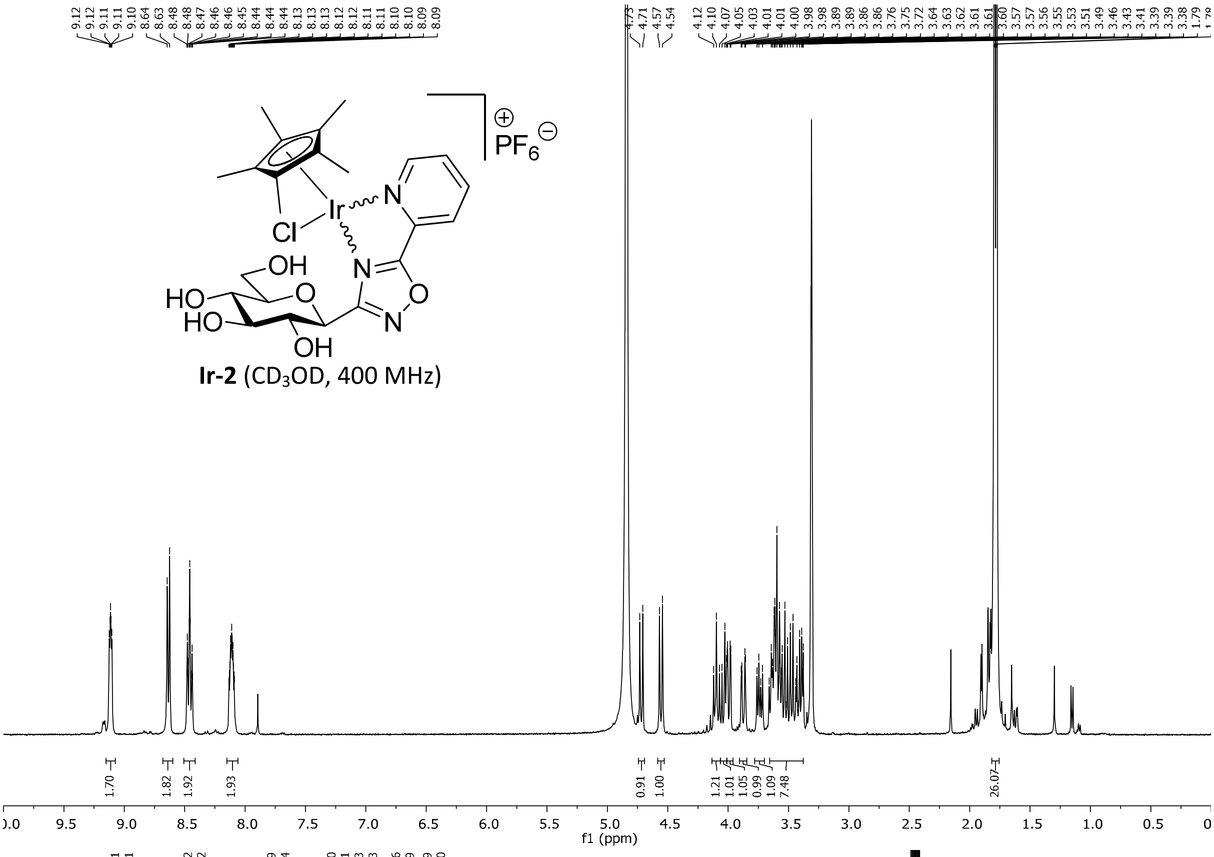

Ir-2 (CD_3OD, 400 MHz)

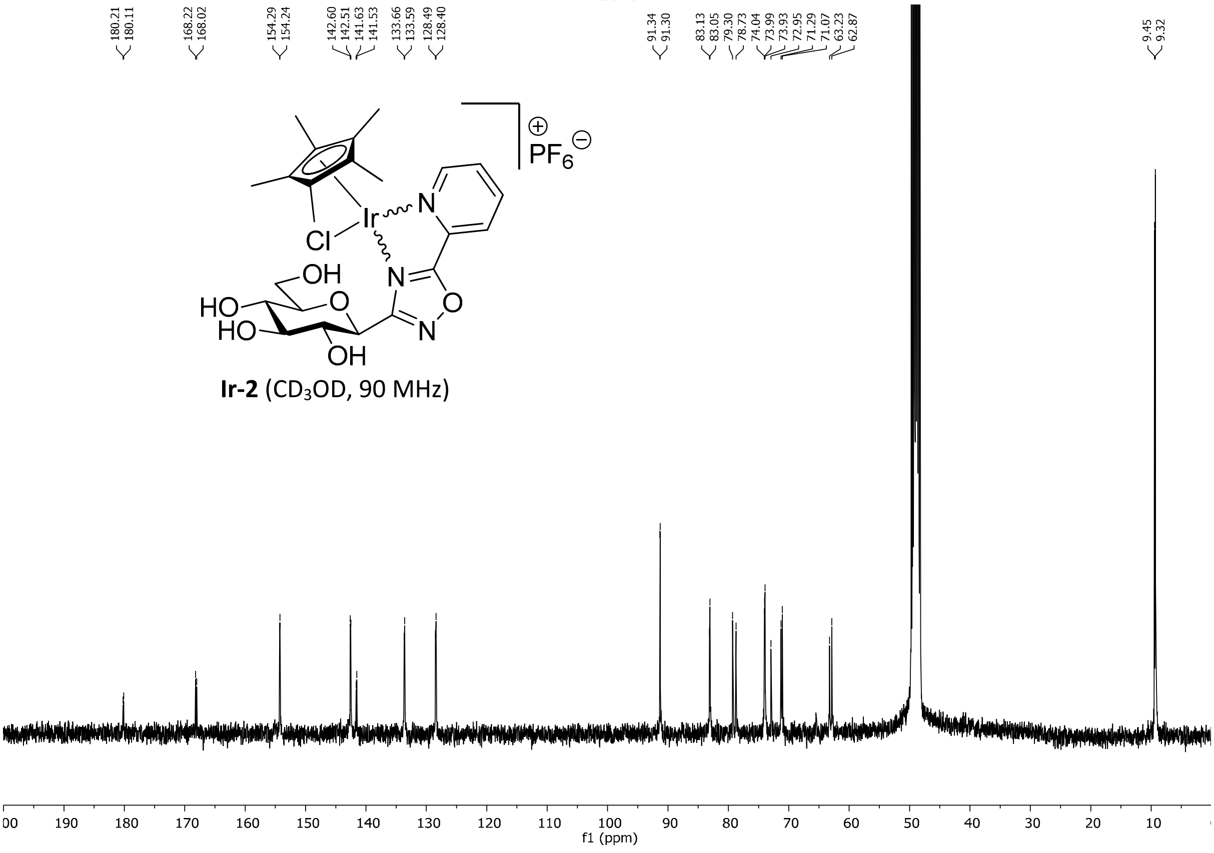

Ir-2 (CD_3OD, 90 MHz)

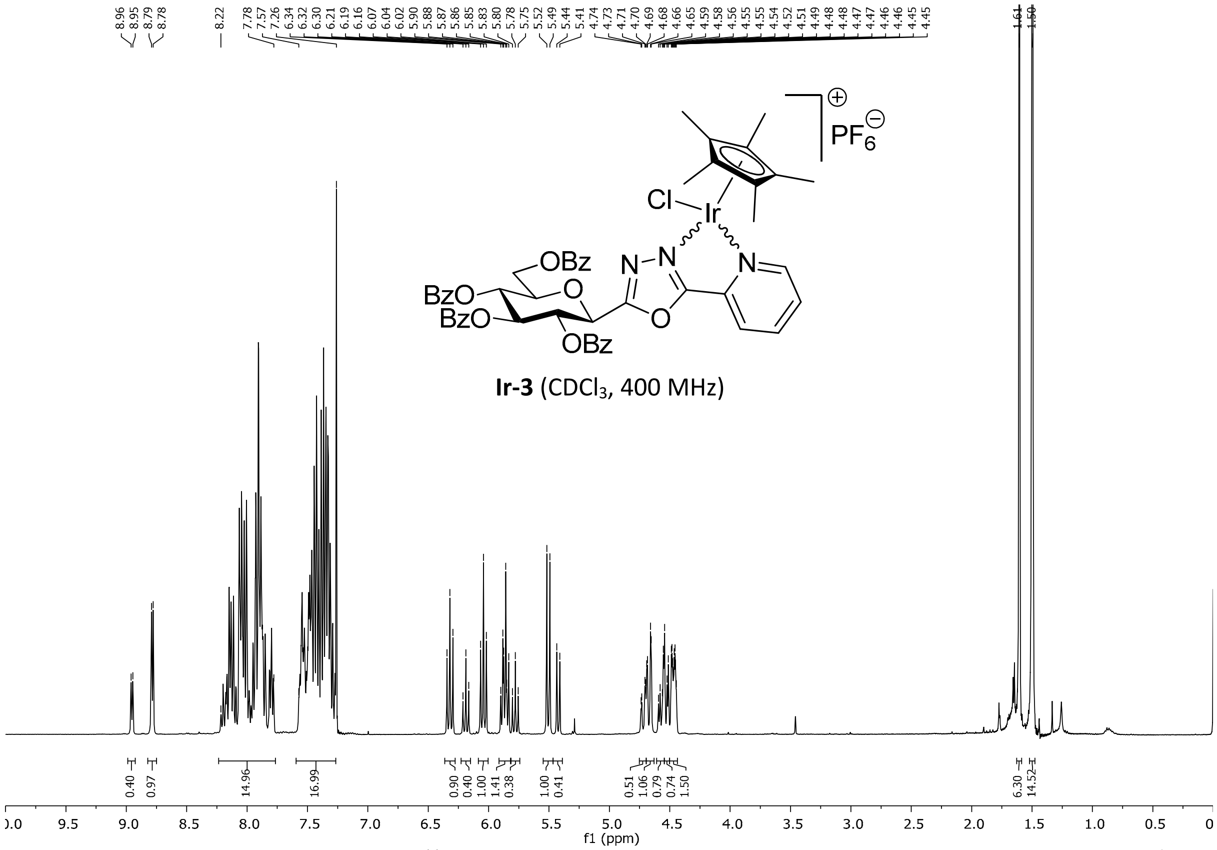
Ir-3 (CDCl$_3$, 400 MHz)

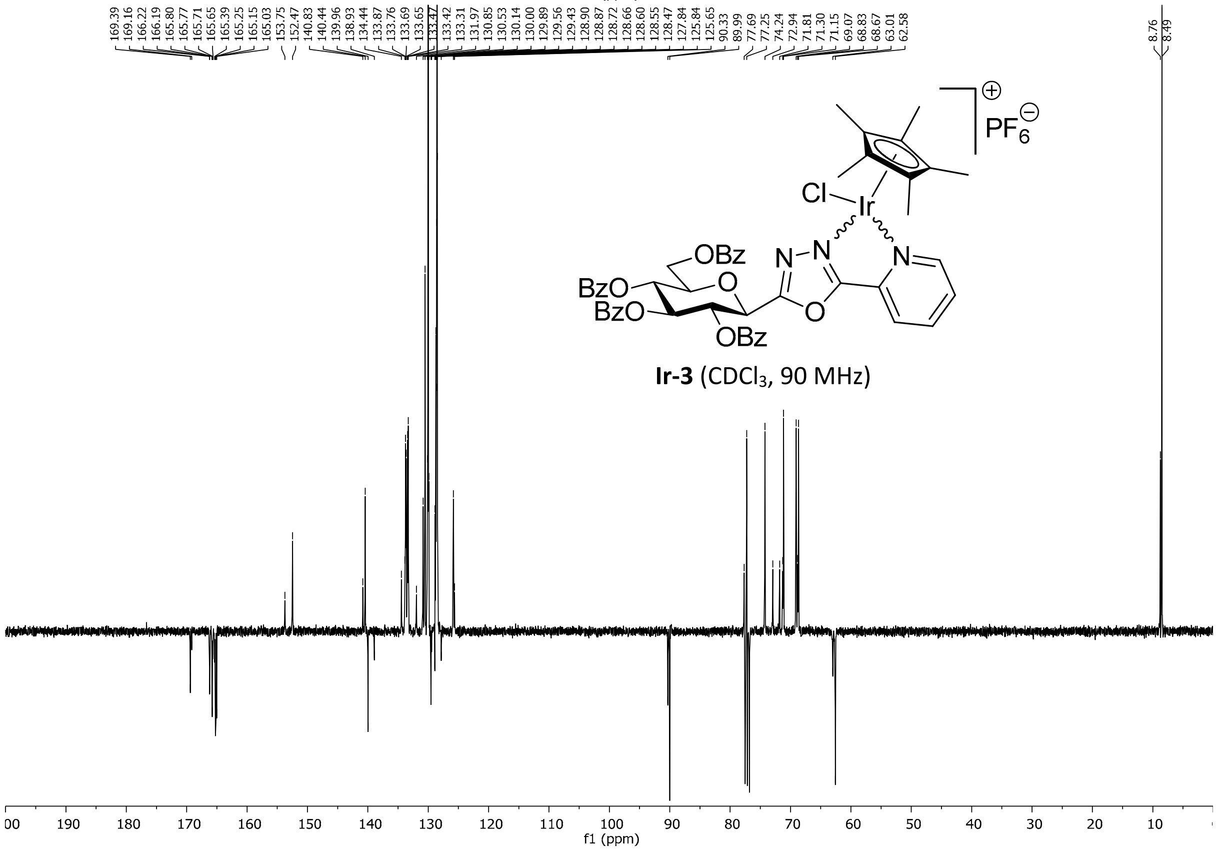
Ir-3 (CDCl$_3$, 90 MHz)

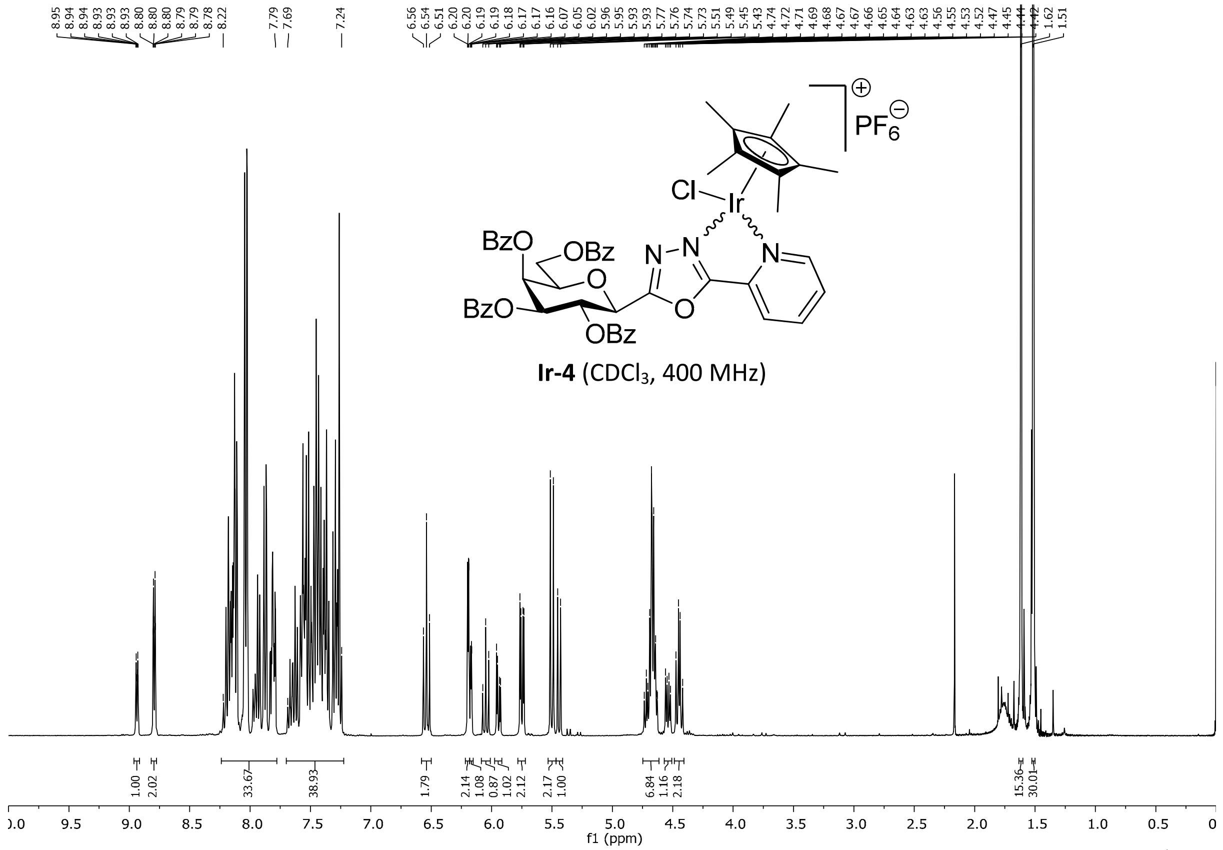
Ir-4 (CDCl3, 400 MHz)
f1 (ppm)

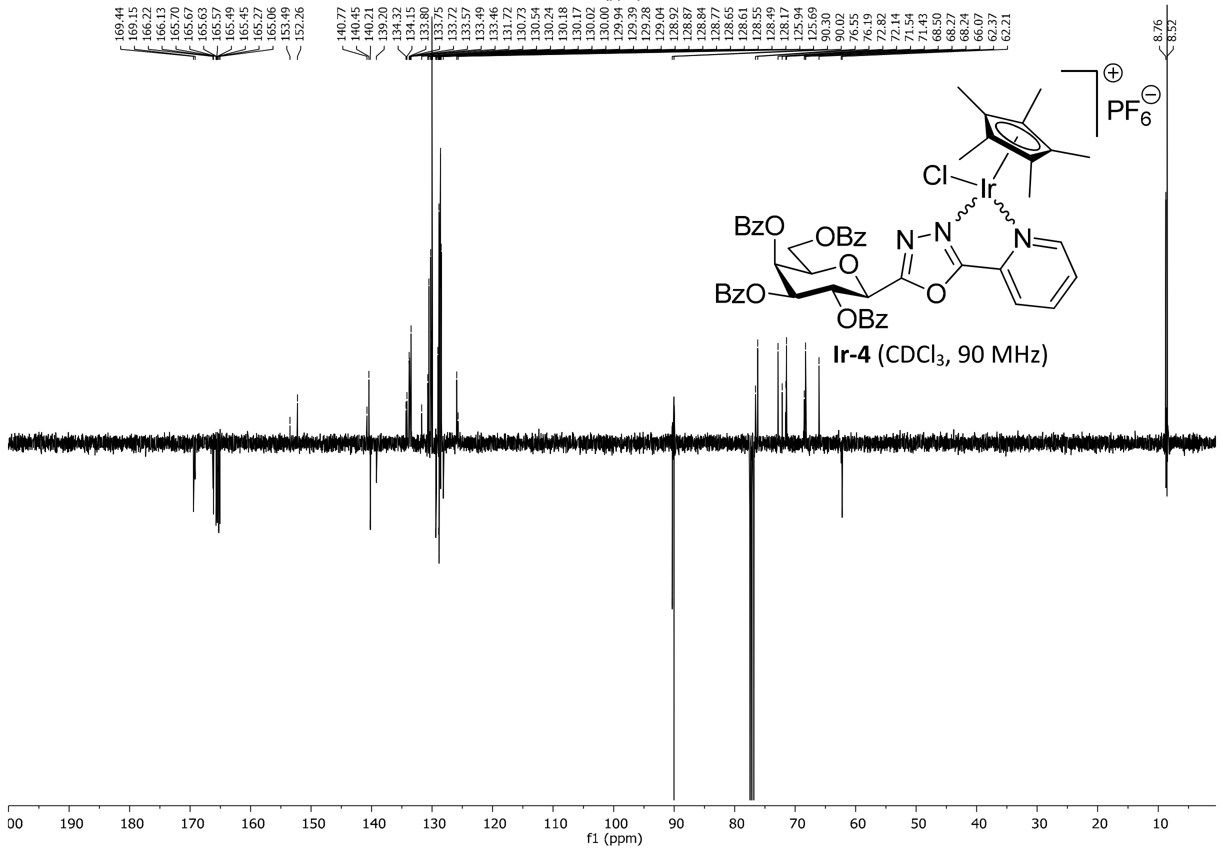
Ir-4 (CDCl3, 90 MHz)
f1 (ppm)

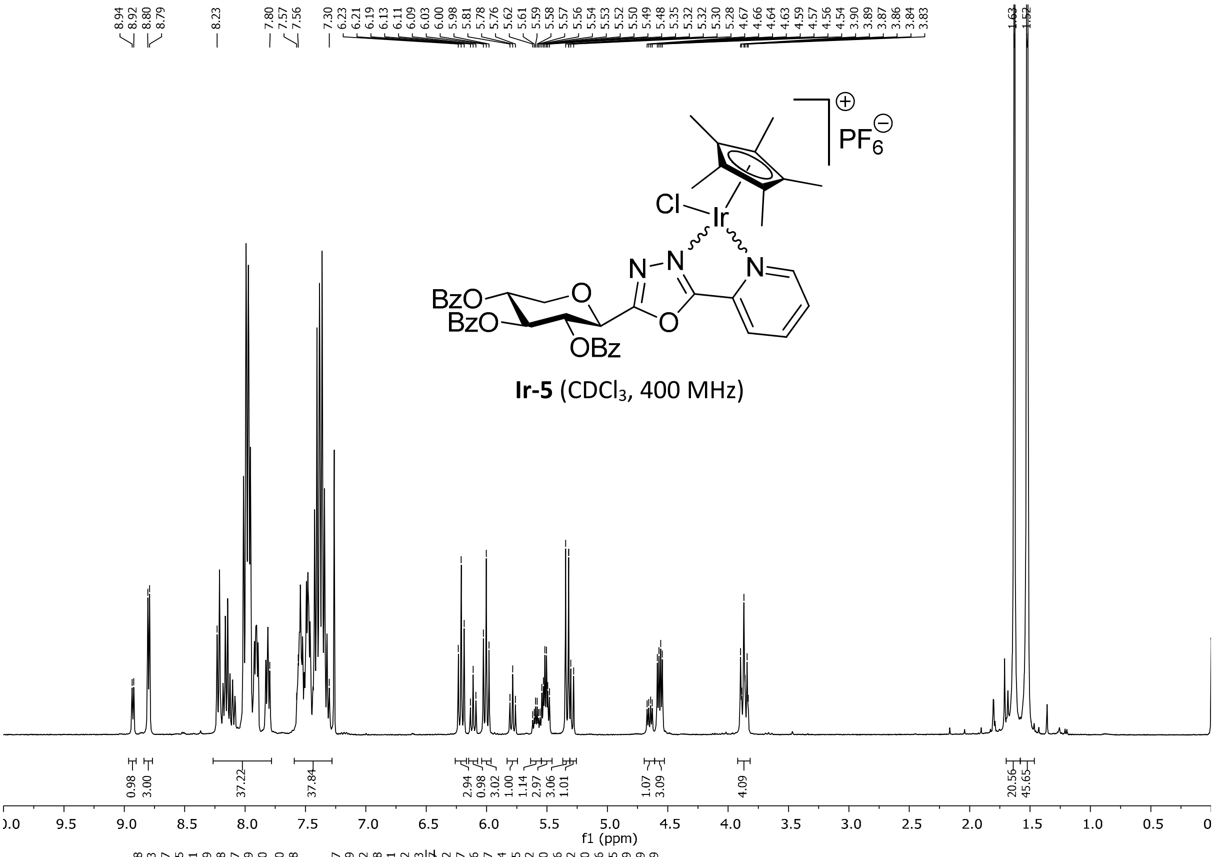

Ir-5 (CDCl$_3$, 400 MHz)

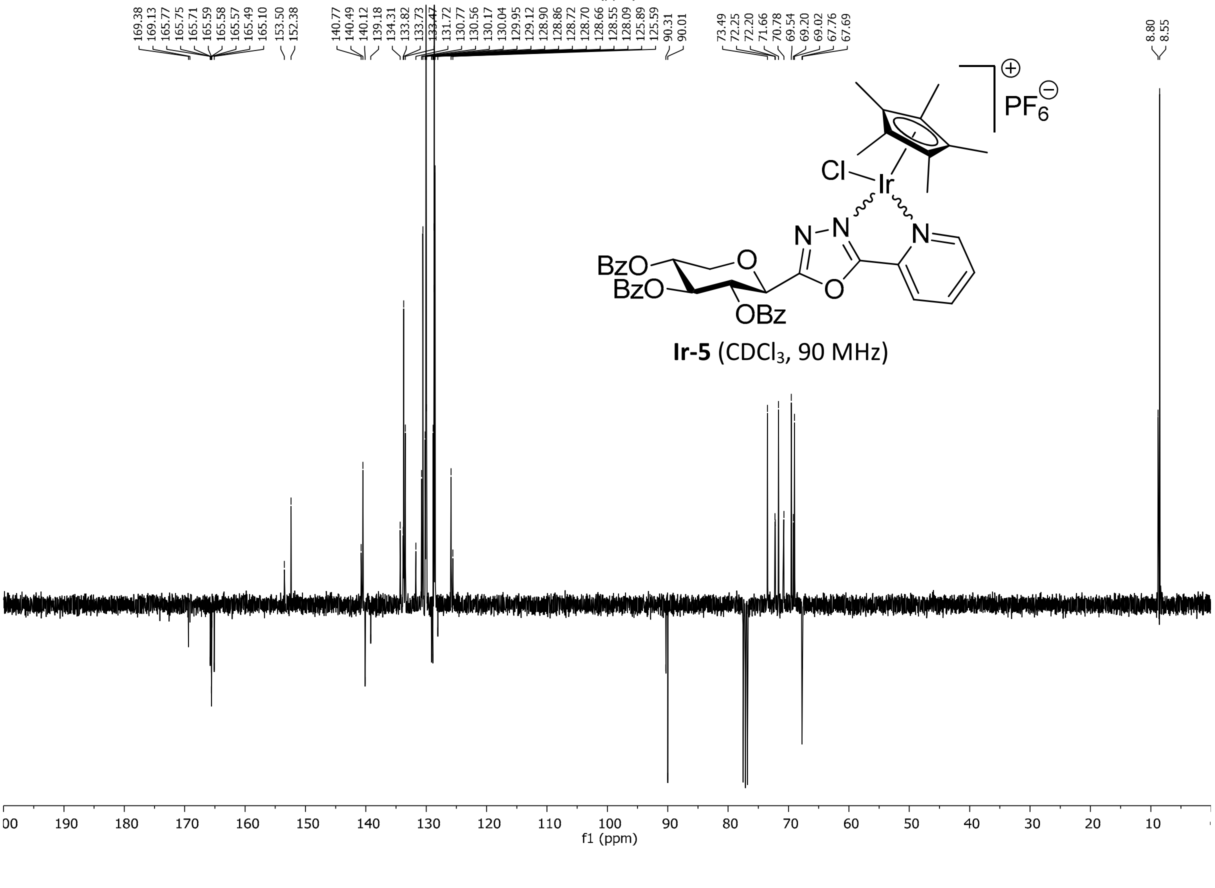

Ir-5 (CDCl$_3$, 90 MHz)

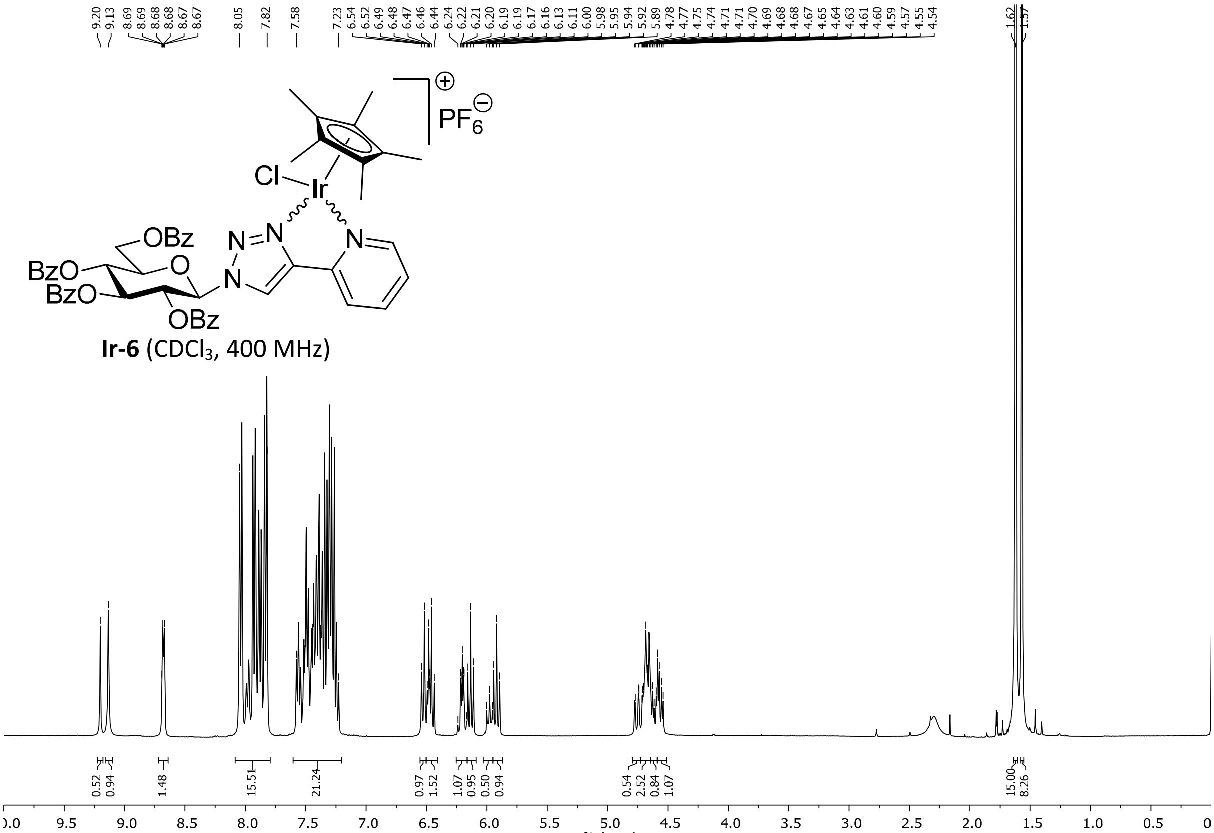
9.20 9.13 8.69 8.69 8.68 8.68 8.67 8.67 8.05 7.82 7.58 7.23 6.54 6.52 6.49 6.48 6.47 6.46 6.44 6.24 6.22 6.21 6.20 6.19 6.19 6.17 6.16 6.13 6.11 6.00 5.98 5.95 5.94 5.92 5.89 4.78 4.77 4.75 4.74 4.71 4.71 4.70 4.69 4.68 4.68 4.67 4.65 4.64 4.63 4.61 4.60 4.59 4.57 4.55 4.54 1.62 1.57
Cl
Ir
PF6
N
N
N
N
O
OBz
BzO
BzO
OBz
Ir-6 (CDCl3, 400 MHz)
0.52 0.94 1.48 15.51 21.24 0.97 1.52 1.07 0.95 0.50 0.94 0.54 2.52 0.84 1.07 15.00 8.26
f1 (ppm)

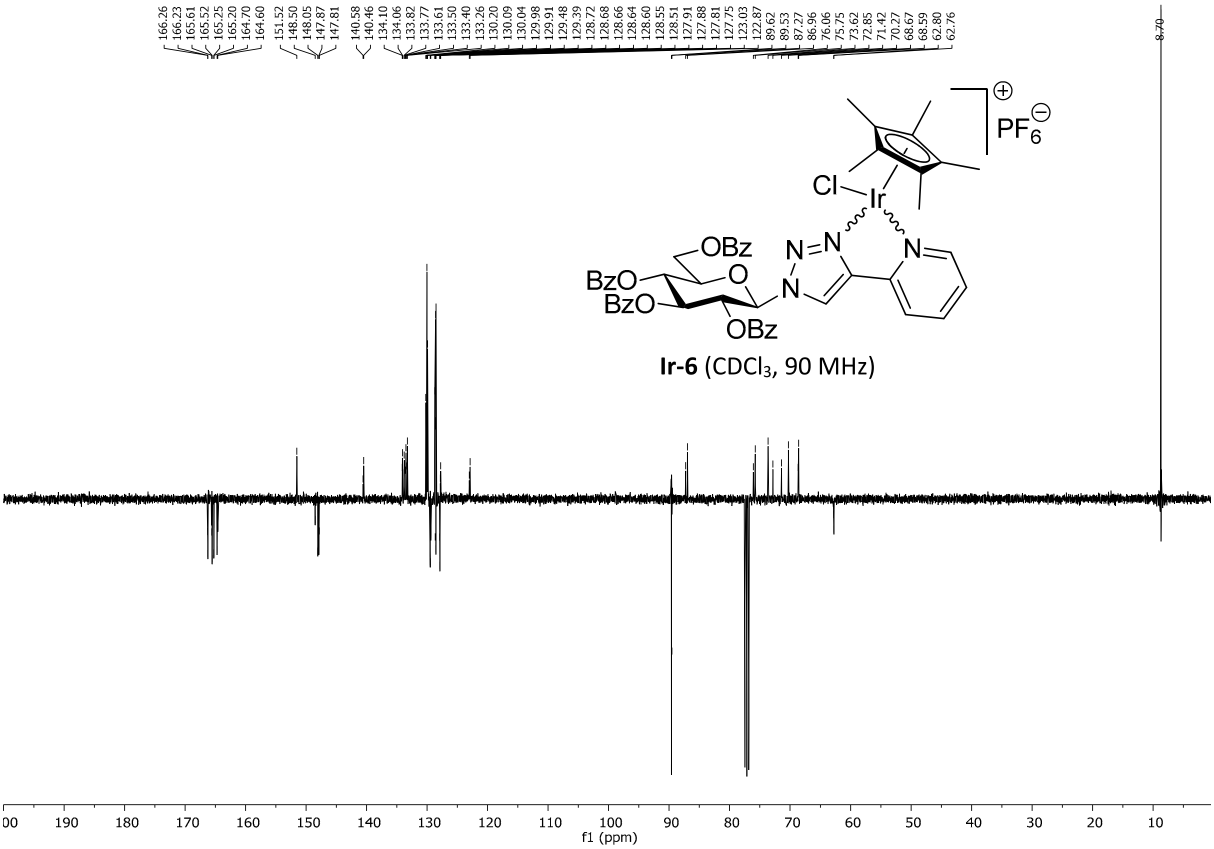
166.26 166.23 165.61 165.52 165.25 165.20 164.70 164.60 151.52 148.50 148.05 147.87 147.81 140.58 140.46 134.10 134.06 133.82 133.77 133.61 133.50 133.40 133.26 130.20 130.09 130.04 129.98 129.91 129.48 129.39 128.72 128.68 128.66 128.64 128.60 128.55 128.51 127.91 127.88 127.81 127.75 123.03 122.87 89.62 89.53 87.27 86.96 76.06 75.75 73.62 72.85 71.42 70.27 68.67 68.59 62.80 62.76 8.70
Cl
Ir
PF6
N
N
N
N
O
OBz
BzO
BzO
OBz
Ir-6 (CDCl3, 90 MHz)
f1 (ppm)

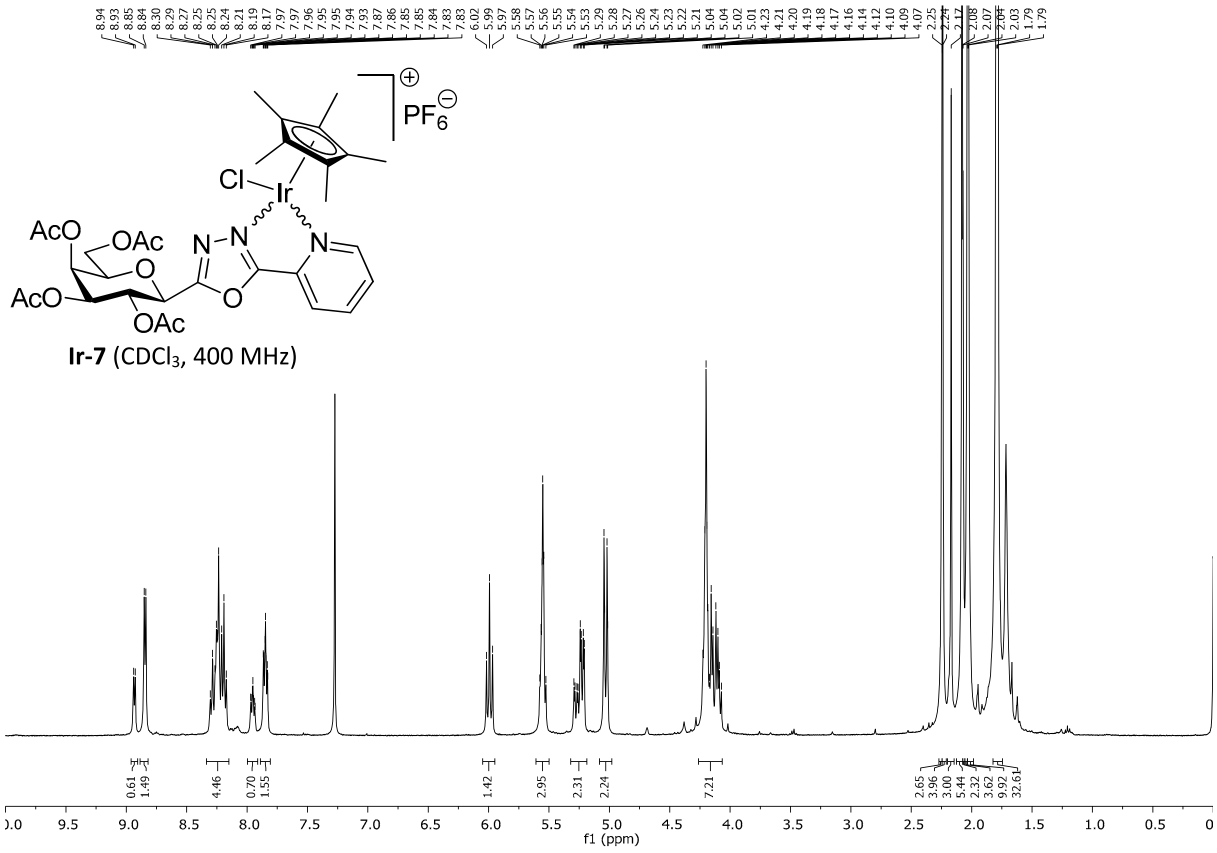

AcO
OAc
AcO
OAc
Cl
Ir
PF6
Ir-7 (CDCl3, 400 MHz)
f1 (ppm)

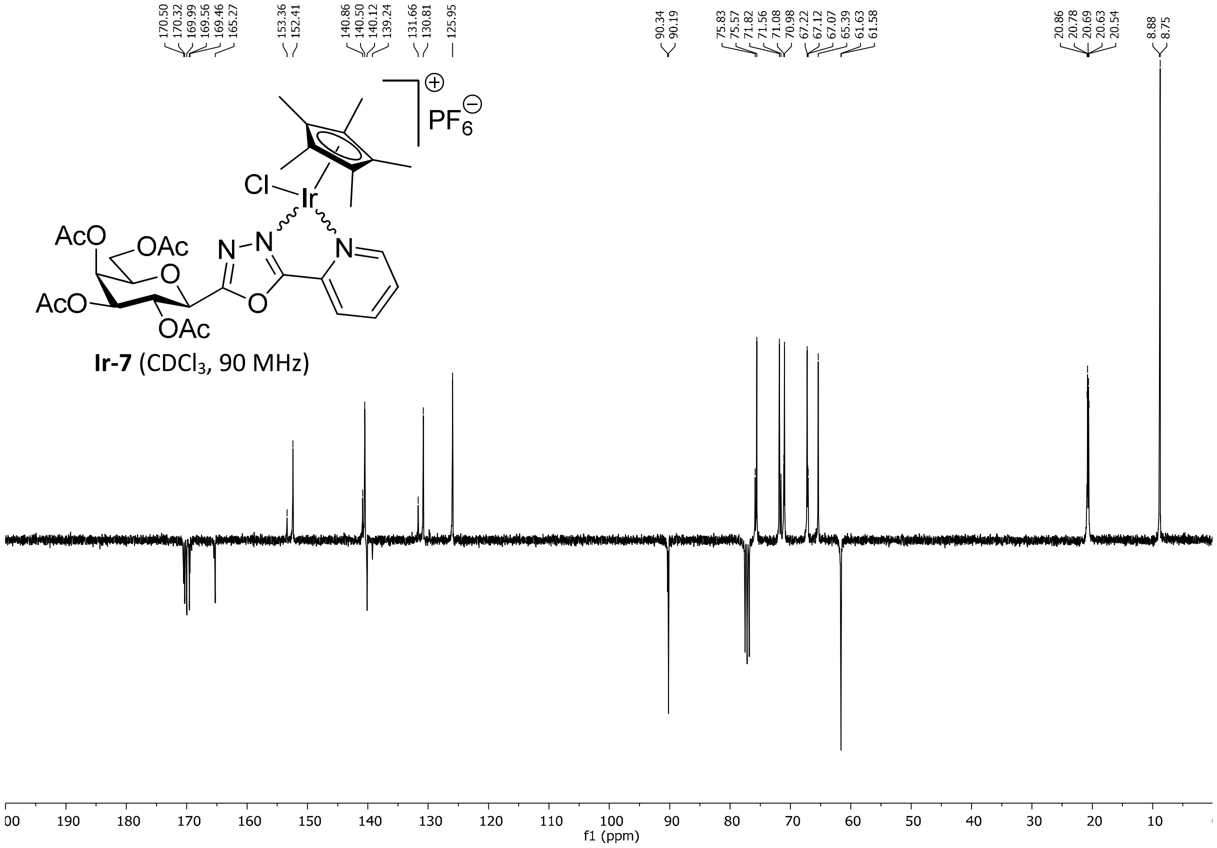

AcO
OAc
AcO
OAc
Cl
Ir
PF6
Ir-7 (CDCl3, 90 MHz)
f1 (ppm)

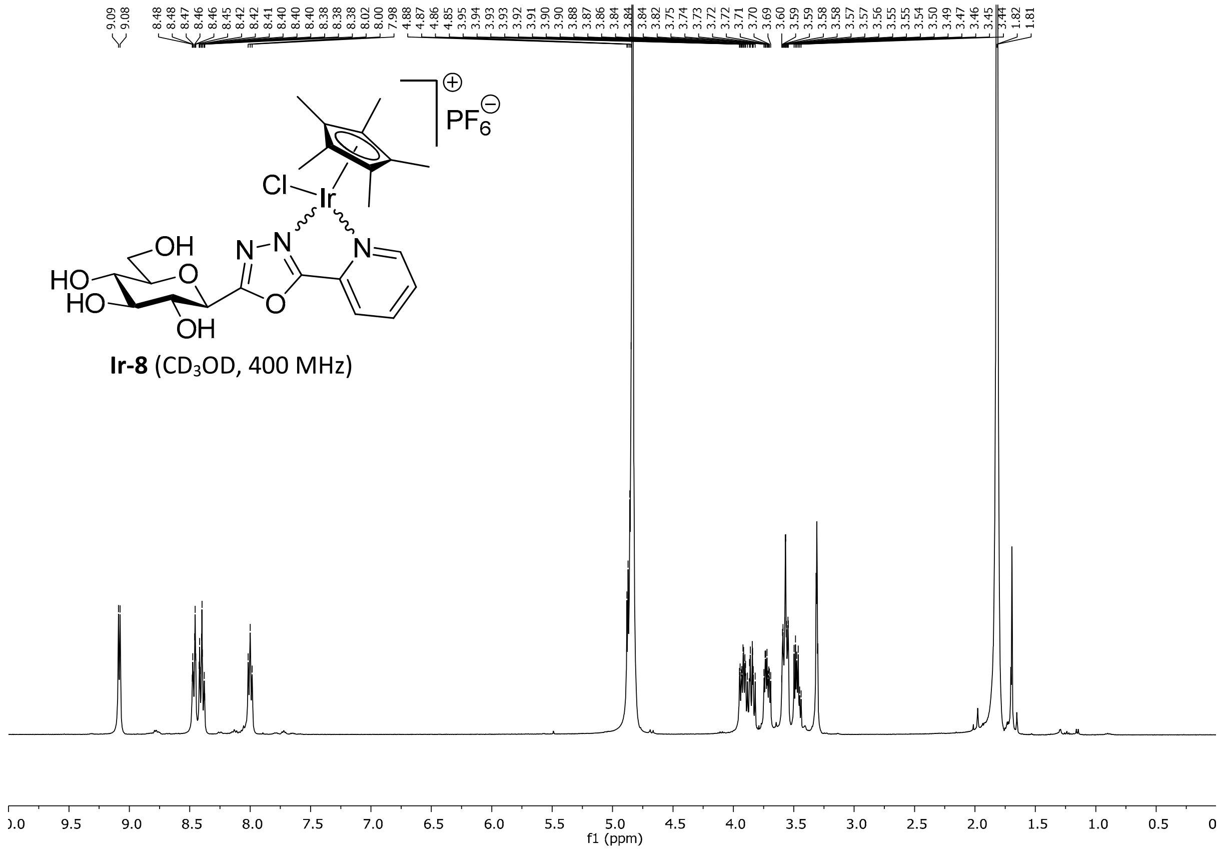

Ir-8 (CD_3OD, 400 MHz)

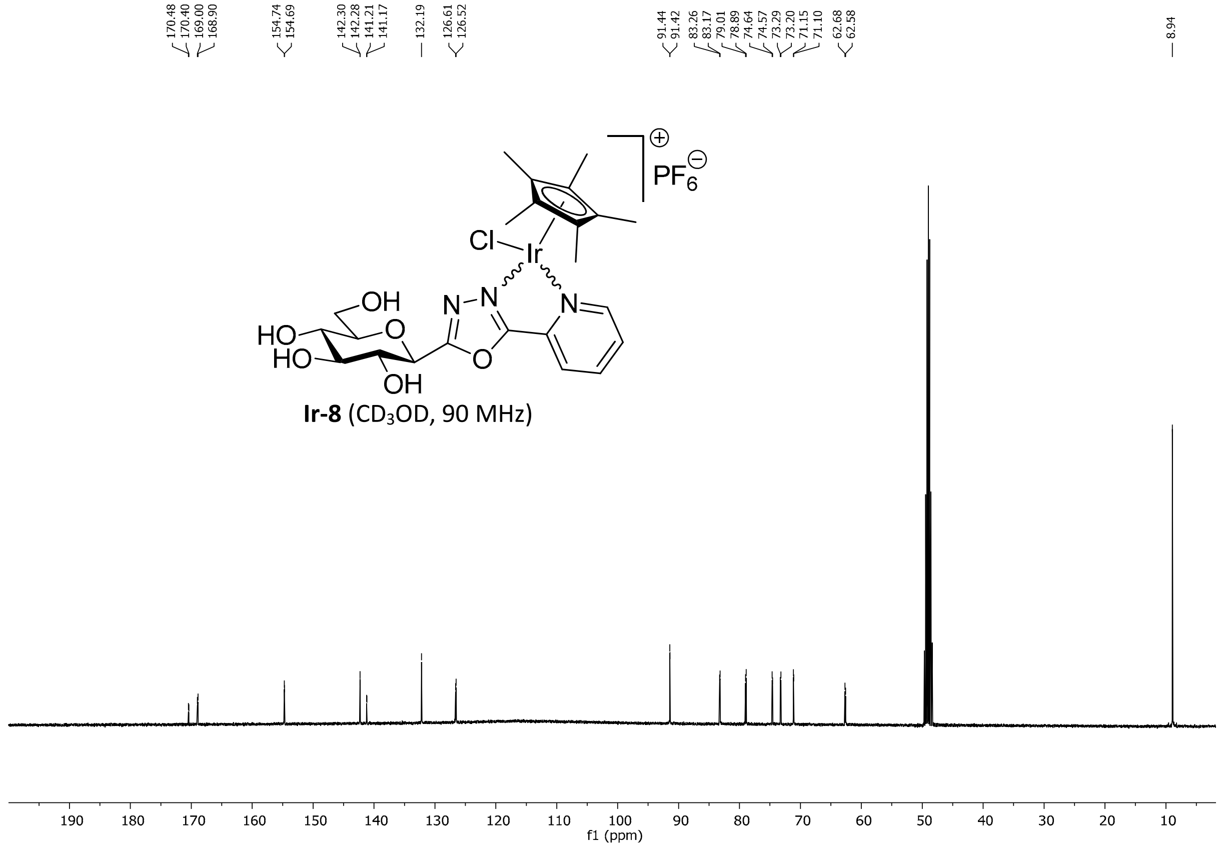

Ir-8 (CD_3OD, 90 MHz)

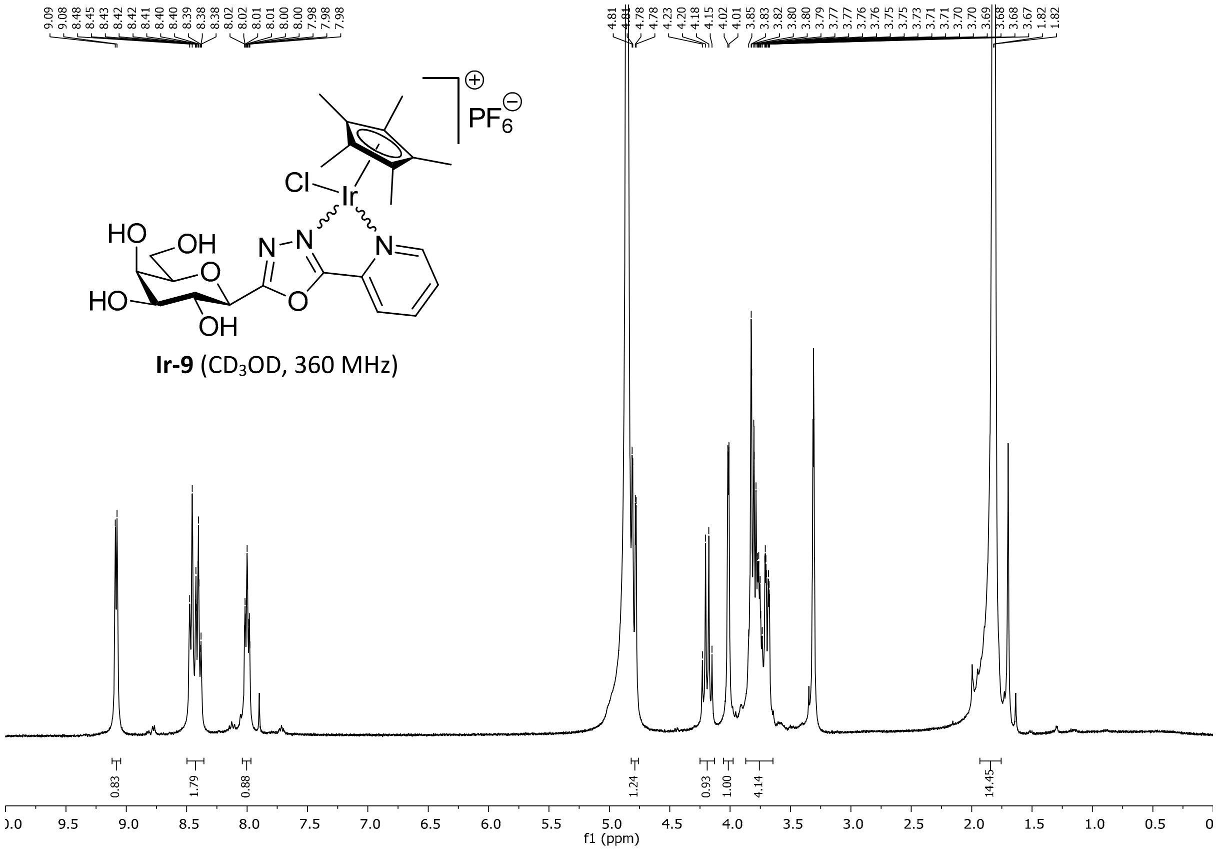

Ir-9 (CD_3OD, 360 MHz)

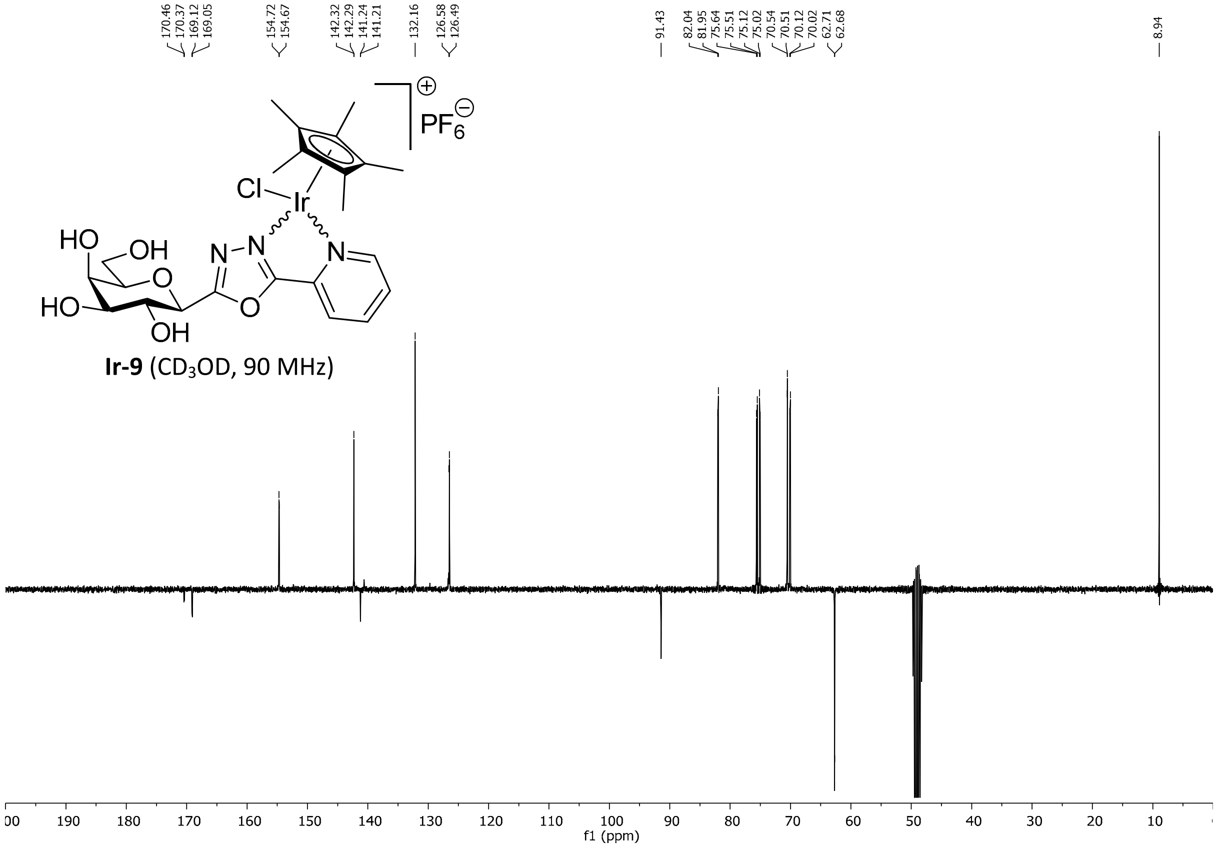

Ir-9 (CD_3OD, 90 MHz)

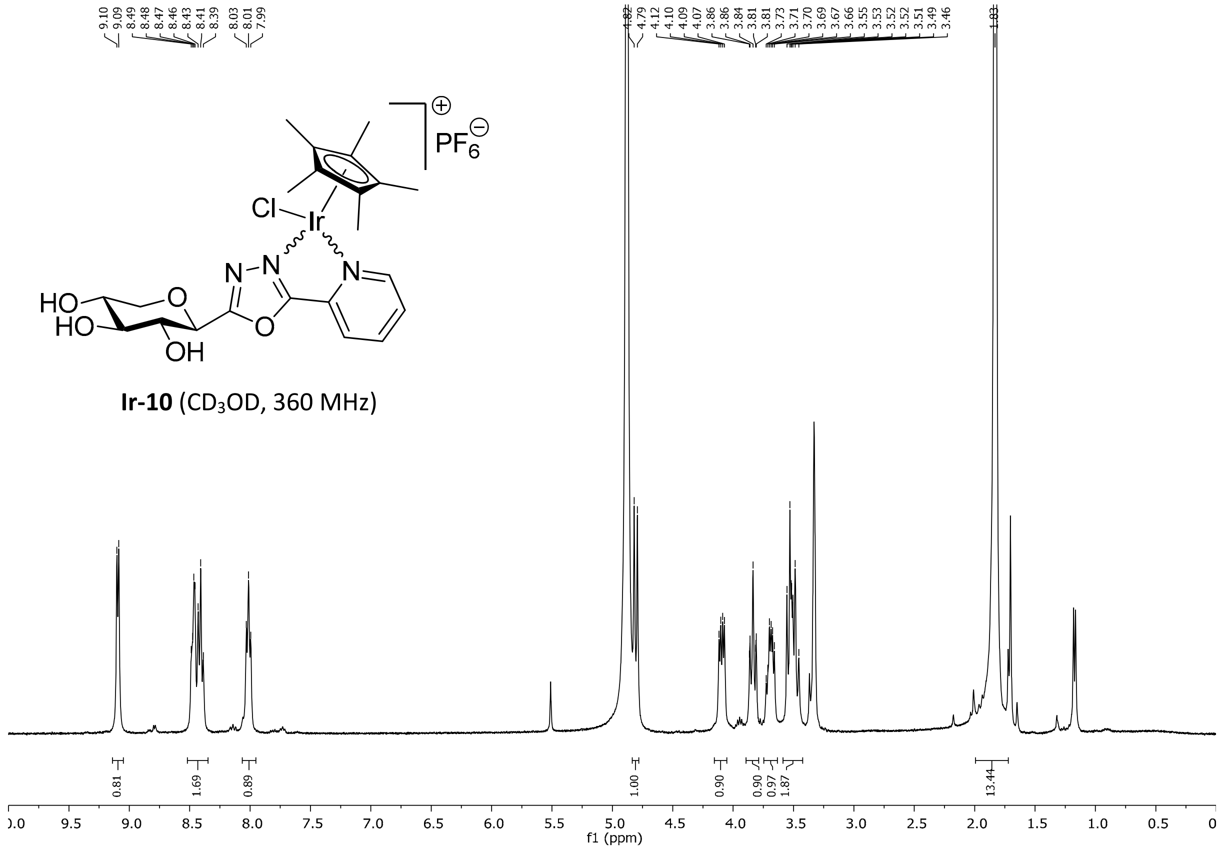

Ir-10 (CD$_3$OD, 360 MHz)

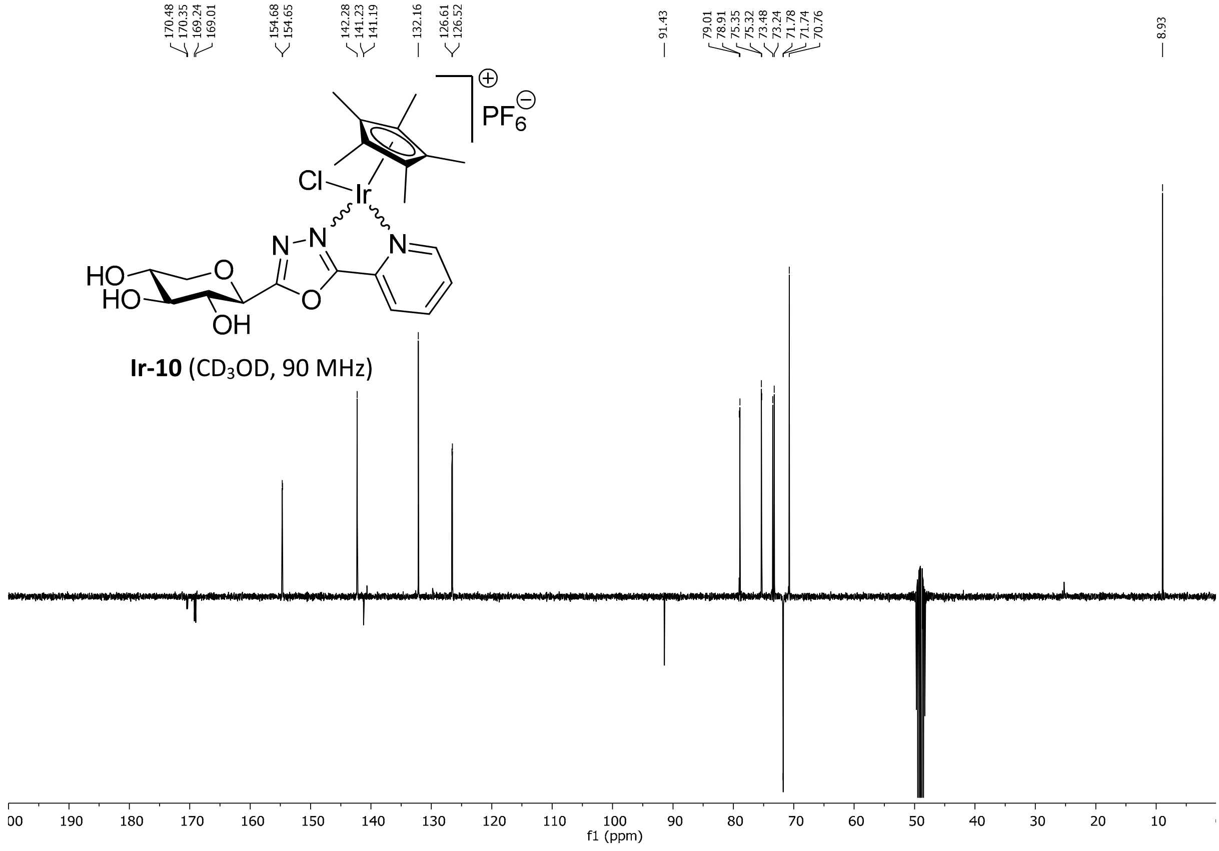

Ir-10 (CD$_3$OD, 90 MHz)

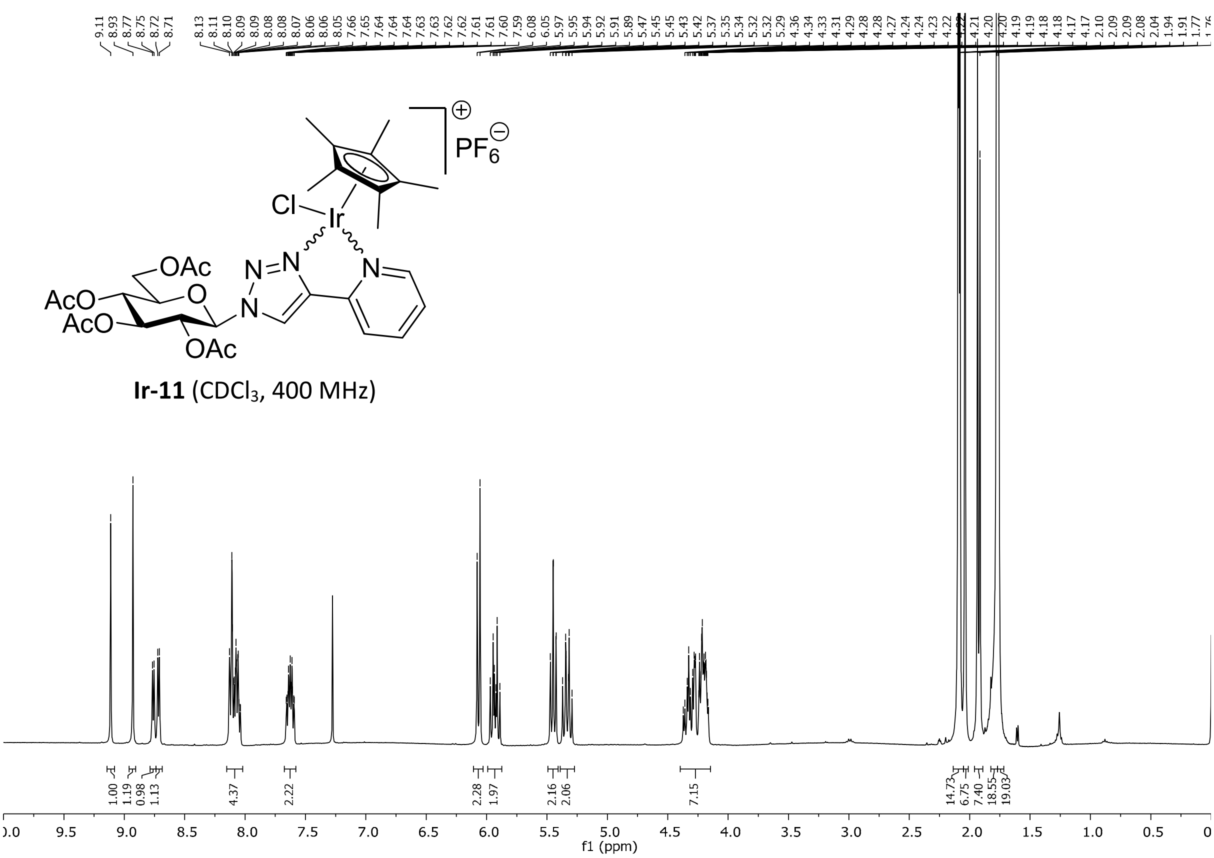

Ir-11 ($CDCl_3$, 400 MHz)

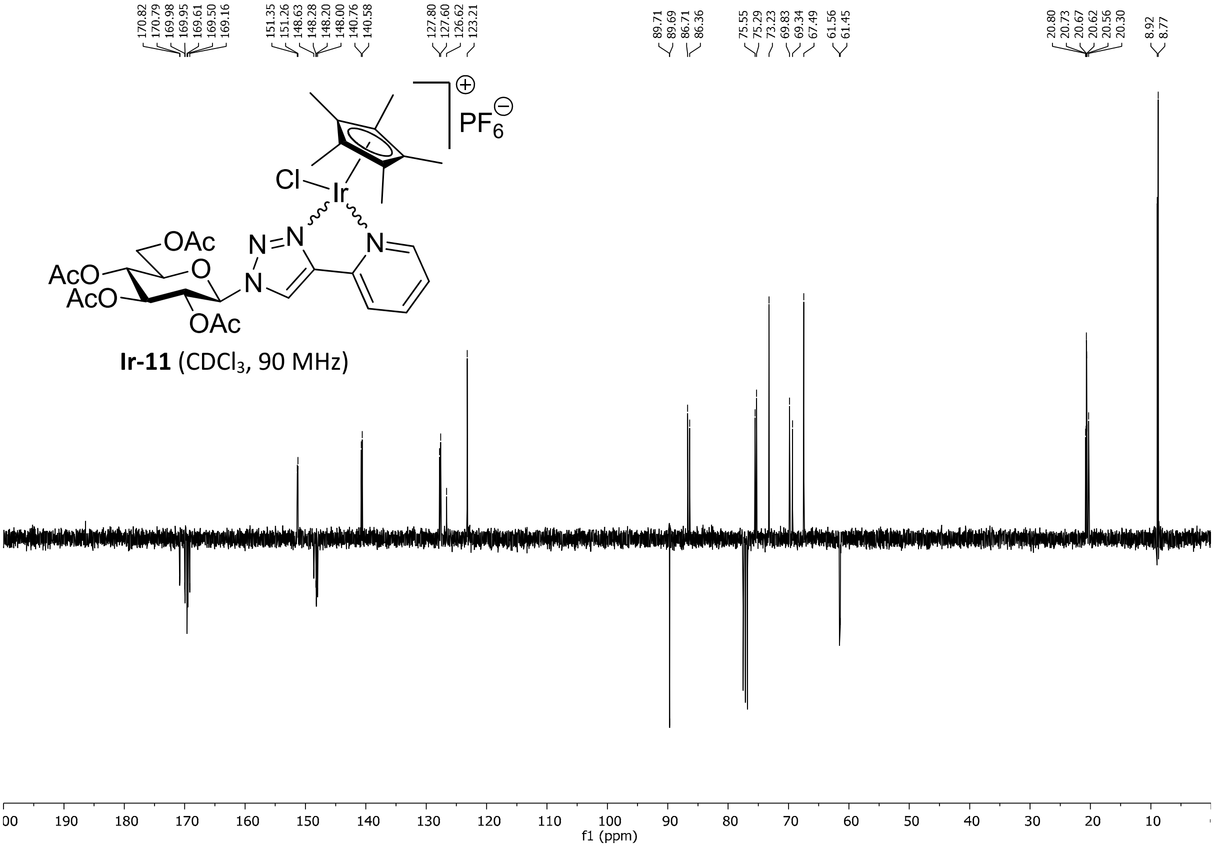

Ir-11 ($CDCl_3$, 90 MHz)

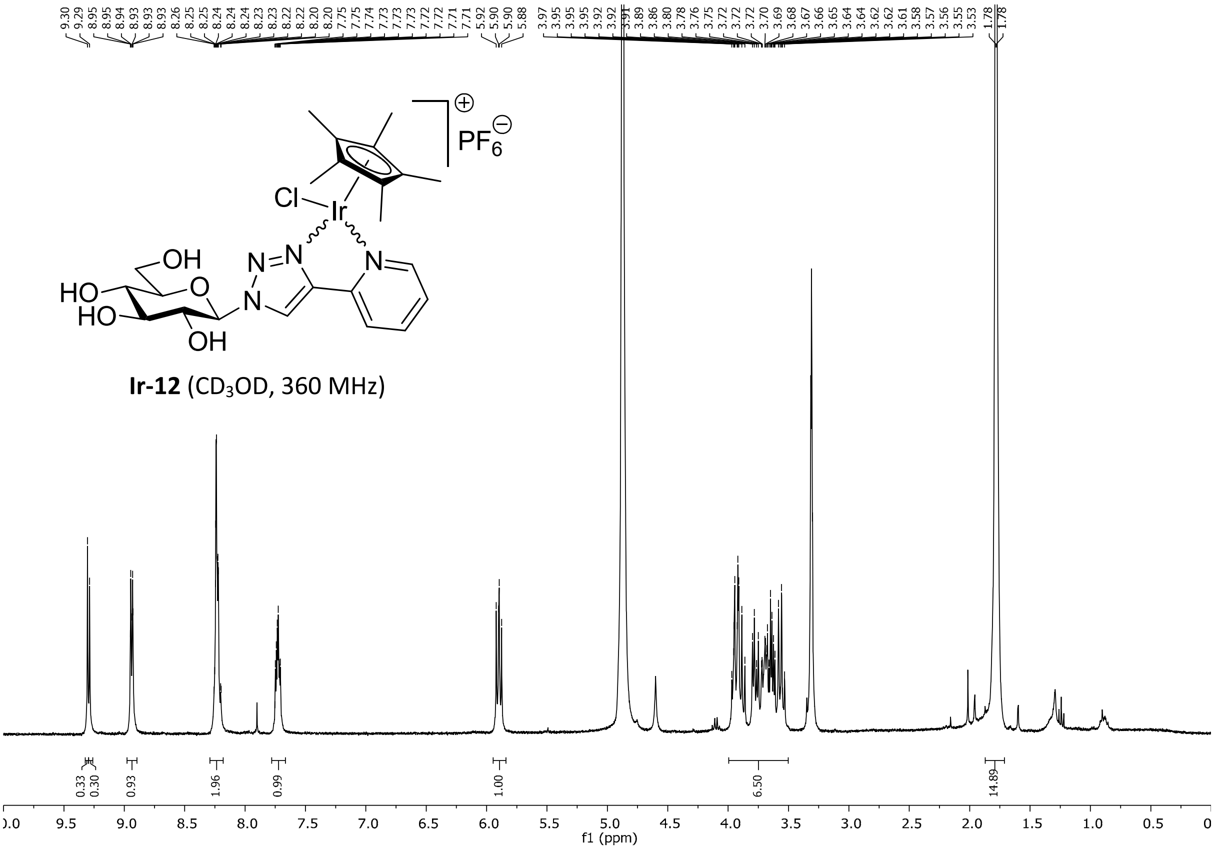

Ir-12 (CD_3OD, 360 MHz)

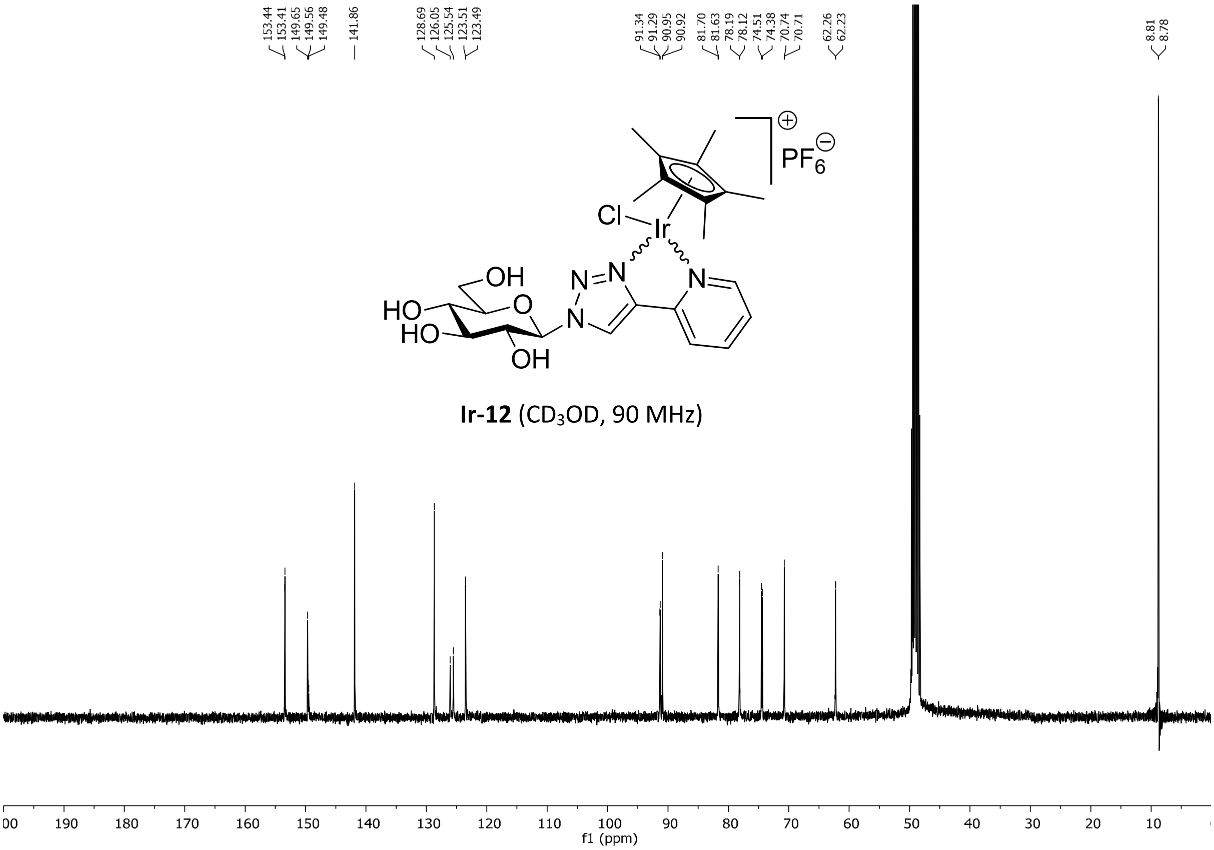

Ir-12 (CD_3OD, 90 MHz)

2. X-Ray diffraction study

X-ray-quality crystals were grown by slow evaporation of the corresponding mother liquors (**Ru-1** from $CHCl_3$, **Ir-2** from MeOH). A crystal well-looking in polarized light microscope was fixed under a microscope onto a Mitegen loop using high-density oil. Diffraction intensity data were collected at room temperature (295-300 K) using a Bruker-D8 Venture diffractometer (Bruker AXS GmbH, Karlsruhe, Germany) equipped with INCOATEC IμS 3.0 (Incoatec GmbH, Geesthacht, Germany) dual (Cu and Mo) sealed tube micro sources and a Photon II Charge-Integrating Pixel Array detector (Bruker AXS GmbH, Karlsruhe, Germany) using Mo Kα (λ = 0.71073 Å) radiation. High multiplicity data collection and integration were performed using APEX3 (version 2017.3-0, Bruker AXS Inc., 2017, Madison, USA) software. Data reduction and multiscan absorption correction were performed using SAINT (version 8.38A, Bruker AXS Inc., 2017, Madison, USA). The structure was solved using direct methods and refined on F^2 using the SHELXL program (Sheldrick 2008) incorporated into the APEX3 suite. Refinement was performed anisotropically for all nonhydrogen atoms. Hydrogen atoms were placed into geometric positions except the protons of alcoholic OHs in Ir-2 which could be found at the difference electron density map and O-H distance should be constrained. The CIF file was manually edited using Publcif software (Westrip 2010), while graphics were prepared using the Mercury program (Macrae, Edgington et al. 2006).

The structures are stabilized by strong hydrogen bonds between the hydroxyl protons and fluorine in **Ir-2** and weak C-H..O, C-H..Cl and C-H..F hydrogen bonds in **Ru-1**. Packing diagrams are shown in Figures S4 and S5, while geometric parameters in Table S1 and Table S3. Hydrogen bond geometry is compiled in Tables S2 and S4.

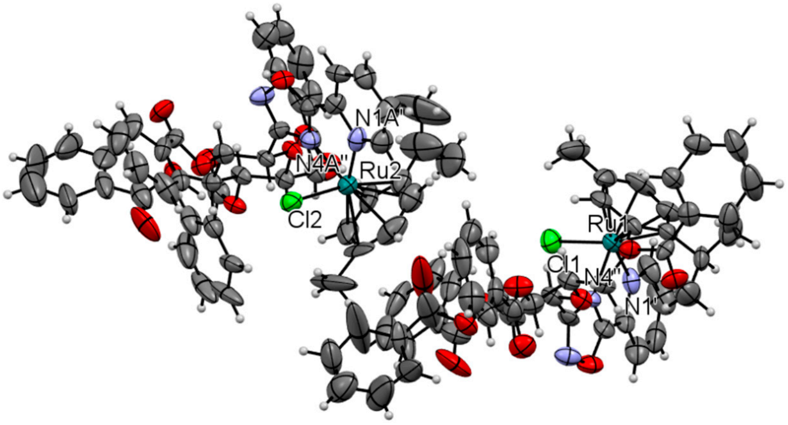

Figure S1. Ortep view of compex **Ru-1** at 50 % probability level. The counter ion and solvent molecules are not shown for clarity. Key bond lengths [Å] and angles [°]: N1'—Ru1 2.118 (14), N4"—Ru1 2.099 (11), Cl1—Ru1 2.377 (5), N4"—Ru1—N1' 77.1 (5), N4"—Ru1—Cl1 84.0 (4), N1'—Ru1—Cl1 83.9 (4), N1A'—Ru2 2.105 (15), N4A"—Ru2 2.077 (13), Cl2—Ru2 2.392 (5), N4A"—Ru2—N1A' 76.6 (5), N4A"—Ru2—Cl2 82.7 (4), N1A'—Ru2—Cl2 84.6 (4).

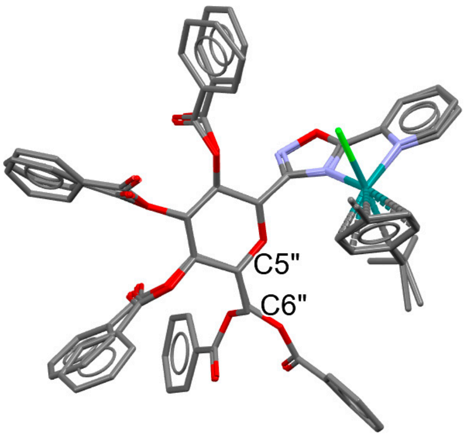

Figure S2. The two molecules of **Ru-1** in the asymmetric unit overlayed as stick modells showing the differences in conformation primarily by rotation of the substituent around the C5”-C6” sigma bond.

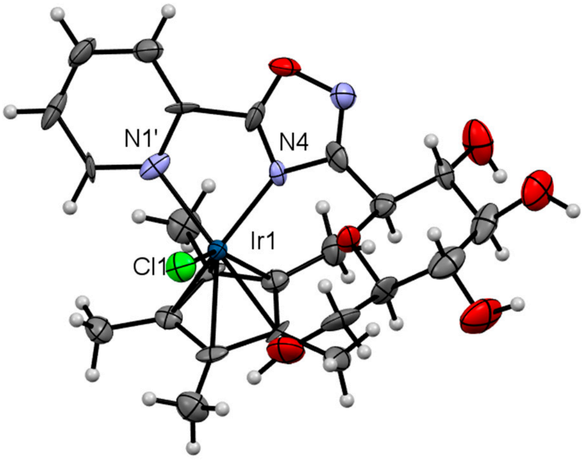

Figure S3. Ortep view of complex **Ir-2** at 50 % probability level. The counter ion is not shown for clarity. Key bond lengths [Å] and angles [°]: Cl1—Ir1 2.393 (4), N1'—Ir1 2.144 (11), N4—Ir1 2.161 (10), N1'—Ir1—Cl1 86.5 (4), N4—Ir1—Cl1 86.4 (3), N1'—Ir1—N4 76.3 (5)

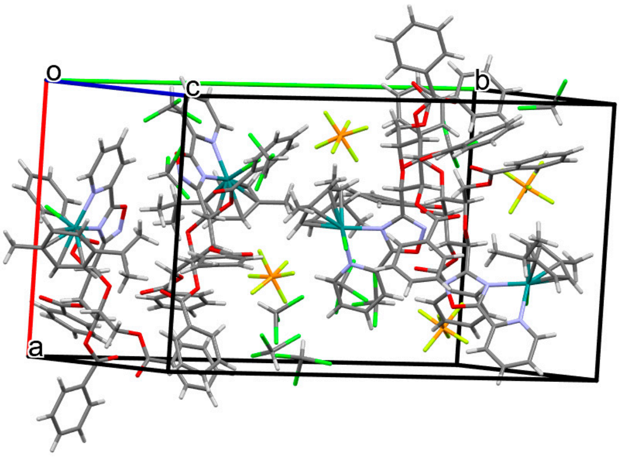

Figure S4. Packing diagram of **Ru-1**

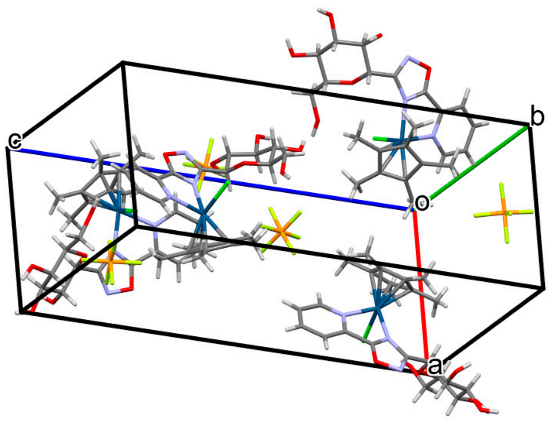

Figure S5. Packing diagram of **Ir-2**

Table S1. Geometric parameters (Å, º) for **Ru-1**

C1—O5	1.414 (18)	C61—C62	1.39 (2)
C1—C3"	1.49 (2)	C60A—O60A	1.225 (19)
C1—C2	1.52 (2)	C60A—O6A	1.367 (18)
C1—H1	1.0000	C60A—C61A	1.48 (2)
C2—O2	1.447 (18)	C62—C63	1.41 (3)
C2—C3	1.51 (2)	C62—H62	0.9500
C2—H2	1.0000	C61A—C66A	1.38 (2)
C1A—O5A	1.422 (16)	C61A—C62A	1.41 (2)
C1A—C3A"	1.49 (2)	C63—C64	1.46 (3)
C1A—C2A	1.51 (2)	C63—H63	0.9500
C1A—H1A	1.0000	C62A—C63A	1.30 (2)
C2'—C3'	1.37 (2)	C62A—H62A	0.9500
C2'—N1'	1.40 (2)	C64—C65	1.37 (2)
C2'—C5"	1.46 (2)	C64—H64	0.9500
C3—O3	1.477 (18)	C63A—C64A	1.39 (3)
C3—C4	1.50 (2)	C63A—H63A	0.9500
C3—H3	1.0000	C65—C66	1.36 (2)
C2A—O2A	1.489 (15)	C65—H65	0.9500
C2A—C3A	1.55 (2)	C64A—C65A	1.38 (2)
C2A—H2A	1.0000	C64A—H64A	0.9500
C3"—N2"	1.288 (18)	C66—H66	0.9500
C3"—N4"	1.404 (18)	C65A—C66A	1.38 (2)
C3'—C4'	1.37 (3)	C65A—H65A	0.9500
C3'—H3'	0.9500	C66A—H66A	0.9500
C4—O4	1.435 (18)	C71—C72	1.39 (2)
C4—C5	1.54 (2)	C71—C76	1.44 (2)
C4—H4	1.0000	C71—C78	1.51 (2)
C3A—O3A	1.415 (18)	C71—Ru1	2.216 (15)
C3A—C4A	1.499 (18)	C72—C73	1.39 (2)
C3A—H3A	1.0000	C72—Ru1	2.123 (17)
C4'—C5'	1.37 (3)	C72—H72	1.0000
C4'—H4'	0.9500	C71A—C72A	1.34 (3)
C5—O5	1.447 (17)	C71A—C76A	1.39 (3)
C5—C6	1.48 (2)	C71A—C78A	1.51 (3)
C5—H5	1.0000	C71A—Ru2	2.19 (2)
C4A—O4A	1.434 (15)	C73—C74	1.39 (3)
C4A—C5A	1.54 (2)	C73—Ru1	2.178 (16)
C4A—H4A	1.0000	C73—H73	1.0000
C5"—N4"	1.290 (19)	C72A—C73A	1.41 (3)

C5"—O1"	1.314 (18)	C72A—Ru2	2.18 (2)
C5'—C6'	1.43 (3)	C72A—H72A	1.0000
C5'—H5'	0.9500	C74—C75	1.41 (3)
C6—O6	1.467 (17)	C74—C77	1.45 (3)
C6—H6A	0.9900	C74—Ru1	2.19 (3)
C6—H6AB	0.9900	C73A—C74A	1.42 (4)
C5A—O5A	1.434 (17)	C73A—Ru2	2.186 (18)
C5A—C6A	1.518 (19)	C73A—H73A	1.0000
C5A—H5A	1.0000	C75—C76	1.42 (2)
C6'—N1'	1.31 (2)	C75—Ru1	2.176 (16)
C6'—H6'	0.9500	C75—H75	1.0000
C6A—O6A	1.448 (17)	C74A—C75A	1.41 (4)
C6A—H6AA	0.9900	C74A—C77A	1.56 (3)
C6A—H6AC	0.9900	C74A—Ru2	2.18 (3)
C20—O20	1.17 (2)	C76—Ru1	2.165 (16)
C20—O2	1.37 (2)	C76—H76	1.0000
C20—C21	1.47 (3)	C75A—C76A	1.42 (3)
C21—C22	1.35 (3)	C75A—Ru2	2.217 (18)
C21—C26	1.39 (3)	C75A—H75A	1.0000
C20A—O20A	1.163 (16)	C77—H77A	0.9800
C20A—O2A	1.377 (17)	C77—H77B	0.9800
C20A—C21A	1.46 (2)	C77—H77C	0.9800
C22—C23	1.40 (2)	C76A—Ru2	2.137 (16)
C22—H22	0.9500	C76A—H76A	1.0000
C21A—C26A	1.38 (2)	C78—C80	1.48 (2)
C21A—C22A	1.42 (2)	C78—C79	1.53 (2)
C23—C24	1.41 (4)	C78—H78	1.0000
C23—H23	0.9500	C77A—H77D	0.9800
C22A—C23A	1.40 (2)	C77A—H77E	0.9800
C22A—H22A	0.9500	C77A—H77F	0.9800
C24—C25	1.43 (4)	C79—H79A	0.9800
C24—H24	0.9500	C79—H79B	0.9800
C23A—C24A	1.32 (3)	C79—H79C	0.9800
C23A—H23A	0.9500	C78A—C79A	1.35 (3)
C25—C26	1.34 (3)	C78A—C80A	1.53 (3)
C25—H25	0.9500	C78A—H78A	1.0000
C24A—C25A	1.37 (3)	C80—H80A	0.9800
C24A—H24A	0.9500	C80—H80B	0.9800
C26—H26	0.9500	C80—H80C	0.9800
C25A—C26A	1.42 (3)	C79A—H79D	0.9800

C25A—H25A	0.9500	C79A—H79E	0.9800
C26A—H26A	0.9500	C79A—H79F	0.9800
C30—O30	1.15 (2)	C80A—H80D	0.9800
C30—O3	1.33 (3)	C80A—H80E	0.9800
C30—C31	1.41 (3)	C80A—H80F	0.9800
C31—C32	1.23 (3)	C92—Cl23	1.68 (3)
C31—C36	1.33 (3)	C92—Cl21	1.75 (3)
C30A—O30A	1.24 (2)	C92—Cl22	1.75 (2)
C30A—O3A	1.32 (2)	C92—H92	1.0000
C30A—C31A	1.42 (3)	C94—Cl42	1.66 (2)
C32—C33	1.47 (3)	C94—Cl43	1.81 (2)
C32—H32	0.9500	C94—Cl41	1.91 (3)
C31A—C32A	1.34 (3)	C94—H94	1.0000
C31A—C36A	1.35 (3)	C95—Cl53	1.73 (2)
C33—C34	1.34 (3)	C95—Cl51	1.75 (2)
C33—H33	0.9500	C95—Cl52	1.77 (2)
C32A—C33A	1.43 (5)	C95—H95	1.0000
C32A—H32A	0.9500	C2A'—C3A'	1.33 (2)
C34—C35	1.13 (3)	C2A'—N1A'	1.399 (19)
C34—H34	0.9500	C2A'—C5A"	1.46 (2)
C33A—C34A	1.28 (6)	C3A"—N2A"	1.313 (17)
C33A—H33A	0.9500	C3A"—N4A"	1.382 (19)
C35—C36	1.51 (3)	C3A'—C4A'	1.37 (2)
C35—H35	0.9500	C3A'—H3A'	0.9500
C34A—C35A	1.39 (5)	C4A'—C5A'	1.36 (2)
C34A—H34A	0.9500	C4A'—H4A'	0.9500
C36—H36	0.9500	C5A"—O1A"	1.305 (17)
C35A—C36A	1.46 (3)	C5A"—N4A"	1.339 (18)
C35A—H35A	0.9500	C5A'—C6A'	1.39 (2)
C36A—H36A	0.9500	C5A'—H5A'	0.9500
C40—O40	1.213 (19)	C6A'—N1A'	1.313 (19)
C40—O4	1.348 (18)	C6A'—H6A'	0.9500
C40—C41	1.43 (2)	N1'—Ru1	2.104 (14)
C41—C42	1.36 (2)	N2"—O1"	1.426 (16)
C41—C46	1.40 (2)	N4"—Ru1	2.110 (12)
C40A—O40A	1.152 (17)	N1A'—Ru2	2.113 (13)
C40A—O4A	1.336 (18)	N2A"—O1A"	1.436 (15)
C40A—C41A	1.52 (2)	N4A"—Ru2	2.079 (13)
C42—C43	1.34 (3)	F16—P1	1.570 (13)
C42—H42	0.9500	F11—P1	1.565 (16)

C41A—C46A	1.38 (2)	F12—P1	1.537 (14)
C41A—C42A	1.42 (2)	F13—P1	1.57 (2)
C43—C44	1.35 (3)	F14—P1	1.515 (17)
C43—H43	0.9500	F15—P1	1.601 (12)
C42A—C43A	1.36 (3)	F21—P2	1.573 (11)
C42A—H42A	0.9500	F22—P2	1.600 (16)
C44—C45	1.46 (3)	F23—P2	1.447 (15)
C44—H44	0.9500	F24—P2	1.521 (15)
C43A—C44A	1.36 (3)	F25—P2	1.532 (13)
C43A—H43A	0.9500	F26—P2	1.65 (2)
C45—C46	1.38 (3)	Cl1—Ru1	2.376 (5)
C45—H45	0.9500	Cl2—Ru2	2.388 (4)
C44A—C45A	1.40 (3)	C93—Cl33	1.62 (2)
C44A—H44A	0.9500	C93—Cl32	1.664 (19)
C46—H46	0.9500	C93—Cl31	1.79 (3)
C45A—C46A	1.42 (2)	C93—H93	1.0000
C45A—H45A	0.9500	C96—Cl62	1.674 (18)
C46A—H46A	0.9500	C96—Cl63	1.657 (18)
C60—O60	1.250 (16)	C96—Cl61	1.715 (19)
C60—O6	1.347 (17)	C96—H96	1.0000
C60—C61	1.45 (2)	Cl62—Cl63	2.52 (2)
C61—C66	1.38 (2)		
O5—C1—C3"	104.8 (13)	C72—C73—Ru1	69.1 (9)
O5—C1—C2	112.4 (12)	C74—C73—H73	119.0000
C3"—C1—C2	108.3 (13)	C72—C73—H73	119.0000
O5—C1—H1	110.4000	Ru1—C73—H73	119.0000
C3"—C1—H1	110.4000	C71A—C72A—C73A	125 (2)
C2—C1—H1	110.4000	C71A—C72A—Ru2	72.7 (14)
O2—C2—C3	112.2 (14)	C73A—C72A—Ru2	71.4 (12)
O2—C2—C1	103.2 (12)	C71A—C72A—H72A	116.1000
C3—C2—C1	109.3 (14)	C73A—C72A—H72A	116.1000
O2—C2—H2	110.6000	Ru2—C72A—H72A	116.1000
C3—C2—H2	110.6000	C73—C74—C75	119 (2)
C1—C2—H2	110.6000	C73—C74—C77	122 (3)
O5A—C1A—C3A"	106.4 (11)	C75—C74—C77	119 (2)
O5A—C1A—C2A	111.0 (12)	C73—C74—Ru1	71.0 (13)
C3A"—C1A—C2A	111.5 (13)	C75—C74—Ru1	70.6 (12)
O5A—C1A—H1A	109.3000	C77—C74—Ru1	131 (2)
C3A"—C1A—H1A	109.3000	C72A—C73A—C74A	116 (2)

C2A—C1A—H1A	109.3000	C72A—C73A—Ru2	71.0 (11)
C3'—C2'—N1'	124.0 (17)	C74A—C73A—Ru2	70.7 (14)
C3'—C2'—C5"	126.0 (18)	C72A—C73A—H73A	121.6000
N1'—C2'—C5"	110.0 (14)	C74A—C73A—H73A	121.6000
O3—C3—C4	105.2 (13)	Ru2—C73A—H73A	121.6000
O3—C3—C2	109.2 (13)	C74—C75—C76	118.7 (18)
C4—C3—C2	109.5 (14)	C74—C75—Ru1	71.8 (14)
O3—C3—H3	110.9000	C76—C75—Ru1	70.6 (9)
C4—C3—H3	110.9000	C74—C75—H75	120.2000
C2—C3—H3	110.9000	C76—C75—H75	120.2000
O2A—C2A—C1A	104.1 (11)	Ru1—C75—H75	120.2000
O2A—C2A—C3A	109.4 (11)	C73A—C74A—C75A	121 (2)
C1A—C2A—C3A	109.4 (12)	C73A—C74A—C77A	122 (3)
O2A—C2A—H2A	111.2000	C75A—C74A—C77A	116 (3)
C1A—C2A—H2A	111.2000	C73A—C74A—Ru2	71.3 (15)
C3A—C2A—H2A	111.2000	C75A—C74A—Ru2	72.8 (14)
N2"—C3"—N4"	110.9 (14)	C77A—C74A—Ru2	126.8 (18)
N2"—C3"—C1	126.1 (14)	C75—C76—C71	123.0 (15)
N4"—C3"—C1	122.5 (13)	C75—C76—Ru1	71.3 (9)
C2'—C3'—C4'	118 (2)	C71—C76—Ru1	72.8 (9)
C2'—C3'—H3'	121.2000	C75—C76—H76	117.8000
C4'—C3'—H3'	121.2000	C71—C76—H76	117.8000
O4—C4—C3	107.5 (14)	Ru1—C76—H76	117.8000
O4—C4—C5	108.8 (14)	C74A—C75A—C76A	116 (2)
C3—C4—C5	108.2 (14)	C74A—C75A—Ru2	69.8 (16)
O4—C4—H4	110.8000	C76A—C75A—Ru2	67.9 (10)
C3—C4—H4	110.8000	C74A—C75A—H75A	121.1000
C5—C4—H4	110.8000	C76A—C75A—H75A	121.1000
O3A—C3A—C4A	106.6 (12)	Ru2—C75A—H75A	121.1000
O3A—C3A—C2A	109.7 (11)	C74—C77—H77A	109.5000
C4A—C3A—C2A	108.4 (12)	C74—C77—H77B	109.5000
O3A—C3A—H3A	110.7000	H77A—C77—H77B	109.5000
C4A—C3A—H3A	110.7000	C74—C77—H77C	109.5000
C2A—C3A—H3A	110.7000	H77A—C77—H77C	109.5000
C5'—C4'—C3'	120 (2)	H77B—C77—H77C	109.5000
C5'—C4'—H4'	119.8000	C71A—C76A—C75A	124 (2)
C3'—C4'—H4'	119.8000	C71A—C76A—Ru2	73.5 (14)
O5—C5—C6	104.4 (13)	C75A—C76A—Ru2	74.0 (11)
O5—C5—C4	106.5 (12)	C71A—C76A—H76A	117.8000
C6—C5—C4	112.3 (13)	C75A—C76A—H76A	117.8000

O5—C5—H5	111.1000	Ru2—C76A—H76A	117.8000
C6—C5—H5	111.1000	C80—C78—C71	109.4 (15)
C4—C5—H5	111.1000	C80—C78—C79	110.0 (18)
O4A—C4A—C3A	109.7 (11)	C71—C78—C79	113.0 (15)
O4A—C4A—C5A	107.9 (11)	C80—C78—H78	108.1000
C3A—C4A—C5A	111.1 (12)	C71—C78—H78	108.1000
O4A—C4A—H4A	109.4000	C79—C78—H78	108.1000
C3A—C4A—H4A	109.4000	C74A—C77A—H77D	109.5000
C5A—C4A—H4A	109.4000	C74A—C77A—H77E	109.5000
N4"—C5"—O1"	114.3 (16)	H77D—C77A—H77E	109.5000
N4"—C5"—C2'	121.8 (16)	C74A—C77A—H77F	109.5000
O1"—C5"—C2'	123.5 (16)	H77D—C77A—H77F	109.5000
C4'—C5'—C6'	119 (2)	H77E—C77A—H77F	109.5000
C4'—C5'—H5'	120.5000	C78—C79—H79A	109.5000
C6'—C5'—H5'	120.5000	C78—C79—H79B	109.5000
O6—C6—C5	107.2 (12)	H79A—C79—H79B	109.5000
O6—C6—H6A	110.3000	C78—C79—H79C	109.5000
C5—C6—H6A	110.3000	H79A—C79—H79C	109.5000
O6—C6—H6AB	110.3000	H79B—C79—H79C	109.5000
C5—C6—H6AB	110.3000	C79A—C78A—C80A	126 (2)
H6A—C6—H6AB	108.5000	C79A—C78A—C71A	121 (2)
O5A—C5A—C6A	109.7 (12)	C80A—C78A—C71A	112 (2)
O5A—C5A—C4A	110.1 (11)	C79A—C78A—H78A	91.2000
C6A—C5A—C4A	113.3 (12)	C80A—C78A—H78A	91.2000
O5A—C5A—H5A	107.9000	C71A—C78A—H78A	91.2000
C6A—C5A—H5A	107.9000	C78—C80—H80A	109.5000
C4A—C5A—H5A	107.9000	C78—C80—H80B	109.5000
N1'—C6'—C5'	122 (2)	H80A—C80—H80B	109.5000
N1'—C6'—H6'	118.9000	C78—C80—H80C	109.5000
C5'—C6'—H6'	118.9000	H80A—C80—H80C	109.5000
O6A—C6A—C5A	109.2 (12)	H80B—C80—H80C	109.5000
O6A—C6A—H6AA	109.8000	C78A—C79A—H79D	109.5000
C5A—C6A—H6AA	109.8000	C78A—C79A—H79E	109.5000
O6A—C6A—H6AC	109.8000	H79D—C79A—H79E	109.5000
C5A—C6A—H6AC	109.8000	C78A—C79A—H79F	109.5000
H6AA—C6A—H6AC	108.3000	H79D—C79A—H79F	109.5000
O20—C20—O2	121 (2)	H79E—C79A—H79F	109.5000
O20—C20—C21	128 (2)	C78A—C80A—H80D	109.5000
O2—C20—C21	109.6 (18)	C78A—C80A—H80E	109.5000
C22—C21—C26	122 (2)	H80D—C80A—H80E	109.5000

C22—C21—C20	123.0 (19)	C78A—C80A—H80F	109.5000
C26—C21—C20	115 (2)	H80D—C80A—H80F	109.5000
O20A—C20A—O2A	124.8 (15)	H80E—C80A—H80F	109.5000
O20A—C20A—C21A	126.6 (15)	Cl23—C92—Cl21	109.9 (18)
O2A—C20A—C21A	108.6 (13)	Cl23—C92—Cl22	114.3 (14)
C21—C22—C23	122 (2)	Cl21—C92—Cl22	110.2 (13)
C21—C22—H22	118.8000	Cl23—C92—H92	107.4000
C23—C22—H22	118.8000	Cl21—C92—H92	107.4000
C26A—C21A—C22A	118.5 (16)	Cl22—C92—H92	107.4000
C26A—C21A—C20A	117.7 (15)	Cl42—C94—Cl43	111.7 (12)
C22A—C21A—C20A	123.7 (14)	Cl42—C94—Cl41	105.3 (12)
C22—C23—C24	115 (3)	Cl43—C94—Cl41	95.9 (12)
C22—C23—H23	122.6000	Cl42—C94—H94	114.1000
C24—C23—H23	122.6000	Cl43—C94—H94	114.1000
C23A—C22A—C21A	119.1 (16)	Cl41—C94—H94	114.1000
C23A—C22A—H22A	120.5000	Cl53—C95—Cl51	108.5 (13)
C21A—C22A—H22A	120.5000	Cl53—C95—Cl52	108.5 (13)
C23—C24—C25	121 (3)	Cl51—C95—Cl52	109.9 (11)
C23—C24—H24	119.3000	Cl53—C95—H95	110.0000
C25—C24—H24	119.3000	Cl51—C95—H95	110.0000
C24A—C23A—C22A	119 (2)	Cl52—C95—H95	110.0000
C24A—C23A—H23A	120.4000	C3A'—C2A'—N1A'	123.4 (15)
C22A—C23A—H23A	120.4000	C3A'—C2A'—C5A"	127.6 (16)
C26—C25—C24	120 (3)	N1A'—C2A'—C5A"	109.0 (14)
C26—C25—H25	119.9000	N2A"—C3A"—N4A"	114.2 (14)
C24—C25—H25	119.9000	N2A"—C3A"—C1A	122.7 (14)
C23A—C24A—C25A	126 (2)	N4A"—C3A"—C1A	123.0 (13)
C23A—C24A—H24A	116.8000	C2A'—C3A'—C4A'	118.3 (16)
C25A—C24A—H24A	116.8000	C2A'—C3A'—H3A'	120.9000
C25—C26—C21	118 (3)	C4A'—C3A'—H3A'	120.9000
C25—C26—H26	120.8000	C5A'—C4A'—C3A'	120.7 (17)
C21—C26—H26	120.8000	C5A'—C4A'—H4A'	119.6000
C24A—C25A—C26A	115 (2)	C3A'—C4A'—H4A'	119.6000
C24A—C25A—H25A	122.6000	O1A"—C5A"—N4A"	114.4 (14)
C26A—C25A—H25A	122.6000	O1A"—C5A"—C2A'	124.7 (14)
C21A—C26A—C25A	122.1 (19)	N4A"—C5A"—C2A'	120.8 (15)
C21A—C26A—H26A	119.0000	C4A'—C5A'—C6A'	117.9 (17)
C25A—C26A—H26A	119.0000	C4A'—C5A'—H5A'	121.0000
O30—C30—O3	118 (2)	C6A'—C5A'—H5A'	121.0000
O30—C30—C31	127 (3)	N1A'—C6A'—C5A'	123.5 (16)

O3—C30—C31	114 (2)	N1A'—C6A'—H6A'	118.3000
C32—C31—C36	115 (3)	C5A'—C6A'—H6A'	118.3000
C32—C31—C30	123 (3)	C6'—N1'—C2'	116.5 (16)
C36—C31—C30	121 (3)	C6'—N1'—Ru1	127.2 (14)
O30A—C30A—O3A	121 (2)	C2'—N1'—Ru1	116.3 (10)
O30A—C30A—C31A	121 (2)	C3"—N2"—O1"	106.1 (12)
O3A—C30A—C31A	118 (2)	C5"—N4"—C3"	103.8 (13)
C31—C32—C33	123 (3)	C5"—N4"—Ru1	114.1 (11)
C31—C32—H32	118.6000	C3"—N4"—Ru1	142.1 (10)
C33—C32—H32	118.6000	C6A'—N1A'—C2A'	116.0 (15)
C32A—C31A—C36A	116 (3)	C6A'—N1A'—Ru2	125.7 (12)
C32A—C31A—C30A	125 (3)	C2A'—N1A'—Ru2	118.1 (10)
C36A—C31A—C30A	118 (2)	C3A"—N2A"—O1A"	103.6 (12)
C34—C33—C32	117 (3)	C5A"—N4A"—C3A"	101.5 (13)
C34—C33—H33	121.7000	C5A"—N4A"—Ru2	115.0 (11)
C32—C33—H33	121.7000	C3A"—N4A"—Ru2	142.9 (10)
C31A—C32A—C33A	119 (4)	C5"—O1"—N2"	104.8 (12)
C31A—C32A—H32A	120.3000	C20—O2—C2	118.0 (14)
C33A—C32A—H32A	120.3000	C30—O3—C3	120.3 (14)
C35—C34—C33	120 (3)	C20A—O2A—C2A	115.6 (11)
C35—C34—H34	120.0000	C40—O4—C4	119.2 (14)
C33—C34—H34	120.0000	C30A—O3A—C3A	121.4 (15)
C34A—C33A—C32A	120 (4)	C1—O5—C5	111.4 (12)
C34A—C33A—H33A	120.1000	C40A—O4A—C4A	116.6 (12)
C32A—C33A—H33A	120.1000	C60—O6—C6	115.6 (12)
C34—C35—C36	121 (3)	C1A—O5A—C5A	111.1 (10)
C34—C35—H35	119.6000	C60A—O6A—C6A	114.6 (12)
C36—C35—H35	119.6000	C5A"—O1A"—N2A"	106.1 (11)
C33A—C34A—C35A	129 (4)	F14—P1—F12	92.9 (10)
C33A—C34A—H34A	115.7000	F14—P1—F13	90.9 (14)
C35A—C34A—H34A	115.7000	F12—P1—F13	91.5 (12)
C31—C36—C35	121 (2)	F14—P1—F11	91.2 (14)
C31—C36—H36	119.6000	F12—P1—F11	94.5 (12)
C35—C36—H36	119.6000	F13—P1—F11	173.6 (12)
C34A—C35A—C36A	107 (3)	F14—P1—F16	176.1 (13)
C34A—C35A—H35A	126.7000	F12—P1—F16	90.3 (7)
C36A—C35A—H35A	126.7000	F13—P1—F16	86.8 (11)
C31A—C36A—C35A	129 (3)	F11—P1—F16	90.8 (8)
C31A—C36A—H36A	115.4000	F14—P1—F15	87.4 (10)
C35A—C36A—H36A	115.4000	F12—P1—F15	179.1 (13)

O40—C40—O4	120.1 (17)	F13—P1—F15	87.7 (10)
O40—C40—C41	124.7 (17)	F11—P1—F15	86.3 (7)
O4—C40—C41	115.1 (16)	F16—P1—F15	89.3 (8)
C42—C41—C46	120.7 (17)	F23—P2—F24	88.9 (13)
C42—C41—C40	118.9 (18)	F23—P2—F25	104.2 (15)
C46—C41—C40	120.3 (17)	F24—P2—F25	90.5 (8)
O40A—C40A—O4A	125.7 (15)	F23—P2—F21	89.9 (8)
O40A—C40A—C41A	125.3 (16)	F24—P2—F21	177.9 (9)
O4A—C40A—C41A	108.9 (15)	F25—P2—F21	91.5 (6)
C43—C42—C41	123 (2)	F23—P2—F22	93.5 (14)
C43—C42—H42	118.4000	F24—P2—F22	88.2 (9)
C41—C42—H42	118.4000	F25—P2—F22	162.2 (11)
C46A—C41A—C42A	119.3 (18)	F21—P2—F22	90.2 (8)
C46A—C41A—C40A	123.7 (17)	F23—P2—F26	168.9 (14)
C42A—C41A—C40A	116.8 (17)	F24—P2—F26	97.4 (13)
C42—C43—C44	118 (3)	F25—P2—F26	85.0 (10)
C42—C43—H43	120.8000	F21—P2—F26	83.5 (9)
C44—C43—H43	120.8000	F22—P2—F26	77.6 (10)
C43A—C42A—C41A	120 (2)	Cl33—C93—Cl32	120.0 (13)
C43A—C42A—H42A	120.1000	Cl33—C93—Cl31	103.0 (18)
C41A—C42A—H42A	120.1000	Cl32—C93—Cl31	110.7 (17)
C43—C44—C45	121 (2)	Cl33—C93—H93	107.5000
C43—C44—H44	119.7000	Cl32—C93—H93	107.5000
C45—C44—H44	119.7000	Cl31—C93—H93	107.5000
C42A—C43A—C44A	122 (3)	Cl62—C96—Cl63	98.2 (15)
C42A—C43A—H43A	118.9000	Cl62—C96—Cl61	107.7 (13)
C44A—C43A—H43A	118.9000	Cl63—C96—Cl61	113.6 (13)
C46—C45—C44	119 (2)	Cl62—C96—H96	112.2000
C46—C45—H45	120.6000	Cl63—C96—H96	112.2000
C44—C45—H45	120.6000	Cl61—C96—H96	112.2000
C43A—C44A—C45A	119 (2)	C96—Cl62—Cl63	40.6 (9)
C43A—C44A—H44A	120.4000	C96—Cl63—Cl62	41.1 (8)
C45A—C44A—H44A	120.4000	N1'—Ru1—N4"	77.3 (5)
C45—C46—C41	118 (2)	N1'—Ru1—C72	91.8 (6)
C45—C46—H46	121.1000	N4"—Ru1—C72	135.8 (6)
C41—C46—H46	121.1000	N1'—Ru1—C76	138.9 (6)
C44A—C45A—C46A	120 (2)	N4"—Ru1—C76	93.4 (5)
C44A—C45A—H45A	120.0000	C72—Ru1—C76	67.0 (6)
C46A—C45A—H45A	120.0000	N1'—Ru1—C73	105.9 (8)
C41A—C46A—C45A	119 (2)	N4"—Ru1—C73	171.7 (6)

C41A—C46A—H46A	120.3000	C72—Ru1—C73	37.6 (7)
C45A—C46A—H46A	120.3000	C76—Ru1—C73	79.1 (6)
O60—C60—O6	120.2 (16)	N1'—Ru1—C75	172.2 (6)
O60—C60—C61	125.5 (15)	N4"—Ru1—C75	108.9 (6)
O6—C60—C61	114.2 (13)	C72—Ru1—C75	80.4 (6)
C66—C61—C62	121.6 (17)	C76—Ru1—C75	38.1 (6)
C66—C61—C60	124.0 (15)	C73—Ru1—C75	67.3 (8)
C62—C61—C60	114.3 (15)	N1'—Ru1—C74	137.9 (9)
O60A—C60A—O6A	121.3 (17)	N4"—Ru1—C74	142.7 (8)
O60A—C60A—C61A	125.7 (16)	C72—Ru1—C74	67.8 (8)
O6A—C60A—C61A	112.8 (15)	C76—Ru1—C74	67.8 (9)
C61—C62—C63	119.0 (19)	C73—Ru1—C74	37.1 (8)
C61—C62—H62	120.5000	C75—Ru1—C74	37.6 (8)
C63—C62—H62	120.5000	N1'—Ru1—C71	104.7 (5)
C66A—C61A—C62A	119.3 (17)	N4"—Ru1—C71	104.0 (5)
C66A—C61A—C60A	122.2 (15)	C72—Ru1—C71	37.2 (5)
C62A—C61A—C60A	118.4 (17)	C76—Ru1—C71	38.2 (5)
C62—C63—C64	119 (2)	C73—Ru1—C71	67.9 (6)
C62—C63—H63	120.7000	C75—Ru1—C71	69.6 (6)
C64—C63—H63	120.7000	C74—Ru1—C71	81.5 (9)
C63A—C62A—C61A	119.3 (19)	N1'—Ru1—Cl1	83.6 (4)
C63A—C62A—H62A	120.3000	N4"—Ru1—Cl1	84.2 (3)
C61A—C62A—H62A	120.3000	C72—Ru1—Cl1	137.8 (5)
C65—C64—C63	118 (2)	C76—Ru1—Cl1	135.8 (5)
C65—C64—H64	120.9000	C73—Ru1—Cl1	103.7 (5)
C63—C64—H64	120.9000	C75—Ru1—Cl1	101.4 (5)
C62A—C63A—C64A	121.7 (19)	C74—Ru1—Cl1	87.9 (8)
C62A—C63A—H63A	119.2000	C71—Ru1—Cl1	169.4 (4)
C64A—C63A—H63A	119.2000	N4A"—Ru2—N1A'	76.9 (5)
C66—C65—C64	122.8 (18)	N4A"—Ru2—C76A	93.8 (7)
C66—C65—H65	118.6000	N1A'—Ru2—C76A	136.0 (8)
C64—C65—H65	118.6000	N4A"—Ru2—C74A	141.5 (12)
C65A—C64A—C63A	121.1 (19)	N1A'—Ru2—C74A	139.7 (12)
C65A—C64A—H64A	119.5000	C76A—Ru2—C74A	67.8 (10)
C63A—C64A—H64A	119.5000	N4A"—Ru2—C72A	137.0 (8)
C65—C66—C61	119.7 (16)	N1A'—Ru2—C72A	92.6 (7)
C65—C66—H66	120.2000	C76A—Ru2—C72A	65.2 (8)
C61—C66—H66	120.2000	C74A—Ru2—C72A	66.9 (10)
C64A—C65A—C66A	117.4 (19)	N4A"—Ru2—C73A	173.4 (6)
C64A—C65A—H65A	121.3000	N1A'—Ru2—C73A	105.6 (8)

C66A—C65A—H65A	121.3000	C76A—Ru2—C73A	80.1 (8)
C65A—C66A—C61A	120.9 (17)	C74A—Ru2—C73A	38.0 (10)
C65A—C66A—H66A	119.6000	C72A—Ru2—C73A	37.6 (8)
C61A—C66A—H66A	119.6000	N4A"—Ru2—C71A	105.7 (8)
C72—C71—C76	114.2 (15)	N1A'—Ru2—C71A	103.3 (9)
C72—C71—C78	121.5 (15)	C76A—Ru2—C71A	37.5 (9)
C76—C71—C78	124.2 (14)	C74A—Ru2—C71A	80.4 (10)
C72—C71—Ru1	67.8 (10)	C72A—Ru2—C71A	35.7 (9)
C76—C71—Ru1	69.0 (9)	C73A—Ru2—C71A	67.8 (9)
C78—C71—Ru1	134.9 (10)	N4A"—Ru2—C75A	108.5 (9)
C71—C72—C73	124.5 (18)	N1A'—Ru2—C75A	170.9 (6)
C71—C72—Ru1	75.0 (9)	C76A—Ru2—C75A	38.1 (8)
C73—C72—Ru1	73.3 (11)	C74A—Ru2—C75A	37.4 (10)
C71—C72—H72	117.4000	C72A—Ru2—C75A	78.5 (8)
C73—C72—H72	117.4000	C73A—Ru2—C75A	68.2 (10)
Ru1—C72—H72	117.4000	C71A—Ru2—C75A	68.5 (10)
C72A—C71A—C76A	117 (2)	N4A"—Ru2—Cl2	82.7 (4)
C72A—C71A—C78A	122 (3)	N1A'—Ru2—Cl2	84.7 (4)
C76A—C71A—C78A	122 (3)	C76A—Ru2—Cl2	137.4 (6)
C72A—C71A—Ru2	71.6 (15)	C74A—Ru2—Cl2	88.9 (7)
C76A—C71A—Ru2	69.1 (12)	C72A—Ru2—Cl2	138.4 (7)
C78A—C71A—Ru2	131.8 (18)	C73A—Ru2—Cl2	103.5 (6)
C74—C73—C72	120 (2)	C71A—Ru2—Cl2	169.3 (7)
C74—C73—Ru1	72.0 (14)	C75A—Ru2—Cl2	103.0 (7)
O5—C1—C2—O2	173.3 (13)	C71—C72—C73—C74	-5 (3)
C3"—C1—C2—O2	-71.4 (15)	Ru1—C72—C73—C74	53 (2)
O5—C1—C2—C3	53.7 (17)	C71—C72—C73—Ru1	-57.8 (15)
C3"—C1—C2—C3	169.0 (12)	C76A—C71A—C72A—C73A	-3 (4)
O2—C2—C3—O3	77.6 (15)	C78A—C71A—C72A—C73A	179 (2)
C1—C2—C3—O3	-168.6 (12)	Ru2—C71A—C72A—C73A	51 (2)
O2—C2—C3—C4	-167.8 (13)	C76A—C71A—C72A—Ru2	-54.0 (19)
C1—C2—C3—C4	-53.9 (17)	C78A—C71A—C72A—Ru2	128 (3)
O5A—C1A—C2A—O2A	176.4 (11)	C72—C73—C74—C75	2 (4)
C3A"—C1A—C2A—O2A	-65.1 (14)	Ru1—C73—C74—C75	53 (2)
O5A—C1A—C2A—C3A	59.5 (14)	C72—C73—C74—C77	-179 (2)
C3A"—C1A—C2A—C3A	178.0 (11)	Ru1—C73—C74—C77	-127 (3)
O5—C1—C3"—N2"	81 (2)	C72—C73—C74—Ru1	-51.3 (18)
C2—C1—C3"—N2"	-39 (2)	C71A—C72A—C73A—C74A	5 (3)
O5—C1—C3"—N4"	-90.1 (16)	Ru2—C72A—C73A—C74A	55.9 (19)

C2—C1—C3"—N4"	149.7 (14)	C71A—C72A—C73A—Ru2	-51 (2)
N1'—C2'—C3'—C4'	-4 (3)	C73—C74—C75—C76	1 (3)
C5"—C2'—C3'—C4'	175.6 (18)	C77—C74—C75—C76	-178 (2)
O3—C3—C4—O4	-65.2 (16)	Ru1—C74—C75—C76	54.6 (15)
C2—C3—C4—O4	177.7 (12)	C73—C74—C75—Ru1	-54 (2)
O3—C3—C4—C5	177.6 (12)	C77—C74—C75—Ru1	127 (3)
C2—C3—C4—C5	60.4 (17)	C72A—C73A—C74A—C75A	-1 (4)
O2A—C2A—C3A—O3A	75.7 (14)	Ru2—C73A—C74A—C75A	55 (2)
C1A—C2A—C3A—O3A	-170.8 (11)	C72A—C73A—C74A—C77A	-178 (2)
O2A—C2A—C3A—C4A	-168.2 (11)	Ru2—C73A—C74A—C77A	-122 (3)
C1A—C2A—C3A—C4A	-54.7 (15)	C72A—C73A—C74A—Ru2	-56.0 (17)
C2'—C3'—C4'—C5'	5 (3)	C74—C75—C76—C71	-1 (3)
O4—C4—C5—O5	179.3 (12)	Ru1—C75—C76—C71	53.7 (13)
C3—C4—C5—O5	-64.2 (17)	C74—C75—C76—Ru1	-55.2 (18)
O4—C4—C5—C6	65.6 (17)	C72—C71—C76—C75	-1 (2)
C3—C4—C5—C6	-177.9 (14)	C78—C71—C76—C75	176.2 (14)
O3A—C3A—C4A—O4A	-68.9 (14)	Ru1—C71—C76—C75	-53.1 (13)
C2A—C3A—C4A—O4A	173.1 (11)	C72—C71—C76—Ru1	52.0 (12)
O3A—C3A—C4A—C5A	171.8 (11)	C78—C71—C76—Ru1	-130.8 (14)
C2A—C3A—C4A—C5A	53.8 (15)	C73A—C74A—C75A—C76A	-3 (4)
C3'—C2'—C5"—N4"	-178.5 (17)	C77A—C74A—C75A—C76A	174.2 (18)
N1'—C2'—C5"—N4"	1 (2)	Ru2—C74A—C75A—C76A	51.1 (16)
C3'—C2'—C5"—O1"	-7 (3)	C73A—C74A—C75A—Ru2	-54 (2)
N1'—C2'—C5"—O1"	172.8 (15)	C77A—C74A—C75A—Ru2	123 (2)
C3'—C4'—C5'—C6'	-5 (3)	C72A—C71A—C76A—C75A	-2 (3)
O5—C5—C6—O6	88.3 (14)	C78A—C71A—C76A—C75A	175.9 (19)
C4—C5—C6—O6	-156.8 (13)	Ru2—C71A—C76A—C75A	-57.1 (16)
O4A—C4A—C5A—O5A	-177.5 (10)	C72A—C71A—C76A—Ru2	55 (2)
C3A—C4A—C5A—O5A	-57.1 (15)	C78A—C71A—C76A—Ru2	-127 (2)
O4A—C4A—C5A—C6A	59.3 (15)	C74A—C75A—C76A—C71A	5 (3)
C3A—C4A—C5A—C6A	179.7 (12)	Ru2—C75A—C76A—C71A	56.9 (18)
C4'—C5'—C6'—N1'	4 (3)	C74A—C75A—C76A—Ru2	-52.0 (18)
O5A—C5A—C6A—O6A	-74.3 (15)	C72—C71—C78—C80	89 (2)
C4A—C5A—C6A—O6A	49.1 (17)	C76—C71—C78—C80	-88.4 (19)
O20—C20—C21—C22	-167 (2)	Ru1—C71—C78—C80	177.9 (13)
O2—C20—C21—C22	6 (3)	C72—C71—C78—C79	-148.3 (17)
O20—C20—C21—C26	14 (3)	C76—C71—C78—C79	35 (2)
O2—C20—C21—C26	-173.9 (16)	Ru1—C71—C78—C79	-59 (2)
C26—C21—C22—C23	-2 (3)	C72A—C71A—C78A—C79A	-77 (4)
C20—C21—C22—C23	178.3 (17)	C76A—C71A—C78A—C79A	105 (4)

O20A—C20A—C21A—C26A	6 (3)	Ru2—C71A—C78A—C79A	16 (5)
O2A—C20A—C21A—C26A	-175.2 (15)	C72A—C71A—C78A—C80A	99 (4)
O20A—C20A—C21A—C22A	-174.0 (17)	C76A—C71A—C78A—C80A	-78 (3)
O2A—C20A—C21A—C22A	5 (2)	Ru2—C71A—C78A—C80A	-168 (3)
C21—C22—C23—C24	6 (3)	O5A—C1A—C3A"—N2A"	95.2 (17)
C26A—C21A—C22A—C23A	2 (2)	C2A—C1A—C3A"—N2A"	-25.9 (19)
C20A—C21A—C22A—C23A	-177.9 (15)	O5A—C1A—C3A"—N4A"	-87.9 (16)
C22—C23—C24—C25	-8 (3)	C2A—C1A—C3A"—N4A"	151.0 (13)
C21A—C22A—C23A—C24A	-1 (3)	N1A'—C2A'—C3A'—C4A'	0 (3)
C23—C24—C25—C26	7 (4)	C5A"—C2A'—C3A'—C4A'	179.4 (16)
C22A—C23A—C24A—C25A	1 (3)	C2A'—C3A'—C4A'—C5A'	2 (3)
C24—C25—C26—C21	-2 (3)	C3A'—C2A'—C5A"—O1A"	-4 (3)
C22—C21—C26—C25	0 (3)	N1A'—C2A'—C5A"—O1A"	175.8 (14)
C20—C21—C26—C25	180 (2)	C3A'—C2A'—C5A"—N4A"	-179.5 (16)
C23A—C24A—C25A—C26A	-2 (3)	N1A'—C2A'—C5A"—N4A"	0 (2)
C22A—C21A—C26A—C25A	-4 (3)	C3A'—C4A'—C5A'—C6A'	-4 (3)
C20A—C21A—C26A—C25A	176.3 (18)	C4A'—C5A'—C6A'—N1A'	4 (3)
C24A—C25A—C26A—C21A	4 (3)	C5'—C6'—N1'—C2'	-2 (3)
O30—C30—C31—C32	-177 (4)	C5'—C6'—N1'—Ru1	176.1 (14)
O3—C30—C31—C32	14 (5)	C3'—C2'—N1'—C6'	3 (3)
O30—C30—C31—C36	-4 (6)	C5"—C2'—N1'—C6'	-176.9 (15)
O3—C30—C31—C36	-173 (3)	C3'—C2'—N1'—Ru1	-176.0 (15)
C36—C31—C32—C33	-11 (5)	C5"—C2'—N1'—Ru1	4.4 (18)
C30—C31—C32—C33	163 (3)	N4"—C3"—N2"—O1"	-3.7 (18)
O30A—C30A—C31A—C32A	13 (4)	C1—C3"—N2"—O1"	-175.5 (15)
O3A—C30A—C31A—C32A	-168 (2)	O1"—C5"—N4"—C3"	0.0 (18)
O30A—C30A—C31A—C36A	-171 (2)	C2'—C5"—N4"—C3"	172.5 (15)
O3A—C30A—C31A—C36A	9 (3)	O1"—C5"—N4"—Ru1	-178.3 (10)
C31—C32—C33—C34	20 (5)	C2'—C5"—N4"—Ru1	-6 (2)
C36A—C31A—C32A—C33A	0 (4)	N2"—C3"—N4"—C5"	2.5 (18)
C30A—C31A—C32A—C33A	177 (3)	C1—C3"—N4"—C5"	174.6 (15)
C32—C33—C34—C35	-22 (5)	N2"—C3"—N4"—Ru1	179.9 (12)
C31A—C32A—C33A—C34A	5 (7)	C1—C3"—N4"—Ru1	-8 (3)
C33—C34—C35—C36	16 (5)	C5A'—C6A'—N1A'—C2A'	-2 (2)
C32A—C33A—C34A—C35A	-7 (9)	C5A'—C6A'—N1A'—Ru2	-178.5 (13)
C32—C31—C36—C35	4 (5)	C3A'—C2A'—N1A'—C6A'	0 (2)
C30—C31—C36—C35	-170 (3)	C5A"—C2A'—N1A'—C6A'	-179.6 (14)
C34—C35—C36—C31	-6 (5)	C3A'—C2A'—N1A'—Ru2	176.9 (13)
C33A—C34A—C35A—C36A	3 (7)	C5A"—C2A'—N1A'—Ru2	-2.6 (17)
C32A—C31A—C36A—C35A	-5 (4)	N4A"—C3A"—N2A"—O1A"	1.0 (17)

C30A—C31A—C36A—C35A	178 (2)	C1A—C3A"—N2A"—O1A"	178.2 (12)
C34A—C35A—C36A—C31A	4 (4)	O1A"—C5A"—N4A"—C3A"	-0.2 (17)
O40—C40—C41—C42	-5 (3)	C2A'—C5A"—N4A"—C3A"	176.0 (14)
O4—C40—C41—C42	175.1 (16)	O1A"—C5A"—N4A"—Ru2	-173.6 (10)
O40—C40—C41—C46	178.8 (18)	C2A'—C5A"—N4A"—Ru2	2.6 (19)
O4—C40—C41—C46	-1 (2)	N2A"—C3A"—N4A"—C5A"	-0.6 (17)
C46—C41—C42—C43	-7 (3)	C1A—C3A"—N4A"—C5A"	-177.7 (14)
C40—C41—C42—C43	177 (2)	N2A"—C3A"—N4A"—Ru2	169.5 (12)
O40A—C40A—C41A—C46A	166.8 (18)	C1A—C3A"—N4A"—Ru2	-8 (3)
O4A—C40A—C41A—C46A	-17 (2)	N4"—C5"—O1"—N2"	-2.1 (18)
O40A—C40A—C41A—C42A	-9 (3)	C2'—C5"—O1"—N2"	-174.5 (15)
O4A—C40A—C41A—C42A	167.8 (15)	C3"—N2"—O1"—C5"	3.5 (17)
C41—C42—C43—C44	8 (4)	O20—C20—O2—C2	4 (3)
C46A—C41A—C42A—C43A	0 (3)	C21—C20—O2—C2	-168.9 (15)
C40A—C41A—C42A—C43A	175.6 (19)	C3—C2—O2—C20	-97.1 (18)
C42—C43—C44—C45	-7 (4)	C1—C2—O2—C20	145.4 (16)
C41A—C42A—C43A—C44A	1 (4)	O30—C30—O3—C3	7 (4)
C43—C44—C45—C46	6 (3)	C31—C30—O3—C3	177 (2)
C42A—C43A—C44A—C45A	0 (4)	C4—C3—O3—C30	132 (2)
C44—C45—C46—C41	-4 (3)	C2—C3—O3—C30	-111 (2)
C42—C41—C46—C45	5 (3)	O20A—C20A—O2A—C2A	14 (2)
C40—C41—C46—C45	-178.8 (17)	C21A—C20A—O2A—C2A	-165.4 (12)
C43A—C44A—C45A—C46A	-2 (3)	C1A—C2A—O2A—C20A	128.9 (13)
C42A—C41A—C46A—C45A	-2 (3)	C3A—C2A—O2A—C20A	-114.2 (14)
C40A—C41A—C46A—C45A	-177.3 (16)	O40—C40—O4—C4	5 (3)
C44A—C45A—C46A—C41A	3 (3)	C41—C40—O4—C4	-174.4 (15)
O60—C60—C61—C66	-159.4 (16)	C3—C4—O4—C40	114.9 (16)
O6—C60—C61—C66	17 (2)	C5—C4—O4—C40	-128.2 (16)
O60—C60—C61—C62	19 (2)	O30A—C30A—O3A—C3A	5 (3)
O6—C60—C61—C62	-164.6 (15)	C31A—C30A—O3A—C3A	-174.7 (15)
C66—C61—C62—C63	3 (3)	C4A—C3A—O3A—C30A	139.2 (14)
C60—C61—C62—C63	-175.6 (18)	C2A—C3A—O3A—C30A	-103.6 (15)
O60A—C60A—C61A—C66A	170.3 (19)	C3"—C1—O5—C5	-178.0 (11)
O6A—C60A—C61A—C66A	-15 (2)	C2—C1—O5—C5	-60.6 (16)
O60A—C60A—C61A—C62A	-10 (3)	C6—C5—O5—C1	-176.5 (12)
O6A—C60A—C61A—C62A	164.4 (15)	C4—C5—O5—C1	64.5 (15)
C61—C62—C63—C64	1 (3)	O40A—C40A—O4A—C4A	-2 (2)
C66A—C61A—C62A—C63A	-3 (3)	C41A—C40A—O4A—C4A	-179.1 (12)
C60A—C61A—C62A—C63A	177.3 (18)	C3A—C4A—O4A—C40A	111.4 (14)
C62—C63—C64—C65	-5 (3)	C5A—C4A—O4A—C40A	-127.4 (13)

C61A—C62A—C63A—C64A	7 (3)	O60—C60—O6—C6	1 (2)
C63—C64—C65—C66	5 (3)	C61—C60—O6—C6	-175.8 (12)
C62A—C63A—C64A—C65A	-6 (4)	C5—C6—O6—C60	-173.1 (12)
C64—C65—C66—C61	-1 (3)	C3A"—C1A—O5A—C5A	175.4 (11)
C62—C61—C66—C65	-3 (3)	C2A—C1A—O5A—C5A	-63.1 (15)
C60—C61—C66—C65	175.2 (15)	C6A—C5A—O5A—C1A	-174.1 (12)
C63A—C64A—C65A—C66A	1 (3)	C4A—C5A—O5A—C1A	60.6 (14)
C64A—C65A—C66A—C61A	2 (3)	O60A—C60A—O6A—C6A	-6 (2)
C62A—C61A—C66A—C65A	-1 (3)	C61A—C60A—O6A—C6A	179.1 (13)
C60A—C61A—C66A—C65A	178.4 (17)	C5A—C6A—O6A—C60A	-141.3 (14)
C76—C71—C72—C73	4 (2)	N4A"—C5A"—O1A"—N2A"	0.8 (17)
C78—C71—C72—C73	-172.9 (15)	C2A'—C5A"—O1A"—N2A"	-175.3 (14)
Ru1—C71—C72—C73	57.0 (15)	C3A"—N2A"—O1A"—C5A"	-1.0 (15)
C76—C71—C72—Ru1	-52.6 (11)	Cl61—C96—Cl62—Cl63	-118.0 (18)
C78—C71—C72—Ru1	130.1 (14)	Cl61—C96—Cl63—Cl62	113.5 (19)

Table S2. Hydrogen-bond geometry (Å, °) for **Ru-1**

D—H···*A*	*D*—H	H···*A*	*D*···*A*	*D*—H···*A*
C1—H1···F21	1.00	2.53	3.370 (19)	142
C1*A*—H1*A*···F15	1.00	2.61	3.485 (18)	146
C3—H3···F21	1.00	2.58	3.414 (18)	140
C2*A*—H2*A*···O60^{i}	1.00	2.24	3.082 (18)	141
C3′—H3′···O40*A*^{ii}	0.95	2.37	3.21 (2)	146
C3*A*—H3*A*···F15	1.00	2.51	3.367 (19)	144
C5—H5···F21	1.00	2.63	3.49 (2)	143
C5—H5···F26	1.00	2.64	3.62 (3)	165
C4*A*—H4*A*···O60^{i}	1.00	2.33	3.168 (18)	140
C6—H6*AB*···N2*A*″^{iii}	0.99	2.60	3.57 (2)	169
C5*A*—H5*A*···F14	1.00	2.60	3.52 (2)	152
C92—H92···F16	1.00	2.45	3.37 (3)	152
C92—H92···F13	1.00	2.48	3.41 (3)	153
C94—H94···F22	1.00	2.26	3.16 (2)	149
C94—H94···F24	1.00	2.55	3.34 (3)	136
C95—H95···F24^{iv}	1.00	2.34	3.08 (3)	130
C5*A*′—H5*A*′···Cl63	0.95	3.01	3.56 (2)	118
C6*A*′—H6*A*′···F23	0.95	2.52	3.08 (2)	118

Symmetry codes: (i) $-x+1, y+1/2, -z+1$; (ii) $-x, y-1/2, -z+1$; (iii) $-x+1, y-1/2, -z+1$; (iv) $x-1, y, z$.

Table S3. Geometric parameters (Å, º) for **Ir-2**

C1"—O5"	1.423 (19)	C52—C57	1.53 (2)
C1"—C3	1.46 (2)	C52—Ir1	2.147 (13)
C1"—C2"	1.54 (2)	C53—C54	1.45 (2)
C1"—H1"	0.98	C53—C58	1.50 (2)
C2"—O2"	1.425 (19)	C53—Ir1	2.157 (15)
C2"—C3"	1.48 (2)	C54—C55	1.43 (2)
C2"—H2"	0.98	C54—C59	1.48 (2)
C2'—N1'	1.354 (19)	C54—Ir1	2.182 (14)
C2'—C3'	1.37 (2)	C55—C60	1.48 (2)
C2'—C5	1.57 (2)	C55—Ir1	2.183 (14)
C3—N2	1.27 (2)	C56—H56A	0.96
C3—N4	1.405 (19)	C56—H56B	0.96
C3—C5	2.00 (2)	C56—H56C	0.96
C3"—O3"	1.42 (2)	C57—H57A	0.96
C3"—C4"	1.50 (3)	C57—H57B	0.96
C3"—H3"	0.98	C57—H57C	0.96
C3'—C4'	1.38 (2)	C58—H58A	0.96
C3'—H3'	0.93	C58—H58B	0.96
C4"—O4"	1.39 (2)	C58—H58C	0.96
C4"—C5"	1.53 (3)	C59—H59A	0.96
C4"—H4"	0.98	C59—H59B	0.96
C4'—C5'	1.35 (3)	C59—H59C	0.96
C4'—H4'	0.93	C60—H60A	0.96
C5—N4	1.257 (19)	C60—H60B	0.96
C5—O1	1.26 (2)	C60—H60C	0.96
C5"—O5"	1.435 (18)	N1'—Ir1	2.144 (11)
C5"—C6"	1.49 (3)	N2—O1	1.423 (18)
C5"—H5"	0.98	N4—Ir1	2.161 (10)
C5'—C6'	1.39 (2)	O2"—H2O	0.860 (14)
C5'—H5'	0.93	O3"—H3O	0.861 (14)
C6"—O6"	1.44 (2)	O4"—H4O	0.858 (14)
C6"—H6"A	0.97	O6"—H6O	0.859 (14)
C6"—H6"B	0.97	F11—P1	1.48 (2)
C6'—N1'	1.349 (18)	F12—P1	1.478 (18)
C6'—H6'	0.93	F13—P1	1.530 (18)
C51—C55	1.43 (2)	F14—P1	1.561 (17)
C51—C56	1.46 (2)	F15—P1	1.55 (2)
C51—C52	1.47 (2)	F16—P1	1.50 (2)
C51—Ir1	2.152 (14)	Cl1—Ir1	2.393 (4)

C52—C53	1.41 (2)		
O5"—C1"—C3	104.3 (13)	C51—C55—Ir1	69.6 (8)
O5"—C1"—C2"	109.4 (13)	C54—C55—Ir1	70.8 (8)
C3—C1"—C2"	111.8 (14)	C60—C55—Ir1	126.3 (11)
O5"—C1"—H1"	110.4	C51—C56—H56A	109.5
C3—C1"—H1"	110.4	C51—C56—H56B	109.5
C2"—C1"—H1"	110.4	H56A—C56—H56B	109.5
O2"—C2"—C3"	113.5 (14)	C51—C56—H56C	109.5
O2"—C2"—C1"	107.4 (13)	H56A—C56—H56C	109.5
C3"—C2"—C1"	110.0 (15)	H56B—C56—H56C	109.5
O2"—C2"—H2"	108.6	C52—C57—H57A	109.5
C3"—C2"—H2"	108.6	C52—C57—H57B	109.5
C1"—C2"—H2"	108.6	H57A—C57—H57B	109.5
N1'—C2'—C3'	122.7 (16)	C52—C57—H57C	109.5
N1'—C2'—C5	111.2 (13)	H57A—C57—H57C	109.5
C3'—C2'—C5	126.0 (15)	H57B—C57—H57C	109.5
N2—C3—N4	113.3 (15)	C53—C58—H58A	109.5
N2—C3—C1"	123.3 (16)	C53—C58—H58B	109.5
N4—C3—C1"	123.1 (15)	H58A—C58—H58B	109.5
N2—C3—C5	74.8 (12)	C53—C58—H58C	109.5
N4—C3—C5	38.5 (9)	H58A—C58—H58C	109.5
C1"—C3—C5	161.0 (14)	H58B—C58—H58C	109.5
O3"—C3"—C2"	107.1 (17)	C54—C59—H59A	109.5
O3"—C3"—C4"	110.9 (15)	C54—C59—H59B	109.5
C2"—C3"—C4"	113.0 (14)	H59A—C59—H59B	109.5
O3"—C3"—H3"	108.6	C54—C59—H59C	109.5
C2"—C3"—H3"	108.6	H59A—C59—H59C	109.5
C4"—C3"—H3"	108.6	H59B—C59—H59C	109.5
C2'—C3'—C4'	118.9 (17)	C55—C60—H60A	109.5
C2'—C3'—H3'	120.5	C55—C60—H60B	109.5
C4'—C3'—H3'	120.5	H60A—C60—H60B	109.5
O4"—C4"—C3"	113.2 (17)	C55—C60—H60C	109.5
O4"—C4"—C5"	106.0 (15)	H60A—C60—H60C	109.5
C3"—C4"—C5"	110.6 (15)	H60B—C60—H60C	109.5
O4"—C4"—H4"	109.0	C6'—N1'—C2'	118.0 (13)
C3"—C4"—H4"	109.0	C6'—N1'—Ir1	124.9 (11)
C5"—C4"—H4"	109.0	C2'—N1'—Ir1	117.2 (10)
C5'—C4'—C3'	119.0 (15)	C3—N2—O1	105.7 (12)
C5'—C4'—H4'	120.5	C5—N4—C3	97.3 (14)

C3'—C4'—H4'	120.5	C5—N4—Ir1	116.1 (12)
N4—C5—O1	122.9 (15)	C3—N4—Ir1	146.6 (11)
N4—C5—C2'	118.2 (16)	C5—O1—N2	100.7 (11)
O1—C5—C2'	118.1 (13)	C2"—O2"—H2O	95 (10)
N4—C5—C3	44.1 (8)	C3"—O3"—H3O	121 (10)
O1—C5—C3	78.8 (10)	C4"—O4"—H4O	111 (10)
C2'—C5—C3	160.9 (14)	C1"—O5"—C5"	114.1 (13)
O5"—C5"—C6"	106.3 (14)	C6"—O6"—H6O	144 (10)
O5"—C5"—C4"	109.6 (14)	F12—P1—F11	95.5 (18)
C6"—C5"—C4"	111.7 (16)	F12—P1—F16	93.1 (17)
O5"—C5"—H5"	109.7	F11—P1—F16	92.6 (14)
C6"—C5"—H5"	109.7	F12—P1—F13	87.4 (13)
C4"—C5"—H5"	109.7	F11—P1—F13	175.6 (16)
C4'—C5'—C6'	120.3 (16)	F16—P1—F13	84.0 (15)
C4'—C5'—H5'	119.9	F12—P1—F15	173 (2)
C6'—C5'—H5'	119.9	F11—P1—F15	89.0 (17)
O6"—C6"—C5"	109.5 (16)	F16—P1—F15	92.1 (17)
O6"—C6"—H6"A	109.8	F13—P1—F15	88.3 (15)
C5"—C6"—H6"A	109.8	F12—P1—F14	89.2 (14)
O6"—C6"—H6"B	109.8	F11—P1—F14	90.4 (12)
C5"—C6"—H6"B	109.8	F16—P1—F14	176.0 (16)
H6"A—C6"—H6"B	108.2	F13—P1—F14	92.9 (13)
N1'—C6'—C5'	121.0 (16)	F15—P1—F14	85.3 (14)
N1'—C6'—H6'	119.5	N1'—Ir1—C52	96.7 (5)
C5'—C6'—H6'	119.5	N1'—Ir1—C51	103.3 (5)
C55—C51—C56	126.1 (14)	C52—Ir1—C51	40.1 (5)
C55—C51—C52	105.9 (13)	N1'—Ir1—C53	123.8 (5)
C56—C51—C52	127.5 (13)	C52—Ir1—C53	38.2 (6)
C55—C51—Ir1	72.0 (8)	C51—Ir1—C53	65.7 (6)
C56—C51—Ir1	129.6 (11)	N1'—Ir1—N4	76.3 (5)
C52—C51—Ir1	69.8 (8)	C52—Ir1—N4	123.6 (6)
C53—C52—C51	108.3 (12)	C51—Ir1—N4	163.7 (4)
C53—C52—C57	127.9 (15)	C53—Ir1—N4	100.7 (5)
C51—C52—C57	123.8 (14)	N1'—Ir1—C54	161.3 (6)
C53—C52—Ir1	71.3 (8)	C52—Ir1—C54	64.9 (6)
C51—C52—Ir1	70.1 (8)	C51—Ir1—C54	65.4 (6)
C57—C52—Ir1	124.9 (10)	C53—Ir1—C54	38.9 (6)
C52—C53—C54	108.9 (14)	N4—Ir1—C54	110.3 (6)
C52—C53—C58	125.7 (15)	N1'—Ir1—C55	138.5 (6)
C54—C53—C58	124.8 (15)	C52—Ir1—C55	64.7 (6)

C52—C53—Ir1	70.5 (9)	C51—Ir1—C55	38.4 (5)
C54—C53—Ir1	71.5 (8)	C53—Ir1—C55	64.3 (6)
C58—C53—Ir1	130.5 (12)	N4—Ir1—C55	145.2 (6)
C55—C54—C53	106.8 (13)	C54—Ir1—C55	38.2 (6)
C55—C54—C59	126.8 (16)	N1'—Ir1—Cl1	86.5 (4)
C53—C54—C59	126.1 (16)	C52—Ir1—Cl1	149.8 (5)
C55—C54—Ir1	70.9 (8)	C51—Ir1—Cl1	109.9 (4)
C53—C54—Ir1	69.6 (8)	C53—Ir1—Cl1	149.8 (4)
C59—C54—Ir1	128.9 (12)	N4—Ir1—Cl1	86.4 (3)
C51—C55—C54	110.0 (13)	C54—Ir1—Cl1	111.0 (5)
C51—C55—C60	125.0 (15)	C55—Ir1—Cl1	93.3 (4)
C54—C55—C60	125.0 (15)		
O5"—C1"—C2"—O2"	179.2 (13)	C52—C53—C54—C55	0.7 (17)
C3—C1"—C2"—O2"	-65.8 (18)	C58—C53—C54—C55	-171.5 (15)
O5"—C1"—C2"—C3"	55.3 (17)	Ir1—C53—C54—C55	61.6 (10)
C3—C1"—C2"—C3"	170.2 (14)	C52—C53—C54—C59	175.2 (15)
O5"—C1"—C3—N2	109.0 (16)	C58—C53—C54—C59	3 (3)
C2"—C1"—C3—N2	-9 (2)	Ir1—C53—C54—C59	-123.9 (17)
O5"—C1"—C3—N4	-64.3 (18)	C52—C53—C54—Ir1	-60.9 (10)
C2"—C1"—C3—N4	177.6 (12)	C58—C53—C54—Ir1	127.0 (16)
O5"—C1"—C3—C5	-51 (5)	C56—C51—C55—C54	174.3 (15)
C2"—C1"—C3—C5	-169 (4)	C52—C51—C55—C54	2.5 (16)
O2"—C2"—C3"—O3"	64.8 (19)	Ir1—C51—C55—C54	-59.5 (10)
C1"—C2"—C3"—O3"	-174.9 (13)	C56—C51—C55—C60	-6 (2)
O2"—C2"—C3"—C4"	-172.8 (15)	C52—C51—C55—C60	-177.3 (14)
C1"—C2"—C3"—C4"	-52 (2)	Ir1—C51—C55—C60	120.7 (15)
N1'—C2'—C3'—C4'	2 (2)	C56—C51—C55—Ir1	-126.3 (16)
C5—C2'—C3'—C4'	-173.7 (16)	C52—C51—C55—Ir1	61.9 (9)
O3"—C3"—C4"—O4"	-69.2 (19)	C53—C54—C55—C51	-2.0 (17)
C2"—C3"—C4"—O4"	170.5 (15)	C59—C54—C55—C51	-176.5 (16)
O3"—C3"—C4"—C5"	172.0 (14)	Ir1—C54—C55—C51	58.7 (10)
C2"—C3"—C4"—C5"	52 (2)	C53—C54—C55—C60	177.8 (15)
C2'—C3'—C4'—C5'	-2 (3)	C59—C54—C55—C60	3 (3)
N1'—C2'—C5—N4	-2 (2)	Ir1—C54—C55—C60	-121.5 (15)
C3'—C2'—C5—N4	173.7 (16)	C53—C54—C55—Ir1	-60.7 (10)
N1'—C2'—C5—O1	-172.8 (14)	C59—C54—C55—Ir1	124.8 (18)
C3'—C2'—C5—O1	3 (2)	C5'—C6'—N1'—C2'	4 (2)
N1'—C2'—C5—C3	-22 (5)	C5'—C6'—N1'—Ir1	-176.9 (12)
C3'—C2'—C5—C3	153 (4)	C3'—C2'—N1'—C6'	-3 (2)

O4"—C4"—C5"—O5"	-176.0 (15)	C5—C2'—N1'—C6'	173.3 (13)
C3"—C4"—C5"—O5"	-53 (2)	C3'—C2'—N1'—Ir1	178.1 (12)
O4"—C4"—C5"—C6"	66 (2)	C5—C2'—N1'—Ir1	-5.8 (16)
C3"—C4"—C5"—C6"	-170.5 (15)	N4—C3—N2—O1	1.5 (16)
C3'—C4'—C5'—C6'	3 (3)	C1"—C3—N2—O1	-172.4 (14)
O5"—C5"—C6"—O6"	65.5 (17)	C5—C3—N2—O1	1.0 (10)
C4"—C5"—C6"—O6"	-175.0 (13)	O1—C5—N4—C3	0 (2)
C4'—C5'—C6'—N1'	-4 (3)	C2'—C5—N4—C3	-170.6 (14)
C55—C51—C52—C53	-2.0 (15)	O1—C5—N4—Ir1	179.1 (13)
C56—C51—C52—C53	-173.7 (15)	C2'—C5—N4—Ir1	8.9 (19)
Ir1—C51—C52—C53	61.4 (10)	C3—C5—N4—Ir1	179.5 (13)
C55—C51—C52—C57	177.3 (13)	N2—C3—N4—C5	-0.8 (16)
C56—C51—C52—C57	6 (2)	C1"—C3—N4—C5	173.1 (16)
Ir1—C51—C52—C57	-119.3 (13)	N2—C3—N4—Ir1	180.0 (11)
C55—C51—C52—Ir1	-63.4 (9)	C1"—C3—N4—Ir1	-6 (3)
C56—C51—C52—Ir1	125.0 (16)	C5—C3—N4—Ir1	-179 (2)
C51—C52—C53—C54	0.8 (16)	N4—C5—O1—N2	1 (2)
C57—C52—C53—C54	-178.4 (14)	C2'—C5—O1—N2	171.5 (13)
Ir1—C52—C53—C54	61.5 (11)	C3—C5—O1—N2	1.0 (10)
C51—C52—C53—C58	172.9 (15)	C3—N2—O1—C5	-1.6 (16)
C57—C52—C53—C58	-6 (2)	C3—C1"—O5"—C5"	179.2 (13)
Ir1—C52—C53—C58	-126.5 (16)	C2"—C1"—O5"—C5"	-61.1 (17)
C51—C52—C53—Ir1	-60.6 (9)	C6"—C5"—O5"—C1"	-179.1 (14)
C57—C52—C53—Ir1	120.1 (15)	C4"—C5"—O5"—C1"	60.0 (19)

Table S4. Hydrogen-bond geometry (Å, °) for **Ir-2**

D—H···*A*	*D*—H	H···*A*	*D*···*A*	*D*—H···*A*
C6′—H6′···O2"^{i}	0.93	2.5	3.159 (19)	128
O2"—H2*O*···F14	0.86 (1)	2.58 (19)	3.00 (2)	111 (15)
O4"—H4*O*···O6"^{ii}	0.86 (1)	1.92 (6)	2.77 (2)	170 (20)

Symmetry codes: (i) -*x*+1, *y*+1/2, -*z*+3/2; (ii) *x*-1/2, -*y*+3/2, -*z*+1.

3. **Table S5.** Distribution coefficient of the new complexes (logD)

Complex		LogD	Complex		logD
**Ru-1**	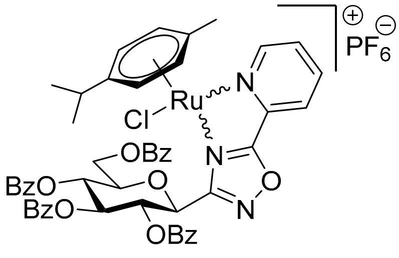	2.79	**Ir-2**	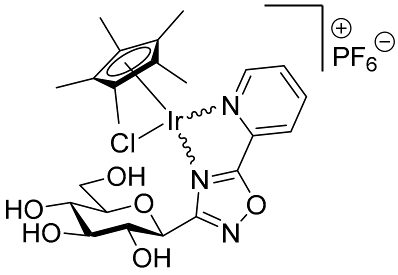	-1.15
**Ru-2**	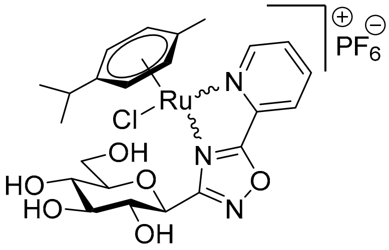	-0.96	**Ir-3**	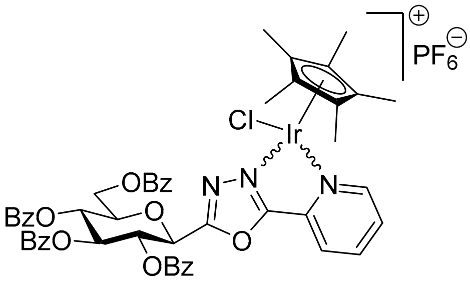	1.46
**Os-3**	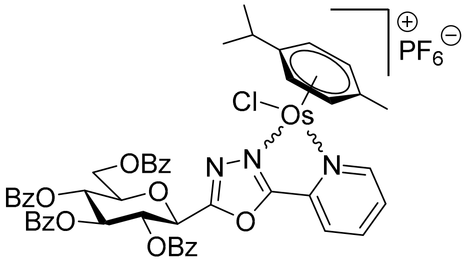	3.32	**Ir-4**	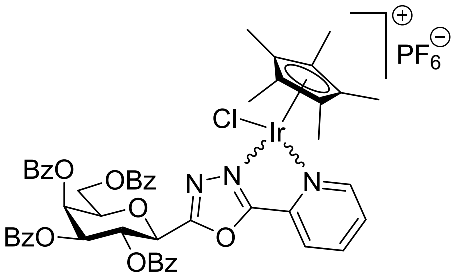	1.46
**Os-4**	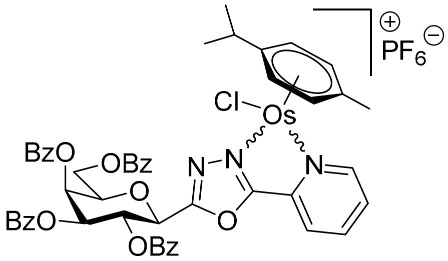	2.87	**Ir-5**	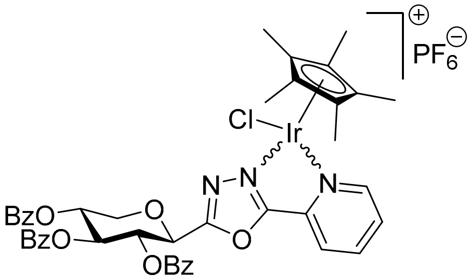	1.80
**Os-5**	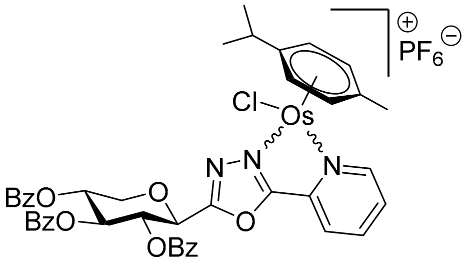	2.36	**Ir-6**	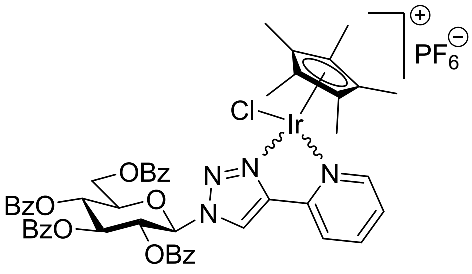	2.36

**Os-6**	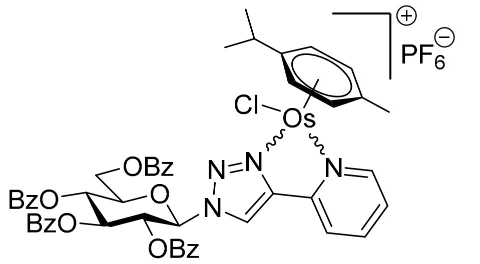	2.33	**Ir-7**	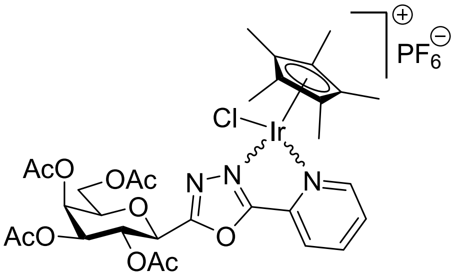	-0.75
**Rh-3**	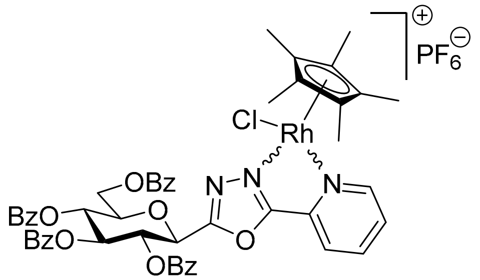	1.60	**Ir-8**	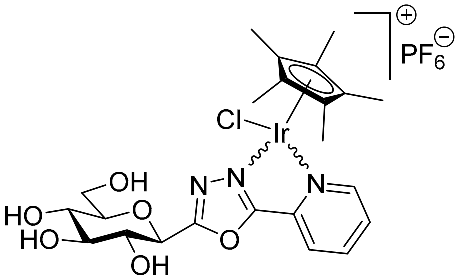	-1.60
**Rh-4**	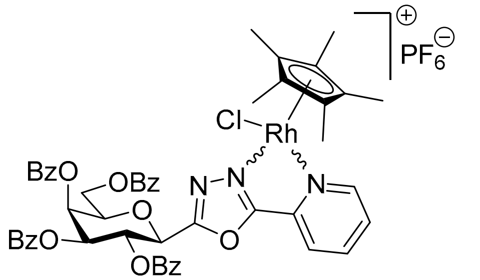	1.64	**Ir-9**	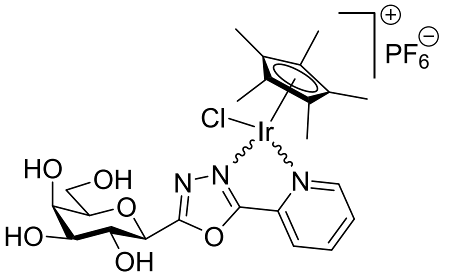	-1.47
**Rh-5**	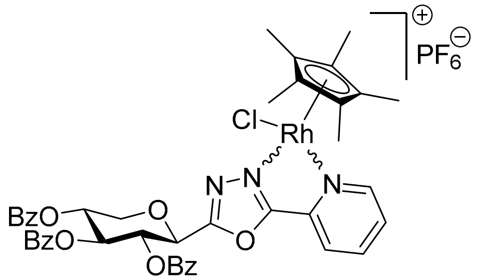	1.80	**Ir-10**	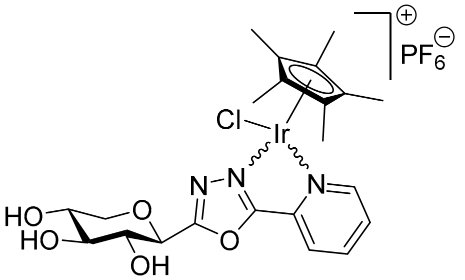	-1.56
**Rh-6**	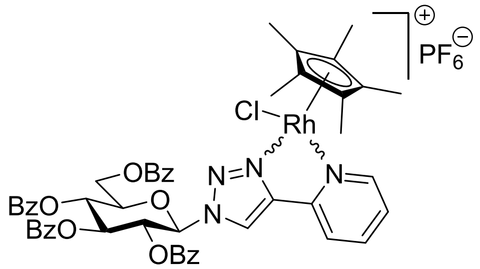	1.85	**Ir-11**	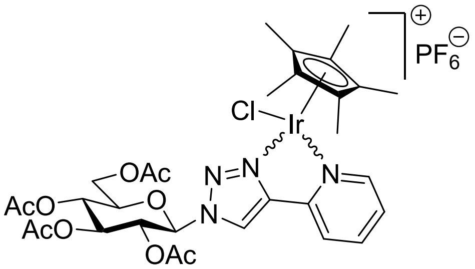	-1.31
**Ir-1**	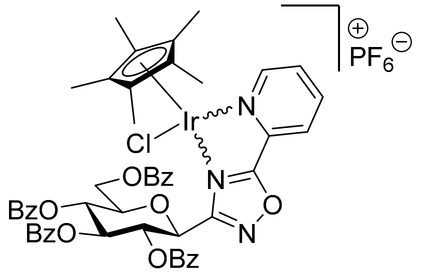	2.87	**Ir-12**	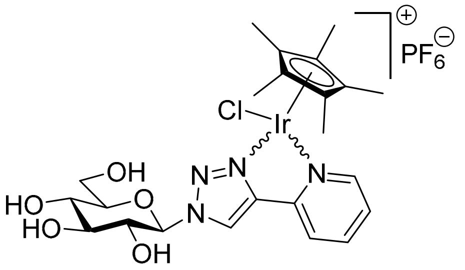	-2.08

4. References

Macrae, C. F., et al. (2006). "Mercury: visualization and analysis of crystal structures." Journal of Applied Crystallography **39**: 453-457.

Sheldrick, G. M. (2008). "A short history of SHELX." Acta Crystallographica a-Foundation and Advances **64**: 112-122.

Westrip, S. P. (2010). "publCIF: software for editing, validating and formatting crystallographic information files." Journal of Applied Crystallography **43**: 920-925.
